# Discovery of New Zosteropenillines from the Seagrass-Derived Fungus *Penicillium yezoense* KMM 4679 by OSMAC Strategy

**DOI:** 10.3390/md24060193

**Published:** 2026-05-30

**Authors:** Elena V. Leshchenko, Gleb V. Borkunov, Alexandr S. Antonov, Ekaterina A. Chingizova, Dmitrii V. Berdyshev, Maria A. Solovova, Roman S. Popov, Ksenia A. Sayankina, Yuliya V. Khudyakova, Sergey N. Baldaev, Natalya Yu. Kim, Anatoly I. Kalinovsky, Andrey V. Gerasimenko, Ekaterina A. Yurchenko, Anton N. Yurchenko

**Affiliations:** 1 G.B. Elyakov Pacific Institute of Bioorganic Chemistry, Far Eastern Branch of the Russian Academy of Sciences, 159 Prospect 100-Letiya Vladivostoka, Vladivostok 690022, Russia; 2 Institute of High Technologies and Advanced Materials, Far Eastern Federal University, Vladivostok 690922, Russia; 3 Institute of Chemistry, Far Eastern Branch of the Russian Academy of Sciences, 159 Prospect 100-Letiya Vladivostoka, Vladivostok 690022, Russia

**Keywords:** marine-derived fungi, *Penicillium yezoense*, OSMAC strategy, secondary metabolites, antimicrobial activity, cytotoxic activity, cardioprotective effects, biofilm formation

## Abstract

Thirteen new decaline polyketides, namely, zosteropenillines T–W (**1**–**4**), 8-hydroxypallidopenilline A (**5**), 13-*epi*-zosteropenilline P (**6**), 11-*epi*-zosteropenilline N (**7**), 15-hydroxyzosteropenilline M (**8**), 8-hydroxyzosteropenilline M (**9**), 11-*epi*-zosteropenilline M (**10**), and zosteropenillines X–Z (**11**–**13**), along with 17 known related compounds (**14**–**30**) were isolated from the ethyl acetate extract of the marine-derived fungus *Penicillium yezoense* KMM 4679 cultivated on MgCl_2_-containing nutrient medium. The structures of the isolated compounds were established based on spectroscopic methods. The absolute configurations of zosteropenillines T (**1**) and V (**3**) were determined using time-dependent density functional theory (TD-DFT) calculations of the ECD spectra. X-ray diffraction analysis data were obtained for the known zosteropenilline S (**28**). A biogenetic pathway for **1**–**13** was proposed. The effects of the compounds on *Staphylococcus aureus* and *Candida albicans* growth and biofilm formation were observed. Zosteropenillines U (**2**), Y (**12**) and Z (**13**) with higher activity against *C. albicans* biofilms were nontoxic for normal cardiomyocyte H9c2 cells, making them promising anti-candidal agents. Moreover, zosteropenillines U and Y demonstrated cardioprotective effects in acute ischemia/reperfusion and CoCl_2_-mimicking hypoxia in vitro models.

## 1. Introduction

Over the past decade, there has been a significant increase in the number of new marine natural products (MNPs) discovered from marine-derived fungi, as reported by Carroll and co-authors [1]. Fungal MNPs have pharmaceutical potential and play a crucial role in the development of new drugs [2,3,4]. However, biosynthetic gene clusters (BGCs) of fungi encoding the production of enzymes involved in the biosynthesis of secondary metabolites may be inactive (“silent”) or expressed at low levels under standard conditions of cultivation and fermentation. This often results in the discovery of the same MNPs from different strains. The rate of finding new fungal MNPs has slowed down. The most effective way to activate “silent” BGCs has been found to be the targeted modification of culture conditions using the One Strain Many Compounds (OSMAC) strategy [5,6]. One of the applications of this strategy is the addition of metal ions, which play a significant role in determining the structural and enzymatic properties of microorganisms [7]. Thus, the addition of MgCl_2_ to the culture medium led to the discovery of the sesquiterpenoid (−)-6-O-demethylpestalothiopsin B from *Ascotricha* sp. ZJ-M5. However, the authors concluded that adding MgCl_2_ to the medium inhibited the formation of other caryophyllene sesquiterpenes produced by this fungus [8]. The addition of Fe_2_(SO_4_)_3_ to a rice medium while cultivating the fungus *Stachybotrys chartarum* TJ403-SS6 resulted in the discovery of six novel phenylspirodrimane dimers—distachydrimanes A–F—with anticancer potential [9]. The usefulness of the metal addition strategy for activating “silent” BGCs in *Penicillium* sp. BB1122 cultivated with cobalt resulted in the discovery of unique compounds with antimicrobial properties, such as neocitreoviridine, 10z-isocitreoviridinol, penicillstressol, and isopenicillstressol [10]. Cultivation of *Streptomyces pratensis* NA-ZhouS1 at 100 μM NiCl_2_ resulted in the production of two new aromatic polyketides, namely, stremycin A and B, with antimicrobial action [11]. A recent use of this approach to cultivate the fungus *Alternaria* sp. ZH-15 on a solid agar medium with the addition of various inorganic additives (KBr, NaCl, MgCl_2_, CaCl_2_, KCl, SrCl_2_, Na_2_SO_4_, NaHCO_3_, and H_3_BO_3_) led to the discovery of a new drimane meroterpenoid, territrem F, bearing a unique borate ring system, which has potential for antiepileptic drug research [12]. Thus, the addition of inorganic components to the culture medium can both affect the activation of “silent” BGCs and serve as additional sources of chemical elements for incorporation into the structure of secondary metabolites.

Fungi of the genus *Penicillium* are among the most widespread terrestrial and marine fungal life forms on the planet [13]. The species belonging to *Penicillium* genus section *Aspergilloides* also have a wide distribution, with the best-known species being *P*. *thomii*, *P*. *glabrum* and *P*. *spinulosum* [14]. Earlier, we reported that *Aspergilloides* section *Thomiorum* series includes the 12 species *P*. *thomii*, *P*. *yezoense*, *P*. *fusisporum*, *P*. *aurantioviolaceum*, *P*. *valentinum*, *P*. *roseoviride*, *P*. *cartierense*, *P*. *contaminatum*, *P*. *austroafricanum*, *P*. *crocicola*, *P*. *grevilleicola* and *P*. *jejuense* [15]. Fungi belonging to the *Thomiorum* series have not been studied from an MNP perspective, except for *P*. *thomii* and *P*. *yezoense*. From the fungi *P*. *thomii*, which is associated with the seagrass *Zostera marina* and brown algae *Sargassum miyabei* and *S*. *pallidum* (Sea of Japan), our scientific group isolated 10 new meroterpenoids austalides [16]; 21 new decalin-type polyketides, pallidopenillines and zosteropenillines [17,18]; 6 new spiroketal polyketides, sargassopenillines [19]; 5 new eudesmane-type sesquiterpenes, thomimarines [20,21]; 2 known guaiane-type sesquiterpenes [21]; and 1 prenylated indole alkaloid [22]. A new xanthone dimer penicillixanthone A [23], new naphthoquinone derivatives penithoketone, 20 new chromone derivatives penithochromones [24,25], new meroterpenoid austalide Y, and two new labdane-type diterpenoids [26] have been discovered from the deep-sea-derived fungus *P*. *thomii*. In summary, the fungi of the *Thomiorum* series are rich sources of terpenes, polyketides, and meroterpenoids. In our previous research, it was shown that the fungus *P*. *yezoense* KMM 4679, isolated from the rhizosphere of the seagrass *Zostera marina* (Sea of Japan), produces ten new decalin-type polyketides zosteropenillines and pallidopenillines [15], together with four known analogs previously isolated from *P*. *thomii*. Moreover, it was shown that 1-acetylpallidopenilline A exhibits strong inhibition of human breast cancer MCF-7 cell colony formation [15].

However, the effect of inorganic salt additives on secondary metabolite production by *Penicillium yezoense* KMM 4679 has not been previously reported. To address this gap, we performed a targeted screening of nontoxic inorganic salts (excluding heavy metals) to evaluate their influence on the metabolic profile of this strain. During preliminary screening, the addition of MgCl_2_ was found to induce distinct alterations in the HPLC metabolic profile of the fungal extract (Figure S142). Thus, a culture medium with added MgCl_2_ was selected for the fermentation and isolation of MNPs.

**Figure 1:**
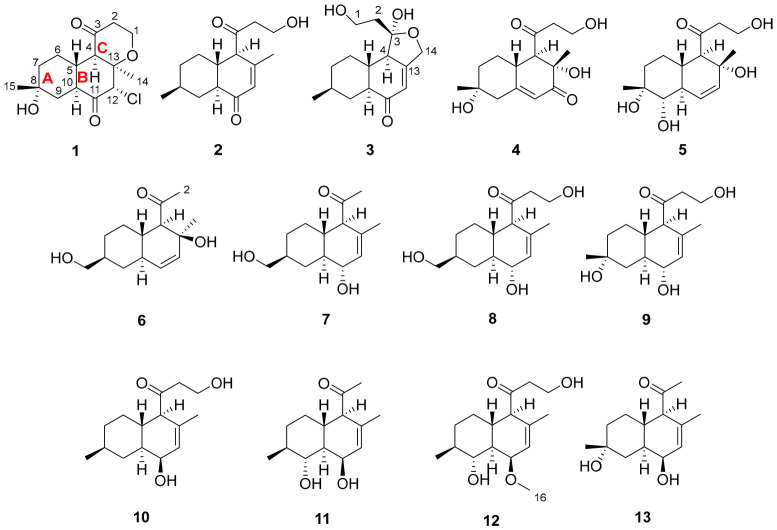
Metabolites isolated from *Penicillium yezoense* KMM 4679.

Thus, in the current study, the OSMAC strategy in part with inorganic additives was applied to *P. yezoense* KMM 4679 to continue the search for new MNPs. Our investigation resulted in the discovery of a series of 13 new decalin polyketides known as zosteropenillines (**1**–**13**) (Figure 1) in addition to 17 known zosteropenillines (**14**–**30**) (Figure S1) previously isolated from the *P*. *yezoense* and *P*. *thomii* fungi under normal cultivation conditions [15,17,18]. Herein, we describe the isolation, structure elucidation, and biological assay results of 30 decalin polyketides produced by *P*. *yezoense* KMM 4679 using the OSMAC approach.

## 2. Results and Discussion

### 2.1. Structure Elucidation

**Figure 2:**
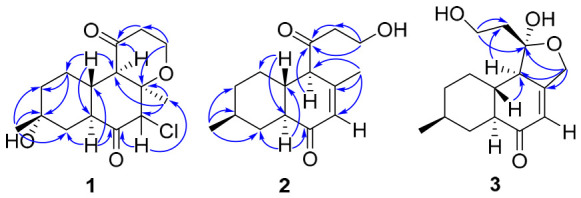
Selected HMBC correlations of **1**–**3.**

The molecular formula of **1** was determined to be C_15_H_21_ClO_4_ by the HRESIMS peak at *m/z* 323.1028 [M + Na]^+^ (Figure S3). A close inspection of the ^1^H and ^13^C NMR and HSQC data for **1** (Table 1 and Table 2, Figures S4–S6) revealed the presence of two methyl (δ_H_ 1.39, δ_C_ 19.7 and δ_H_ 1.29, δ_C_ 31.6) groups, five methylenes (δ_C_ 25.9, δ_C_ 37.2, δ_C_ 37.3, δ_C_ 37.7) including one oxygen-bearing (δ_H_ 4.19, 4.00, δ_C_ 61.6) and four *sp^3^*-methines (δ_H_ 3.33, δ_C_ 41.0; δ_H_ 1.97, δ_C_ 41.2; δ_H_ 2.64, δ_C_ 58.5; δ_H_ 3.88, δ_C_ 62.8), two quaternary oxygen-bearing *sp^3^*-carbons (δ_C_ 68.9, δ_C_ 82.4) and two quaternary *sp^2^*-carbons (δ_C_ 203.9, δ_C_ 207.9). These data and the five degrees of unsaturation from the molecular formula suggested that **1** possessed three rings and two carbonyl groups. The ^1^H-^1^H COSY data (Figures S2 and S7) and HMBC correlations (Figure 2 and Figure S8) H-4 (δ_H_ 2.64)/C-5, C-6; H-5 (δ_H_ 1.97)/C-4, C-6, C-7, C-10; H_2_-6 (δ_H_ 1.67, 1.34)/C-5, C-7, C-8; H_3_-15 (δ_H_ 1.29)/C-7, C-8, C-9; H-9a (δ_H_ 1.90)/C-7, C-10, C-15; H-9b (δ_H_ 1.49)/C-7, C-10, C-11, C-15; H-10 (δ_H_ 3.33)/C-4, C-5, C-6, C-9, C-11; H-12 (δ_H_ 3.88)/C-10, C-4, C-11, C-13, C-14; H_3_-14 (δ_H_ 1.39)/C-4, C-13, and C-12 revealed the presence of a decalin moiety (A and B rings) (Figure 1) and established the location of the methyl groups at C-8 and C-13, the location of the oxygen function at C-8 and C-13, and the location of the carbonyl at C-11 in **1**. The ^1^H-^1^H COSY data (Figures S2 and S7) and HMBC correlations Figure 2 and Figure S8) H-1a (δ_H_ 4.19)/C-2, C-13; H_2_-2 (δ_H_ 2.73, 2.20)/C-1, C-3; H-4/C-3 established a saturated *γ*-pyrone derivative moiety (C ring) in **1**.

**Figure 3:**
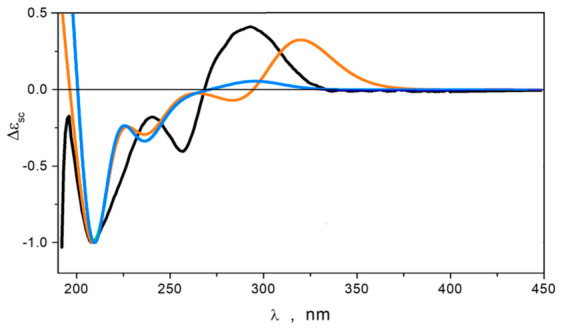
The experimental and calculated ECD spectra of **1**. Black—experimental, blue—calculated for 4*R*,5*S*,8*R*,10*S*,12*R*,13*R*, orange—calculated for 4*R*,5*S*,8*R*,10*S*,12*S*,13*R*. Different values of the UV shift were used for different stereoisomers: Δλ = +10 nm for 12*S* and Δλ = +20 nm for 12*R.*

The planar structure of **1** is very similar to those of zosteropenillines A–E [18] and peaurantiogriseol E [27], which contain a *γ*-pyrone moiety (ring C). According to the literature data, the cyclohexanone ring B with carbonyl at C-11 is present in the following isolated decalin polyketides: betaenones B–F [28,29] isolated from the fungus of *Phoma betae*, stemphyloxin I isolated from the fungus *Stemphylium botryosum* [30], 10-hydroxy-18-methoxylbetaenone from the fungus *Microsphaeropsis* sp. [31] and eujavanicol C from *Eupenicillium javanicum* [32]. Chlorine atoms have previously been found at C-11 in decalin polyketides, such as zosteropenilline S isolated from the fungus *Penicillum yezoense* KMM 4679 [15], pallidopenilline E isolated from the fungus *Penicillium thomii* KMM 4675 [17], and libertalide I–K isolated from the fungus *Libertasomyces* sp. [33]. The combination of a carbonyl at C-11 and a chlorine atom at C-12 in ring B and a *γ*-pyrone ring C was found for the first time in zosteropenilline and pallidopenilline-type decalin polyketides. The relative configurations of **1** were assigned based on a ROESY experiment and the ^1^H-^1^H coupling constants (Table 2). The observed ROESY correlations (Figure S9) between H_3_-15 (δ_H_ 1.29)/H-5 (δ_H_ 1.97); H-4 (δ_H_ 2.64)/H-10 (δ_H_ 3.33) and H_3_-14 (δ_H_ 1.39); and the magnitudes of the vicinal coupling constants H-4 (*J* = 11.5 Hz), H-5 (*J* = 11.8, 3.3 Hz), H-10 (*J* = 11.7, 3.3 Hz), and H-9a (*J* = 14.3, 3.0 Hz) (Table 2) indicated the *trans*-fusion of the A and B rings, as well as the B and C rings, *β*-orientation of H-5 and H_3_-15, and *α*-orientation of H-4, H_3_-14, and H-10. The presence of ROESY correlations H-12 (δ_H_ 3.88)/H-1a (δ_H_ 4.19) and H_3_-14/H-1b (δ_H_ 4.00) allowed us to assume the *β*-orientation H-12. On the other hand, the observed ROESY cross-peak H-12/H_3_-14 contradicts the *erythro* stereoconfiguration. The absolute configuration of **1** was calculated by quantum-chemical TD-DFT calculation of its ECD spectrum [15]. The performed conformational rearrangements accounted for the inversion of all rings in **1**. A comparison of the theoretical ECD spectra for the 12*S* and 12*R* stereoisomers of **1** with the corresponding experimental ECD spectrum is shown in Figure 3. It is seen that the absolute stereochemistry determination of **1** based only on the ECD spectroscopy data is not clear. Thus, only the position of the most intensive negative band can be described correctly for both stereoisomers, while the characteristics of the two other bands (at λ ≈ 265 nm and λ ≈ 300 nm) cannot. The 4*R*,5*S*,8*R*,10*S*,12*R*,13*R* stereoisomer looks to some extent like a preferrable candidate, since the position of the band at λ ≈ 300 nm was described correctly, but the intensity of this band is very underestimated in the calculated spectrum relative to the experimental one. The limitations of ECD spectroscopy are well recognized, particularly for chiral compounds exhibiting characteristic bands at λ ≤ 220 nm, where experimental constraints such as pronounced solvent absorption compromise the spectral quality [20,34]. This issue is especially characteristic of flexible, saturated chiral systems with freely rotating substituents, where the absence of chromophores yields weak Cotton effects, thereby hindering reliable stereochemical assignment. In such cases, complementary chiroptical techniques can provide critical structural insights.

The theoretical NMR data, calculated with the cam- B3LYP/6-311G(d)_GIAO_PCM method for stereoisomers of **1**, were compared to the experimental ones (Table S4, Figures S140 and S141). The calculated chemical shifts δ_C_ are systematically overestimated by about 3.8 and 6.2 ppm for *S*-12 and *R*-12, respectively. However, as one can see from the linear regression analysis results, the theoretical data reproduce well the relative positions of signals in the spectra and are better for the *S*-12 stereoisomer (Figure S141). The root mean square deviation (SD) obtained for *S*-12 is ~2.3 ppm, which is approximately half of what was obtained for *R*-12 (5.4 ppm). Such values of the systematic errors and SD values are in the range of the standard practice for quantum-chemical modeling at DFT or the MP2 level of theory using basis sets of mediu m quality (such as the 6-311G(d)) and even using the cc-pvTz basis set. The theoretical values δ_C_ of C-13 for both stereoisomers are reproduced with one and the same quality, while the value δ_C_ of C-12 significantly stand out from the linear regression line for *R*-12. This tendency is maintained when the cc-pvTz basis set was used for modeling.

**Figure 4:**
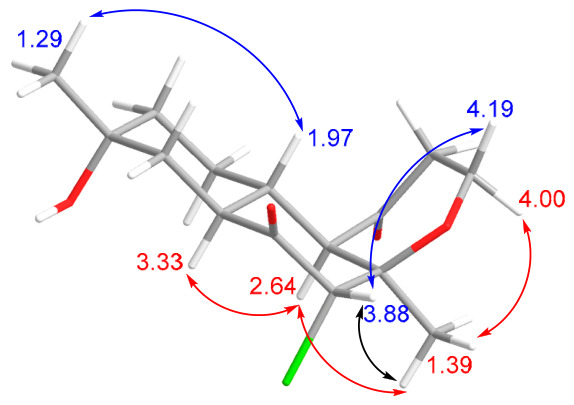
Selected ROESY correlations of **1** (conformer calculated using ECD and theoretical NMR)**.**

The combined exploration of ECD and NMR theoretical investigations was used earlier for solving stereochemical problems in previous works [35,36]. As it was shown in [35], the intramolecular transformations, leading to *S*/*R*-isomerization, may result in the changing of the sign of the relative chemical shift for nuclei, from neighboring to stereocenter under study. In present work, the effect is pronounced too, but without changing the sign. The complete set of presented data allows us to choose *S*-12 as a more preferrable candidate for stereochemical assignment. The proposed conformer is fully consistent with the ROESY spectrum data (Figure 4). This allowed us to confirm that compound **1** has an 4*R*,5*S*,8*R*,10*S*,12*S*,13*R* absolute configuration. Thus, the chemical structure of **1** was established and named zosteropenilline T.

The molecular formula of **2** was established to be C_15_H_22_O_3_ by the HRESIMS peak at *m/z* 273.1471 [M + Na]^+^ (Figure S13). A close inspection of the ^1^H and ^13^C NMR, DEPT, and HSQC data for **2** (Table 1 and Table 2, Figures S14–S17) revealed the presence of two methyl (δ_H_ 1.84, δ_C_ 21.8 and δ_H_ 0.94, δ_C_ 22.4) groups, five methylenes (δ_C_ 31.4, δ_C_ 33.7, δ_C_ 33.8, δ_C_ 44.9) including one oxygen-bearing (δ_H_ 3.90, δ_C_ 57.4), four *sp^3^*-methines (1.42, δ_C_ 31.7; δ_H_ 1.96, δ_C_ 41.7; δ_H_ 1.99, δ_C_ 48.6; δ_H_ 3.23, δ_C_ 61.6), one *sp^2^*-methine (δ_H_ 5.97, δ_C_ 128.8) group, and three quaternary *sp^2^*-carbons (δ_C_ 154.7, δ_C_ 199.1, δ_C_ 212.0). These data and the five degrees of unsaturation from the molecular formula suggested that **2** possessed two rings, one double bond, and two carbonyl groups. The ^1^H-^1^H COSY data (Figures S12 and S18) and HMBC correlations (Figure 2 and Figure S19) H-4 (δ_H_ 3.23)/C-5, C-6, C-12, C-13; H-5 (δ_H_ 1.96)/C-3, C-6, C-10; H_3_-15 (δ_H_ 0.94)/C-7, C-8, C-9; H-10 (δ_H_ 1.99)/C-4, C-5, C-9, C-11; H-12 (δ_H_ 5.97)/C-10, C-4, C-14; H_3_-14 (δ_H_ 1.84)/C-4, C-13, and C-12 revealed the presence of a decalin moiety (A and B rings) and established the location of the methyl groups at C-8 and C-13 and the location of the carbonyls at C-11 in **2**. The HMBC correlations H_2_-1 (δ_H_ 3.90)/C-2, C-3; H_2_-2 (δ_H_ 2.75, 2.71)/C-1, C-3; H-4/C-3 established the location of a 3-hydroxy-1-oxopropyl residue at C-4 in **2**. The relative configurations of **2** were assigned based on the ROESY (Figures S12 and S20) correlations H_3_-15 (δ_H_ 0.94)/H-5 (δ_H_ 1.96) and magnitudes ^1^H-^1^H coupling constants H-4 (*J* = 9.9 Hz), H-5 (*J* = 12.5, 10.0, 3.0 Hz), H-9b (*J* = 13.7, 3.2 Hz) and H-10 (*J* = 12.5, 3.5 Hz) (Table 2). Thus, the chemical structure of **2** was established and named zosteropenilline U.

**Figure 5:**
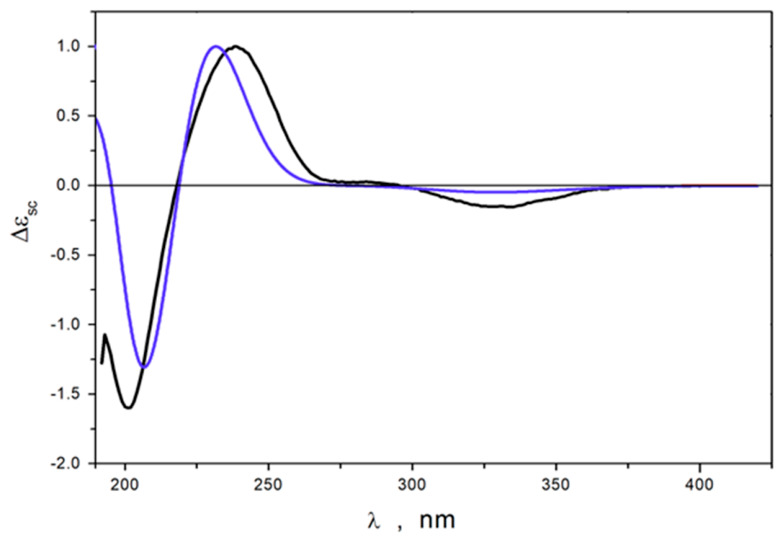
The experimental and calculated ECD spectra of **3**. Black color—experimental, blue color—calculated for 3*R*,4*R*,5*S*,8*S*,10*S*.

The molecular formula of **3** was established to be C_15_H_23_O_4_ by the HRESIMS peak at *m/z* 289.1418 [M + H]^+^ (Figure S24). The general features of the ^13^C NMR spectrum of **3** (Table 1 and Table 2) for the A ring (C-5–C-10), C-11 and C-15 resembled those of **2**. However, a close inspection of the ^1^H and ^13^C NMR, DEPT, and HSQC data (Figures S25–S28) for **3** revealed the presence of three different methylenes (δ_C_ 40.4) including two oxygen-bearing (δ_H_ 4.25, 3.96, δ_C_ 59.7; δ_H_ 4.64, 4.61, δ_C_ 67.3), one *sp^3^*-methine (δ_H_ 2.46, δ_C_ 55.4), one *sp^2^*-methine (δ_H_ 5.94, δ_C_ 120.4), one quaternary oxygen-bearing *sp^3^*-carbon (δ_C_ 106.3) and one quaternary *sp^2^*-carbon (δ_C_ 166.1). These data and the five degrees of unsaturation from the molecular formula suggested that **3** possessed three rings, one carbonyl group and one double bond. The ^1^H-^1^H COSY data (Figures S23 and S29) and HMBC correlations (Figure 2 and Figure S30) H-4 (δ_H_ 2.46)/C-10; H-5 (δ_H_ 1.92)/C-4, C-10; H-10 (δ_H_ 1.95)/C-4, C-11 established the B ring in the decalin moiety, the location of the carbonyl at C-11 and ∆^12^ in **3**. HMBC correlations H_2_-14 (δ_H_ 4.64, 4.61)/C-3, C-4, C-13; H-12 (δ_H_ 5.94)/C-14 established the tetrahydrofuran ring (C ring) and its attachment to C-4 and C-13 in **3**. HMBC correlations H_2_-1/C-2, C-3 established the hydroxyethyl fragment at C-3 in **3**. ROESY (Figures S23 and S31) correlations H_3_-15 (δ_H_ 0.95)/H-5 (δ_H_ 1.92); H-10 (δ_H_ 1.95)/H-4 (δ_H_ 2.46) together with the observed magnitude *^3^J* coupling constant H-4 (*J* = 9.9 Hz) indicated a *trans*-fusion of the A and B rings, an *α*-orientation H-10 and H-4, a *β*-orientation H_3_-15, H-5 and substituent at C-4 (ring C). It is worth noting that the identical chemical shift values of H-10 and H-2a make it difficult to determine the relative configuration of **3** using ROESY data. The statistically averaged theoretical ECD spectrum of 3*R*,4*R*,5*S*,8*S*,10*S* was compared with the experimental spectrum of **3** (Figure 5). A good correspondence between the two spectra was obtained in the whole diapason of *λ* values. Since the ECD spectrum for 3*S*,4*S*,5*R*,8*R*,10*R* is a mirror image of the ECD spectrum of 3*R*,4*R*,5*S*,8*S*,10*S*, this points out that the compound under study is 3*R*,4*R*,5*S*,8*S*,10*S*. Thus, the chemical structure of **3** was established and named zosteropenilline V.

The molecular formula of **4** was determined to be C_15_H_22_O_5_ by the HRESIMS peak at *m/z* 305.1367 [M + Na]^+^ (Figure S35). The general features of the ^1^H and ^13^C NMR spectra of **4** resembled those of zosteropenilline K [18] except for the C-6–C-9 and C-15 carbon signals and the H_2_-6, H_2_-7, H_2_-9, H_2_-15 proton signals (Table 1 and Table 2, Figures S36 and S37). These data, including DEPT, HSQC, HMBC, and ^1^H-^1^H COSY experiments (Figures S34, S38–S41), together with the same molecular formula as zosteropenilline K (*m/z* 305.1370 [M + Na]^+^) [18] indicated the presence of the methyl and hydroxyl groups at C-8 in **4** instead of a hydroxymethyl group in zosteropenilline K. The relative configurations of **4** were assigned based on ROESY (Figures S34 and S42) correlations H-5/H_3_-14 and the observed ^1^H-^1^H coupling constant H-4 (*J* = 9.7 Hz). Thus, the chemical structure of **4** was established and named zosteropenilline W.

The molecular formula of **5** was determined to be C_15_H_24_O_5_ by the HRESIMS peak at *m/z* 307.1521 [M + Na]^+^ (Figure S46). A close inspection of the ^1^H and ^13^C NMR, DEPT, and HSQC (Table 1 and Table 2, Figures S47–S50) data for **5** revealed the presence of two methyl (δ_H_ 1.19, δ_C_ 25.8; δ_H_ 1.27, δ_C_ 27.4) groups, four methylenes (δ_C_ 24.1, δ_C_ 37.5, δ_C_ 49.2) including one oxygen-bearing (δ_H_ 3.91, 3.86, δ_C_ 58.1), four *sp^3^*-methines (δ_H_ 1.59, δ_C_ 39.0; δ_H_ 2.16, δ_C_ 43.4; δ_H_ 2.96, δ_C_ 63.0) including one oxygen-bearing (δ_H_ 3.03, δ_C_ 77.2), two *sp^2^*-methine (δ_H_ 5.97, δ_C_ 127.4; δ_H_ 5.55, δ_C_ 134.9), two quaternary *sp^3^*-carbons (δ_C_ 71.5; δ_C_ 72.4) and one quaternary *sp^2^*-carbon (δ_C_ 213.9). The general features of the ^13^C NMR of **5** resembled those of pallidopenilline A [15] except for the C-6–C-10 and C-15 carbon signals and proton signals of the A ring and H_3_-15. The relative configurations of **5** were assigned based on ROESY correlations (Figures S45 and S53) H-9 (δ_H_ 3.03)/H-5 (δ_H_ 1.59)/H_3_-15 (δ_H_ 1.27); H-10 (δ_H_ 2.16)/H-4 (δ_H_ 2.96) and the observed ^1^H-^1^H coupling constants H-4 (*J* = 11.6 Hz), H-9 (*J* = 10.8 Hz) and H-10 (*J* = 10.7 Hz) (Table 2). Thus, compound **5** was named 8-hydroxypallidopenilline A.

The molecular formula of **6** was established to be C_14_H_22_O_3_ by the HRESIMS peak at *m/z* 261.1469 [M + Na]^+^ (Figure S57). A close inspection of the ^1^H and ^13^C NMR, DEPT, and HSQC data for **6** (Table 3 and Table 4, Figures S58–S61) revealed the presence of two methyl (δ_H_ 1.29, δ_C_ 28.9; δ_H_ 2.27, δ_C_ 34.6) groups, four methylenes (δ_C_ 29.2, δ_C_ 29.4, δ_C_ 35.1) including one oxygen-bearing (δ_H_ 3.47, 3.49, δ_C_ 68.2), four *sp^3^*-methines (δ_H_ 1.65, δ_C_ 40.6; δ_H_ 1.79, δ_C_ 39.6; δ_H_ 1.81, δ_C_ 42.2; δ_H_ 2.59, δ_C_ 61.6), two *sp^2^*-methine (δ_H_ 5.54, δ_C_ 132.9; δ_H_ 5.56, δ_C_ 132.5), one oxygen-bearing quaternary *sp^3^*-carbon (δ_C_ 69.4) and one quaternary *sp^2^*-carbon (δ_C_ 215.8). These data and the four degrees of unsaturation from the molecular formula suggested that **6** possessed two rings, one carbonyl group, and one double bond. The ^1^H-^1^H COSY data and HMBC correlations (Figures S56, S62 and S63) H-4 (δ_H_ 2.59)/C-3, C-5, C-10, C-13; H-5 (δ_H_ 1.79)/C-4, C-6, C-10, C-13; H_2_-15 (δ_H_ 3.49; 3.47)/C-7, C-8, C-9; H-9b (δ_H_ 0.92)/C-5, C-7, C-8, C-10, C-15; H-11 (δ_H_ 5.54)/C-5, C-9, C-10, C-13; H-12 (δ_H_ 5.56)/C-4, C-10, C-13; H_3_-14 (δ_H_ 1.29)/C-4, C-12, C-13 revealed the presence of a decalin moiety (A and B rings) and established the location of the oxygen-bearing methylene group at C-8, the location of the methyl C-13 and ∆^11^ in **6**. HMBC correlations H_3_-2 (δ_H_ 2.27)/C-3 and C-4 (δ_C_ 61.6) revealed the location of an acetyl group at C-4 in **6**. The planar structure of **6** was very similar to those obtained for zosteropenilline P, previously isolated from the *P. yezoense* KMM 4679 [15]. However, the chemical shift H_3_-14 (δ_H_ 1.29) of **6** was similar to the same methyl H_3_-14 (δ_H_ 1.30) with an *α*-orientation in previously isolated zosteropenilline F [17], while the chemical shifts H_3_-14 with a *β*-orientation in this polyketide series are always 1.19–1.20 ppm (in CDCl_3_) [15,18]. Additionally, ROESY (Figures S56 and S64) correlations H_3_-14/H-10 confirmed the *α*-orientation H_3_-14 in **6**. Moreover, according to the literature data, the [*α*]_D_^20^ = −54.3 for zosteropenilline P [15], while for **6**, it is the opposite [*α*]_D_^20^ = +48.1. Thus, compound **6** is an epimer of zosteropenilline P at C-13 and is named 13-*epi*-zosteropenilline P.

The molecular formula of **7** was determined to be C_14_H_22_O_3_ based on the HRESIMS peak at *m/z* 261.1467 [M + Na]^+^ (Figure S68). The ^1^H and ^13^C NMR spectra for **7** (Table 3 and Table 4, Figures S69 and S70) were very similar to those obtained for zosteropenilline N, previously isolated from *P. yezoense* KMM 4679 [15], except for the C-5, C-10 and C-11 carbon signals and the H_2_-9, H-10, H-11 and H-12 proton signals. These data, ^1^H-^1^H COSY cross-peaks (Figure S72) H-12/H-11, H-11/H-10, H-10/H_2_-9, H_2_-9/H-8, H-8/H_2_-7, H_2_-6/H-5, H-5/H-4, and H-10 and HMBC correlations (Figure S74) H_3_-14 (δ_H_ 1.60)/C-4, C-12, and C-13; H_2_-15 (δ_H_ 3.49 (2H))/C-7, C-8, and C-9; H-12 (δ_H_ 5.59)/C-4 and C-14; H_3_-2 (δ_H_ 2.10)/C-3 and C-4 established the A and B rings, the location of the hydroxyl group at C-11, the hydroxymethyl group at C-8, the acetyl group at C-4, and the ∆^12^ double bond. Thus, the planar structure of **7** was the same as that of zosteropenilline N. The observed ROESY correlations (Figures S67 and S75) between H-4 (δ_H_ 2.81)/H-10 (δ_H_ 1.18) and H-5 (δ_H_ 1.52)/H-11 (δ_H_ 3.90) together with the magnitude of the coupling constant H-11 (*J* = 9.0 Hz) indicated the *trans*-fusion of the A and B rings, the *β*-orientation of the acetyl group and the *α*-orientation of 11-OH. The coupling constant pattern of H-7b (*J* = 13.0, 3.5 Hz) indicates its axial orientation for H-6b, H-7b and H-8. This suggests a *β*-orientation hydroxymethylene substituent at C-8. According to the literature data, [*α*]_D_^20^ = −60.3 for zosteropenilline N with *β*-orientation of 11-OH [15], and the opposite, [*α*]_D_^20^ = + 57.0, is found for **7**; therefore, the structure of **7** was established as an epimer of zosteropenilline N at C-11. Thus, the chemical structure of **7** was established and named 11-*epi*-zosteropenilline N.

The molecular formula of **8** was determined to be C_15_H_24_O_4_ based on the HRESIMS peak at *m/z* 291.1572 [M + Na]^+^ (Figure S77). The general features of the ^13^C NMR spectrum of **8** resembled those of **7,** except for the C-1–C-3 carbon signals (Table 4, Figure S79). HMBC correlations (Figure S83) H_2_-1 (δ_H_ 3.85)/C-2, C-3; H_2_-2 (δ_H_ 2.70, 2.64)/C-1, C-3 established the location of a 3-hydroxy-1-oxopropyl residue at C-4 in **8**. Compound **8** was named 15-hydroxyzosteropenilline M.

The molecular formula of **9** was determined to be C_15_H_24_O_4_ based on the HRESIMS peak at *m/z* 291.1575 [M + Na]^+^ (Figure S86). The ^1^H and ^13^C NMR spectra of **9** (Table 3 and Table 4, Figures S87 and S88) were similar to those obtained for 11-*epi*-8-hydroxyzosteropenilline M, previously isolated from *P. yezoense* KMM 4679 [15], except for C-10–C-13. These data, along with the ^1^H-^1^H COSY data (Figure S91) and HMBC correlations (Figure S92) H-5 (δ_H_ 1.54)/C-10; H-6b (δ_H_ 1.48)/C-10; H-7b (δ_H_ 1.35)/C-5, C-6; H-9 (δ_H_ 2.21)/C-5, C-8, C-10; H-10 (δ_H_ 1.54)/C-5; H-11 (δ_H_ 3.86)/C-10; H_3_-14 (δ_H_ 1.60)/C-4, C-13, and C-12, revealed the presence of a decalin moiety and established the location of the methyl groups at C-8 and C-13, the location of the oxygen function at C-8 and C-11 and the ∆^12^ double bond in **9**. The chemical shift of C-11 (δ_C_ 72.4) in the ^13^C NMR spectra of **9** was similar to the chemical shift of C-11 (δ_C_ 72.6) in the ^13^C NMR spectra of zosteropenilline M [15]. The 11-OH group in zosteropenilline M was established to be in an *α*-orientation. According to the literature data for zosteropenilline M, [*α*]_D_^20^ = +161.4, and for 11-*epi*-8-hydroxyzosteropenilline M, [*α*]_D_^20^ = −57.6 [15], while the mean of the angle of rotation of the plane of polarization of light of compound **9** [*α*]_D_^20^ = +62.2. These data established the *α*-orientation of the 11-OH group in **9**. It is worth noting that the identical chemical shift values of H-10 and H-5, H_2_-1 and H-11 make it difficult to determine the relative configuration of **9** using ROESY data. Compound **9** was named 8-hydroxyzosteropenilline M.

The molecular formula of **10** was established to be C_15_H_24_O_3_ based on the HRESIMS peak at *m/z* 275.1627 [M + Na]^+^ (Figure S97). A close inspection of the ^1^H and ^13^C NMR, DEPT, and HSQC data for **10** (Table 5 and Table 6, Figures S98–S101) revealed the presence of two methyl (δ_H_ 0.92, δ_C_ 22.6 and δ_H_ 1.60, δ_C_ 21.5) groups, five methylenes (δ_C_ 31.8, δ_C_ 34.3, δ_C_ 36.8, δ_C_ 43.0) including one oxygen-bearing (δ_H_ 3.84, 3.83, δ_C_ 57.8), five *sp^3^*-methines (δ_H_ 1.74, δ_C_ 33.7; δ_H_ 1.44, δ_C_ 32.3; δ_H_ 1.27, δ_C_ 41.7; δ_H_ 2.75, δ_C_ 62.8; δ_H_ 3.88, δ_C_ 66.8), one sp^2^-methine (δ_C_ 127.4), and two quaternary *sp^2^*-carbon (δ_C_ 214.5, δ_C_ 134.9). These data and the four degrees of unsaturation from the molecular formula suggested that **10** possessed two rings, one carbonyl, and one double bond. The ^1^H-^1^H COSY data (Figures S96 and S102) and HMBC correlations (Figure S103) H-5 (δ_H_ 1.74)/C-4, C-6, C-10, C-11; H-6a (δ_H_ 1.72)/C-5; H-6b (δ_H_ 1.09)/C-4, C-5, C-8, C-10; H_3_-15 (δ_H_ 0.92)/C-7, C-8, C-9; H-9a (δ_H_ 1.60)/C-5, C-8, C-11, C-15; H-9b (δ_H_ 1.19)/C-5, C-8, C-10, C-11, C-15; H-10 (δ_H_ 1.27)/C-4, C-5, C-6, C-9; H-11 (δ_H_ 3.88)/C-5, C-9, C-10, C-12, C-13; H-12 (δ_H_ 5.87)/C-4, C-10, C-11, C-14; H_3_-14 (δ_H_ 1.60)/C-4, C-12, and C-13 revealed the presence of a decalin moiety and established the location of the methyl groups at C-8 and C-13, the location of the oxygen function at C-11 and C-13 and the location of the ∆^12^ in **10**. The ^1^H and ^13^C NMR spectra of **10** were very similar to those obtained for zosteropenilline M [15], except for C-5 and C-9–C-13. These data, together with a comparison of the angles of rotation of the plane of polarization of light ([*α*]_D_^20^ = +161.4 for zosteropenilline M and [*α*]_D_^20^ = +6.12 for **10**), a comparison of the difference in the chemical shifts of carbon C-11 (δ_C_ 66.8 for 10, δ_C_ 72.6 for zosteropenilline M), and a comparison of the coupling constants H-11 (*J* = 4.2, 3.3 Hz) in **10** and H-11 (*J* = 9.0 Hz) in zosteropenilline M established **10** as an epimer at C-11 of zosteropenilline M with 11-OH in the *β*-configuration. Compound **10** was named 11-*epi*-zosteropenilline M.

The molecular formula of **11** was determined to be C_14_H_22_O_3_ based on the HRESIMS peak at *m/z* 261.1467 [M + Na]^+^ (Figure S108), which is similar to that of compound **7**. A close inspection of the ^1^H and ^13^C NMR, DEPT, and HSQC data for **11** (Table 5 and Table 6, Figures S109–S112) revealed the presence of three methyl (δ_H_ 1.05, δ_C_ 18.5; δ_H_ 1.63, δ_C_ 21.3 and δ_H_ 2.13, δ_C_ 28.0) groups, two methylenes (δ_C_ 31.1, δ_C_ 32.2), six *sp^3^*-methines (δ_H_ 1.84, δ_C_ 33.3; δ_H_ 1.45, δ_C_ 39.1; δ_H_ 1.22, δ_C_ 48.2; δ_H_ 2.75, δ_C_ 62.9) including two oxygen-bearing (δ_H_ 3.37, δ_C_ 75.3; δ_H_ 4.41, δ_C_ 62.5), one *sp^2^*-methine (δ_H_ 3.88, δ_C_ 126.5) and two quaternary *sp^2^*-carbons (δ_C_ 210.9, δ_C_ 135.3). These data and the four degrees of unsaturation from the molecular formula suggest that **11** possesses two rings, one carbonyl, and one double bond. The ^1^H-^1^H COSY data (Figures S107 and S113) and HMBC correlations (Figure S114) H-5 (δ_H_ 1.84)/C-4, C-6, C-10; H_3_-15 (δ_H_ 1.05)/C-7, C-8, C-9; H-9 (δ_H_ 3.37)/C-7, C-8, C-10, C-15; H-10 (δ_H_ 1.22)/C-5, C-6, C-8; H-11 (δ_H_ 4.41)/C-5, C-9, C-10, C-13; H-12 (δ_H_ 5.88)/C-4, C-10, C-11, C-14; H_3_-14 (δ_H_ 1.63)/C-4, C-12, and C-13; H_3_-2 (δ_H_ 2.13)/C-3 and C-4 revealed the presence of a decalin moiety and established the location of the methyl groups at C-3, C-8, and C-13; the location of the acetyl group at C-4; the location of the oxygen function at C-9, C-11, and C-13; and the location of the ∆^12^ in **11**. ROESY correlations (Figures S107 and S115) H-5/H-9 and H-10/H-8 together with the value of the coupling constants H-9 (*J* = 9.9 Hz), H-11 (*J* = 4.6 Hz) and H-4 (*J* = 9.6 Hz) indicate the *α*-configuration of 9-OH and the *β*-configuration of 11-OH in **11**. Compound **11** was named zosteropenilline X.

The molecular formula of **12** was determined to be C_16_H_26_O_4_ based on the HRESIMS peak at *m/z* 305.1735 [M + Na]^+^ (Figure S117). The general features of the ^13^C NMR signals (Figure S119) of the A and B rings of **12** resembled those of **11**, except for C-11. HMBC correlations (Figures S116 and S123) H_3_-16 (δ_H_ 3.43)/C-11, H-12 (δ_H_ 6.02)/C-4, C-10, C-11, C-14, H-4 (δ_H_ 2.70)/C-3, H_2_-2 (δ_H_ 2.73, 2.58)/C-1 and C-3, and H_2_-1 (δ_H_ 3.83, 3.82)/C-2 and C-3 established the location of the methoxy group at C-11 and the location of the 3-hydroxy-1-oxopropyl residue at C-4. ROESY correlations H-5/H-9; 10/H-4 and H-8 together with the value of the coupling constants H-9 (*J* = 9.9 Hz), H-11 (*J* = 4.8, 3.4 Hz) and H-4 (*J* = 9.7 Hz) indicate the *α*-configuration of 9-OH and the *β*-configuration of 11-OMe in **12**. Compound **12** was named zosteropenilline Y.

The molecular formula of **13** was determined to be C_14_H_22_O_3_ based on the HRESIMS peak at *m/z* 261.1469 [M + Na]^+^ (Figure S128). The general features of the ^13^C NMR (Figure S130, Table 5) signals of the A and B rings of **13** resembled those of **9**, except for C-5, C-11, C-12, and C-13. The presence of a methyl group (δ_H_ 2.15, δ_C_ 28.6) suggested the location of the acetyl group at C-4. The value of the coupling constant H-11 (*J* = 5.8 Hz) and the chemical shift C-11 (δ*_C_* 66.7) indicated the *β*-configuration of 11-OH in **13**. Compound **13** was named zosteropenilline Z.

**Figure 6:**
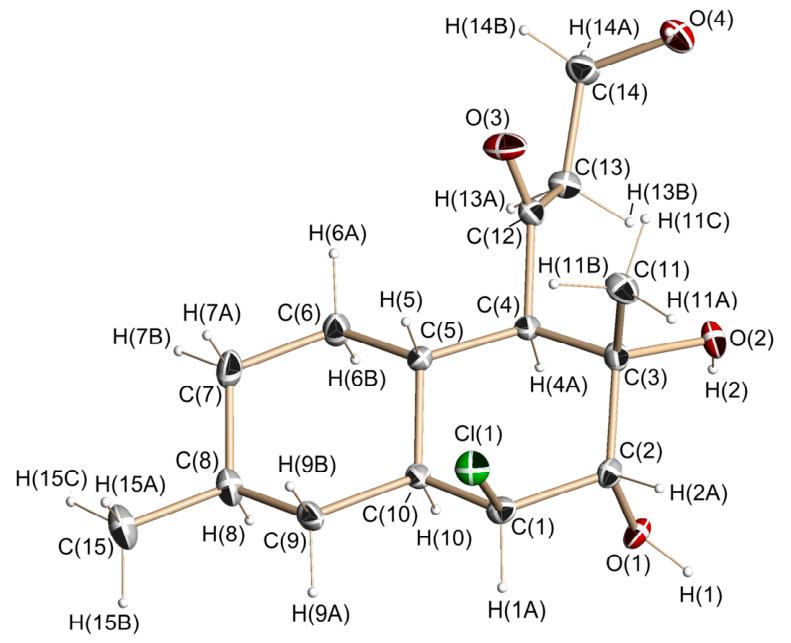
Single-crystal X-ray diffraction structure of **28** with displacement ellipsoids drawn at the 50% probability level.

The crystal structure of zosteropenilline S (Figure S1, compound **28**) is formed from C_15_H_25_ClO_4_ molecules united by intermolecular bonds (Figure 6) and H_2_O molecules. Along the direction (100), C_15_H_25_ClO_4_ molecules are stacked and interconnected by O–H···O hydrogen bonds from hydroxyl groups into double bands (Table S3). H-bonds of O−H···O with the help of H_2_O molecules, as well as weak hydrogen bonds with C−H···O and C−H···Cl double tapes are combined into layers parallel to the plane (001) (Table S3, Figure S138). In the direction (001), the layers are connected to each other by van der Waals interactions (Figure S139).

### 2.2. Proposed Biosynthetic Pathway for Decaline Polyketide Thomiorum Series

In our previous paper, we proposed a biosynthetic pathway for the biosynthesis of secondary metabolites isolated from *Penicillium yezoense* KMM 4679 cultivated on rice media [15]. In this study, in addition to the polyketides **18**, **19**, and **21**–**30** previously isolated from this fungus (for which a biosynthetic scheme has been proposed), and the new compounds **1**–**13**, we also isolated some zosteropenillines **14**–**17** and **20** previously isolated from the fungus *P. thomii* KMM 4674 [18], for which a biosynthetic scheme had not yet been proposed. In this regard, we expanded and supplemented the proposed biosynthetic scheme, which includes all metabolites isolated from *P. yezoense* KMM 4679.

**Figure 7:**
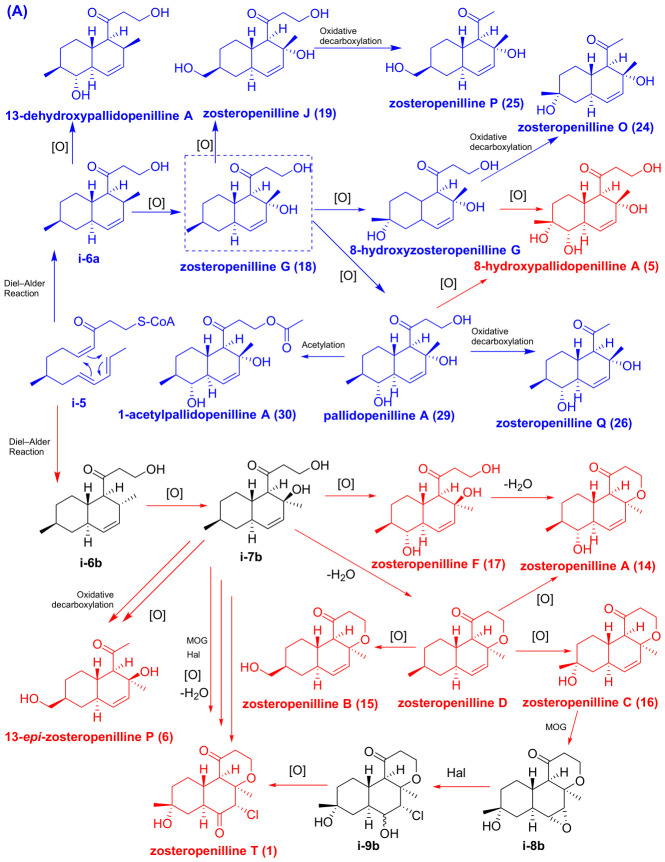

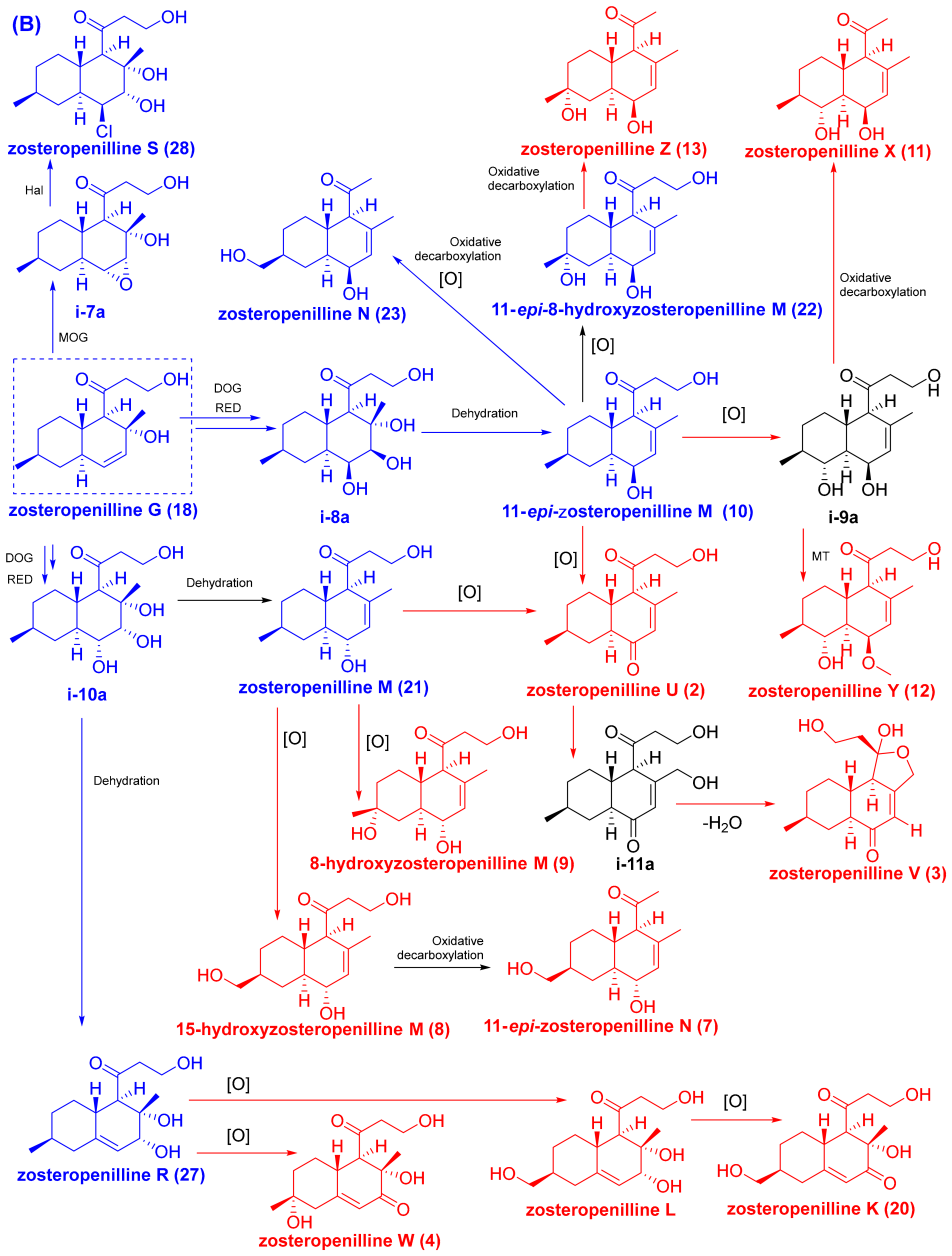
(**A**) Proposed biosynthetic pathway for compounds **5**, **6**, and **14**–**17**. Blue—previously reported part of the scheme, black—newly proposed intermediates, red—newly proposed transformations. MOG—monooxygenase, Hal—halogenation, DOG—dioxygenase, RED—reductase, MT—methyltransferase. (**B**) Proposed biosynthetic pathway for compounds **2**–**4** and **7**–**13**. Blue—previously reported part of the scheme, black—newly proposed intermediates, red—newly proposed transformations. MOG—monooxygenase, Hal—halogenation, DOG—dioxygenase, RED—reductase, MT—methyltransferase.

Previously [15], we suggested that the formation of the decalin skeleton of zosteropenillines occurs from the intermediate **i-5** through intramolecular Diels–Alder condensation, similar to lovastatin [37]. In this case, decalin derivatives with both Me-14*β* and Me-14*α* can be formed (Figure 7A). The key element in the biogenesis of Me-14*β*-derivatives is likely zosteropenilline G (**18**), as described earlier [15]. The formation of rarer Me-14*α* derivatives obviously occurs sequentially through intermediates **i-6b** and **i-7b**. In this case, the *β*-configuration of 13-OH allows cyclization of the side chain, leading to the formation of a tetrahydropyranone cycle in zosteropenilline T (**1**), 13-*epi*-zosteropenilline P (**6**), zosteropenillines A–C (**14**–**16**), and F (**17**) isolated in this study, as well as zosteropenillines D and E previously obtained from another strain [18]. Interestingly, zosteropenilline F (**17**) is the only isolated variant of the Me-14α derivative with a free side chain. In addition, despite the fact that zosteropenilline T (**1**) is most likely formed from zosteropenilline C (**16**) via the epoxy derivative **i-8b**, the possibility of oxidation of the double bond of the intermediate **i-7b** and the formation of the tetrahydropyranone cycle at the last stage of the biosynthesis of **1** cannot be excluded. The formation of 8-hydrohypallidopenilline A (**5**) by the hydroxylation of pallidopenilline A (**29**) or 8-hydroxyzosteropenilline G should also be considered equally probable.

The isolation of compound **10**, which was previously suggested as a hypothetical intermediate in the biosynthesis of 11 *β*-OH-derivatives with Δ^12^, provides good evidence for the proposed biosynthetic scheme (Figure 7B). New zosteropenillines X–Z (**11**–**13**) are hydroxy derivatives of **10**, while compounds **11** and **13** with shortened side chains at C-4 are products of the oxidative decarboxylation of proposed intermediate **i-9a** and 11-*epi*-8-hydroxyzosteropenilline M (**22**). The 11-OH group in compounds **10** and **21** might be oxidized to a ketone, resulting in zosteropenilline U (**2**), which is converted to zosteropenilline V (**3**) through an intermediate **i-11a** by cyclization via nucleophilic addition of 14-OH to C-**3** to form a tetrahydrofuran ring. 15-Hydroxyzosteropenilline M (**8**) and 8-hydroxyzosteropenilline M (**9**) are hydroxy derivatives of zosteropenilline M (**21**), and 11-*epi*-zosteropenilline M (**7**) is a decarboxylated derivative of **8**. Hydroxylation at C-15 of zosteropenilline R (**27**) results in zosteropenilline L. Oxidation of 12-OH in **27** and zosteropenilline L transforms them into zosteropenillines K (**20**) and W (**4**), respectively.

### 2.3. Bioactivity of Isolated Compounds

The antimicrobial and cytotoxic activities of compounds **2**–**10**, **12**, and **13** were evaluated. The activities of compounds **1** and **11** could not be studied because of insufficient amounts available after structure determination.

The effects of the compounds on the growth and biofilm formation of *Staphylococcus aureus*, *Escherichia coli*, and *Candida albicans* test strains were studied.

**Figure 8:**
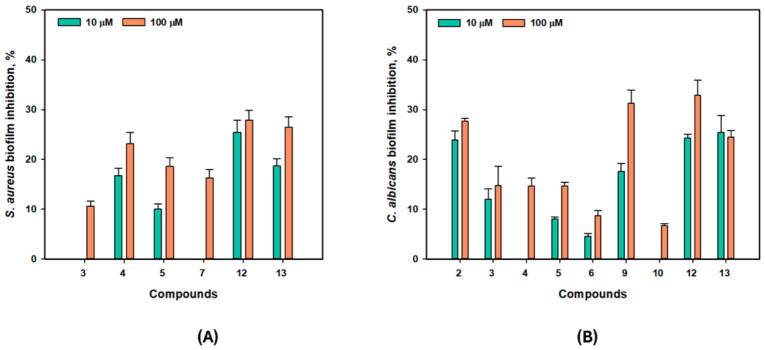
Influence of the compounds on *Staphylococcus aureus* (**A**) and *Candida albicans* (**B**) biofilm formation.

All compounds were unactive against *E. coli*. All compounds at 100 µM showed weak inhibition of the growth of *S. aureus* by 7.5–19.6%. The most effective compounds were **8** and **13**, which inhibited the growth of *S. aureus* by 28.6% and 21.0%, respectively. The *S. aureus* biofilm formation was affected by compounds **3**–**5**, **7**, **12**, and **13** (Figure 8A). The positive control gentamicin caused the total inhibition of bacterial growth and biofilm formation at 1 μg/mL.

Moreover, the compounds weakly affected the growth of *C. albicans* by 5–8%, but compounds **2**–**6**, **9**, **10**, **12**, and **13** more significantly inhibited the *C. albicans* biofilm formation (Figure 8B). Compound **2** did not inhibit the growth of *C. albicans*, but this compound at 100 µM prevented the formation of *C. albicans* biofilms by 27.7% and by 23.9% at 10 µM. Compound **9** also did not inhibit the growth of *C. albicans* but inhibited the formation of *C. albicans* biofilms by 31.3% and 17.6% at 100 µM and 10 µM, respectively. Both compounds **12** and **13** at 100 µM shown a weak inhibitory effect on *C. albicans* growth and more significantly inhibited its biofilm formation. At 10 µM, **12** and **13** inhibited *C. albicans* growth by 3–4% and *C. albicans* biofilm formation by 24.3% and 25.4%, respectively. The positive control amphotericin B caused total inhibition of *C. albicans* growth and biofilm formation at 1.25 μg/mL.

This is the first report on the effects of zosteropenillines on *C. albicans* biofilm formation. Earlier, 7α-hydroxygeosmin significantly inhibited biofilm formation and destroyed a preformed biofilm of fungi via downregulation of the expression of the adhesion-related genes HWP1, ALS1, ALS3, ECE1, EAP1, and BCR1 [38]. Campafungin A has shown antiproliferative and antihyphal activity against *C. albicans*, and its action may involve inhibition of a cAMP-dependent PKA pathway [39]. Moreover, it is impossible to exclude the effect of zosteropenillines on the activity of enzymes close to hydroxymethylglutaryl-coenzyme A (HMG-CoA) reductase, which is the target of other decalin derivatives (statins). However, this requires further investigation.

The large structural diversity and insufficient knowledge of all representatives of the zosteropenilline series of compounds complicate the analysis of structural features affecting their anti-biofilm activity. Compound **12**, which was more active in this assay, has a methoxy group at C-11. However, compound **13** does not have the methoxy group or the the oxo-propyl substituent, and with the oxygen function a different position compared to compound **12**, it was also active. The formation of a tetrahydrofuran ring C in compound **3** resulted in a decrease in activity against *C. albicans* biofilms and increased activity against *S. aureus* biofilm formation compared with **2**. It may be noted that compounds **6**–**8**, which have a hydroxymethyl group at C-8, were less active in this assay.

**Figure 9:**
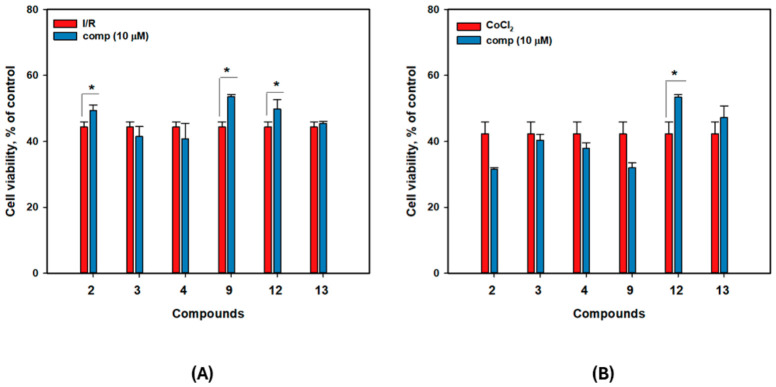
Cardioprotective activity of some isolated compounds. (**A**) The influence of the compounds on the viability of I/R-treated H9c2 cells. (**B**) The influence of the compounds on the viability of CoCl_2_-treated H9c2 cells. The data are presented as the mean ± SEM. The viability of untreated cells was 100.0 ± 2.5%. Asterisks indicate the significance of differences with *p* < 0.05 in one-way ANOVA.

The cytotoxicity of the compounds to normal H9c2 cardiomyocytes was investigated. The IC_50_s were calculated only for **3**, **4**, and **7** as 84.0 ± 0.4 µM, 98.3 ± 8.1 µM, and 73.0 ± 5.5 µM, respectively. The other compounds were nontoxic up to 100 µM. The known fungal toxin gliotoxin at 1 μM decreased the viability of H9c2 cells by 95.6%. The cardioprotective effects of the compounds were studied in ischemia/reperfusion (I/R) and CoCl_2_-mimicking chronic hypoxia in vitro models. Zosteropenilline U (**2**), 8-hydroxyzosteropenilline M (**9**), and zosteropenilline Y (**12**) at 10 µM increased the viability of I/R-treated H9c2 cells by 11.3%, 20.7%, and 12.1%, respectively (Figure 9). However, only compound **12** increased the viability of CoCl_2_-treated cells by 26.3%.

It is worth noting that all the compounds with cardioprotective properties had the 3-hydroxy-1-oxopropyl substituent at C-4 and ∆^12^ double bond in their structure, which seems to be related to their structure–activity relationship. Earlier, zosteropenillines M, O, R, and S increased the viability of CoCl_2_-treated HEK293 cells [15]. Zosteropenillines B, H, and J decreased NO levels in LPS-stimulated RAW264.7 cells [18]. However, the pathways included in the cytoprotective effects of these compounds are unknown and will be studied in the future.

Thus, a detailed discussion of the structure–activity relationship in the series of decalin derivatives of the zosteropenilline series requires the accumulation of a large amount of data in further research.

## 3. Materials and Methods

### 3.1. General Experimental Procedures

Optical rotations were measured on a Perkin-Elmer 343 polarimeter (Perkin Elmer, Waltham, MA, USA) in MeOH. UV spectra were recorded using a Shimadzu UV-1601PC spectrometer (Shimadzu Corporation, Kyoto, Japan) in MeOH. ECD spectra were measured using a Chirascan-Plus CD Spectrometer (Applied Photophysics, Leatherhead, UK) in MeOH. ^1^H and ^13^C NMR spectra were recorded in CDCl_3_ on a Bruker Avance III-700 spectrometer (Bruker BioSpin GmbH, Rheinstetten, Germany) operating at 700 and 176 MHz, respectively. HRESIMS spectra were obtained using a Bruker maXis Impact II mass spectrometer (Bruker Daltonics GmbH, Rheinstetten, Germany).

Plates precoated with Si gel (5–17 μm, 4.5 × 6.0 cm, Imid Ltd., Moscow, Russia) and Si gel 60 RP-18 F254S (20 × 20 cm, Merck KGaA, Darmstadt, Germany) were used for thin-layer chromatography (TLC). Preparative HPLC was carried out on a SepaBean machine (Santai Technologies, Changzhou, China) with a UV detector combined with an SepaFlash FP LT- ELSD detector (Santai Technologies, Changzhou, China) using a Buchi glass column (49 × 230 mm) (BUCHI Labortechnik AG, Flawil, Switzerland) passed over a Si gel KSK (50/100 μm, Imid Ltd., Russia), and on a Shimadzu LC-20 (Shimadzu, Kyoto, Japan) and Agilent 1100 (Agilent Technologies, Santa Clara, CA, USA) chromatographs using Shimadzu RID-20A (Shimadzu, Kyoto, Japan) and Agilent 1100 RID (Agilent Technologies, Santa Clara, CA, USA) detectors and YMC ODS-AM (5 µm, 250 × 10 mm; YMC Co, Kyoto, Japan), Hydro-RP (4 µm, 250 × 10 mm; Phenomenex, Torrance, CA, USA), YMC Chiral NEA (R)-NP (5 µm, 250 × 4.6 mm; YMC Co, Kyoto, Japan), HyperClone ODS (5 µm, 250 × 4.6 mm; Phenomenex, Torrance, CA, USA), ChromeCore Polar C18 (5 µm, 250 × 10 mm; NanoChrome Technologies, Suzhou, China) and Luna C18(2) (5 µm, 150 × 4.6 mm; Phenomenex, Torrance, CA, USA) columns.

### 3.2. Fungal Strain, Phylogenetic Analysis, and Molecular Identification of the Fungal Strain

The isolation and identification of the fungal strain *Penicillium yezoense* KMM 4679 has been described previously [15].

### 3.3. Cultivation of P. yezoense KMM 4679

The fungal strain was cultured for 21 days at 22 °C in 100 Erlenmeyer flasks (500 mL) on rice medium (RM) and 100 μM MgCl_2_·6H_2_O (Lenreactiv Ltd., Saint Petersburg, Russia). The RM contained 20.0 g of white round-grain polished rice *Oryza sativa* grade extra “Japonka” (Primorsky region, Russia), yeast extract powder (0.02 g, Himedia RM027, HiMedia Laboratories LLC., Maharashtra, India), KH_2_PO_4_ (0.01 g, Lenreactiv Ltd., Russia), sodium potassium tartrate (0.01 g, Reakhim Ltd., Moscow, Russia), and natural seawater (40 mL, Vodolaznaya bay, Troitsa bay, the Sea of Japan, September 2022) [40]. The natural salinity of the water used was 37.8 g/L.

### 3.4. Extraction and Isolation

At the end of the incubation period, the mycelia and medium were extracted with EtOAc (5 L). The obtained extract was concentrated to dryness. The residue was dissolved in H_2_O–EtOH (4:1) (300 mL) and was extracted with n-hexane (0.2 L × 3) and EtOAc (0.2 L × 3). After evaporation of the EtOAc layer, the residual material (8.74 g) was purified via low-pressure liquid column chromatography on a Buchi B-688 Chromatography Pump using a Buchi glass column (49 × 230 mm) passed over a Si gel column, which was eluted, followed by a step gradient from 100% n-hexane to n-hexane–EtOAc (0:100) (total volume 20 L). Fractions (250 mL) were collected, dried on a rotary evaporator, weighed, and combined based on the TLC analysis (Si gel, toluene-isopropanol, 6:1 and 3:1, *v*/*v*). As a result, we obtained purified fractions: Zp-107-Mg-A (484.6 mg), Zp-107-Mg-B (325.9 mg), Zp-107-Mg-C (2.23 g), and Zp-107-Mg-D (267 mg).

The n-hexane–EtOAc (80:20) eluate (fraction Zp-107-Mg-A, 484.6 mg) was purified on a YMC-ODS-AM column eluted with MeCN–H_2_O (70:30) to yield three subfractions: Zp-107-Mg-A-1 (174.9 mg), Zp-107-Mg-A-2 (41.9 mg), and Zp-107-Mg-A-3 (100.5 mg). Subfraction Zp-107-Mg-A-1 (174.9 mg) was purified on a Hydro-RP column eluted with CH_3_CN–H_2_O (35:65) to yield subfractions Zp-107-Mg-A-1-1 (6 mg), Zp-107-Mg-A-1-3 (6.7 mg), Zp-107-Mg-A-1-5 (2.5 mg), Zp-107-Mg-A-1-6 (17.2 mg), and individual compounds **21** (18.6 mg), **25** (17.9 mg), and **26** (8.3 mg). Subfraction Zp-107-Mg-A-1-1 (6 mg) was purified on a Hydro-RP column eluted with MeOH–H_2_O (55:45) to yield **13** (3.1 mg). Subfraction Zp-107-Mg-A-1-3 (6.7 mg) was purified on a Hydro-RP column eluted with CH_3_OH–H_2_O (60:40) to yield **6** (0.9 mg) and **24** (4.1 mg). Zp-107-Mg-A-1-5 (2.5 mg) was purified on a Hydro-RP column eluted with CH_3_OH–H_2_O (65:35) to yield **11** (1.7 mg). Subfraction Zp-107-Mg-A-1-6 (17.2 mg) was purified on a Hydro-RP column eluted with CH_3_OH–H_2_O (65:35) to yield **3** (1.2 mg) and **15** (5.1 mg). Subfraction Zp-107-Mg-A-2 (41.9 mg) was purified on a YMC Chiral NEA (R)-NP column eluted with CH_3_CN–H_2_O (30:70) to yield **2** (5.0 mg) and **10** (22.6 mg). Subfraction Zp-107-Mg-A-3 (100.5 mg) was purified on a ChromeCore Polar C18 column eluted with CH_3_CN–H_2_O (55:45) to yield **18** (79.6 mg).

The n-hexane–EtOAc (60:40) eluate (fraction Zp-107-Mg-B, 325.9 mg) was purified on a YMC-ODS-AM column eluted with CH_3_CN–H_2_O (70:30) to yield three subfractions: Zp-107-Mg-B-1 (199 mg), Zp-107-Mg-B-2 (70.8 mg), and Zp-107-Mg-B-3 (30.2 mg). Subfraction Zp-107-Mg-B-1 (199 mg) was purified on a Hydro-RP column eluted with CH_3_CN–H_2_O (30:70) to yield subfractions Zp-107-Mg-B-1-1 (12 mg) and **30** (5.7 mg). Subfraction Zp-107-Mg-B-1-1 (12 mg) was purified on a Hydro-RP column eluted with CH_3_OH–H_2_O (55:45) to yield **17** (8.4 mg). Subfraction Zp-107-Mg-B-2 (70.8 mg) was purified on a Chrome-Core Polar C18 column eluted with CH_3_CN–H_2_O (45:55) to yield **28** (26.5 mg).

The n-hexane-EtOAc (40:60) eluate (fraction Zp-107-Mg-C, 2.23 g) was purified on a YMC-ODS-AM column eluted with CH_3_CN–H_2_O (60:40) to yield subfraction Zp-107-Mg-C-1 (832 mg). Subfraction Zp-107-Mg-C-1 (832 mg) was purified on a YMC-ODS-AM column eluted with CH_3_CN–H_2_O (60:40) to yield Zp-107-Mg-C-1-1 (637 mg). Subfraction Zp-107-Mg-C-1-1 (637 mg) was purified on a Hydro-RP column eluted with CH_3_OH–H_2_O (50:50) to yield subfractions Zp-107-Mg-C-1-1-2 (25.6 mg), Zp-107-Mg-C-1-1-3 (13.5 mg), Zp-107-Mg-C-1-1-4 (8.4 mg), Zp-107-Mg-C-1-1-5 (28.0 mg), Zp-107-Mg-C-1-1-7 (11 mg), Zp-107-Mg-C-1-1-8 (15.9 mg), Zp-107-Mg-C-1-1-9 (42.2 mg), Zp-107-Mg-C-1-1-10 (7.4 mg), and Zp-107-Mg-C-1-1-11 (3.0 mg). Subfraction Zp-107-Mg-C-1-1-2 (25.6 mg) was purified on a HyperClone column eluted with CH3OH–H2O (50:50) to yield **8** (4.2 mg), **22** (9.3 mg), and **20** (1.5 mg). Subfraction Zp-107-Mg-C-1-1-3 (13.5 mg) was purified on a HyperClone column eluted with CH_3_OH–H_2_O (25:75) to yield **1** (0.7 mg) and **9** (7.7 mg). Subfraction Zp-107-Mg-C-1-1-4 (8.4 mg) was purified on a HyperClone column eluted with CH_3_OH–H_2_O (20:80) to yield **4** (1.3 mg). Subfraction Zp-107-Mg-C-1-1-5 (28.0 mg) was purified on a YMC Chiral NEA (R)-NP column eluted with CH_3_OH–H_2_O (20:80) to yield **19** (11 mg). Subfraction Zp-107-Mg-C-1-1-7 (28.0 mg) was purified on a YMC Chiral NEA (R)-NP column eluted with CH_3_OH–H_2_O (40:60) to yield **14** (5.5 mg). Subfraction Zp-107-Mg-C-1-1-8 (20.8 mg) was purified on a YMC Chiral NEA (R)-NP column eluted with CH_3_OH–H_2_O (30:70) to yield **7** (1.3 mg) and **29** (1.6 mg). Subfraction Zp-107-Mg-C-1-1-9 (42.2 mg) was purified on a YMC Chiral NEA (R)-NP column eluted with CH_3_OH–H_2_O (35:65) to yield **23** (4.3 mg) and **27** (1.6 mg). Subfraction Zp-107-Mg-C-1-1-10 (7.4 mg) was purified on a HyperClone column eluted with CH_3_OH–H_2_O (30:70) to yield **12** (2.9 mg). Subfraction Zp-107-Mg-C-1-1-11 (3.0 mg) was purified on a HyperClone column eluted with CH_3_OH–H_2_O (40:60) to yield **16** (1.3 mg). Finally, compound **29** (80 mg) was obtained by recrystallization of the solid residue of fraction Zp-107-Mg-C-1-1 (637 mg).

The n-hexane–EtOAc (40:60) eluate (Zp-107-Mg-D, 267 mg) was purified on a YMC-ODS-AM column eluted with CH_3_CN–H_2_O (70:30) to yield subfraction Zp-107-Mg-D (209.4 mg). The subfraction was purified on a Luna C18(2) column eluted with CH_3_CN–H_2_O (20:80) to yield subfraction Zp-107-Mg-D-2 (20 mg). Subfraction Zp-107-Mg-D-2 (20 mg) was purified on a ChromeCore Polar C18 column eluted with CH_3_CN–H_2_O (15:85) to yield **5** (1.8 mg).

### 3.5. Spectral Data

Zosteropenilline T (**1**): colorless amorphous solid; [α]_D_^20^ −119.1 (*c* 0.02, MeOH); UV (0.7 mM, MeOH) λ_max_ (log ε) 196 (3.75), 227 (3.29), 246 (3.35) nm (Figure S10); CD (0.7 mM, MeOH), λ_max_ (∆ε) 209 (−2.29), 257 (−0.94), 293 (+0.96) nm (Figure S11); ^1^H and ^13^C NMR data, see Table 1 and Table 2, Supplementary Figures S4–S9; HRESIMS [M + Na]^+^ *m/z* 323.1028 (calcd. for C_15_H_21_ClO_4_Na 323.1021, ∆ −2.2 ppm) (Figure S3).

Zosteropenilline U (**2**): colorless amorphous solid; [α]_D_^20^ +83.3 (*c* 0.04, MeOH); UV (0.7 mM, MeOH) λ_max_ (log ε) 198 (3.93), 203 (3.93), 233 (4.27) nm (Figure S21); CD (0.7 mM, MeOH), λ_max_ (∆ε) 200 (−29.8), 236 (+1.94), 249 (−1.12), 294 (+8.05) nm (Figure S22); ^1^H and ^13^C NMR data, see Table 1 and Table 2, Supplementary Figures S14–S20; HRESIMS [M + Na]+ m/z 273.1471 (calcd. for C_15_H_22_O_3_Na 273.1461, ∆ −3.7 ppm); [M − H]^−^ *m/z* 249.1495 (calcd. for C_15_H_21_O_3_ 249.1496, ∆ +0.4 ppm) (Figure S13).

Zosteropenilline V (**3**): colorless amorphous solid; [α]_D_^20^ +28.6 (*c* 0.11, MeOH); UV (0.6 mM, MeOH) λ_max_ (log ε) 197 (3.84), 211 (3.72), 238 (4.05) nm (Figure S32); CD (0.6 mM, MeOH), λ_max_ (∆ε) 201 (−16.86), 239 (+10.6), 329 (−1.60) nm (Figure S33); ^1^H and ^13^C NMR data, see Table 1 and Table 2, Supplementary Figures S25–S31; HRESIMS [M + Na]^+^ *m/z* 289.1418 (calcd. for C_15_H_22_O_4_Na 289.1410, ∆ −2.8 ppm); [M − H]^−^ *m/z* 265.1447 (calcd. for C_15_H_21_O_4_ 265.1445, ∆ −0.8 ppm); [M + H]^+^ *m/z* 267.1590 (calcd. for C_15_H_22_O_4_ 265.1591, ∆ −0.4 ppm) (Figure S24).

Zosteropenilline W (**4**): colorless amorphous solid; [α]_D_^20^ +20.4 (*c* 0.14, MeOH); UV (3.1 mM, MeOH) λ_max_ (log ε) 198 (3.55), 207 (3.51), 245 (3.75) nm (Figure S43); CD (3.1 mM, MeOH), λ_max_ (∆ε) 195 (+0.78), 219 (−9.28), 242 (+3.01), 287 (+1.45) nm (Figure S44); ^1^H and ^13^C NMR data, see Table 1 and Table 2, Supplementary Figures S36–S41; HRESIMS [M + Na]^+^ *m/z* 305.1367 (calcd. for C_15_H_22_O_5_Na 305.1359, ∆ −2.6 ppm); [M − H]^−^ *m/z* 281.1393 (calcd. for C_15_H_22_O_5_ 281.1394, ∆ +0.4 ppm); [M + H]^+^ *m/z* 283.1541 (calcd. for C_15_H_22_O_5_ 283.1540, ∆ −0.4 ppm) (Figure S35).

8-Hydroxypallidopenilline A (**5**): colorless amorphous solid; [α]_D_^20^ −62.5 (*c* 0.09, MeOH); UV (1.6 mM, MeOH) λ_max_ (log ε) 197 (3.84), 259 (2.49), 278 (2.25) nm (Figure S54); CD (1.6 mM, MeOH), λ_max_ (∆ε) 197 (−2.78), 212 (+0.63), 294 (−1.35) nm (Figure S55); ^1^H and ^13^C NMR data, see Table 1 and Table 2, Supplementary Figures S47–S53; HRESIMS [M + Na]^+^ *m/z* 307.1521 (calcd. for C_15_H_24_O_5_Na 305.1359, ∆ −1.6 ppm); [M − H]^−^ *m/z* 283.1546 (calcd. for C_15_H_23_O_5_ 283.1551, ∆ +1.8 ppm); [M + Cl]^−^ *m/z* 319.1313 (calcd. for C_15_H_24_O_5_Cl 319.1318, ∆ +1.6 ppm) (Figure S46).

13-*Epi*-zosteropenilline P (**6**): colorless amorphous solid; [α]_D_^20^ +48.1 (*c* 0.16, MeOH); UV (1.9 mM, MeOH) λ_max_ (log ε) 196 (3.77) nm (Figure S65); CD (1.9 mM, MeOH), λ_max_ (∆ε) 196 (+0.20), 204 (−1.59), 292 (+0.43) nm (Figure S66); ^1^H and ^13^C NMR data, see Table 5 and Table 6, Supplementary Figures S58–S64; HRESIMS [M + Na]^+^ *m/z* 267.1469 (calcd. for C_14_H_22_O_3_Na 261.1461, ∆ −3.1 ppm); [M − H]^−^ *m/z* 237.1493 (calcd. for C_14_H_21_O_3_ 237.1496, ∆ +1.3 ppm) (Figure S57).

11-*Epi*-zosteropenilline N (**7**): colorless amorphous solid; [α]_D_^20^ +57.7 (*c* 0.16, MeOH); ^1^H and ^13^C NMR data, see Table 3 and Table 4, Supplementary Figures S69–S75; HRESIMS [M + Na]^+^ *m/z* 261.1467 (calcd. for C_14_H_22_O_3_Na 261.1461, ∆ −2.6 ppm) (Figure S68).

15-Hydroxyzosteropenilline M (**8**): colorless amorphous solid; [α]_D_^20^ +23.5 (*c* 0.19, MeOH); ^1^H and ^13^C NMR data, see Table 3 and Table 4, Supplementary Figures S78–S84; HRESIMS [M + Na]^+^ *m/z* 291.1572 (calcd. for C_15_H_24_O_4_Na 291.1567, ∆ −1.7 ppm); [M − H]^−^ *m/z* 267.1596 (calcd. for C_15_H_23_O_4_ 267.1602, ∆ +2.2 ppm); [M + Cl]^−^ *m/z* 303.1365 (calcd. for C_15_H_24_O_4_Cl 303.1369, ∆ +1.3 ppm) (Figure S77).

8-Hydroxyzosteropenilline M (**9**): colorless amorphous solid; [α]_D_^20^ +62.2 (*c* 0.12, MeOH); UV (0.5 mM, MeOH) λ_max_ (log ε) 197 (4.07), 265 (2.68), 277 (2.70) nm (Figure S94); CD (0.5 mM, MeOH), λ_max_ (∆ε) 195 (+2.75), 220 (+0.76), 294 (+2.63) nm (Figure S95); ^1^H and ^13^C NMR data, see Table 3 and Table 4, Supplementary Figures S87–S93; HRESIMS [M + Na]^+^ *m/z* 291.1575 (calcd. for C_15_H_24_O_4_Na 291.1567, ∆ −2.7 ppm); [M − H]^−^ *m/z* 267.1600 (calcd. for C_15_H_23_O_4_ 267.1602, ∆ +0.7 ppm); [M + Cl]^−^ *m/z* 303.1366 (calcd. for C_15_H_24_O_4_Cl 303.1369, ∆ +1.0 ppm) (Figure S86).

11-*Epi*-zosteropenilline M (**10**): colorless amorphous solid; [α]_D_^20^ +6.12 (*c* 0.05, MeOH); UV (1.6 mM, MeOH) λ_max_ (log ε) 197 (3.95), 267 (2.48), 276 (2.48) nm (Figure S105); CD (1.6 mM, MeOH), λ_max_ (∆ε) 197 (−5.44), 212 (+1.87), 293 (+3.09) nm (Figure S106); ^1^H and ^13^C NMR data, see Table 3 and Table 4, Supplementary Figures S98–S104; HRESIMS [M + Na]^+^ *m/z* 275.1627 (calcd. for C_15_H_24_O_3_Na 275.1618, ∆ −3.3 ppm); [M − H]^−^ *m/z* 251.1651 (calcd. for C_15_H_24_O_3_ 251.1653, ∆ +0.8 ppm) (Figure S97).

Zosteropenilline X (**11**): colorless amorphous solid; [α]_D_^20^ −17.3 (*c* 0.11, MeOH); ^1^H and ^13^C NMR data, see Table 3 and Table 4, Supplementary Figures S109–S115; HRESIMS [M + Na]^+^ *m/z* 261.1467 (calcd. for C_14_H_22_O_3_Na 261.1461, ∆ −2.3 ppm); [M − H]^−^ *m/z* 237.1497 (calcd. for C_14_H_21_O_3_ 237.1496, ∆ −0.4 ppm) (Figure S108).

Zosteropenilline Y (**12**): colorless amorphous solid; [α]_D_^20^ −47.5 (*c* 0.12, MeOH); UV (c 1 mM, MeOH) λ_max_ (log ε) 197 (3.99) nm (Figure S125); CD (0.1 mM, MeOH), λ_max_ (∆ε) 195 (−0.15), 206 (−5.75), 294 (+1.85) nm (Figure S126); ^1^H and ^13^C NMR data, see Table 5 and Table 6, Supplementary Figures S118–S124; HRESIMS [M + Na]^+^ *m/z* 305.1735 (calcd. for C_16_H_26_O_4_Na 305.1723, ∆ −3.9 ppm) (Figure S117).

Zosteropenilline Z (**13**): colorless amorphous solid; [α]_D_^20^ −6.67 (*c* 0.06, MeOH); UV (2.1 mM, MeOH) λ_max_ (log ε) 197 (3.78) nm (Figure S136); CD (2.1 mM, MeOH), λ_max_ (∆ε) 196 (−2.91), 213 (+0.59), 290 (+1.72) nm (Figure S137); ^1^H and ^13^C NMR data, see Table 5 and Table 6, Supplementary Figures S129–S135; HRESIMS [M + Na]^+^ *m/z* 261.1469 (calcd. for C_14_H_22_O_3_Na 261.1461, ∆ −3.1 ppm); [M − H]^−^ *m/z* 237.1492 (calcd. for C_14_H_21_O_3_ 237.1496, ∆ +1.7 ppm) (Figure S128).

### 3.6. X-Ray Crystallographic Data of 28

Experimental intensity data for C_15_H_25_ClO_4_·H_2_O (**28**) were collected at T = 120(2)K on a BRUKER Kappa APEX2 diffractometer with graphite monochromated MoKα radiation (λ = 0.71073 Å). The intensity data were corrected for absorption using the multi-scan method. The structure was solved using direct methods and refined by least-squares calculations in an anisotropic approximation for non-hydrogen atoms. Hydrogen atoms for the molecule C_15_H_25_ClO_4_ were placed in geometrically idealized positions and refined using the riding-model approximation. The position parameters of the H atoms of water molecules were refined freely with Uiso(H) = 1.5Ueq(Ow). Data collection, reduction, and refinement of the lattice parameters were performed using the Apex2 software package (Bruker. APEX 2 V.7, Bruker AXS Inc., Madison, WI, USA (2010)). All calculations were performed using the SHELX program suite [41,42]. The main crystallographic data, details of the refinement, and bond lengths and angles of the crystal structure of **28** are shown in Tables S1 and S2.

The crystallographic data (accession numbers CCDC 2546332) can be obtained free of charge from the Cambridge Crystallographic Data Center at http://www.ccdc.cam.ac.uk/data_request/cif (or from the Cambridge Crystallographic Data Centre, 12 Union Road, Cambridge, UK; fax: +44-1223-336-033 or email: deposit@ccdc.cam.uk).

C_15_H_25_ClO_4_, M = 304, Crystal size: 0.38 × 0.23 × 0.12 mm^3^, orthorhombic, space group Orthorhombic, P2_1_2_1_2_1_, a = 7.2622(6), b = 7.7796(7), c = 28.257(3), V = 1596.5(2) Å3, Z = 26, D_calc._ = 1.343 g/cm^3^, μ = 0.258 mm^−1^. F000 = 696, θ_max_ = 28.12°, 14521/ reflections collected, 3897 unique [R(_int_) = 0.0228]. Final GooF = 1.049; for I > 2 σ (I) R1 = 0.0259, wR2 = 0.0630; for all data R1 = 0.0279, wR2 = 0.0639, |Δρ|_max_ = 0.255e/Å3.

### 3.7. Quantum-Chemical Modeling

All quantum-chemical calculations were performed using the B3LYP exchange-correlation functional, the polarization continuum model (PCM), and 6-311G(d) basis set implemented in the Gaussian 16 program package [43]. The statistical weights (g_im_) of the individual conformations were calculated according to the following equation:

(1) $$g_{im}=e^{\frac{-\Delta G_{im}}{RT}}∕\sum_{i}e^{\frac{-\Delta G_{im}}{RT}}$$

where ΔG_im_ = G_i_–G_m_ is the relative Gibbs free energy and the index “m” denotes the most stable conformation.

The ECD spectra were calculated using the time-dependent density functional theory (TDDFT), B3LYP functional, PCM model, and 6-311G(d) basis set for conformations, where the relative Gibbs free energies satisfied the relation ΔG_im_ ≤ 4 kcal/mol. To describe the short-wave region of the ECD spectra, 95 electronic transitions were calculated for each conformation of **1**. The Gauss-type functions were used to simulate the individual bands in the theoretical spectra. The bandwidths ζ = 0.16 eV and the UV shifts Δλ = +1 nm were used for the best correspondence between the experimental and calculated spectra for **1**.

The scaled theoretical and experimental ECD spectra were obtained according to the equation:

(2) $$\Delta \varepsilon_{sc}\left(\lambda \right)=\Delta \varepsilon \left(\lambda \right)∕\left|\Delta \varepsilon \left(\lambda_{peak}\right)\right|$$

where the denominator |Δε (λ_peak_)| is the modulo of the peak value for the positive characteristic band at λ ≈ 216 nm in the corresponding ECD spectrum.

### 3.8. Bioassays

#### 3.8.1. Antimicrobial Assay

The Gram-positive bacterium *Staphylococcus aureus* ATCC 21027, Gram-negative bacterium *Escherichia coli* VKPM (B-7935) and yeast-like fungus *Candida albicans* KMM 455 strains were fermented on solid medium Mueller Hinton broth with agar (16.0 g/L) in a Petri dish at 37 °C for 24 h.

The antimicrobial activity of the compound was tested at concentrations ranging from 100 µM and lower. The effect of the compound on bacterial growth was estimated as described by [44]. Gentamicin and amphotericin B were used as positive controls against *S. aureus*, *E. coli* and *C. albicans*, and a 1% dimethyl sulfoxide (DMSO) solution in PBS was used as a negative control. The optical density of the bacterial suspension after 18 h was measured at λ = 620 nm. The effect of the compound on biofilm formation for 18 h was tested using the MTT reagent (Sigma-Aldrich, St. Louis, MO, USA) in accordance with [45]. The optical density of the obtained solution was measured at λ = 570 nm. A Multiskan FS spectrophotometer (Thermo Scientific Inc., Beverly, MA, USA) was used for both assays. The results were calculated as percentages of the control data.

#### 3.8.2. Cell Culture

Rat cardiomyocyte H9c2 cells were kindly provided by Prof. Dr. Gunhild von Ams-berg from the Martini-Klinik Prostate Cancer Center, University Hospital Hamburg-Eppendorf, Hamburg, Germany. H9c2 cells were cultured in DMEM with 10% fetal bovine serum and 1% penicillin/streptomycin (BioloT, St. Petersburg, Russia) at 37 °C in a humidified atmosphere with 5% (*v*/*v*) CO_2_ and seeded for experiments at a concentration of 3 × 10^3^ cells/well.

#### 3.8.3. Cell Viability Assay

All compounds were dissolved in DMSO such that the final concentration of DMSO in the cell culture did not exceed 1%. DMSO was used as a control. Compounds at concentrations up to 100 µM were added to the wells for 48 h, and the viability of the cells was measured using an MTT (3-(4,5-dimethylthiazol-2-yl)-2,5-diphenyltetrazolium bromide) assay, which was performed according to the manufacturer’s instructions (Sigma-Aldrich, Munich, Germany). The absorbance of the converted formazan was measured using a Multiskan FC microplate photometer (Thermo Scientific, Waltham, MA, USA) at λ = 570 nm. The known fungal toxin gliotoxin was used as a control. The results are presented as percentages of control data, and IC_50_s were calculated if possible.

#### 3.8.4. In Vitro Ischemia/Reperfusion (I/R) and CoCl_2_-Mimicking Hypoxia Modeling

H9c2 cells were seeded in 96-well plates (3 × 10^3^ cells per well) and incubated overnight.

For ischemia/reperfusion (I/R) modeling, the full culture medium was removed and poor medium (30% DMEM, 70% PBS) with 500 µM of CoCl_2_ was added for 5 h. The poor medium was then replaced with full culture medium. After 30 min, the compounds were added at a concentration of 10 µM. The viability of H9c2 cells was measured after 24 h using the MTT assay, as described above.

For CoCl_2_-mimicking hypoxia modeling, the H9c2 cells were treated with a dH_2_O-solution of CoCl_2_ at a concentration of 500 µM for 1 h. The compounds were then added at a concentration of 10 µM for 47 h. The viability of H9c2 cells was measured using the MTT assay, as described above.

#### 3.8.5. Statistical Data Evaluation

All data were obtained twice in three independent replicates, and the calculated values are expressed as the mean ± standard error of the mean (SEM). One-way ANOVA was performed using SigmaPlot 14.0 (Systat Software Inc., San Jose, CA, USA) to determine statistical significance. The differences were considered statistically significant at *p* < 0.05.

## 4. Conclusions

Thirteen new decaline polyketides, zosteropenillines T–W (**1**–**4**), 8-hydroxypallidopenilline A (**5**), 13-*epi*-zosteropenilline P (**6**), 11-*epi*-zosteropenilline N (**7**), 15-hydroxyzosteropenilline M (**8**), 8-hydroxyzosteropenilline M (**9**), 11-*epi*-zosteropenilline M (**10**), and zosteropenillines X–Z (**11**–**13**), together with 17 known related compounds (**14**–**30**) were isolated from the ethyl acetate extract of the marine-derived fungus *Penicillium yezoense* KMM 4679 cultivated on MgCl_2_-containing rice media. The absolute configurations of zosteropenillines T (**1**) and V (**3**) were determined using time-dependent density functional theory (TD-DFT) calculations of the ECD spectra. Single-crystal X-ray diffraction analysis was successfully carried out for the known metabolite zosteropenilline S (**28**), yielding high-resolution crystallographic data that unambiguously confirmed its molecular structure, relative configuration, and conformational features. Based on the principles of biosynthesis, a proposed biogenetic pathway was developed to explain the structural diversity of decalinne polyketides produced by the fungus *Penicillium yezoense* KMM 4679. The effects of the compounds on the *Staphylococcus aureus* and *Candida albicans* growth and biofilm formation were observed. Zosteropenillines U (**2**), Y (**12**) and Z (**13**) with higher activity against *C. albicans* biofilms were nontoxic for normal cardiomyocyte H9c2 cells, which make them promising as anti-candidal agents. Moreover, zosteropenillines U and Y demonstrated cardioprotective effects in acute ischemia/reperfusion and CoCl_2_-mimicking hypoxia in vitro models.

## Figures and Tables

**Table 1:** ^13^C NMR data (δ_C_ in ppm, type) for compounds **1**–**5.**

No	1	2	3	4	5
1	61.6, CH_2_	57.4, CH_2_	59.7, CH_2_	57.9, CH_2_	58.1, CH_2_
2	37.3, CH_2_	44.9, CH_2_	40.4, CH_2_	48.9, CH_2_	49.2, CH_2_
3	207.3, C	212.0, C	106.3, C	211.3, C	213.9, C
4	58.5, CH	61.6, CH	55.4, CH	61.4, CH	63.0, CH
5	41.2, CH	41.7, CH	40.6, CH	38.6, CH	39.0, CH
6	25.9, CH_2_	31.4, CH_2_	31.1, CH_2_	28.1, CH_2_	24.1, CH_2_
7	37.7, CH_2_	33.8, CH_2_	33.6, CH_2_	37.3, CH_2_	37.5, CH_2_
8	68.9, C	31.7, CH	32.0, CH	71.3, C	71.5, C
9	37.2, CH_2_	33.7, CH_2_	34.3, CH_2_	48.0, CH_2_	77.2, CH
10	41.0, CH	48.6, CH	49.6, CH	164.6, C	43.4, CH
11	203.9, C	199.1, C	199.8, C	121.7, C	127.4, CH
12	62.8, CH	128.8, CH	120.4, CH	199.9, C	134.9, CH
13	82.4, C	154.7, C	166.1, C	74.2, C	72.4, C
14	19.7, CH_3_	21.8, CH_3_	67.3, CH_2_	21.8, CH_3_	25.8, CH_3_
15	31.6, CH_3_	22.4, CH_3_	22.6, CH_3_	30.8, CH_3_	27.4, CH_3_

Chemical shifts were measured at 176.04 MHz in CDCl_3._

**Table 2:** ^1^H NMR spectroscopic data (δ_H_ in ppm, mult., *J* in Hz) for compounds **1**–**5.**

No	1	2	3	4	5
1	4.19, dd (11.6, 9.3) 4.00, td (11.8, 4.3)	3.90, m (2H)	4.25, t (10.8, 2.0) 3.96, dt (10.8, 4.1)	3.96, ddd (11.4, 7.7, 3.6) 3.83, ddd (11.4, 6.4, 3.9)	3.91, ddd (10.9, 7.2, 3.6) 3.86, ddd (10.6, 6.6, 3.7)
2	2.73, ddd (15.4, 11.3, 9.5) 2.20, dd (15.4, 4.2)	2.75 (18.5, 6.0, 4.4) 2.71 (18.5, 6.3, 4.3)	2.36, ddd (14.7, 10.8, 4.3) 1.95, m	3.15, ddd (18.0, 6.4, 3.6) 2.67, ddd (18.0, 7.7, 3.9)	3.08, ddd (19.9, 6.8, 3.7) 2.61, ddd (18.0, 7.1, 3.8)
4	2.64, d (11.5)	3.23, d (9.9)	2.46, dq (9.9, 2.0)	3.08, d (9.7)	2.96, d (11.6)
5	1.97, qd (11.8, 3.3)	1.96, ddd (12.5, 10.0, 3.0)	1.92, m	2.86, m	1.59, m
6	1.67, m 1.34, m	1.68, m 1.27, m	2.15, dq (12.6, 3.5) 1.25, m	1.56, m 1.71, m	1.49, m 1.26, m
7	1.68, m 1.30, m	1.69, m 0.87, m	1.70, dq (13.4, 3.1) 0.93, m	1.66, m 1.73, m	1.80, m 1.45, td (14.0, 4.5)
8	-	1.42, m	1.43, m	-	-
9	1.90, dt (14.3, 3.0) 1.49, dd (14.3, 12.2)	2.26, dq (13.7, 3.2) 0.85, m	2.31, dq (13.7, 3.2) 0.83, q (11.6)	2.47, dd (14.9, 2.8) 2.34, dt (14.9, 1.5)	3.03, d (10.8)
10	3.33, td (11.7, 3.3)	1.99, td (12.5, 3.5)	1.95, m	-	2.16, tt (10.7, 2.3)
11	-	-	-	5.91, t (2.0)	5.97, dd (10.2, 1.4)
12	3.88, s	5.97, brs	5.94, q (2.0)	-	5.55, dd (10.1, 2.8)
14	1.39, s	1.84, s	4.64, (16.0, 2.1) 4.61, (16.0, 2.1)	1.19, s	1.19, s
15	1.29, s	0.94, d (6.5)	0.95, d (6.5)	1.34, s	1.27, s

Chemical shifts were measured at 700 MHz in CDCl_3._

**Table 3:** ^13^C NMR data (δ_C_ in ppm, type) for compounds **6**–**9.**

No	6	7	8	9
1	-	-	57.9, CH_2_	57.7, CH_2_
2	34.6, CH_3_	28.0, CH_3_	43.2, CH_2_	43.7, CH_2_
3	215.8, C	211.2, C	214.4, C	214.5, C
4	61.6, CH	62.8, CH	62.1, CH	61.6, CH
5	39.6, CH	39.1, CH	39.2, CH	38.5, CH
6	29.4, CH_2_	30.7, CH_2_	30.7, CH_2_	26.5, CH_2_
7	29.2, CH_2_	28.6, CH_2_	28.6, CH_2_	37.7, CH_2_
8	40.6, CH	39.4, C	39.4, C	69.0, C
9	35.1, CH_2_	32.7, CH_2_	32.6, CH_2_	42.0, CH_2_
10	42.2, CH	44.8, CH	44.8, CH	38.5, CH
11	132.9, CH	72.6, C	72.5, C	72.4, C
12	132.5, CH	129.4, CH	129.8, CH	129.9, CH
13	69.4, C	132.4, C	132.0, C	132.1, C
14	28.9, CH_3_	21.1, CH_3_	21.1, CH_3_	21.8, CH_3_
15	68.2, CH_2_	68.3, CH_2_	68.2, CH_2_	31.6, CH_3_

Chemical shifts were measured at 176.04 MHz in CDCl_3._

**Table 4:** ^1^H NMR spectroscopic data (δ_H_ in ppm, mult., *J* in Hz) for compounds **6**–**9.**

No	6	7	8	9
1	-	-	3.85, m (2H)	3.85, m (2H)
2	2.27, s	2.10, s	2.70, ddd (18.3, 11.3, 4.5) 2.64, ddd (18.0, 10.5, 5.2)	2.72, ddd (18.3, 10.6, 4.3) 2.65, ddd (18.3, 10.6, 6.1)
4	2.59, d (10.8)	2.81, d (10.0)	2.85, d (9.7)	2.92, m
5	1.79, m	1.52, m	1.58, m	1.54, m
6	1.64, m 1.19, qd (12.7, 3.5)	1.73, m 1.16, m	1.72, m 1.16, m	1.55, m 1.48, m
7	1.85, m 1.04, qd (12.7, 3.7)	1.82, m 0.97, qd (13.0, 3.5)	1.80, m 0.96, qd (12.9, 3.6)	1.64, m 1.35, m
8	1.65, m	1.54, m	1.55, m	-
9	1.89, m 0.92, q	2.34 dq (12.7, 3.5) 0.77 q (12.1)	2.33, dq (12.8, 3.0) 0.76, q (12.2)	2.21, dt (13.9, 3.2) 1.13, m
10	1.81, m	1.18, m	1.20, m	1.54, m
11	5.54, brd (10.0)	3.90, dm (9.0)	3.90, dm (9.0)	3.86, m
12	5.56, dd (9.9, 1.8)	5.59, brs	5.59, brs	5.59, brs
14	1.29, s	1.60, brs	1.60, brs	1.60, s
15	3.49, dd (10.5; 6.3) 3.47, dd (10.5, 6.3)	3.49, d (6.2), (2H)	3.49, d (6.3), (2H)	1.26, s

Chemical shifts were measured at 700 MHz in CDCl_3._

**Table 5:** ^13^C NMR data (δ_C_ in ppm, type) for compounds **10**–**13.**

No	10	11	12	13
1	57.8, CH_2_	-	58.2, CH_2_	-
2	43.0, CH_2_	28.0, CH_3_	40.9, CH_2_	28.6, CH_3_
3	214.5, C	210.9, C	213.9, C	211.3, C
4	62.8, CH	62.9, CH	63.6, CH	62.7, CH
5	33.7, CH	33.3, CH	33.6, CH	33.3, CH
6	31.8, CH_2_	31.1, CH_2_	31.5, CH_2_	26.8, CH_2_
7	34.3, CH_2_	32.2, CH_2_	32.3, CH_2_	37.8, CH_2_
8	32.3, CH	39.1, CH	39.4, CH	69.8, C
9	36.8, CH_2_	75.3, CH	75.1, CH	40.3, CH_2_
10	41.7, CH	48.2, CH	48.3, CH	37.2, CH
11	66.8, CH	62.5, CH	70.6, CH	66.7, CH
12	127.4, CH	126.5, CH	125.0, CH	127.0, CH
13	134.9, C	135.3, C	135.6, C	135.5, C
14	21.5, CH_3_	21.3, CH_3_	21.9, CH_3_	21.4, CH_3_
15	22.6, CH_3_	18.5, CH_3_	18.7, CH_3_	31.8, CH_3_
16	-	-	56.9, CH_3_	-

Chemical shifts were measured at 176.04 MHz in CDCl_3._

**Table 6:** ^1^H NMR spectroscopic data (δ_H_ in ppm, mult., *J* in Hz) for compounds **10**–**13.**

No	10	11	12	13
1	3.84, d (5.5) 3.83, d (5.4)	-	3.83, dd (10.7, 5.4) 3.82, dd (10.7, 5.4)	-
2	2.76, ddd (18.3, 10.8, 5.9) 2.65, ddd (18.3, 10.7, 5.8)	2.13, s	2.73, ddd (18.0, 10.7, 6.2) 2.58, ddd (18.0, 10.7, 6.2)	2.15, s
4	2.75, d (10.3)	2.75, d (9.6)	2.70, d (9.7)	2.81, d (9.5)
5	1.74, td (11.5, 3.9)	1.84, dd (10.4, 3.8)	1.92, m	1.71, m
6	1.72, m 1.09, qd (12.9, 3.4)	1.65, dq (12.8, 3.8) 1.11, dd (13.2, 3.8)	1.63, m 1.04, m	1.54, m 1.51, m
7	1.66, dm (13.0) 0.89, m	1.70, dq (13.2, 3.8) 1.04, m	1.67, m 1.03, m	1.62, m 1.38, td (13.3, 4.6)
8	1.44, m	1.45, m	1.39, m	-
9	1.60, m 1.19, q (12.2)	3.37, t (9.9)	3.37, t (9.9)	1.64, m 1.60, m
10	1.27, tt (11.9, 3.4)	1.22, m	1.21, ddd (12.5, 9.9, 3.4)	1.65, m
11	3.88, dd (4.2, 3.3)	4.41, t (4.6)	3.95, dd (4.8, 3.4)	3.85, brd (5.8)
12	5.87, dq (5.8, 3.3)	5.88, brd (5.7)	6.02, dt (5.7, 1.8)	5.88, d (5.8)
14	1.60, brs	1.63, brs	1.64, s	1.62, s
15	0.92, d (6.5)	1.05, d (6.5)	1.04, d (6.4)	1.27, s
16	-	-	3.43, s	-

Chemical shifts were measured at 700 MHz in CDCl_3._

## Data Availability

All data generated or analyzed during this study are included in this published article and its Supplementary Materials.

## Supplementary Materials

The following supporting information can be downloaded at https://www.mdpi.com/article/10.3390/md24060193/s1. Spectral Data.
