# Supplementary material for: Discovery of New Zosteropenillines from the Seagrass-Derived Fungus Penicillium yezoense KMM 4679 by OSMAC Strategy
Source: Mar Drugs. 2026 May 30;24(6):193. doi: 10.3390/md24060193 (PMC13301470; doi:10.3390/md24060193)

Supporting Information

Article

Discovery of New Zosteropenillines from the Seagrass-Derived Fungus *Penicillium yezoense* KMM 4679 by OSMAC Strategy

Elena V. Leshchenko [1,2,*], Gleb V. Borkunov [1,2], Alexandr S. Antonov [1], Ekaterina A. Chingizova [1], Dmitrii V. Berdyshev [1], Maria A. Solovova [2], Roman S. Popov [1], Ksenia A. Sayankina [3], Yuliya V. Khudyakova [1], Sergey N. Baldaev [1], Natalya Yu. Kim [1], Anatoly I. Kalinovsky [1], Andrey V. Gerasimenko [3], Ekaterina A. Yurchenko [1] and Anton N. Yurchenko [1]

1 G.B. Elyakov Pacific Institute of Bioorganic Chemistry, Far Eastern Branch of the Russian Academy of Sciences, 159 Prospect 100-letiya Vladivostoka, Vladivostok 690022, Russian Federation; antonov_as@piboc.dvo.ru (A.S.A.); martyyas@mail.ru (E.A.Ch); berdyshev@piboc.dvo.ru (D.V.B.); prs_90@mail.ru (R.S.P.); 161070@rambler.ru (Y.V.K.); baldaevsergey@gmail.com (S.N.B); natalya_kim@mail.ru (N.Y.K.); kaaniw@piboc.dvo.ru (A.I.K.); eyurch@piboc.dvo.ru (E.A.Y.); yurchenkoan@piboc.dvo.ru (A.N.Y.).

2 Institute of High Technologies and Advanced Materials, Far Eastern Federal University, Vladivostok 690922, Russian Federation; leschenko.ev@dvfu.ru (E.V.L.); gborkunov@gmail.com (G.V.B.); solovova.ma@dvfu.ru (M.A.S.).

3 Institute of Chemistry, Far Eastern Branch of the Russian Academy of Sciences, 159 Prospect 100-letiya Vladivostoka, Vladivostok 690022, Russian Federation; ksu906090@mail.ru (K.A.S.); gerasimenko@ich.dvo.ru (A.V.G.).

* Correspondence: leshchenko.ev@dvfu.ru, ORCID 0000-0002-9429-5684

Contents

Figure S1. Known zosteropenillines and pallidopenillines are isolated from *P. yezoense* KMM 4679....... 6

Figure S2. (A) Key COSY, **(B)** HMBC and (**C**) ROESY correlations of **1** 7

Figure S3. HRESIMS for **1** 7

Figure S4. ^{1}H NMR spectrum of **1** measured at 700 MHz in $CDCl_3$........ 9

Figure S5. ^{13}C NMR spectrum of **1** measured at 176 MHz in $CDCl_3$........ 10

Figure S6. HSQC spectrum of **1** measured in $CDCl_3$ 11

Figure S7. COSY spectrum of **1** measured in $CDCl_3$ 12

Figure S8. HMBC spectrum of **1** measured in $CDCl_3$ 13

Figure S9. ROESY spectrum of **1** measured in $CDCl_3$ 14

Figure S10. UV spectrum of **1** measured in MeOH........ 21

Figure S11. CD spectrum of **1** measured in MeOH 22

Figure S12. (A) Key COSY, (B) HMBC and (C) ROESY correlations of **2** 23

Figure S13. HRESIMS for **2** 23

Figure S14. ^{1}H NMR spectrum of **2** measured at 700 MHz in $CDCl_3$........ 25

Figure S15. ^{13}C NMR spectrum of **2** measured at 176 MHz in $CDCl_3$........ 26

Figure S16. DEPT-135 spectrum of **2** measured at 176 MHz in $CDCl_3$ 27

Figure S17. HSQC spectrum of **2** measured in $CDCl_3$ 28

Figure S18. COSY spectrum of **2** measured in $CDCl_3$ 29
Figure S19. HMBC spectrum of **2** measured in $CDCl_3$ 30
Figure S20. ROESY spectrum of **2** measured in $CDCl_3$ 31
Figure S21. UV spectrum of **2** measured in MeOH............ 32
Figure S22. CD spectrum of **2** measured in MeOH............ 33
Figure S23. (**A**) Key COSY, (**B**) HMBC and (**C**) ROESY correlations of **3** 34
Figure S24. HRESIMS for **3**............ 34
Figure S25. ^{1}H NMR spectrum of **3** measured at 700 MHz in $CDCl_3$............ 36
Figure S26. ^{13}C NMR spectrum of **3** measured at 176 MHz in $CDCl_3$............ 37
Figure S27. DEPT-135 spectrum of **3** measured at 176 MHz in $CDCl_3$............ 38
Figure S28. HSQC spectrum of **3** measured in $CDCl_3$ 39
Figure S29. COSY spectrum of **3** measured in $CDCl_3$ 40
Figure S30. HMBC spectrum of **3** measured in $CDCl_3$ 41
Figure S31. ROESY spectrum of **3** measured in $CDCl_3$ 42
Figure S32. UV spectrum of **3** measured in MeOH............ 45
Figure S33. CD spectrum of **3** measured in MeOH............ 46
Figure S34. (**A**) Key COSY, (**B**) HMBC and (**C**) ROESY correlations of **4** 47
Figure S35. HRESIMS for **4**............ 47
Figure S36. ^{1}H NMR spectrum of **4** measured at 700 MHz in $CDCl_3$............ 50
Figure S37. ^{13}C NMR spectrum of **4** measured at 176 MHz in $CDCl_3$............ 51
Figure S38. DEPT-135 spectrum of **4** measured at 176 MHz in $CDCl_3$............ 52
Figure S39. HSQC spectrum of **4** measured in $CDCl_3$ 53
Figure S40. COSY spectrum of **4** measured in $CDCl_3$ 54
Figure S41. HMBC spectrum of **4** measured in $CDCl_3$ 55
Figure S42. ROESY spectrum of **4** measured in $CDCl_3$............ 56
Figure S43. UV spectrum of **4** measured in MeOH............ 57
Figure S44. CD spectrum of **4** measured in MeOH............ 58
Figure S45. (**A**) Key COSY, (**B**) HMBC and (**C**) ROESY correlations of **5** 59
Figure S46. HRESIMS for **5**............ 59
Figure S47. ^{1}H NMR spectrum of **5** measured at 700 MHz in $CDCl_3$............ 61
Figure S48. ^{13}C NMR spectrum of **5** measured at 176 MHz in $CDCl_3$............ 62
Figure S49. DEPT-135 spectrum of **5** measured at 176 MHz in $CDCl_3$............ 63
Figure S50. HSQC spectrum of **5** measured in $CDCl_3$ 64
Figure S51. COSY spectrum of **5** measured in $CDCl_3$ 65
Figure S52. HMBC spectrum of **5** measured in $CDCl_3$ 66
Figure S53. ROESY spectrum of **5** measured in $CDCl_3$............ 67
Figure S54. UV spectrum of **5** measured in $CDCl_3$ 68

Figure S55. CD spectrum of **5** measured in $CDCl_3$ 69
Figure S56. (**A**) Key COSY, (**B**) HMBC and (**C**) ROESY correlations of **6** 70
Figure S57. HRESIMS for **6** 70
Figure S58. ^{1}H NMR spectrum of **6** measured at 700 MHz in $CDCl_3$ 72
Figure S59. ^{13}C NMR spectrum of **6** measured at 176 MHz in $CDCl_3$ 73
Figure S60. DEPT-135 spectrum of **6** measured at 176 MHz in $CDCl_3$ 74
Figure S61. HSQC spectrum of **6** measured in $CDCl_3$ 75
Figure S62. COSY spectrum of **6** measured in $CDCl_3$ 76
Figure S63. HMBC spectrum of **6** measured in $CDCl_3$ 77
Figure S64. ROESY spectrum of **6** measured in $CDCl_3$ 78
Figure S65. UV spectrum of **6** measured in MeOH 80
Figure S66. CD spectrum of **6** measured in MeOH 81
Figure S67. (**A**) Key COSY, (**B**) HMBC and (**C**) ROESY correlations of **7** 82
Figure S68. HRESIMS for **7** 82
Figure S69. ^{1}H NMR spectrum of **7** measured at 700 MHz in $CDCl_3$ 84
Figure S70. ^{13}C NMR spectrum of **7** measured at 176 MHz in $CDCl_3$ 85
Figure S71. DEPT-135 spectrum of **7** measured at 176 MHz in $CDCl_3$ 86
Figure S72. HSQC spectrum of **7** measured in $CDCl_3$ 87
Figure S72. COSY spectrum of **7** measured in $CDCl_3$ 88
Figure S74. HMBC spectrum of **7** measured in $CDCl_3$ 89
Figure S75. ROESY spectrum of **7** measured in $CDCl_3$ 90
Figure S76. (**A**) Key COSY, (**B**) HMBC and (**C**) ROESY correlations of **8** 91
Figure S77. HRESIMS for **8** 91
Figure S78. ^{1}H NMR spectrum of **8** measured at 700 MHz in $CDCl_3$ 94
Figure S79. ^{13}C NMR spectrum of **8** measured at 176 MHz in $CDCl_3$ 95
Figure S80. DEPT-135 spectrum of **8** measured at 176 MHz in $CDCl_3$ 96
Figure S81. HSQC spectrum of **8** measured in $CDCl_3$ 97
Figure S82. COSY spectrum of **8** measured in $CDCl_3$ 98
Figure S83. HMBC spectrum of **8** measured in $CDCl_3$ 99
Figure S84. ROESY spectrum of **8** measured in $CDCl_3$ 100
Figure S85. (A) Key COSY and (B) HMBC correlations of **9** 103
Figure S86. HRESIMS for **9** 103
Figure S87. ^{1}H NMR spectrum of **9** measured at 700 MHz in $CDCl_3$ 105
Figure S88. ^{13}C NMR spectrum of **9** measured at 176 MHz in $CDCl_3$ 106
Figure S89. DEPT-135 spectrum of **9** measured at 176 MHz in $CDCl_3$ 107
Figure S90. HSQC spectrum of **9** measured in $CDCl_3$ 108
Figure S91. COSY spectrum of **9** measured in $CDCl_3$ 109

Figure S92. HMBC spectrum of **9** measured in $CDCl_3$ 110
Figure S93. ROESY spectrum of **9** measured in $CDCl_3$ 111
Figure S94. UV spectrum of **9** measured in MeOH 112
Figure S95. CD spectrum of **9** measured in MeOH 113
Figure S96. (A) Key COSY and (B) HMBC correlations of **10** 114
Figure S97. HRESIMS for **10** 114
Figure S98. ^{1}H NMR spectrum of **10** measured at 700 MHz in $CDCl_3$ 117
Figure S99. ^{13}C NMR spectrum of **10** measured at 176 MHz in $CDCl_3$ 118
Figure S100. DEPT-135 spectrum of **10** measured at 176 MHz in $CDCl_3$ 119
Figure S101. HSQC spectrum of **10** measured in $CDCl_3$ 120
Figure S102. COSY spectrum of **10** measured in $CDCl_3$ 121
Figure S103. HMBC spectrum of **10** measured in $CDCl_3$ 122
Figure S104. ROESY spectrum of **10** measured in $CDCl_3$ 123
Figure S105. UV spectrum of **10** measured in MeOH 124
Figure S106. CD spectrum of **10** measured in MeOH 125
Figure S107. (**A**) Key COSY, (**B**) HMBC and (**C**) ROESY correlations of **11** 126
Figure S108. HRESIMS for **11** 127
Figure S109. ^{1}H NMR spectrum of **11** measured at 700 MHz in $CDCl_3$ 129
Figure S110. ^{13}C NMR spectrum of **11** measured at 176 MHz in $CDCl_3$ 130
Figure S111. DEPT-135 spectrum of **11** measured at 176 MHz in $CDCl_3$ 131
Figure S112. HSQC spectrum of **11** measured in $CDCl_3$ 132
Figure S113. COSY spectrum of **11** measured in $CDCl_3$ 133
Figure S114. HMBC spectrum of **11** measured in $CDCl_3$ 134
Figure S115. ROESY spectrum of **11** measured in $CDCl_3$ 135
Figure S116. (**A**) Key COSY, (**B**) HMBC and (**C**) ROESY correlations of **12** 138
Figure S117. HRESIMS for **12** 139
Figure S118. ^{1}H NMR spectrum of **12** measured at 700 MHz in $CDCl_3$ 140
Figure S119. ^{13}C NMR spectrum of **12** measured at 176 MHz in $CDCl_3$ 141
Figure S120. DEPT-135 spectrum of **12** measured at 176 MHz in $CDCl_3$ 142
Figure S121. HSQC spectrum of **12** measured in $CDCl_3$ 143
Figure S122. COSY spectrum of **12** measured in $CDCl_3$ 144
Figure S123. HMBC spectrum of **12** measured in $CDCl_3$ 145
Figure S124. ROESY spectrum of **12** measured in $CDCl_3$ 146
Figure S125. UV spectrum of **12** measured in MeOH 150
Figure S126. CD spectrum of **12** measured in MeOH 151
Figure S127. (**A**) Key COSY, (**B**) HMBC and (**C**) ROESY correlations of **13** 152
Figure S128. HRESIMS for **13** 152

Figure S129. ^{1}H NMR spectrum of **13** measured at 700 MHz in $CDCl_3$ 155
Figure S130. ^{13}C NMR spectrum of **13** measured at 176 MHz in $CDCl_3$ 156
Figure S131. DEPT-135 spectrum of **13** measured at 176 MHz in $CDCl_3$ 157
Figure S132. HSQC spectrum of **13** measured in $CDCl_3$ 158
Figure S133. COSY spectrum of **13** measured in $CDCl_3$ 159
Figure S135. ROESY spectrum of **13** measured in $CDCl_3$ 161
Figure S136. UV spectrum of **13** measured in MeOH 164
Figure S137. CD spectrum of **13** measured in MeOH 165
Table S1. Crystal data and structure refinement for **28** 166
Table S2. Bond lengths and angles for **28** 167
Table S3. Hydrogen bonds for **28** 168
Figure S138. A fragment of a layer of stacked and interconnected hydrogen bonds O–H···O, C–H···Cl and C–H···O molecules $C_{15}H_{25}ClO_4$ and H_2O, depicted in two projections: parallel to the plane (011) – **a** and (110) – **b** 169
Figure S139. The crystal structure of $C_{15}H_{25}ClO_4 \cdot H_2O$ 170
Table S4. Calculated and experimental chemical shifts (δ_C/ppm) for compound **1** 171
Figure S140. Dependence of the δ_C calculated and δ_C experimental chemical shifts for compound **1** ... 172
Figure S141. Comparison of calculated values for two conformers (12*R* and 12*S*) for compound **1**...... 173
Figure S142. HPLC of fungal extracts cultivated on Rice Media (RM) and RM + $MgCl_2$ 174

Figure S1. Known zosteropenillines and pallidopenillines are isolated from *P. yezoense* KMM 4679

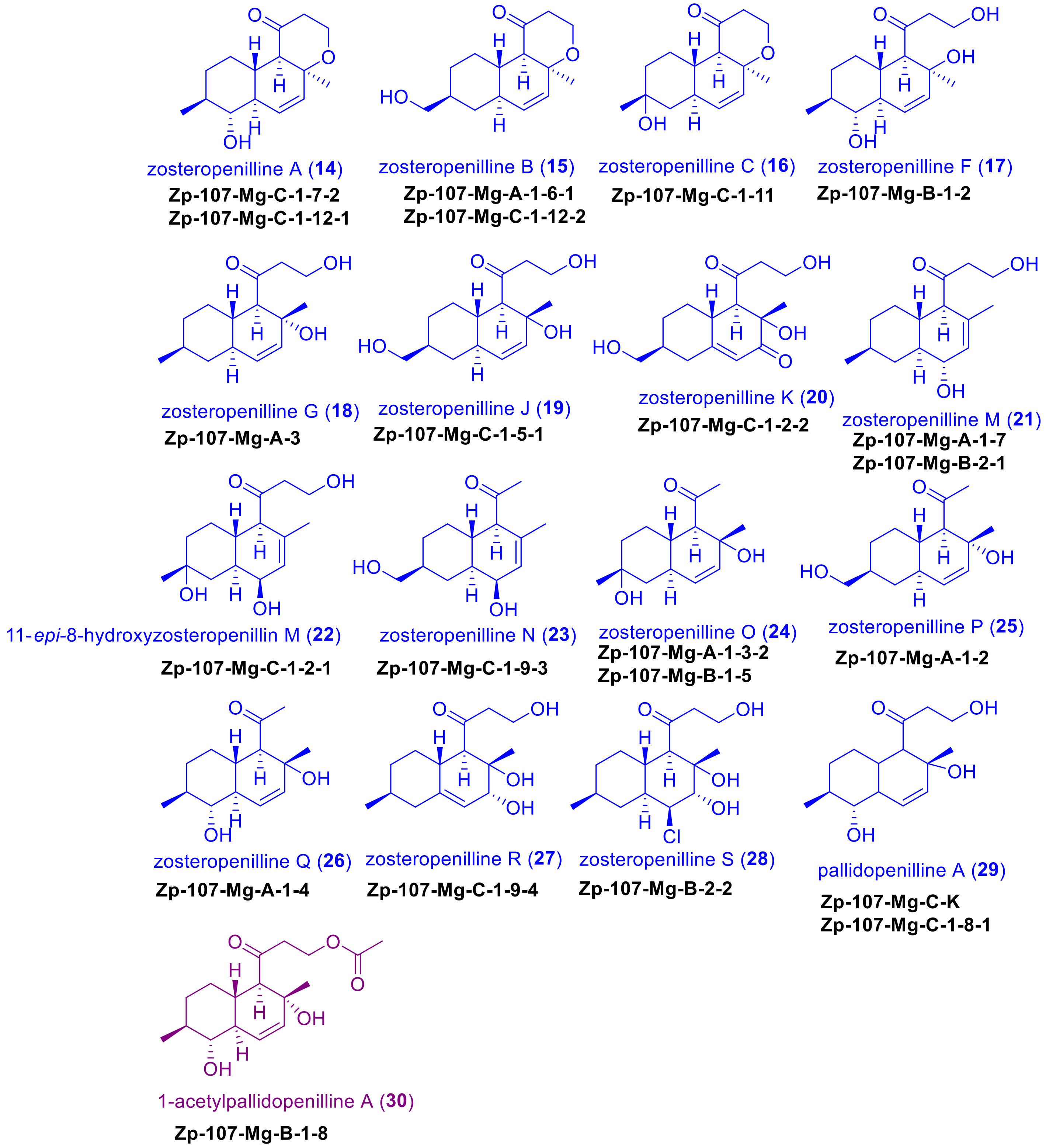

Figure S2. **(A)** Key COSY, **(B)** HMBC and (**C**) ROESY correlations of **1**

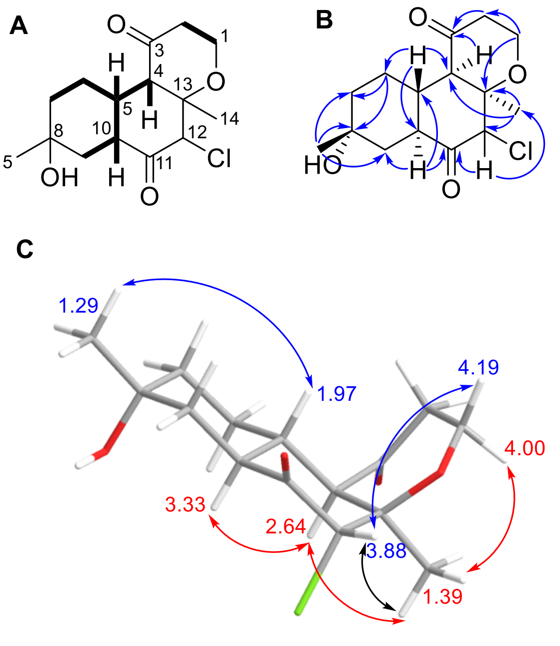

Figure S3. HRESIMS for **1**

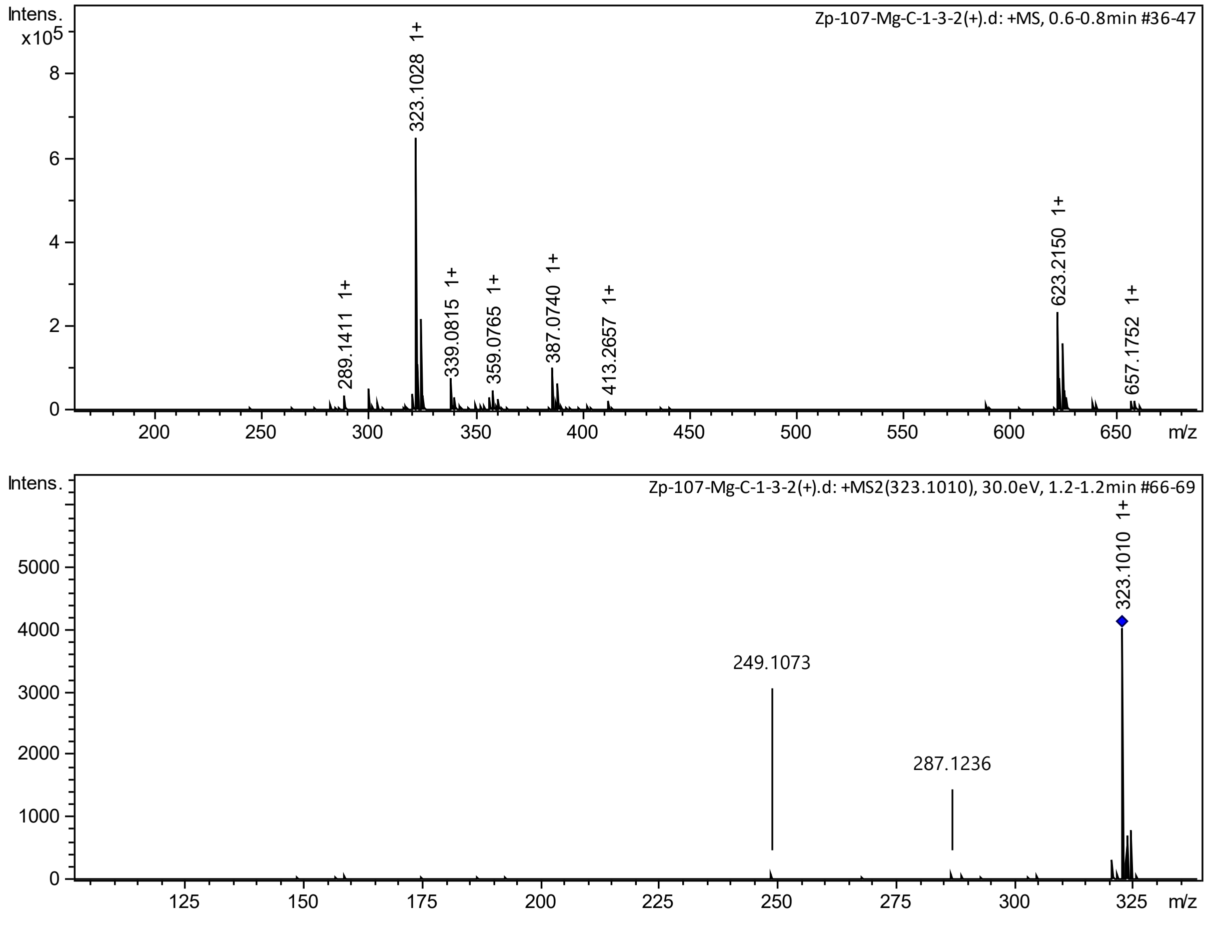

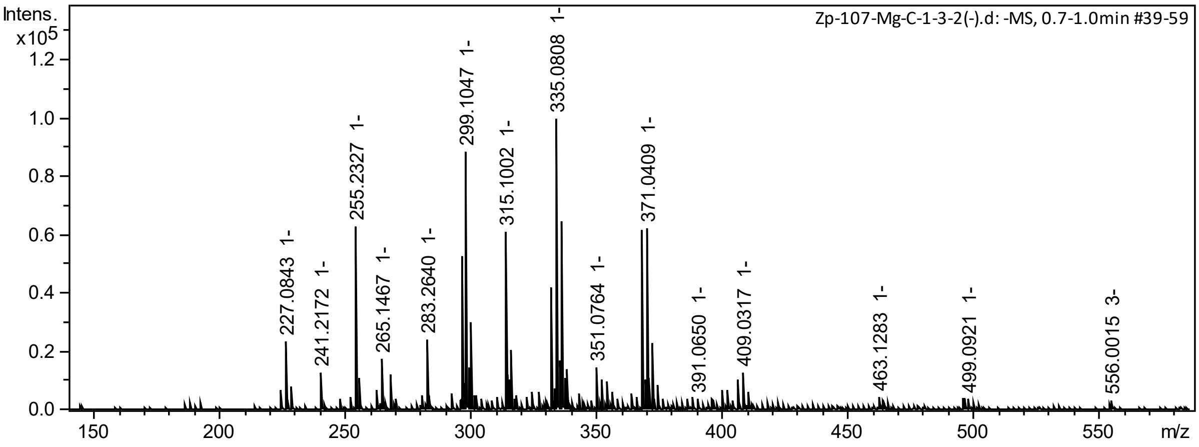

	meas	calc	Δ (ppm)
$[M+Na]^+$	323,1028	323,1021	-2,2

Figure S4. ^{1}H NMR spectrum of **1** measured at 700 MHz in $CDCl_3$

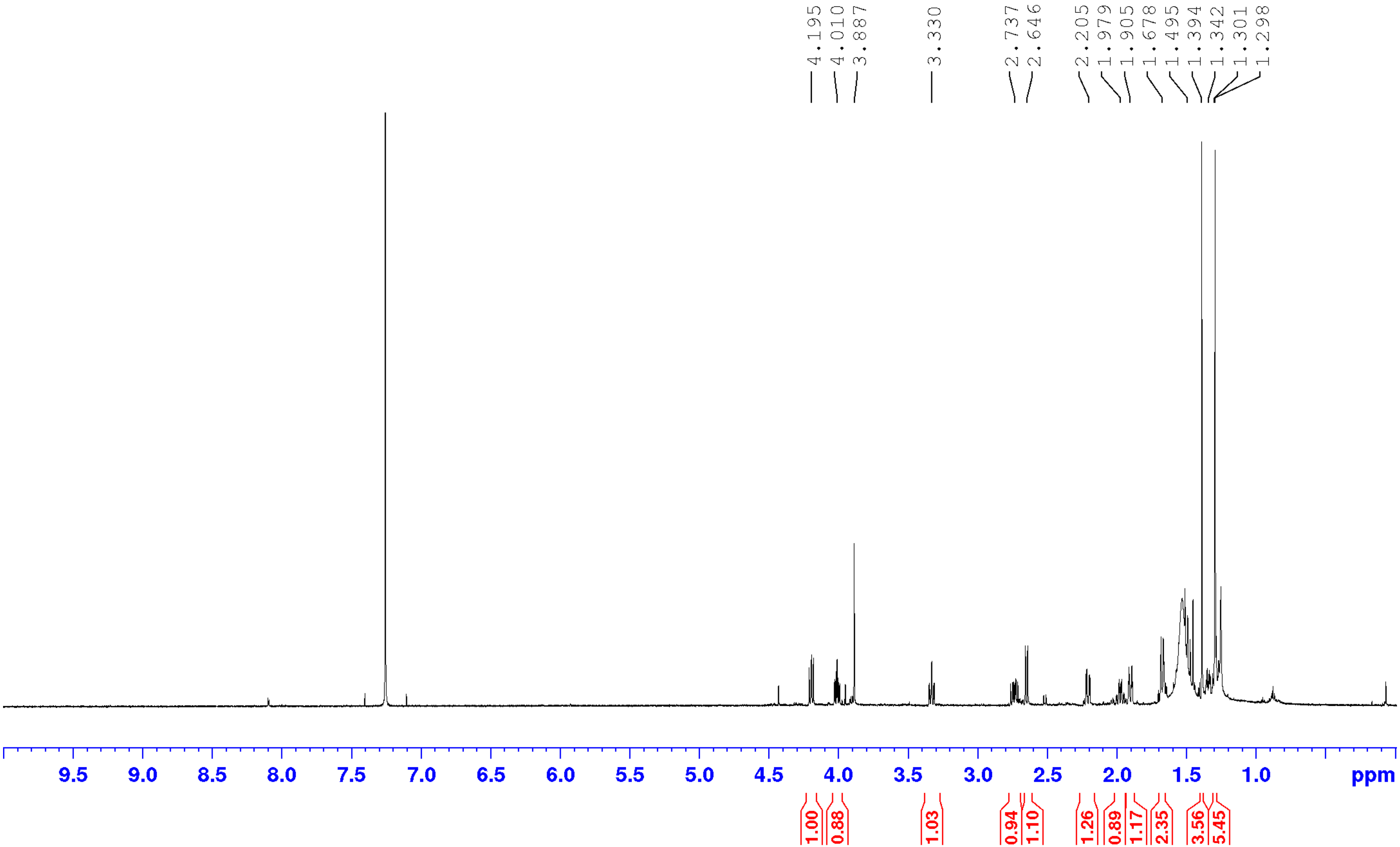

Figure S5. ^{13}C NMR spectrum of **1** measured at 176 MHz in $CDCl_3$

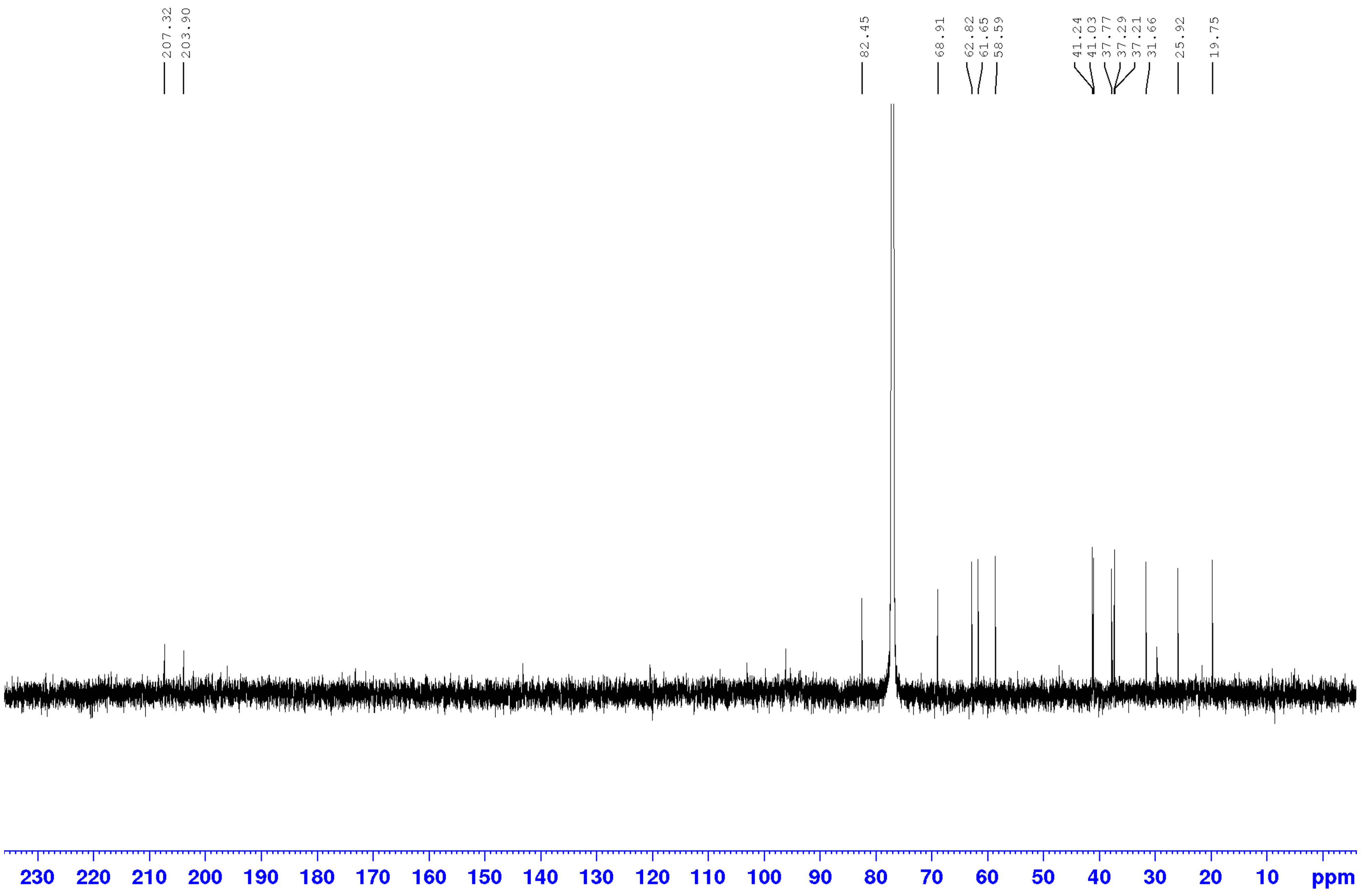

Figure S6. HSQC spectrum of **1** measured in $CDCl_3$

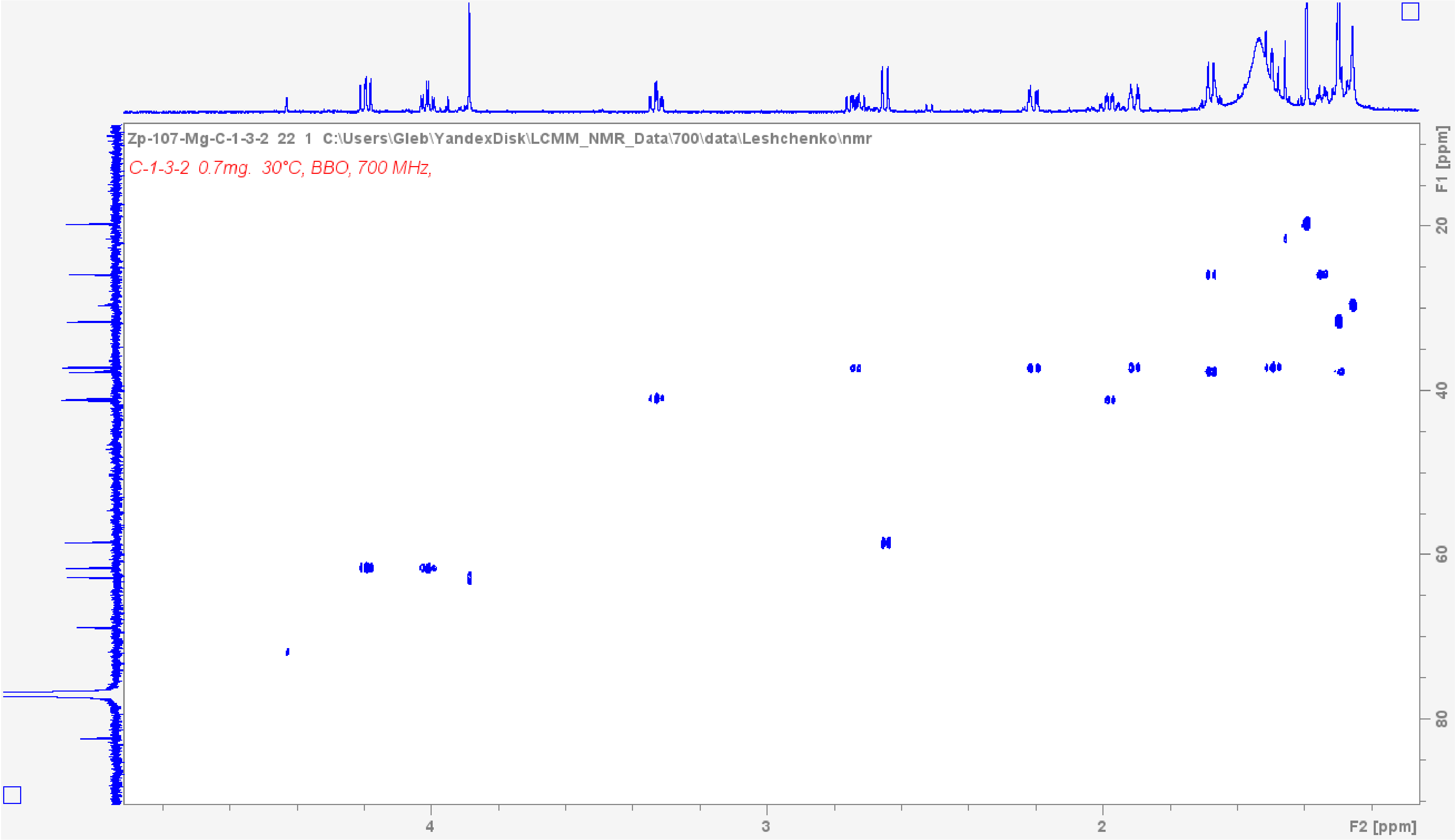

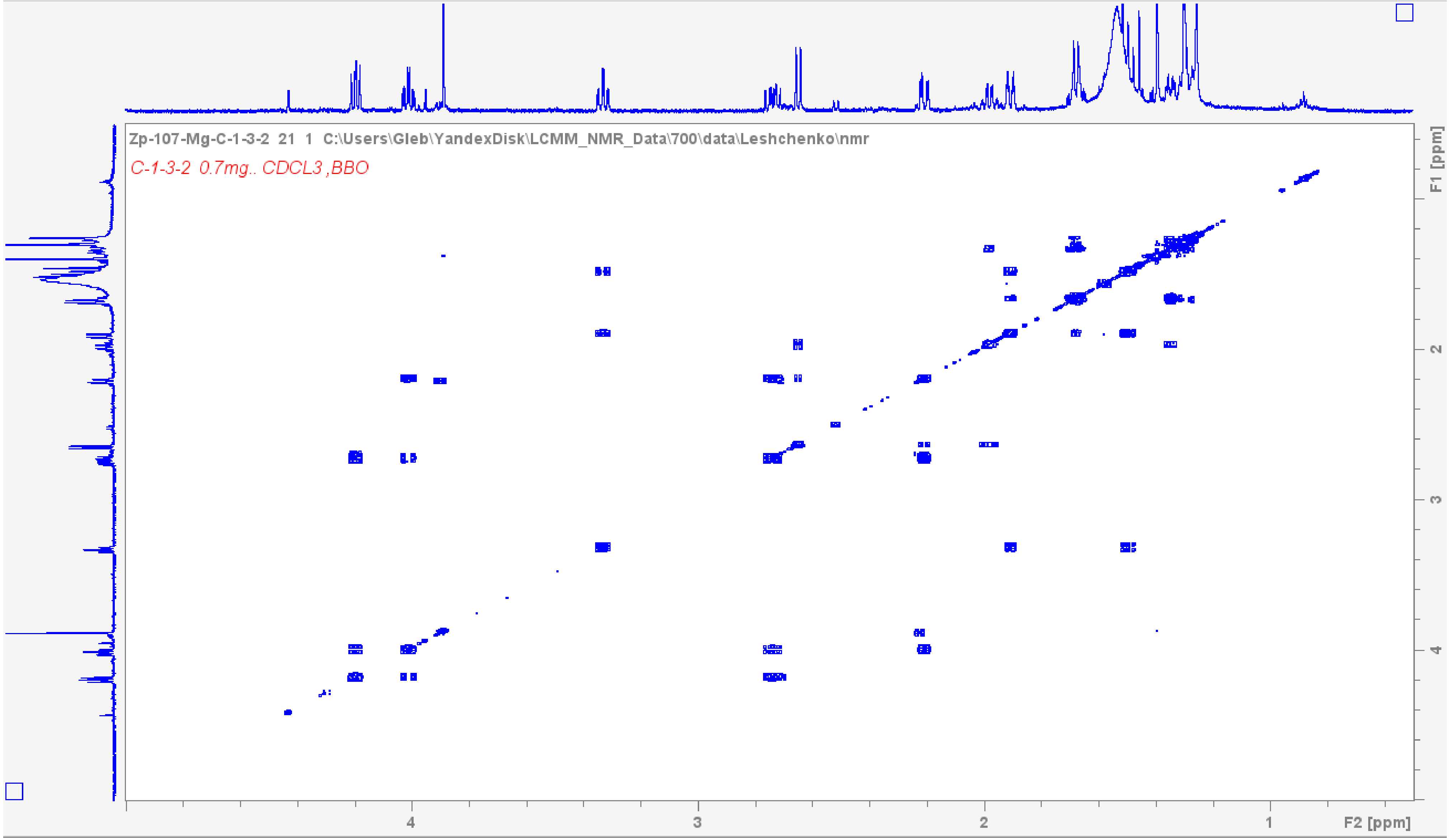

Figure S7. COSY spectrum of **1** measured in $CDCl_3$

Figure S8. HMBC spectrum of **1** measured in $CDCl_3$

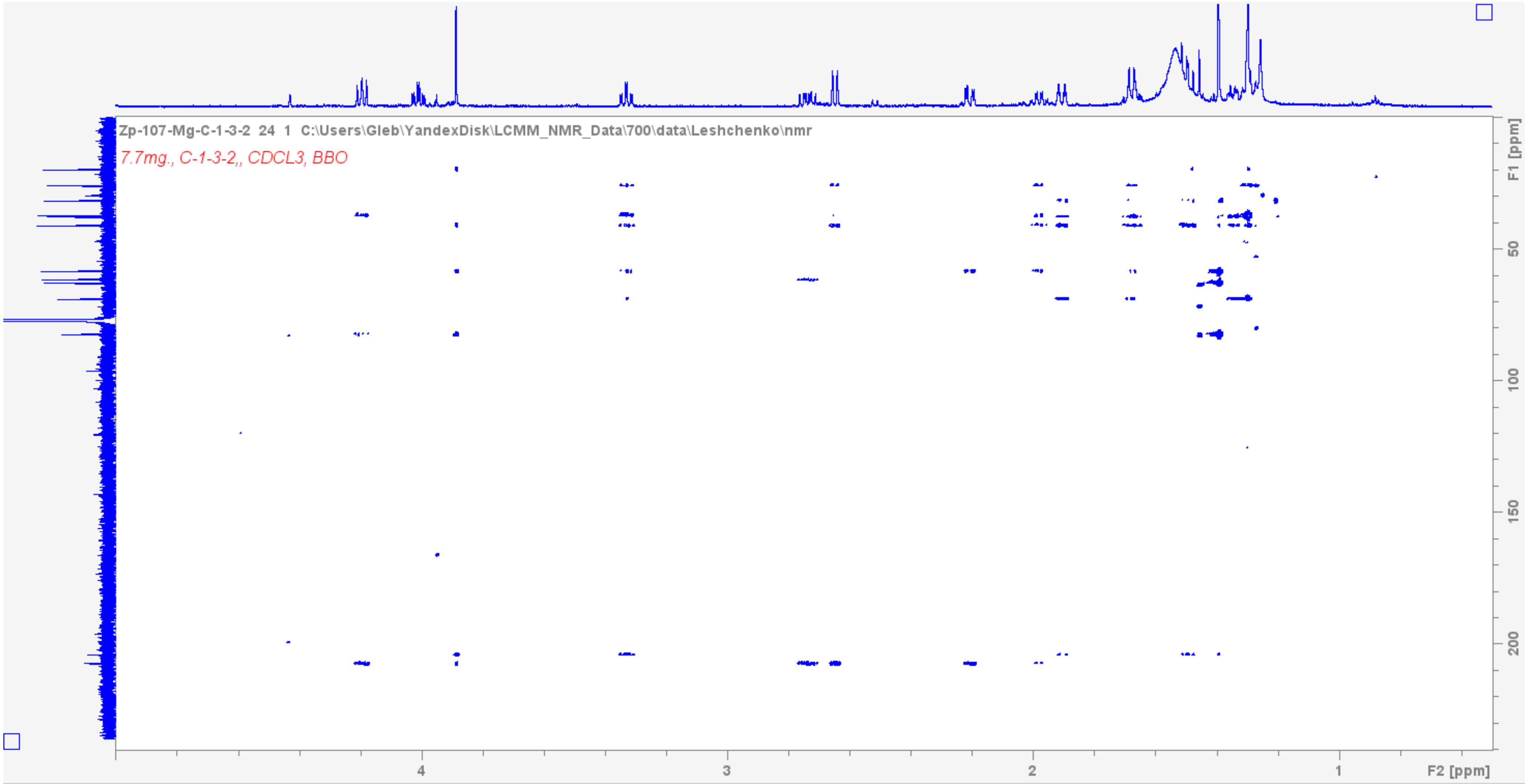

Figure S9. ROESY spectrum of **1** measured in $CDCl_3$

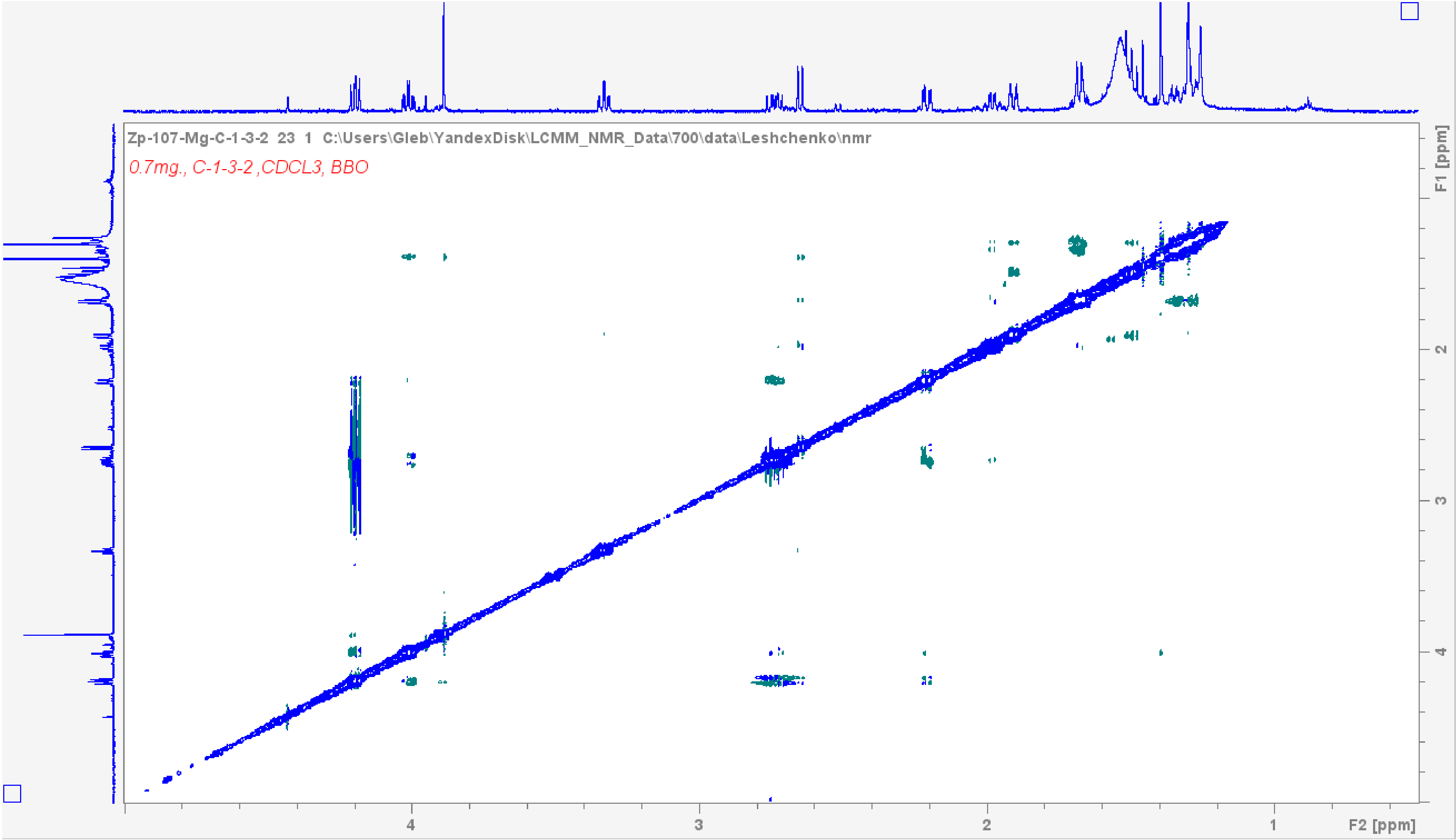

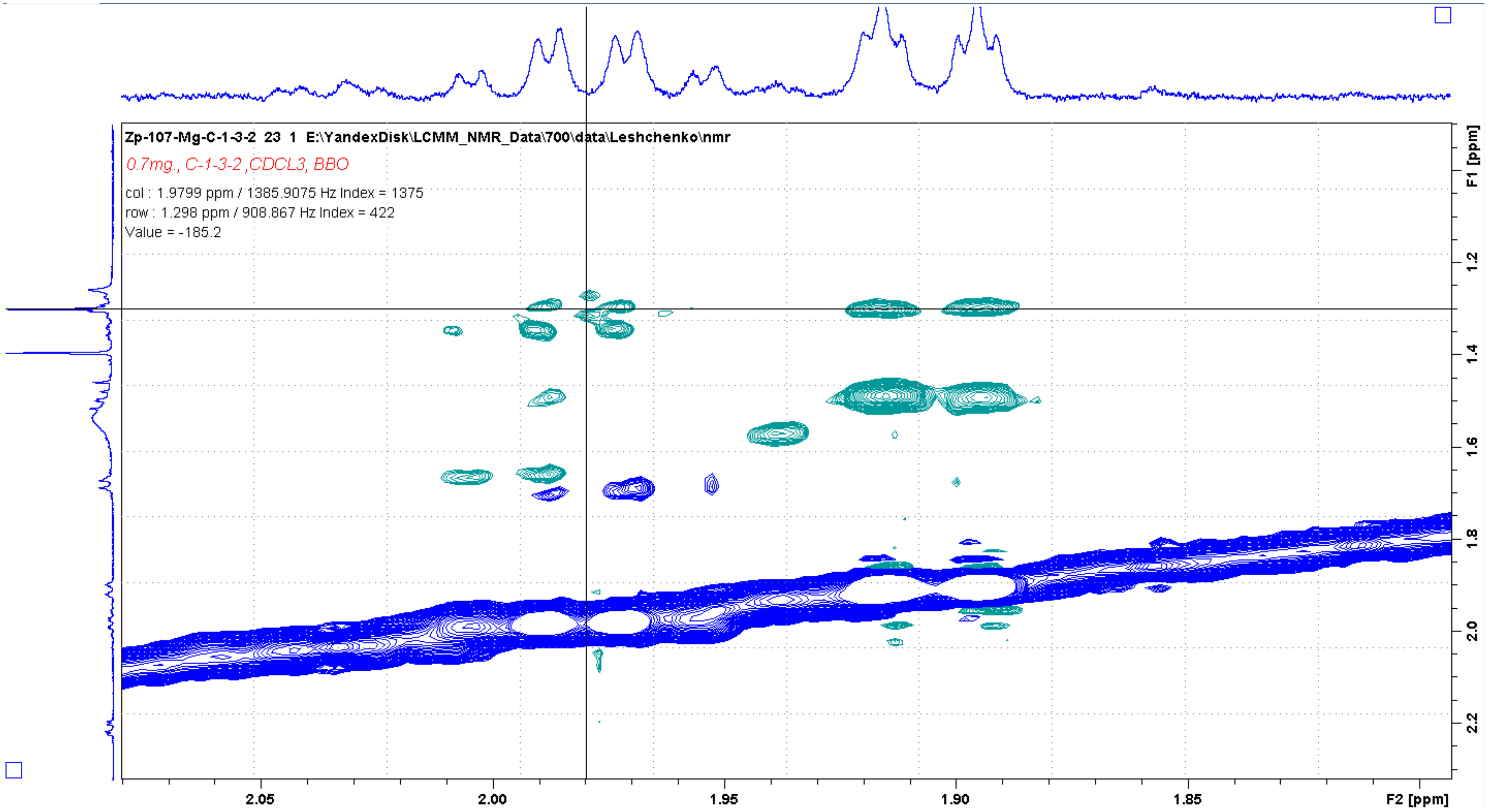

Zp-107-Mg-C-1-3-2 23 1 E:\YandexDisk\LCMM_NMR_Data\700\data\Leshchenko\nmr
0.7mg., C-1-3-2 ,CDCL3, BBO
col : 1.9799 ppm / 1385.9075 Hz Index = 1375
row : 1.298 ppm / 908.867 Hz Index = 422
Value = -185.2
F1 [ppm]
1.2
1.4
1.6
1.8
2.0
2.2
2.05
2.00
1.95
1.90
1.85
F2 [ppm]

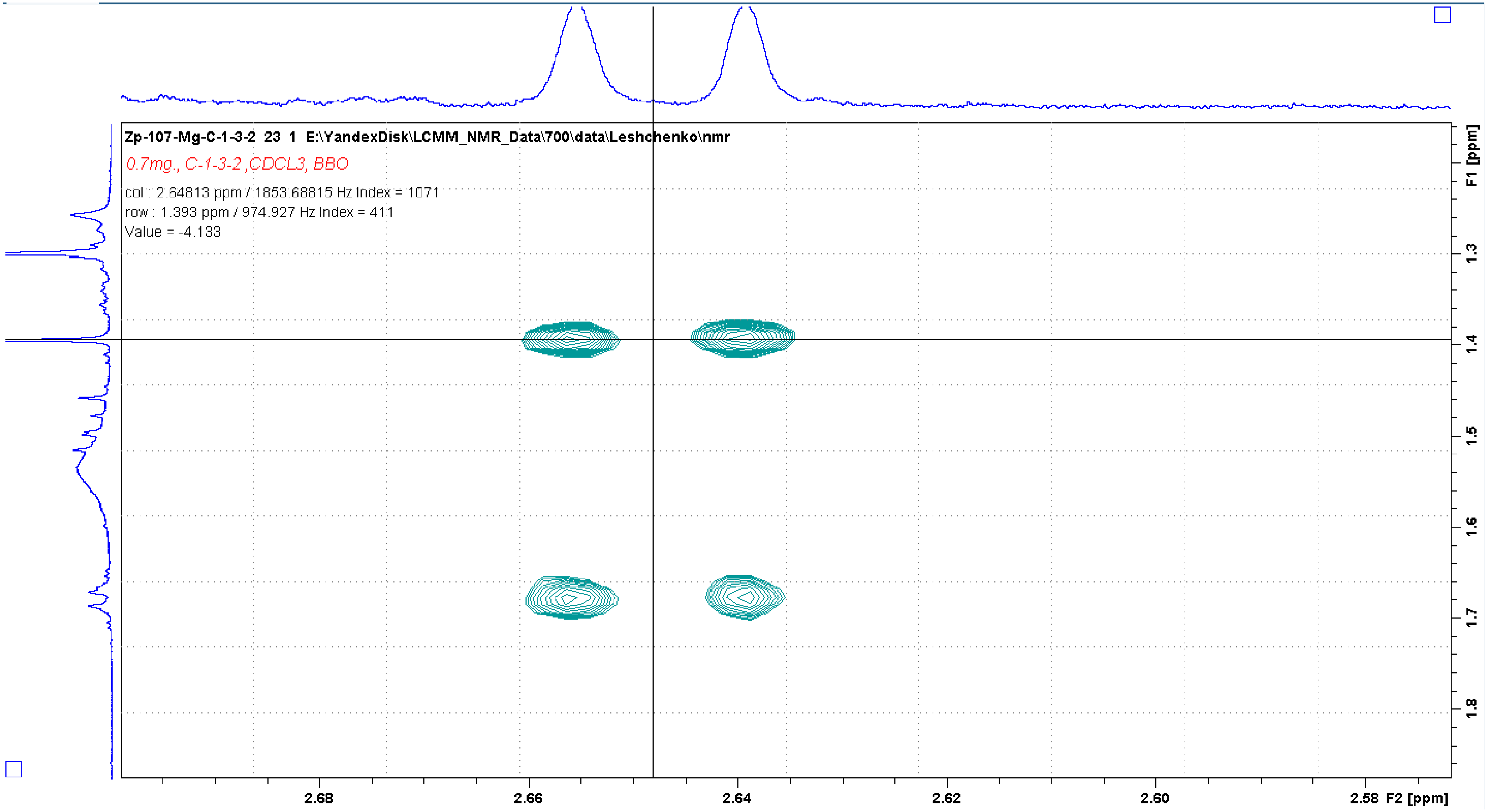
Zp-107-Mg-C-1-3-2 23 1 E:\YandexDisk\LCMM_NMR_Data\700\data\Leshchenko\nmr
0.7mg., C-1-3-2 ,CDCL3, BBO
col : 2.64813 ppm / 1853.68815 Hz Index = 1071
row : 1.393 ppm / 974.927 Hz Index = 411
Value = -4.133
F1 [ppm]
1.3
1.4
1.5
1.6
1.7
1.8
2.68
2.66
2.64
2.62
2.60
2.58
F2 [ppm]

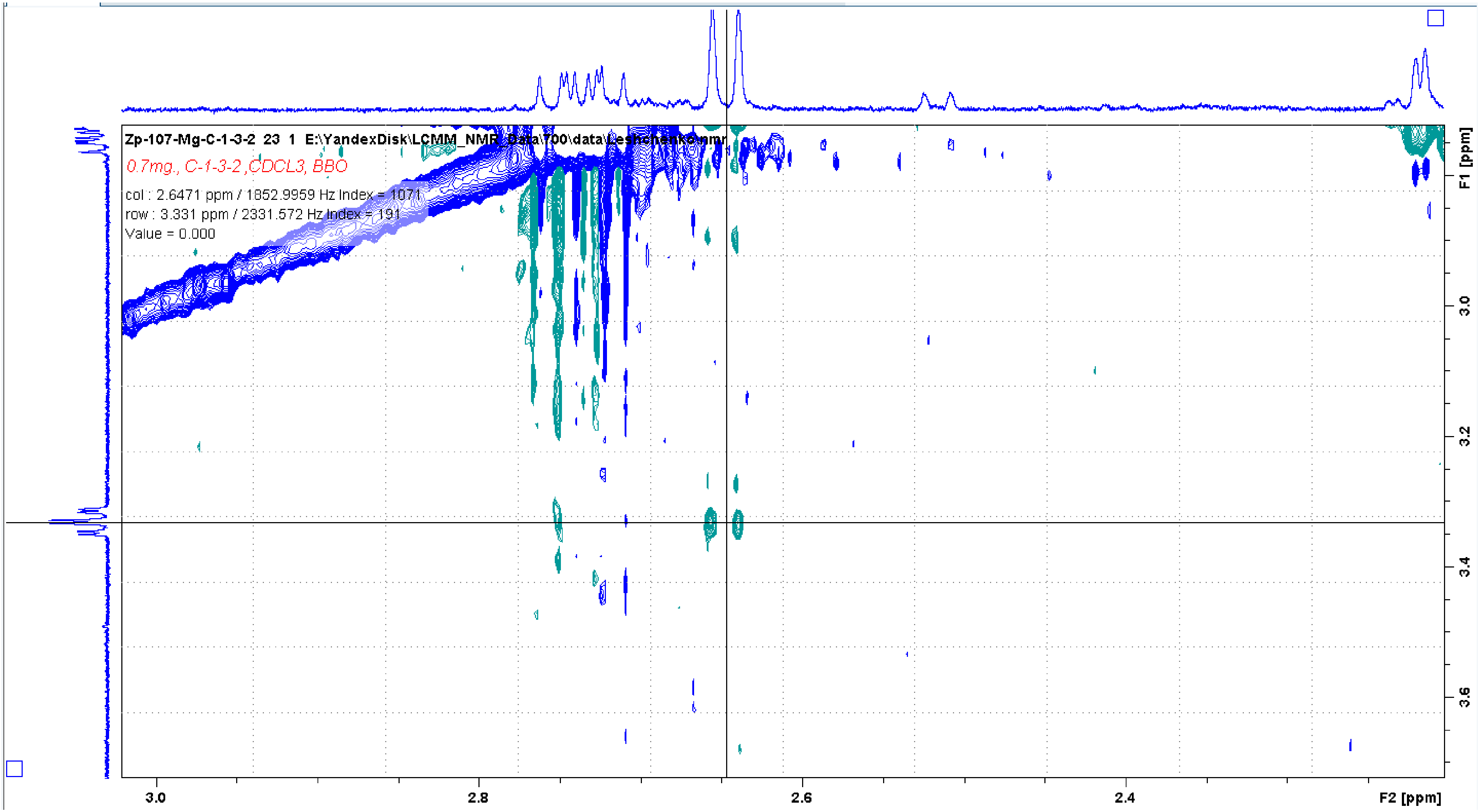

Zp-107-Mg-C-1-3-2 23 1 E:\YandexDisk\LCMM_NMR_Data\700\data\Leshchenko\nmr
0.7mg., C-1-3-2 ,CDCL3, BBO
col : 2.6471 ppm / 1852.9959 Hz Index = 1071
row : 3.331 ppm / 2331.572 Hz Index = 191
Value = 0.000
F1 [ppm]
3.0
3.2
3.4
3.6
3.0
2.8
2.6
2.4
F2 [ppm]

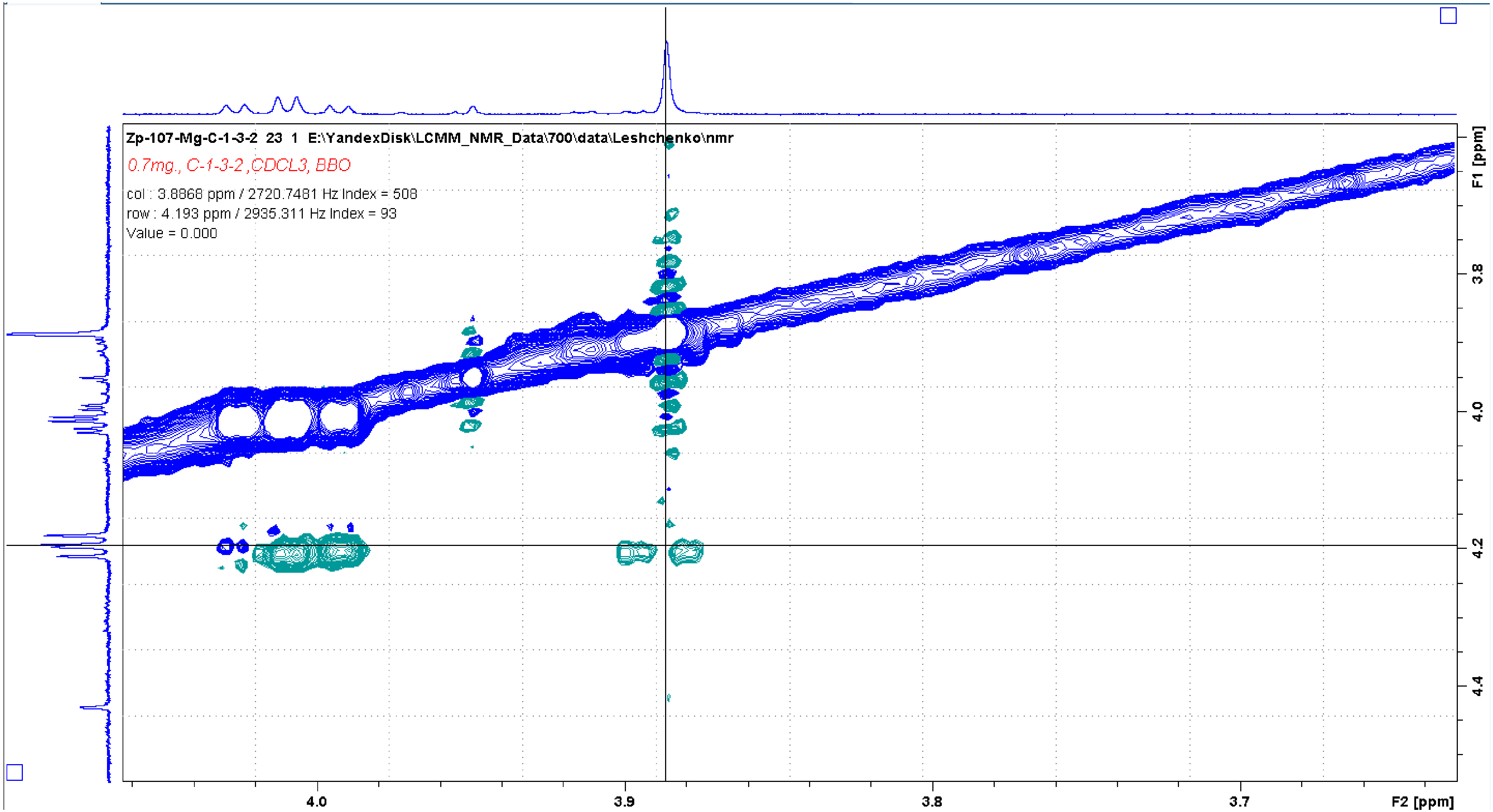
Zp-107-Mg-C-1-3-2 23 1 E:\YandexDisk\LCMM_NMR_Data\700\data\Leshchenko\nmr
0.7mg., C-1-3-2 ,CDCL3, BBO
col : 3.8868 ppm / 2720.7481 Hz Index = 508
row : 4.193 ppm / 2935.311 Hz Index = 93
Value = 0.000
F1 [ppm]
3.8
4.0
4.2
4.4
4.0
3.9
3.8
3.7
F2 [ppm]

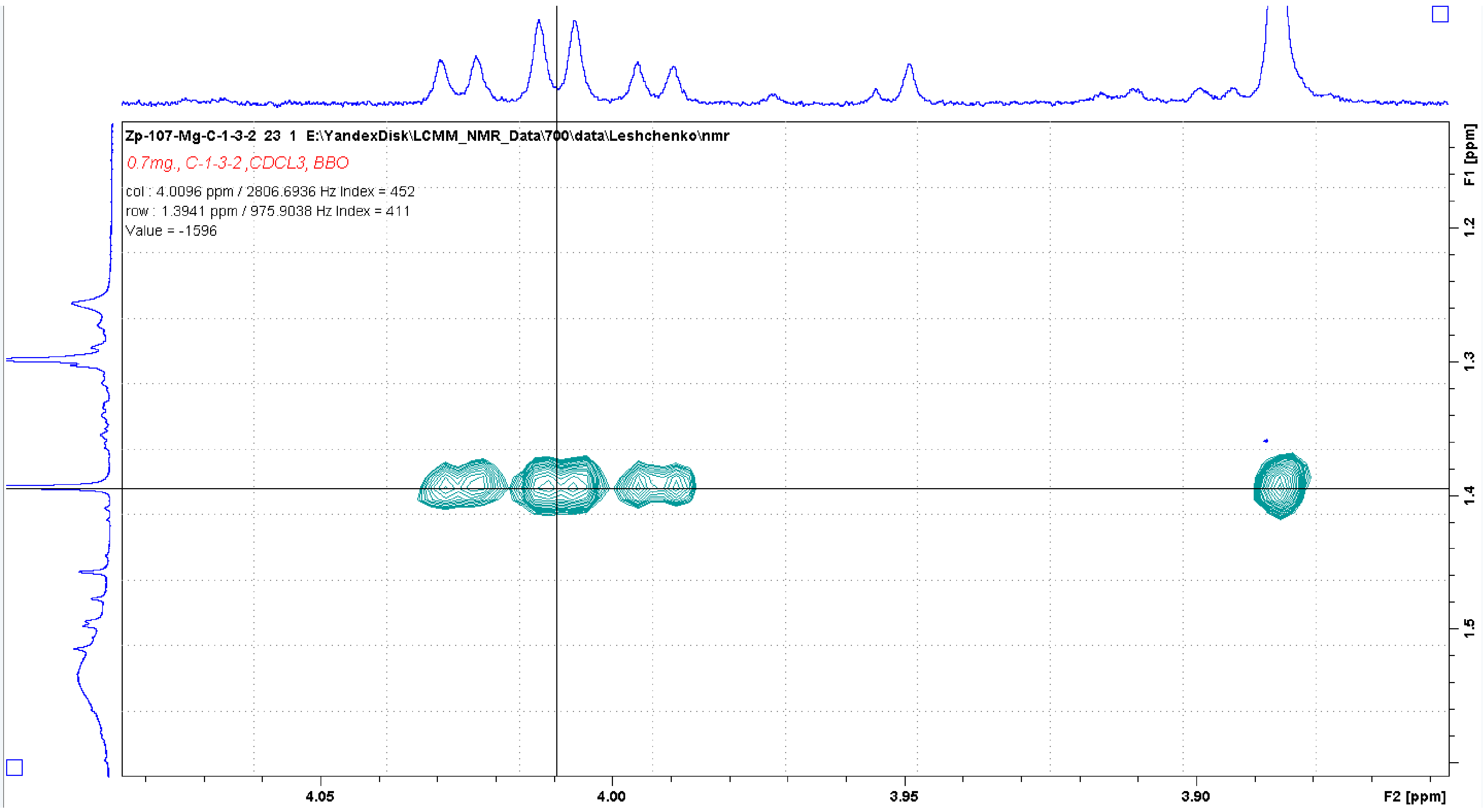
Zp-107-Mg-C-1-3-2 23 1 E:\YandexDisk\LCMM_NMR_Data\700\data\Leshchenko\nmr
0.7mg., C-1-3-2 ,CDCL3, BBO
col : 4.0096 ppm / 2806.6936 Hz Index = 452
row : 1.3941 ppm / 975.9038 Hz Index = 411
Value = -1596
F1 [ppm]
1.2
1.3
1.4
1.5
4.05
4.00
3.95
3.90
F2 [ppm]

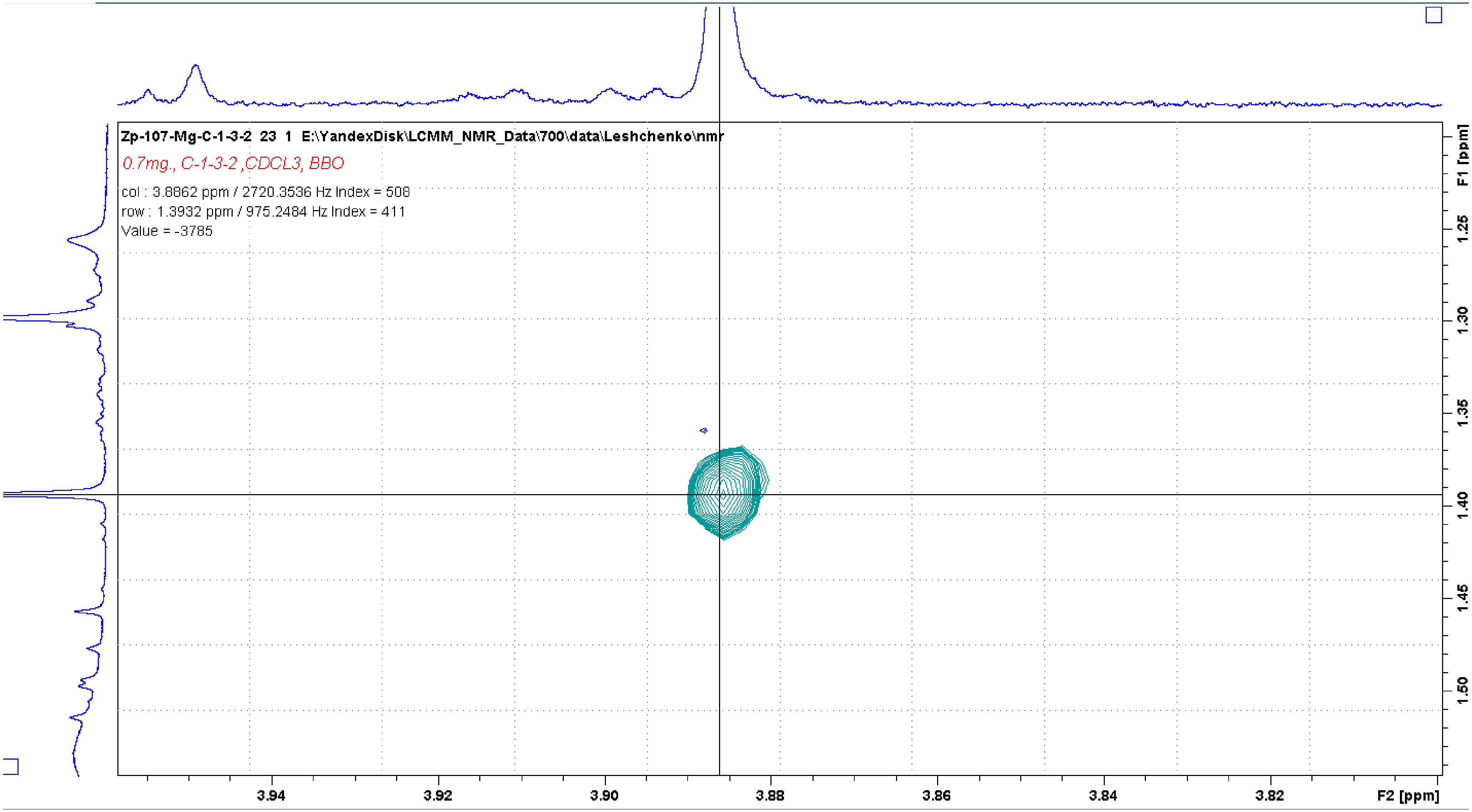

Zp-107-Mg-C-1-3-2 23 1 E:\YandexDisk\LCMM_NMR_Data\700\data\Leshchenko\nmr
0.7mg., C-1-3-2 ,CDCL3, BBO
col : 3.8862 ppm / 2720.3536 Hz Index = 508
row : 1.3932 ppm / 975.2484 Hz Index = 411
Value = -3785
F1 [ppm]
1.25
1.30
1.35
1.40
1.45
1.50
3.94
3.92
3.90
3.88
3.86
3.84
3.82
F2 [ppm]

Figure S10. UV spectrum of **1** measured in MeOH

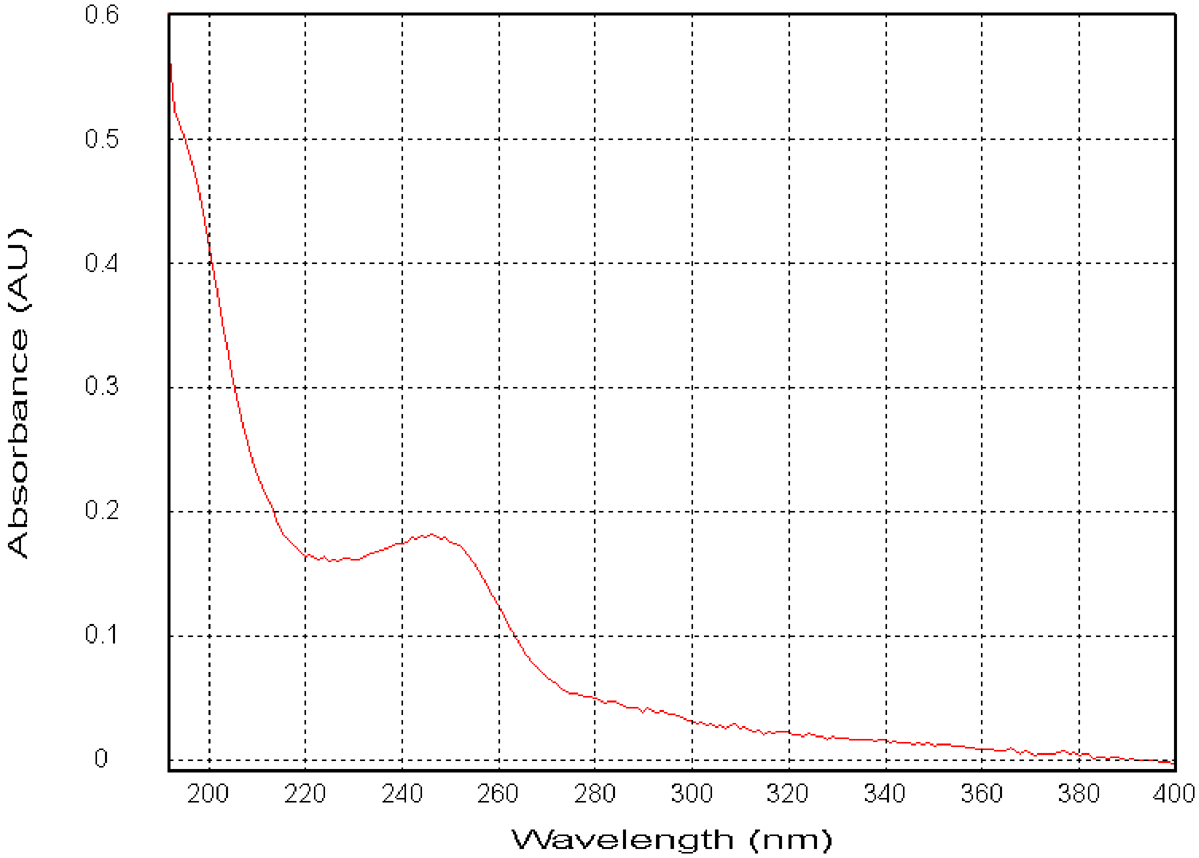

Figure S11. CD spectrum of **1** measured in MeOH

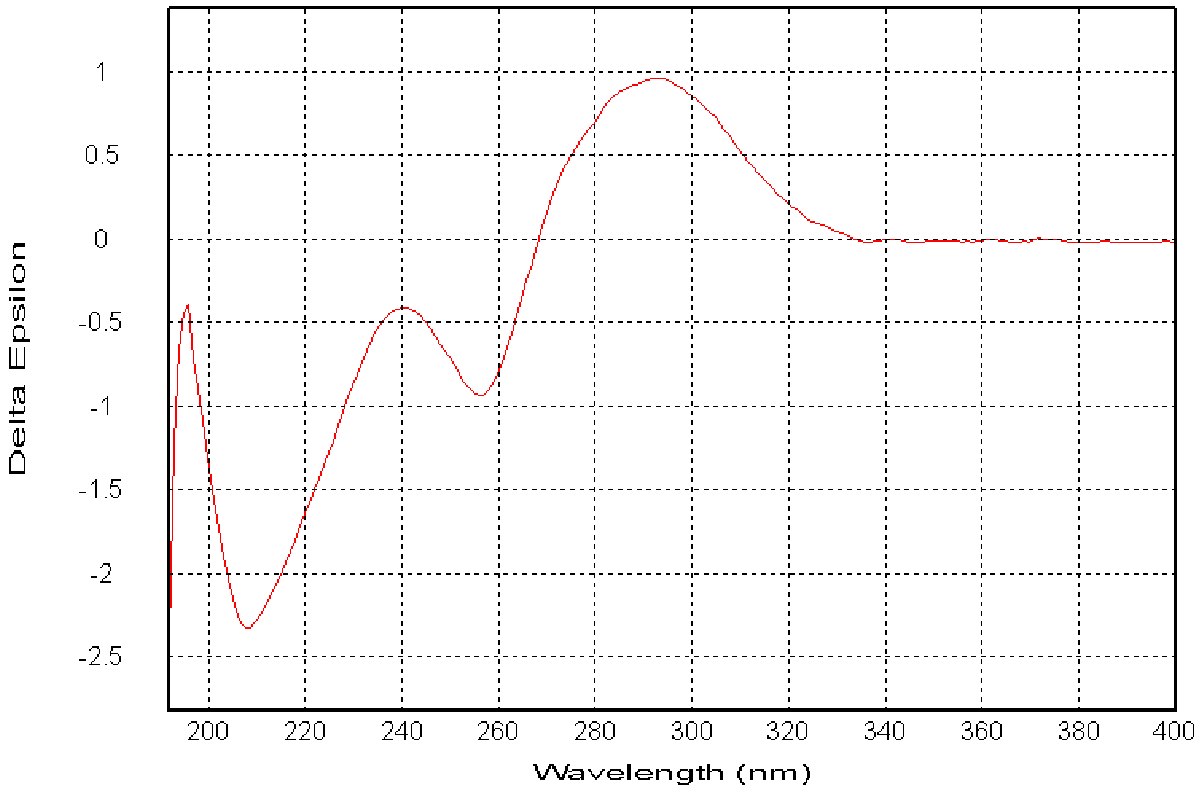

Figure S12. (A) Key COSY, (B) HMBC and (C) ROESY correlations of **2**

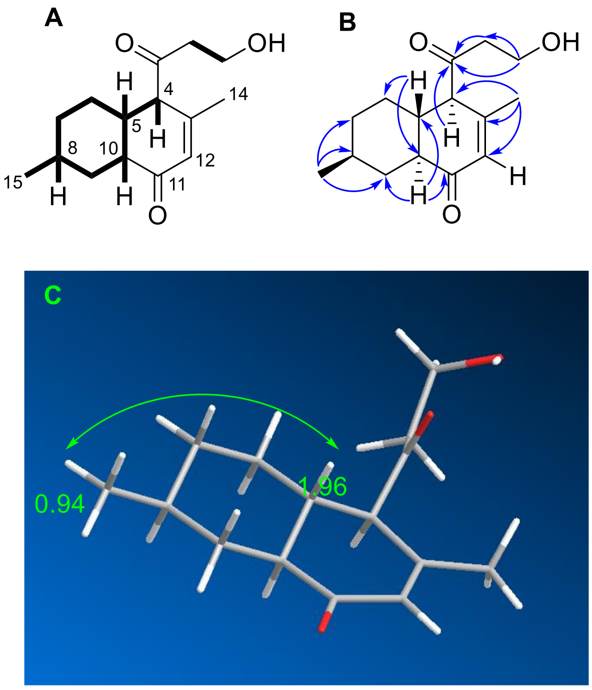

Figure S13. HRESIMS for **2**

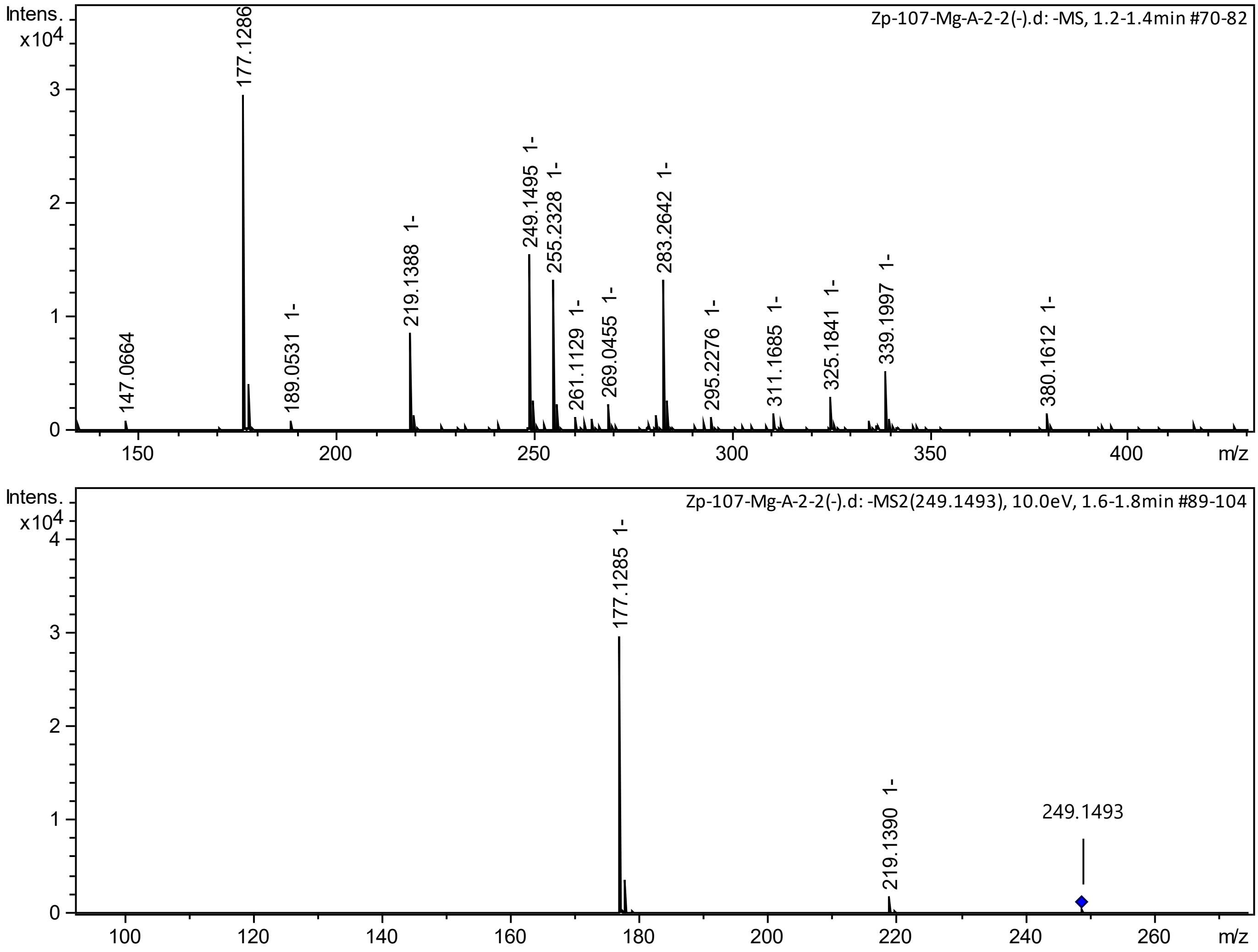

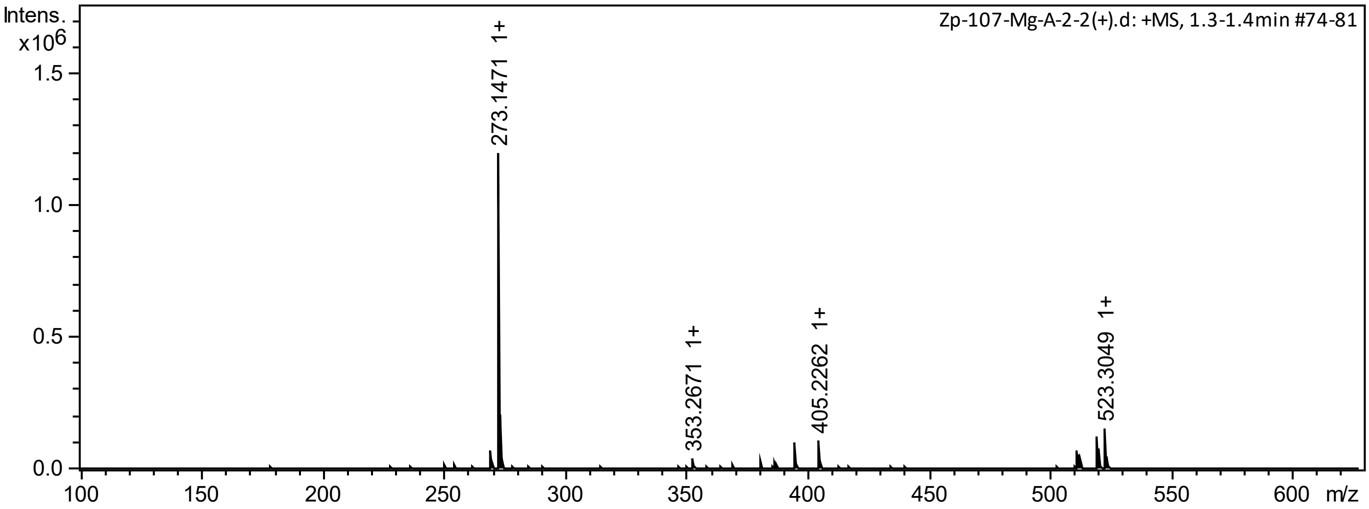
Intens.
x10^6
Zp-107-Mg-A-2-2(+).d: +MS, 1.3-1.4min #74-81
273.1471 1+
353.2671 1+
405.2262 1+
523.3049 1+
m/z

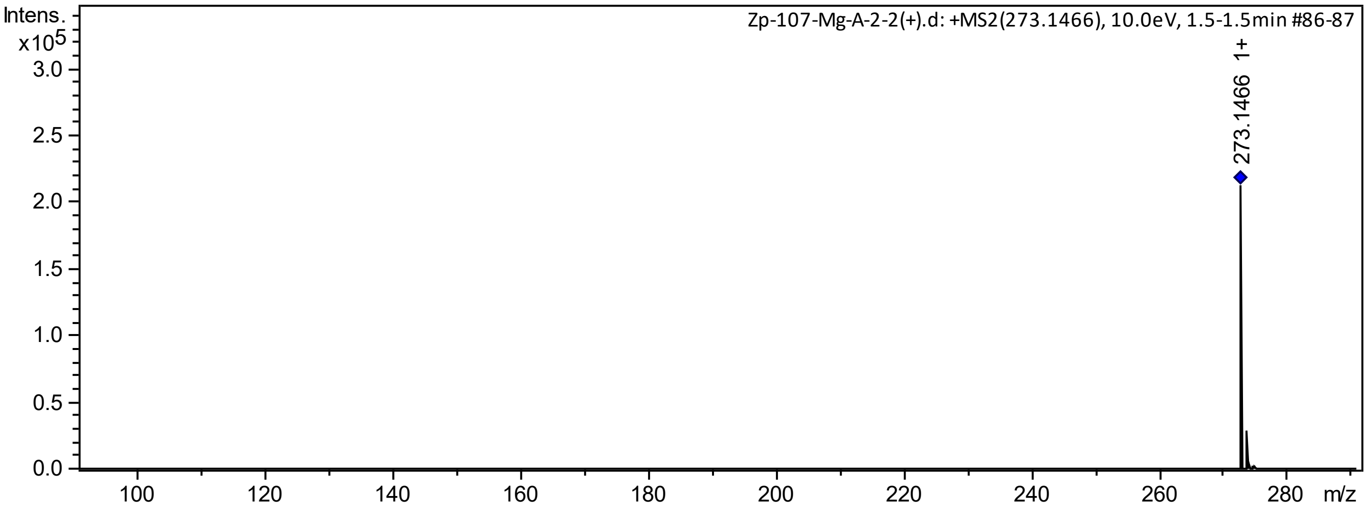
Intens.
x10^5
Zp-107-Mg-A-2-2(+).d: +MS2(273.1466), 10.0eV, 1.5-1.5min #86-87
273.1466 1+
m/z

Figure S14. ^{1}H NMR spectrum of **2** measured at 700 MHz in $CDCl_3$

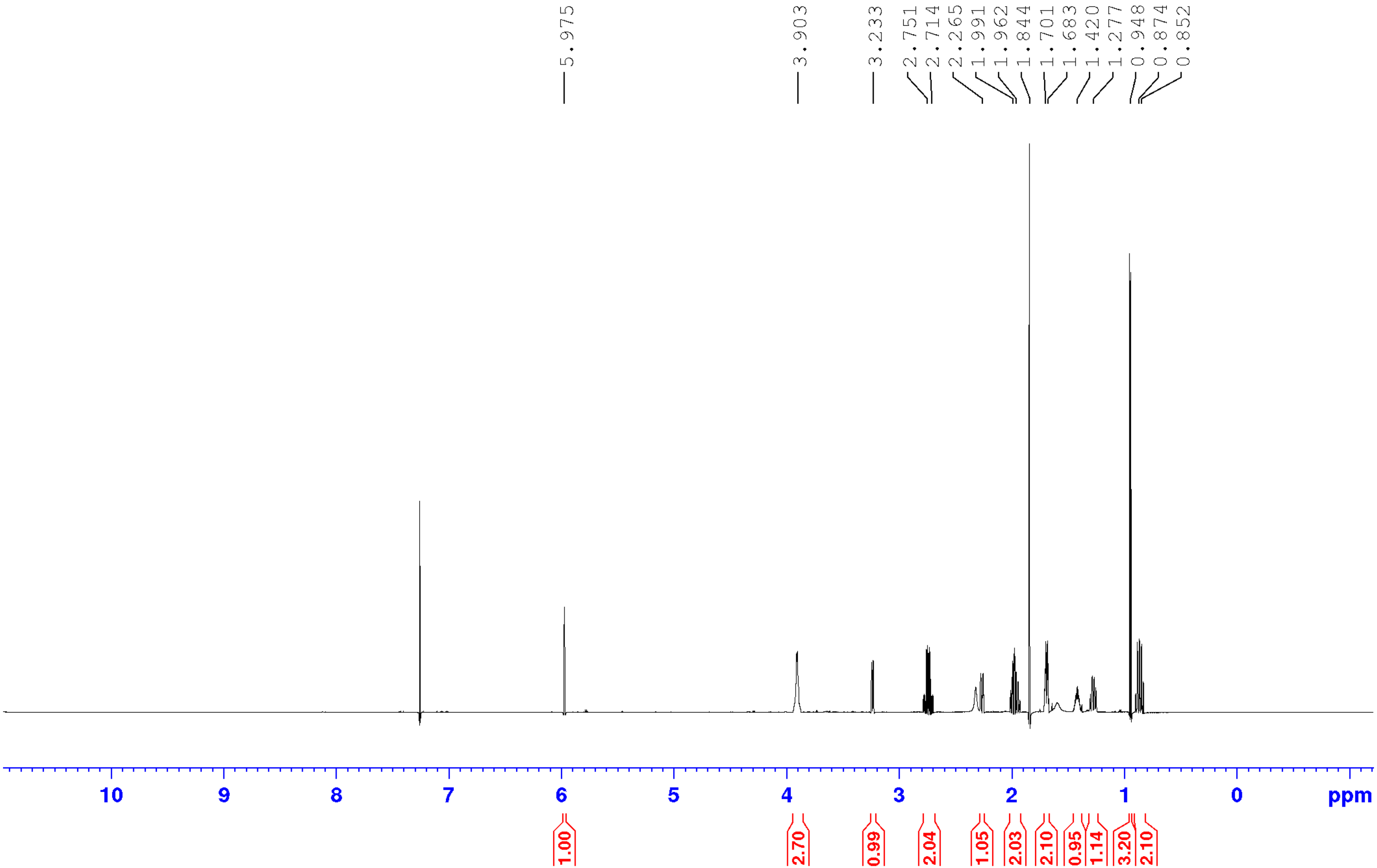

Figure S15. ^{13}C NMR spectrum of **2** measured at 176 MHz in $CDCl_3$

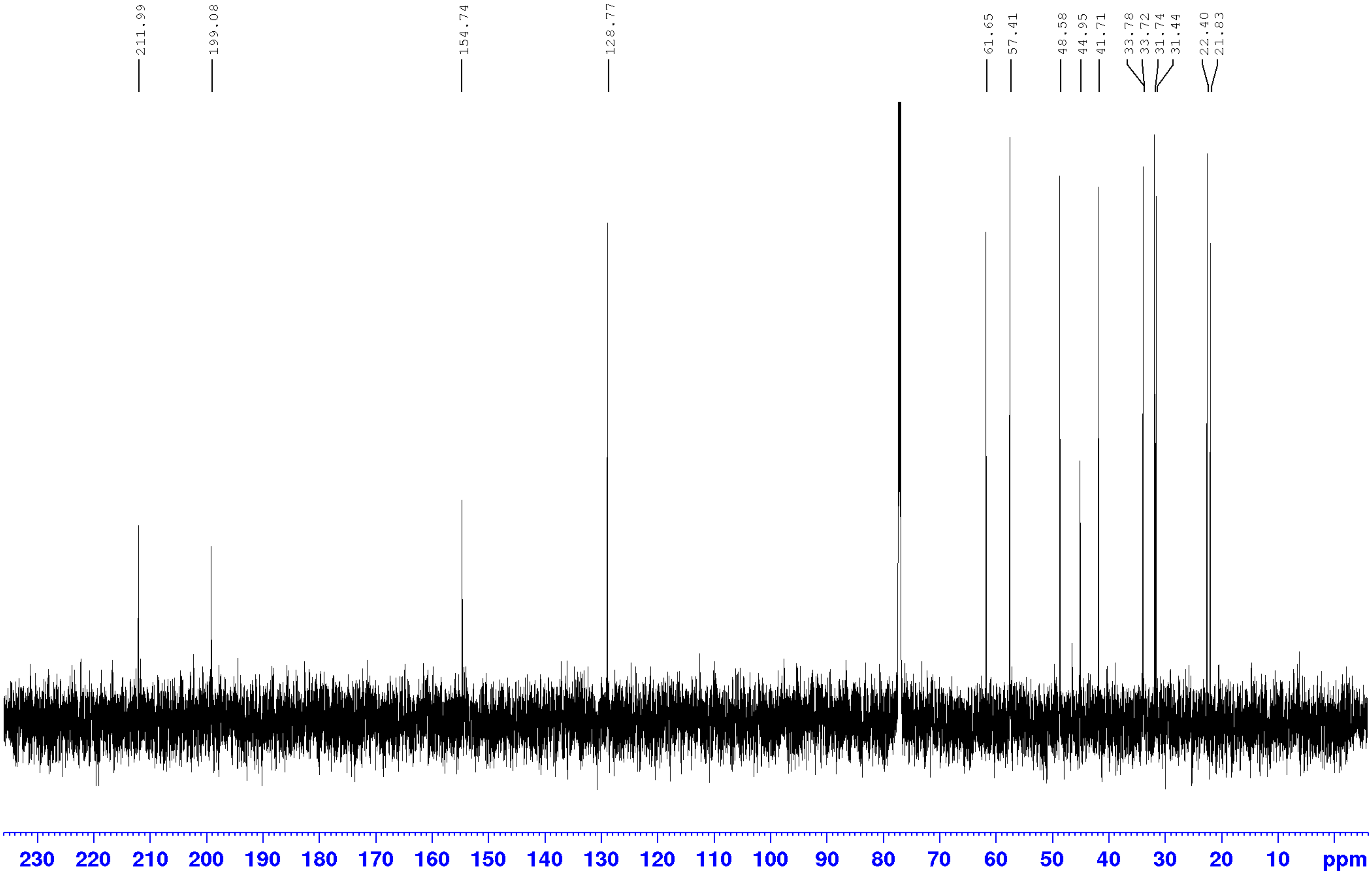

Figure S16. DEPT-135 spectrum of **2** measured at 176 MHz in $CDCl_3$

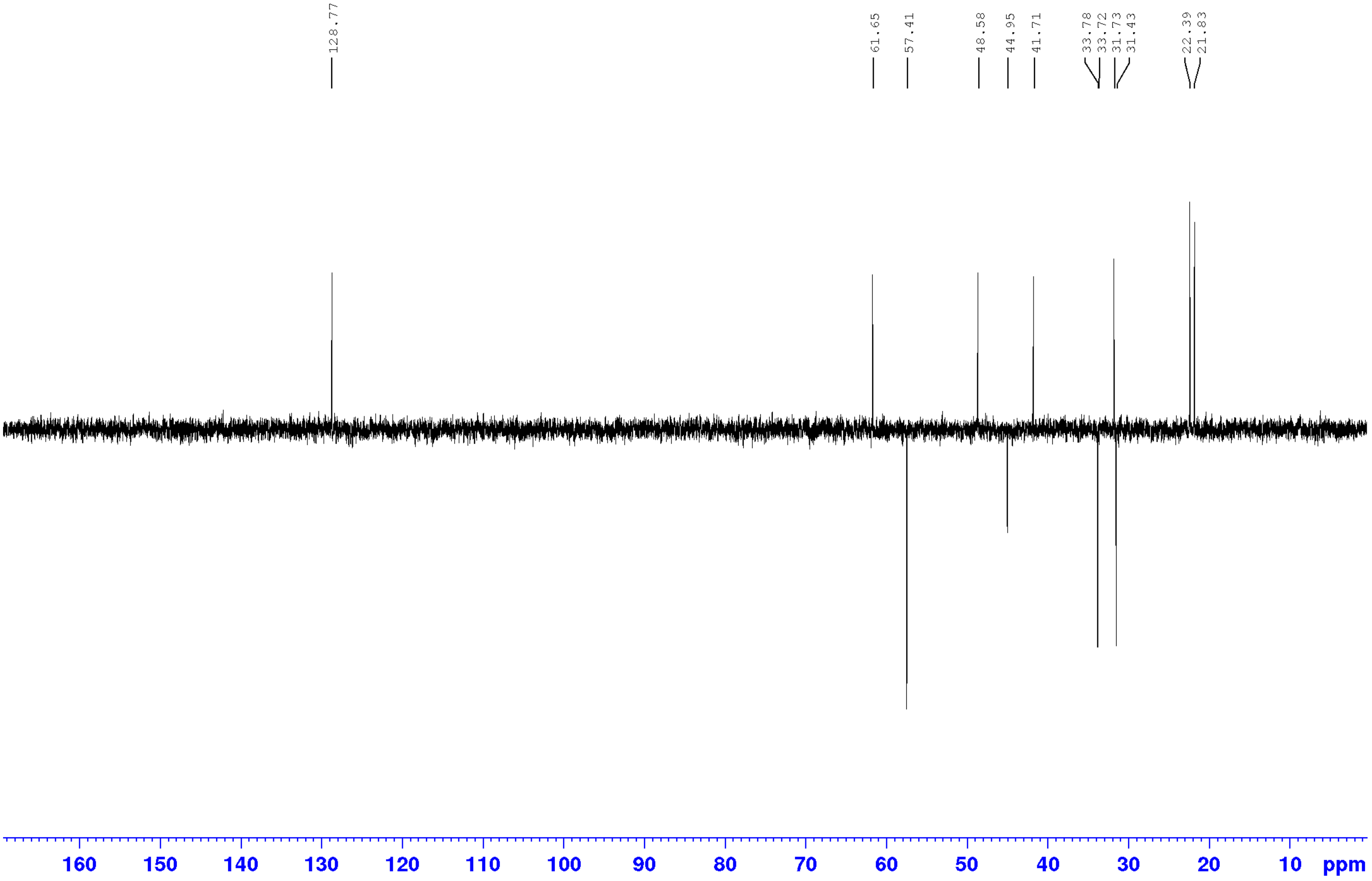

Figure S17. HSQC spectrum of **2** measured in $CDCl_3$

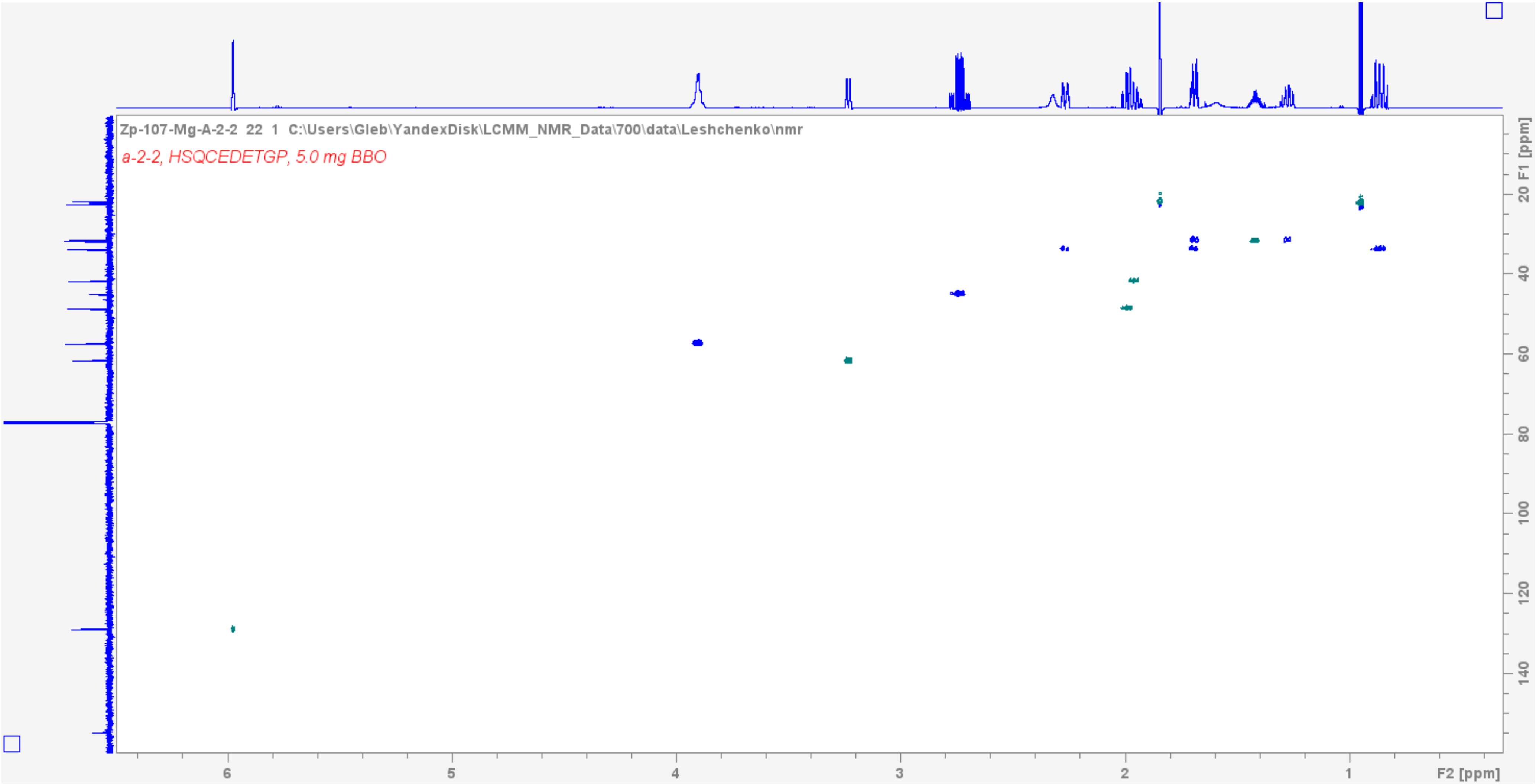

Figure S18. COSY spectrum of **2** measured in $CDCl_3$

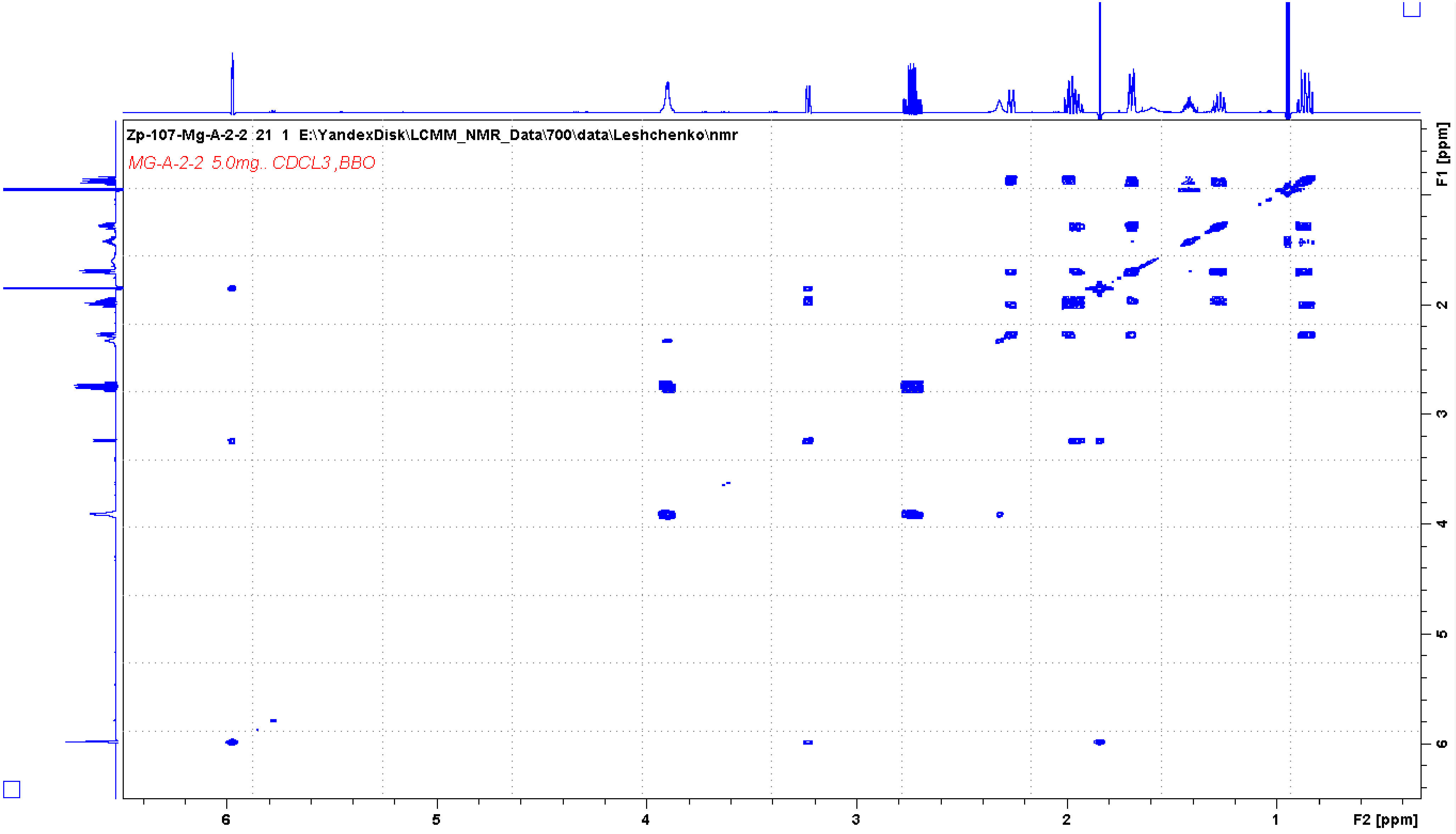

Figure S19. HMBC spectrum of **2** measured in $CDCl_3$

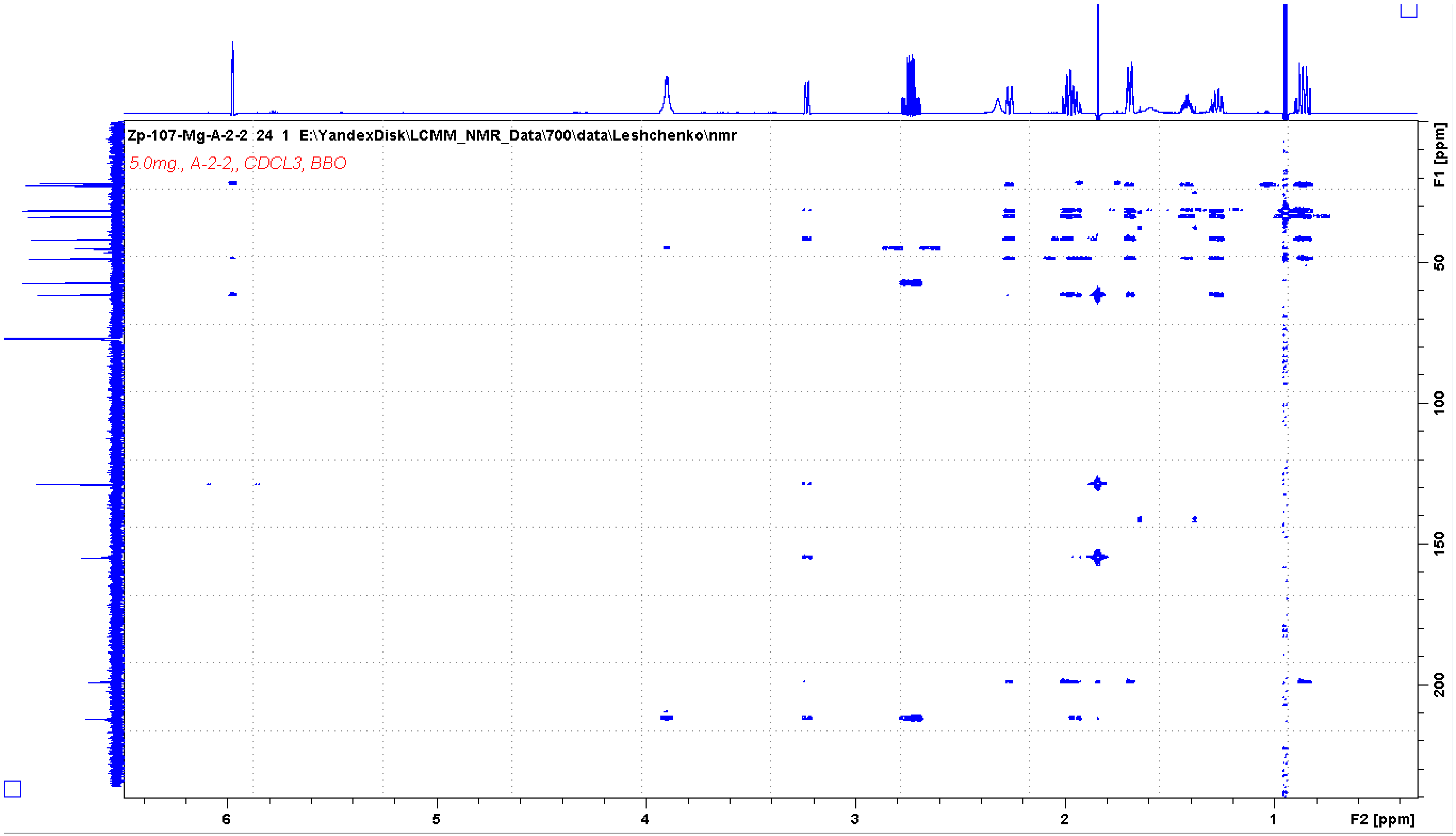

Figure S20. ROESY spectrum of **2** measured in $CDCl_3$

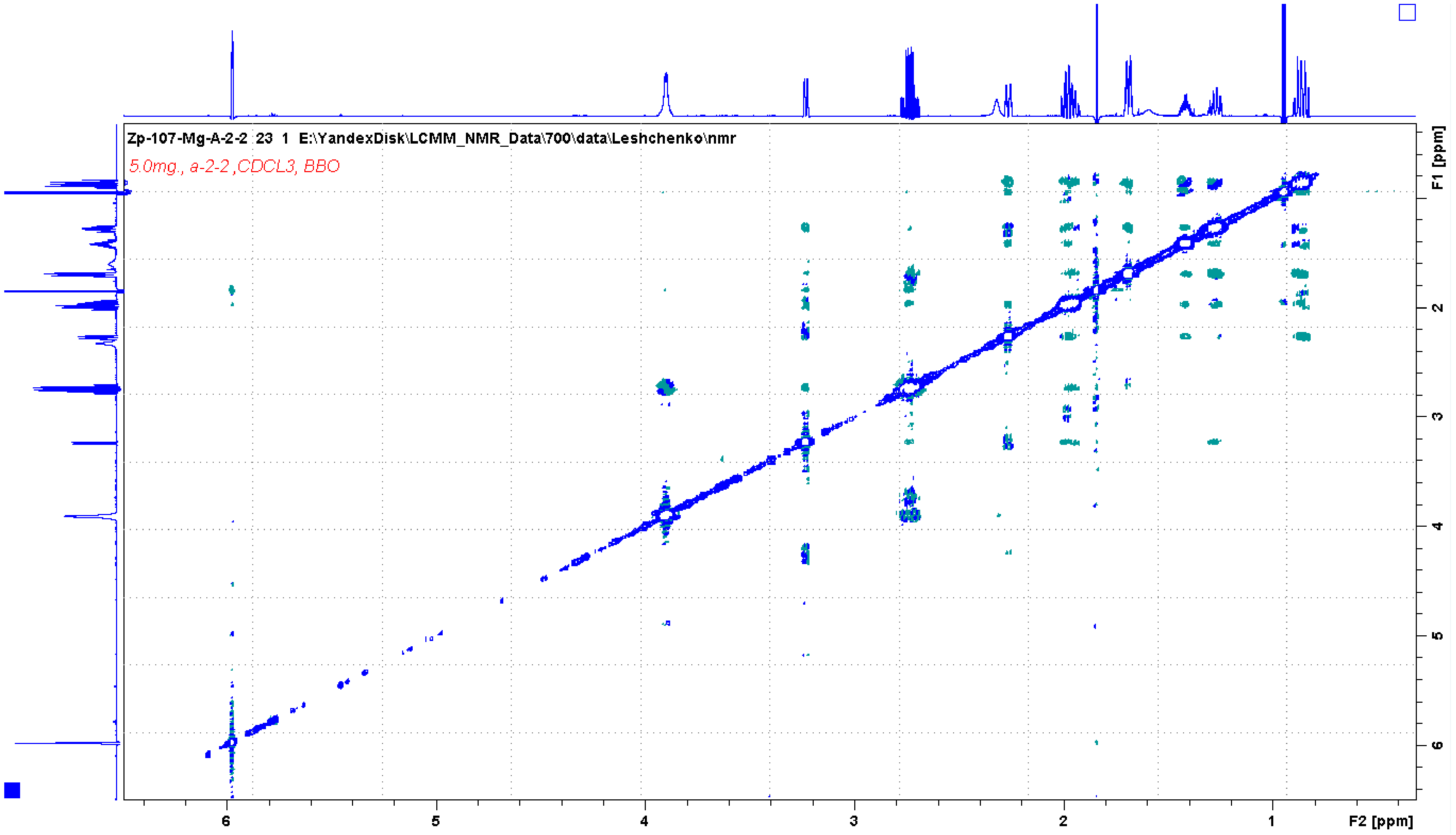

Figure S21. UV spectrum of **2** measured in MeOH

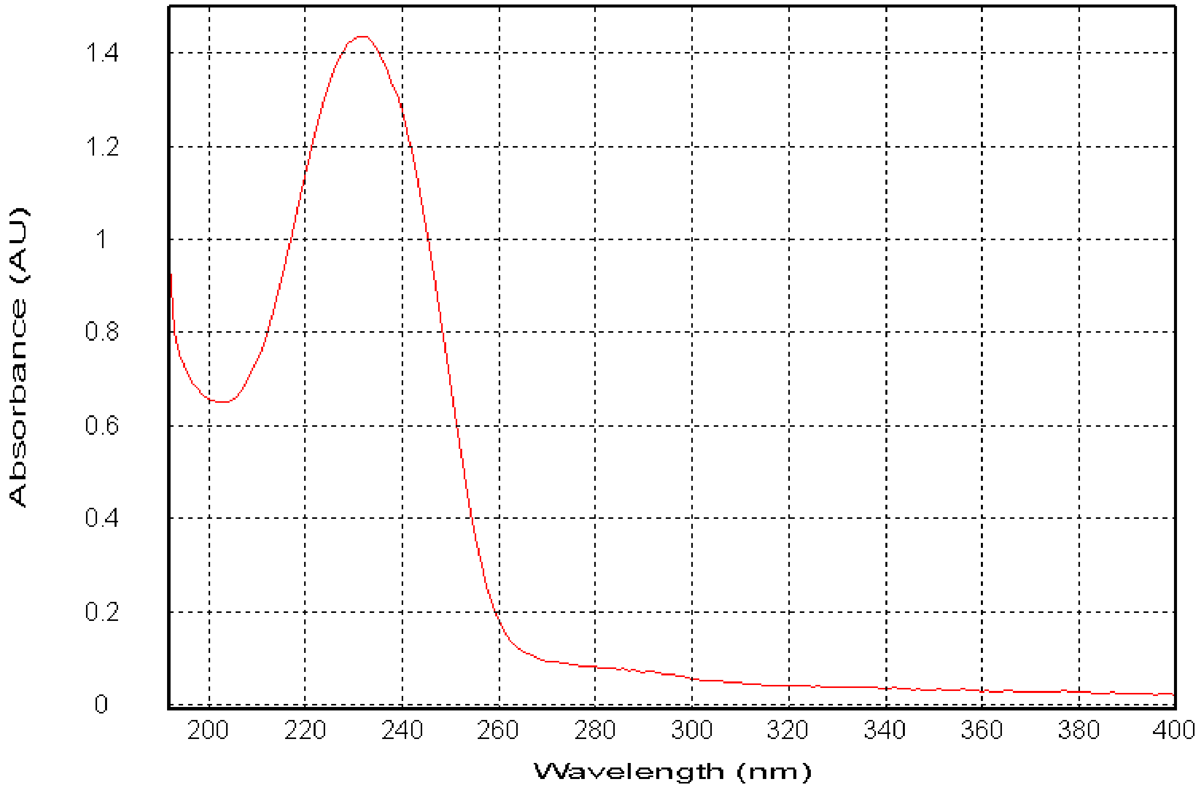

Figure S22. CD spectrum of **2** measured in MeOH

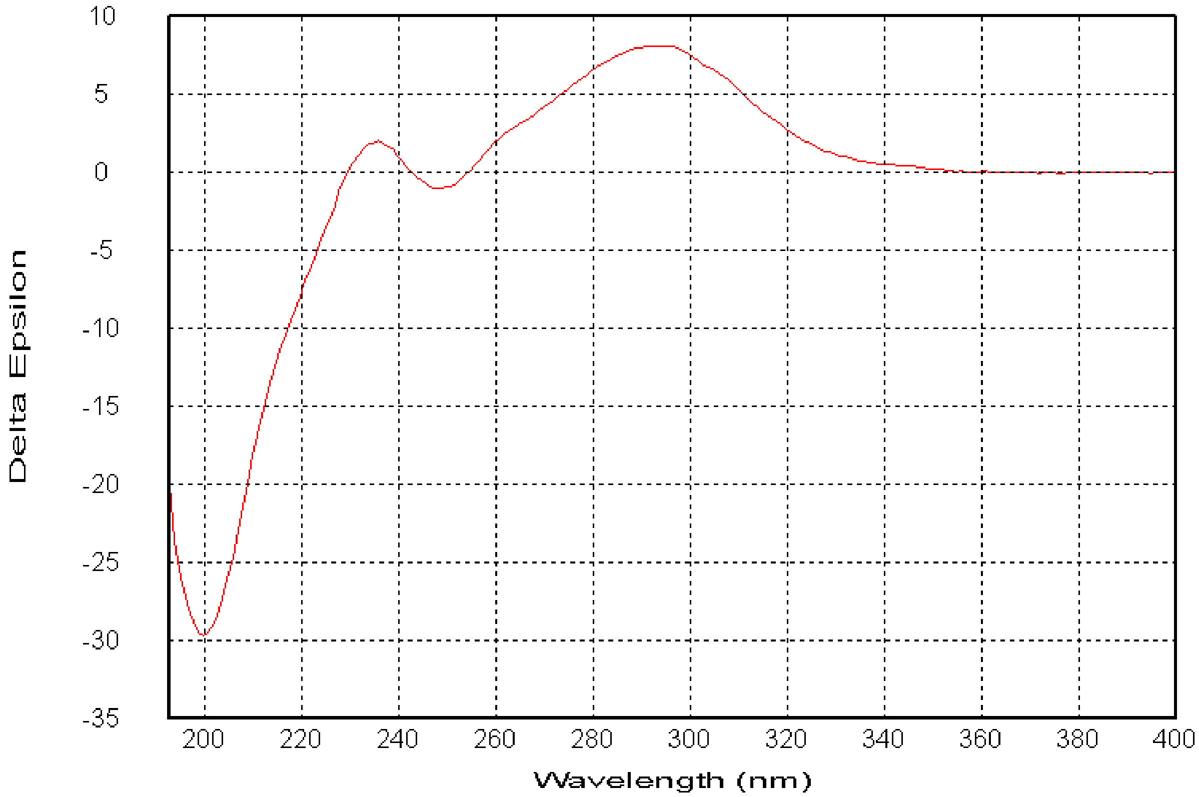

Figure S23. (**A**) Key COSY, (**B**) HMBC and (**C**) ROESY correlations of **3**

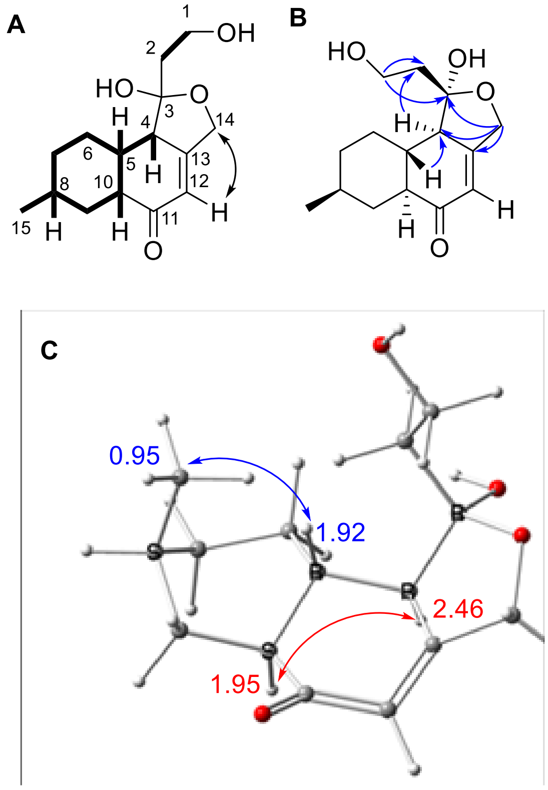

Figure S24. HRESIMS for **3**

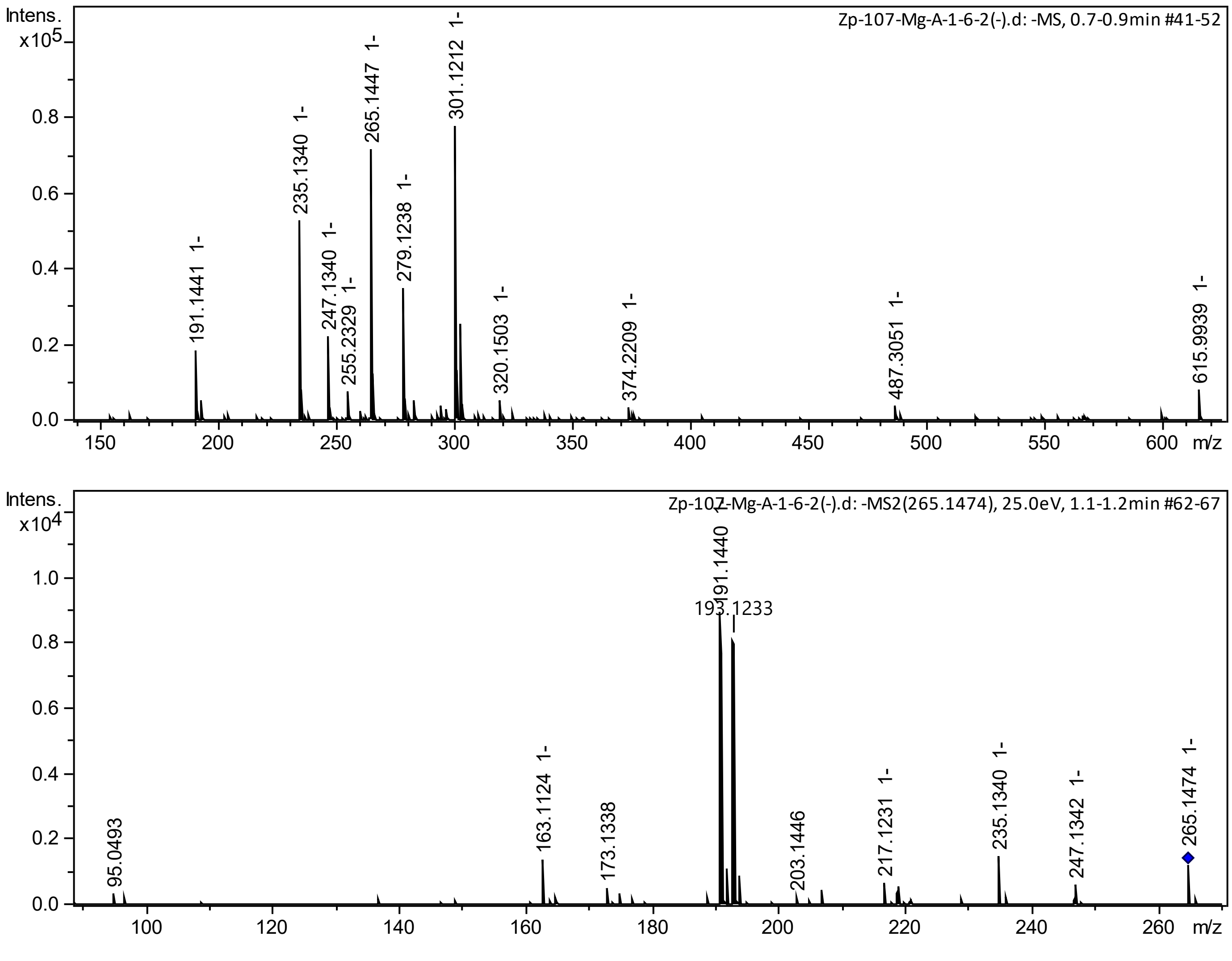

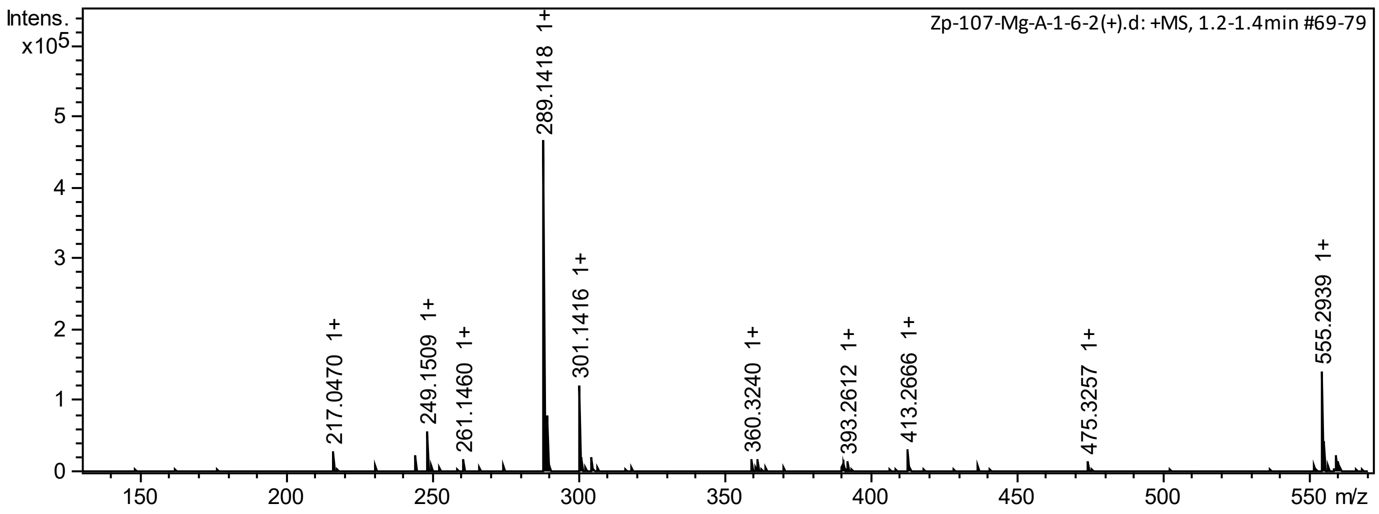

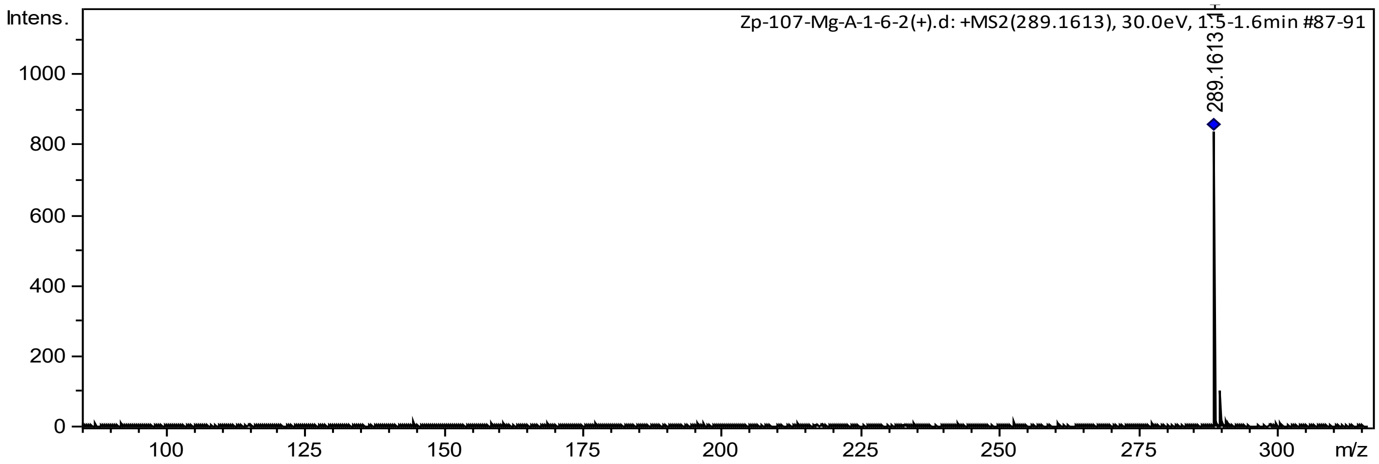

	meas	calc	Δ (ppm)
$[M-H]^-$	265,1447	265,1445	-0,8
$[M+Cl]^-$	301,1212	301,1212	0,0
$[M+H]^+$	267,1590	267,1591	0,4
$[M+Na]^+$	289,1418	289,141	-2,8

Figure S25. ^{1}H NMR spectrum of **3** measured at 700 MHz in $CDCl_3$

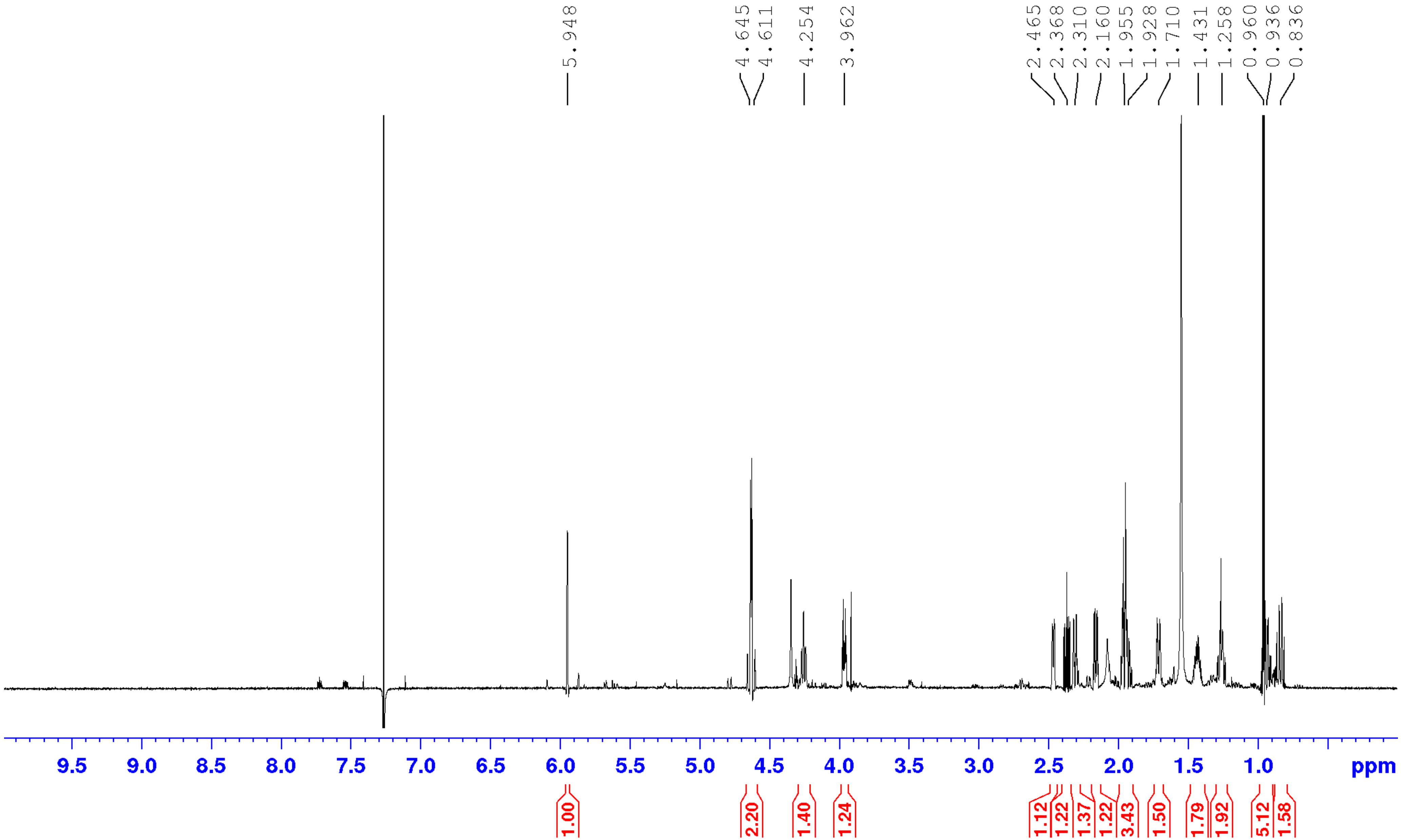

Figure S26. ^{13}C NMR spectrum of **3** measured at 176 MHz in $CDCl_3$

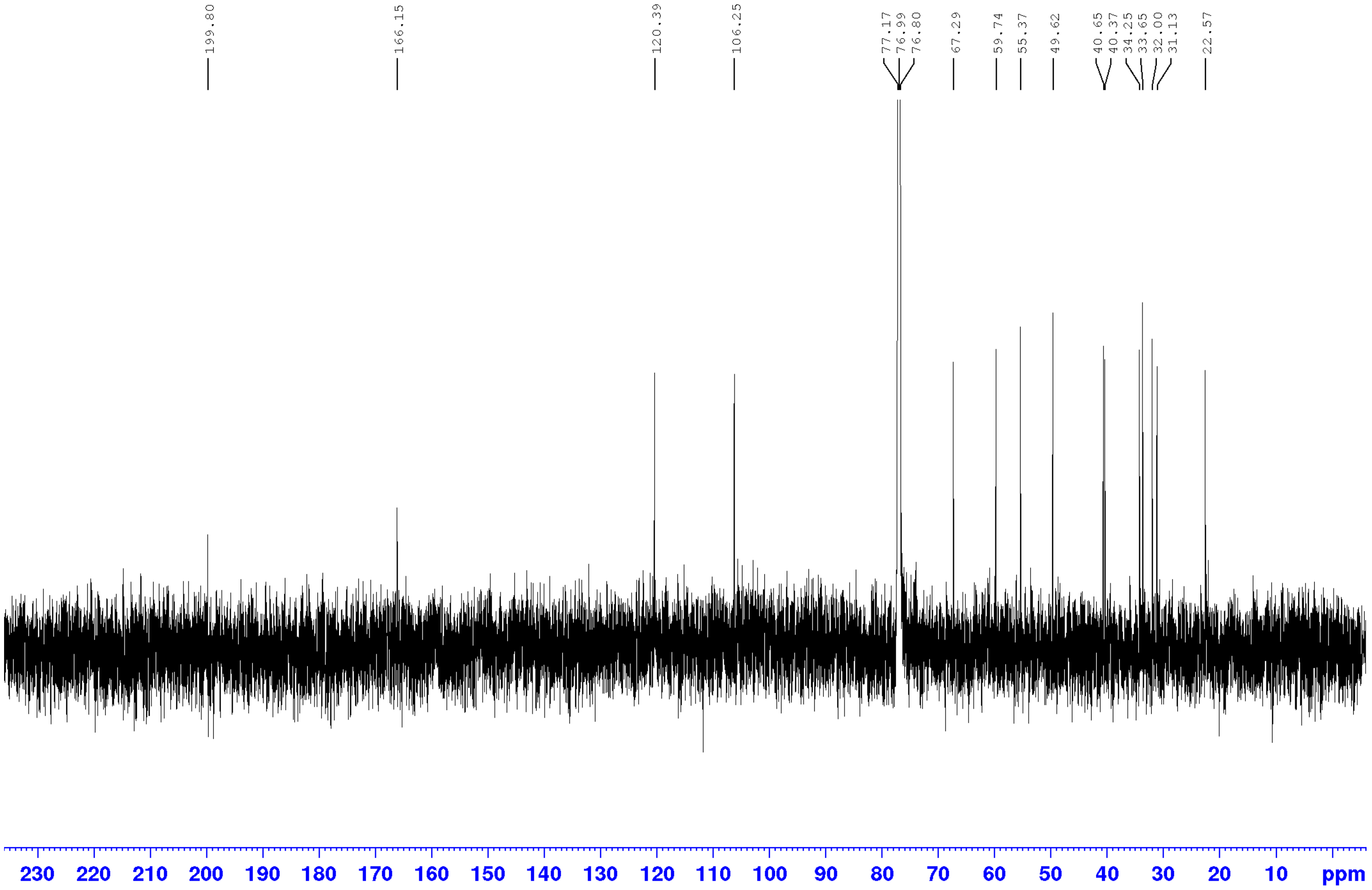

Figure S27. DEPT-135 spectrum of **3** measured at 176 MHz in $CDCl_3$

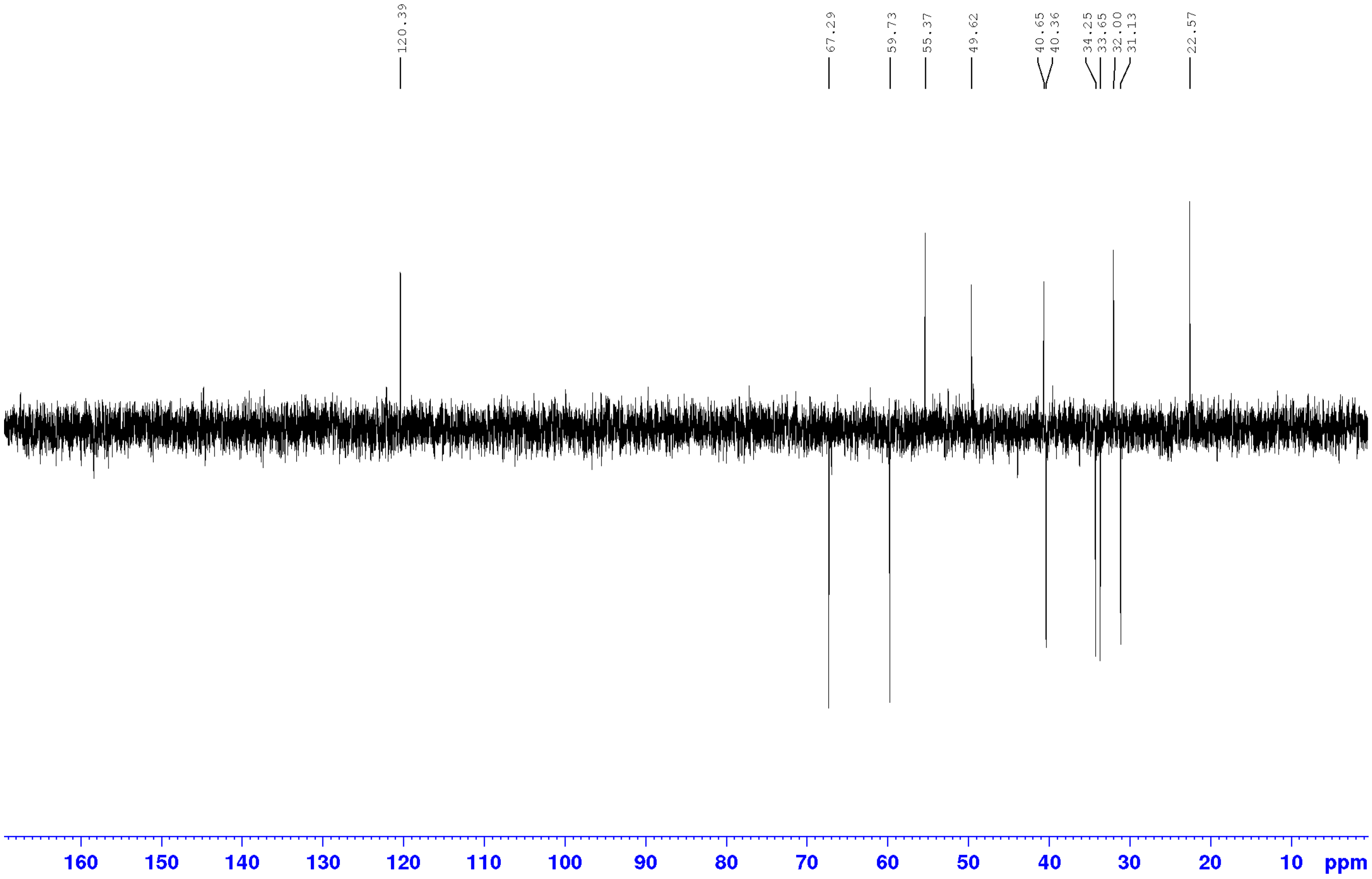

Figure S28. HSQC spectrum of **3** measured in $CDCl_3$

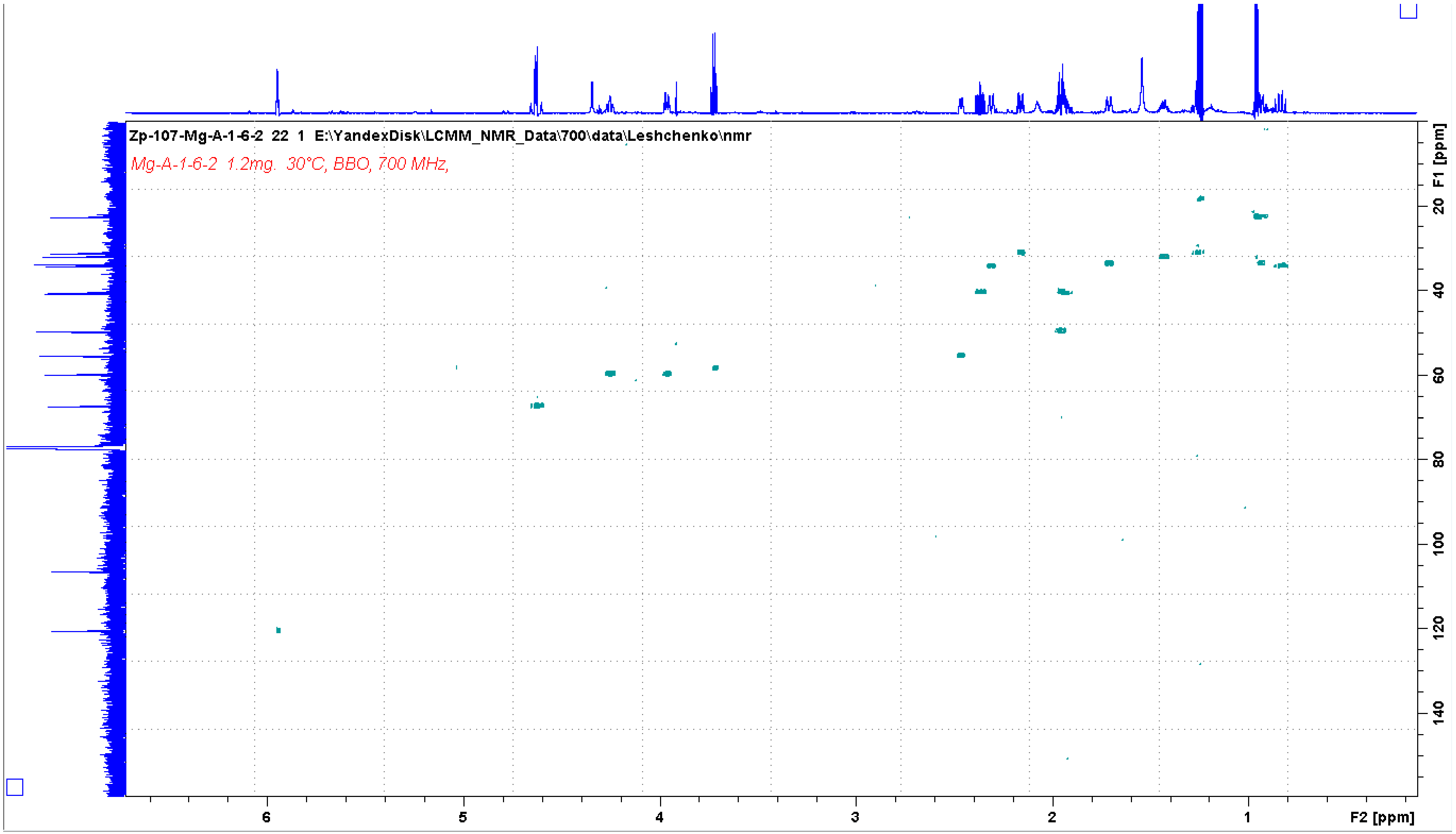

Figure S29. COSY spectrum of **3** measured in $CDCl_3$

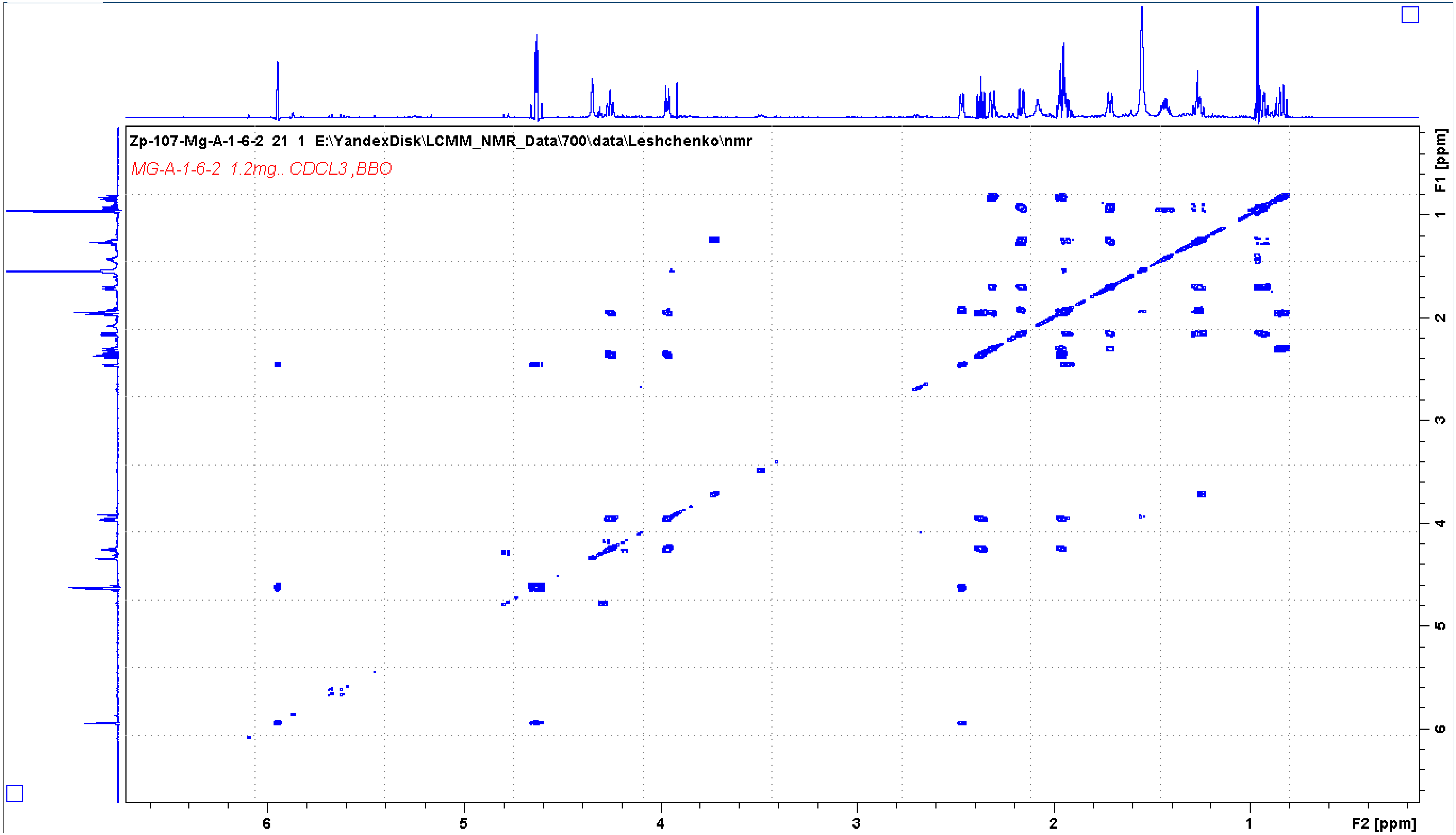

Figure S30. HMBC spectrum of **3** measured in $CDCl_3$

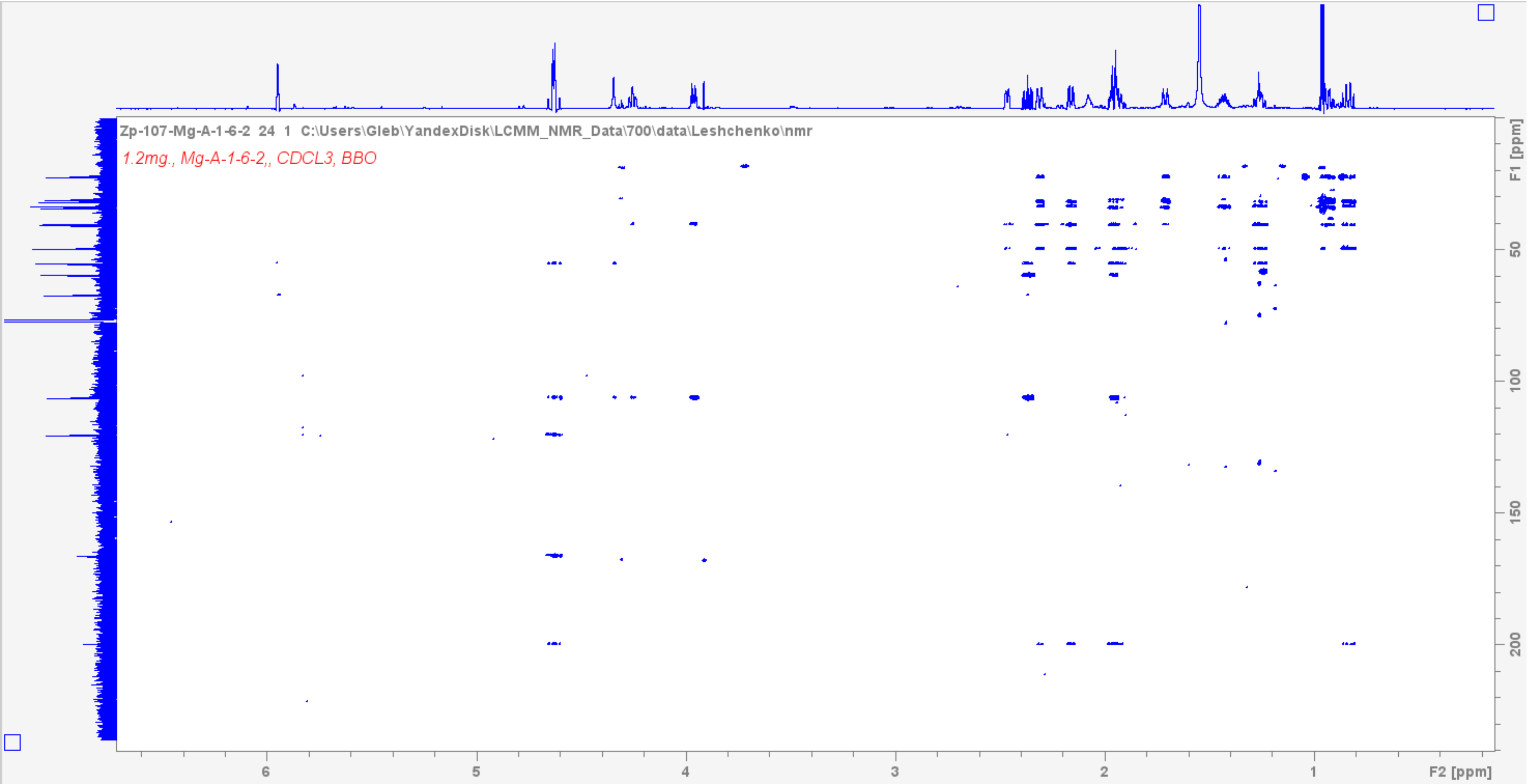

Figure S31. ROESY spectrum of **3** measured in $CDCl_3$

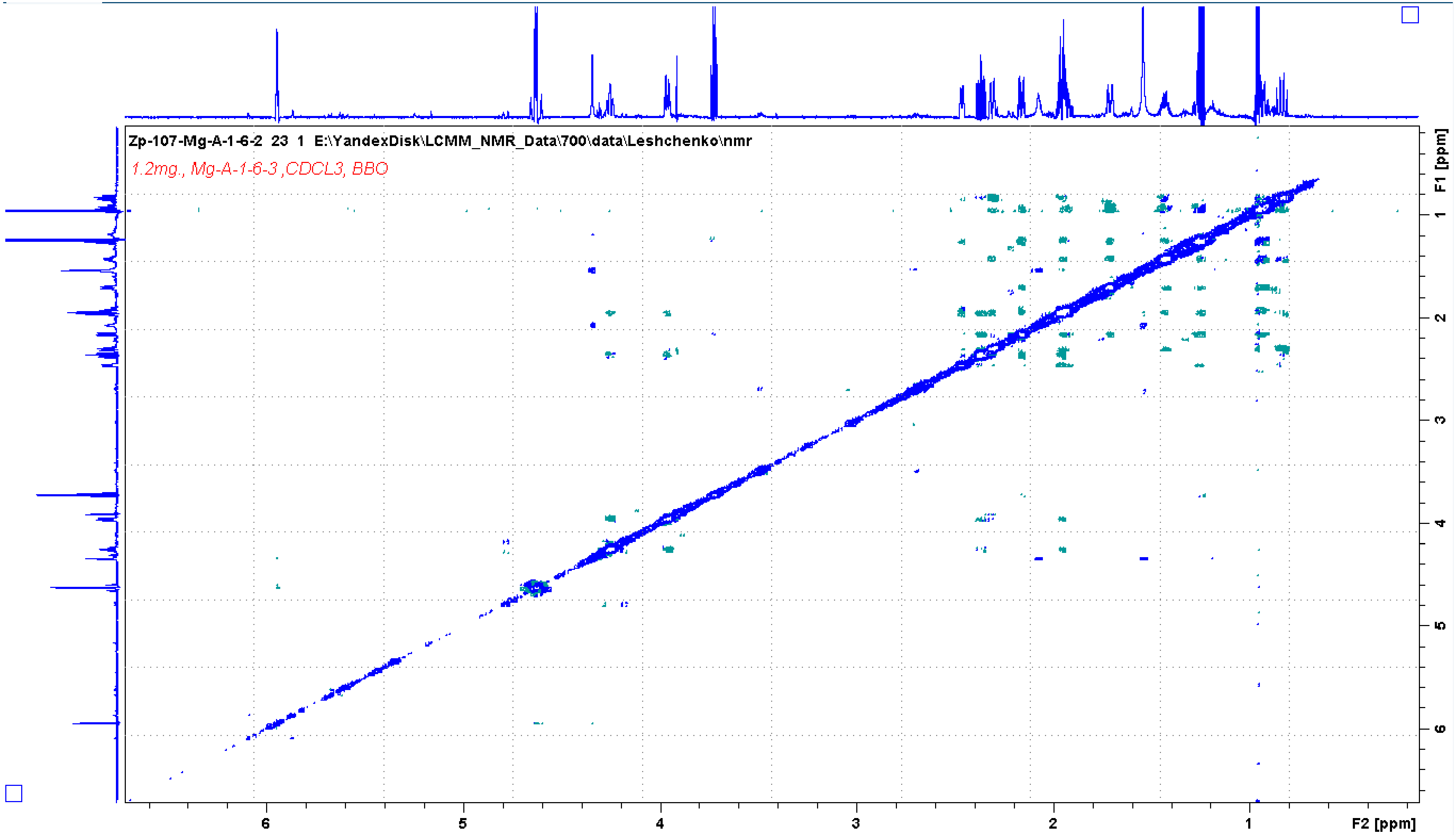

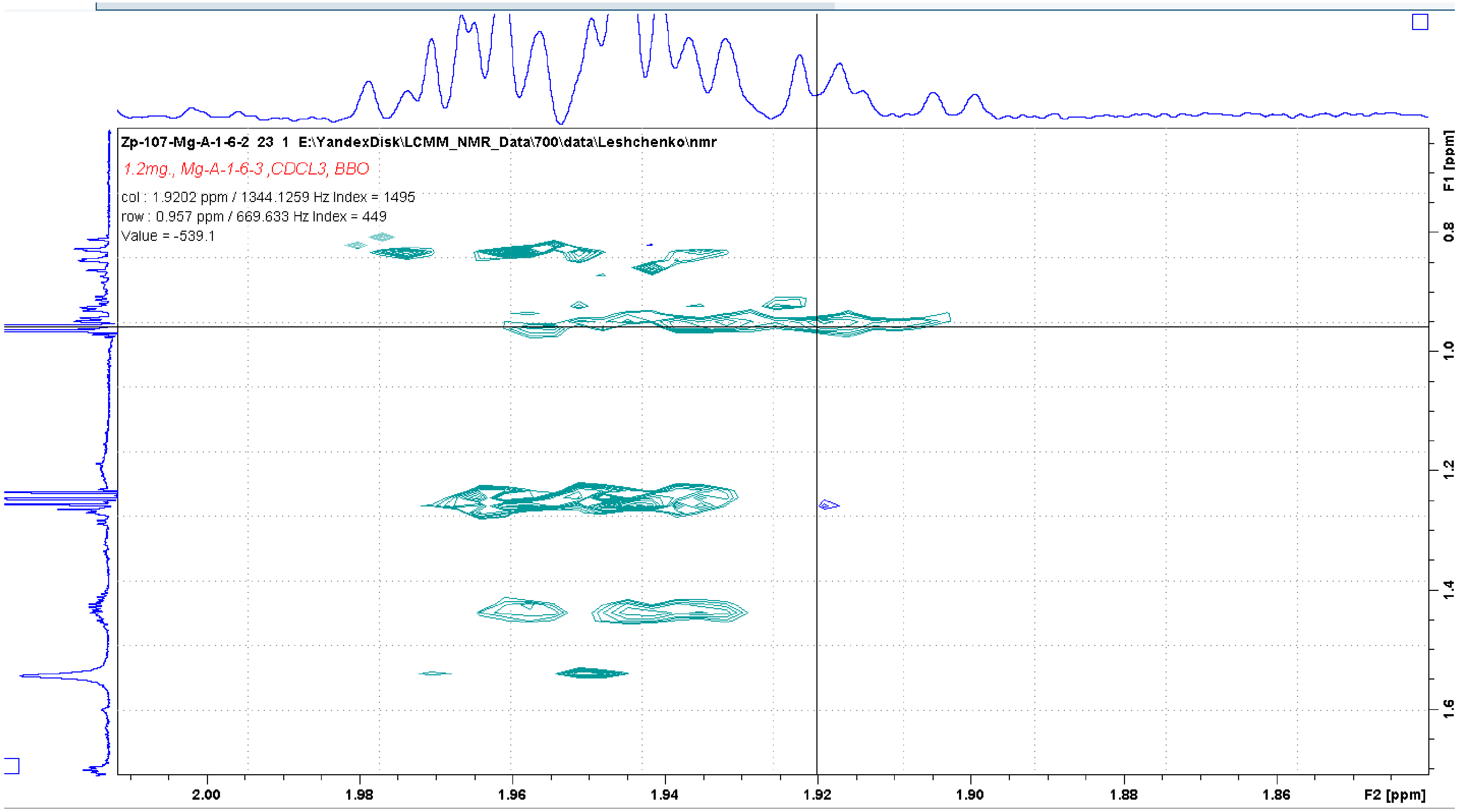
Zp-107-Mg-A-1-6-2 23 1 E:\YandexDisk\LCMM_NMR_Data\700\data\Leshchenko\nmr
1.2mg., Mg-A-1-6-3 ,CDCL3, BBO
col : 1.9202 ppm / 1344.1259 Hz Index = 1495
row : 0.957 ppm / 669.633 Hz Index = 449
Value = -539.1
F1 [ppm]
0.8
1.0
1.2
1.4
1.6
2.00
1.98
1.96
1.94
1.92
1.90
1.88
1.86
F2 [ppm]

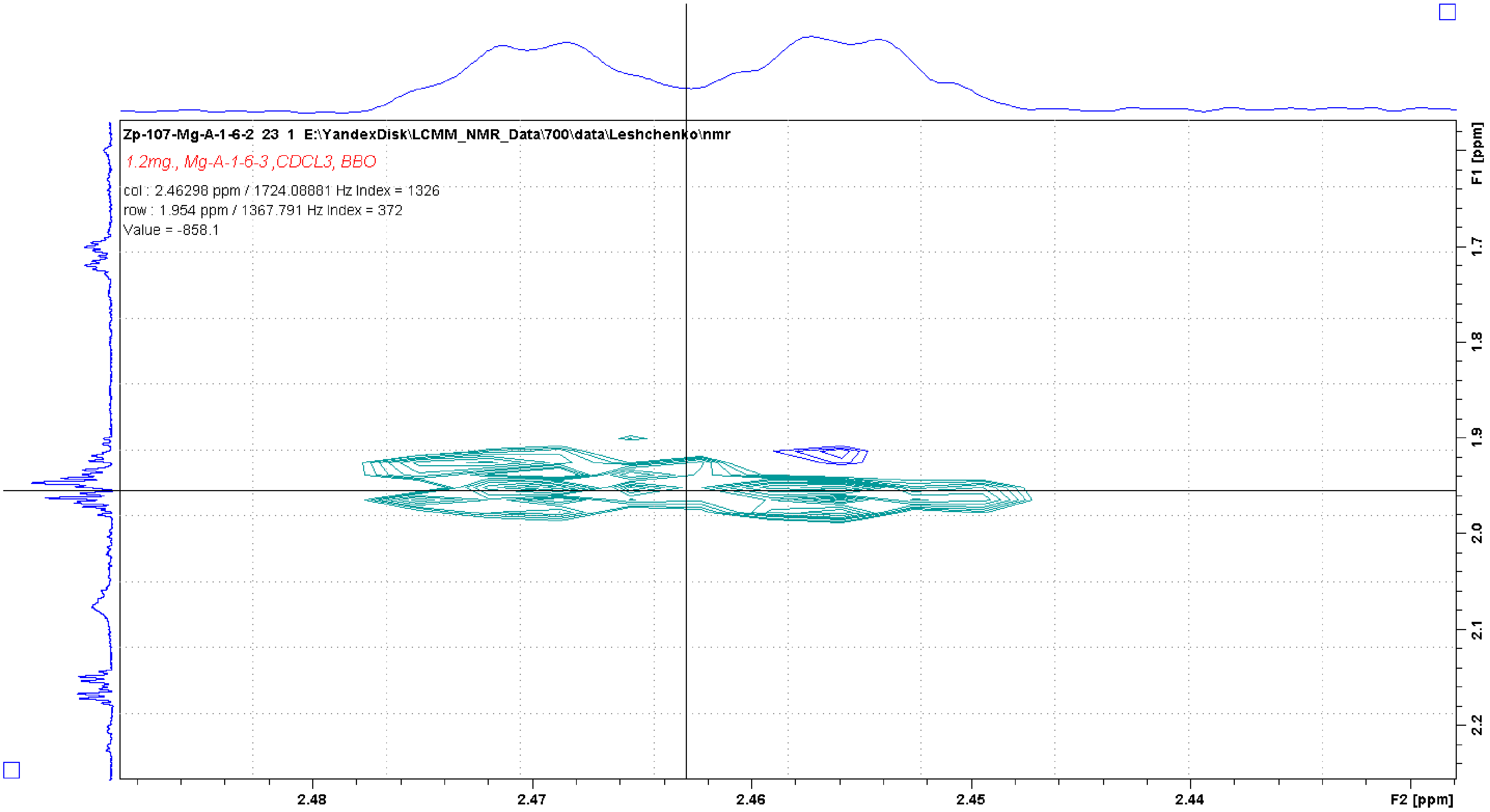

Zp-107-Mg-A-1-6-2 23 1 E:\YandexDisk\LCMM_NMR_Data\700\data\Leshchenko\nmr
1.2mg., Mg-A-1-6-3 ,CDCL3, BBO
col : 2.46298 ppm / 1724.08881 Hz Index = 1326
row : 1.954 ppm / 1367.791 Hz Index = 372
Value = -858.1
F1 [ppm]
1.7
1.8
1.9
2.0
2.1
2.2
2.48
2.47
2.46
2.45
2.44
F2 [ppm]

Figure S32. UV spectrum of **3** measured in MeOH

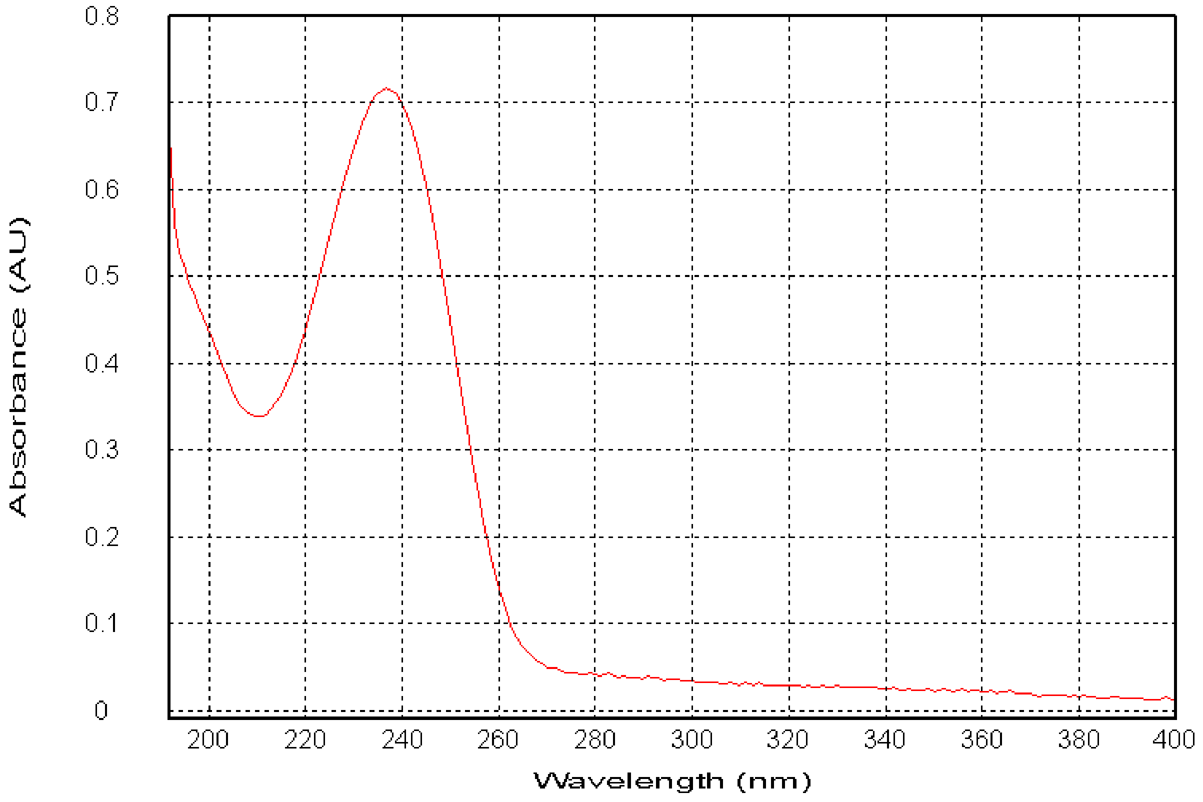

Figure S33. CD spectrum of **3** measured in MeOH

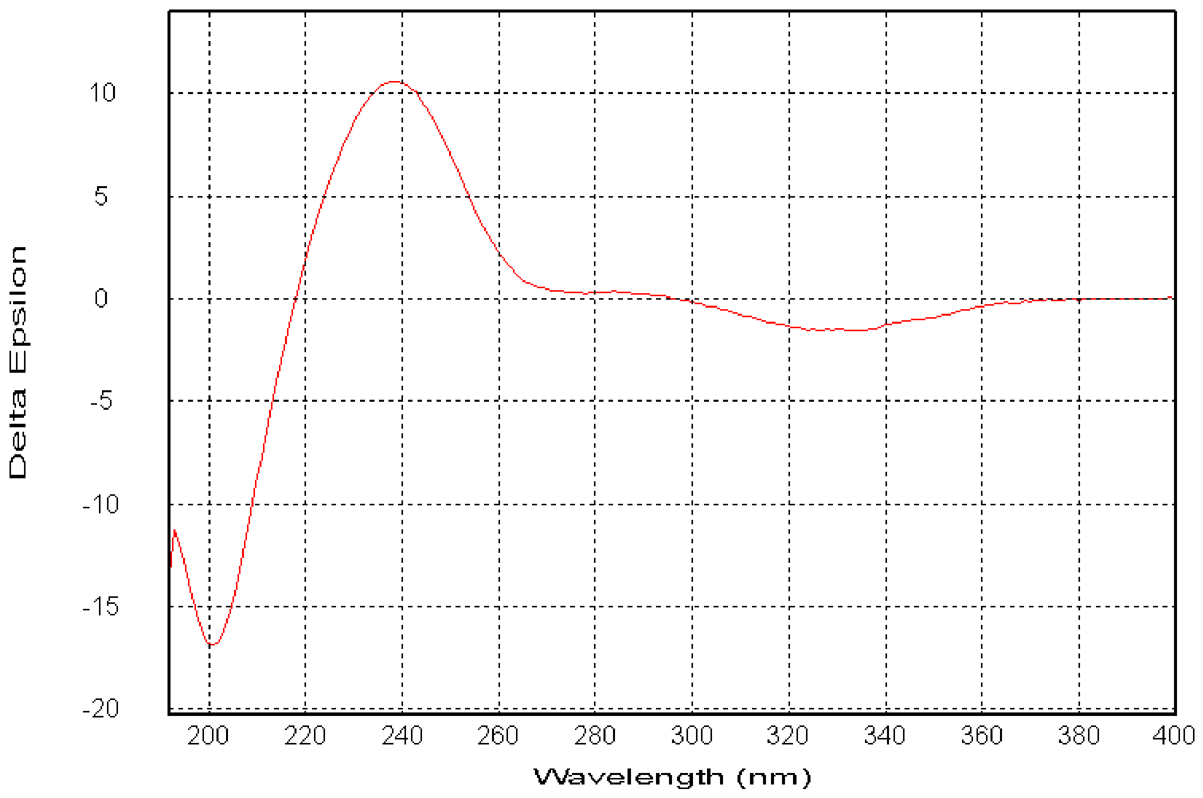

Figure S34. (**A**) Key COSY, (**B**) HMBC and (**C**) ROESY correlations of **4**

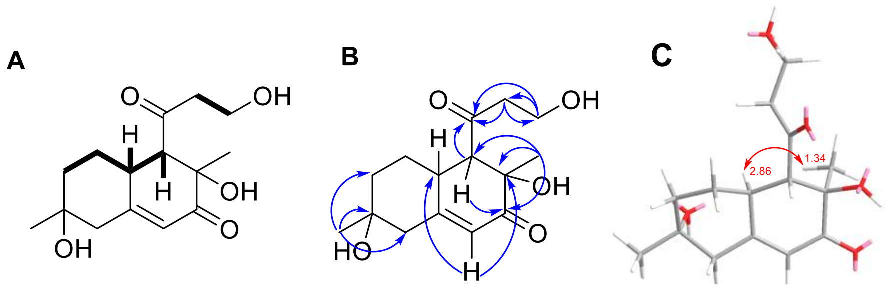

Figure S35. HRESIMS for **4**

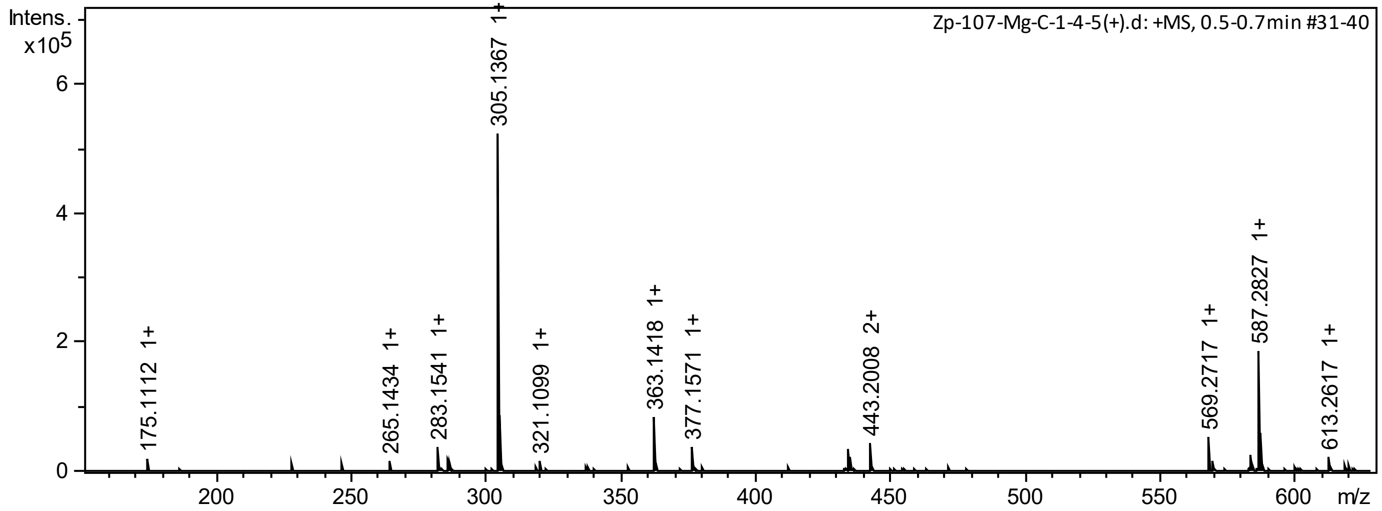

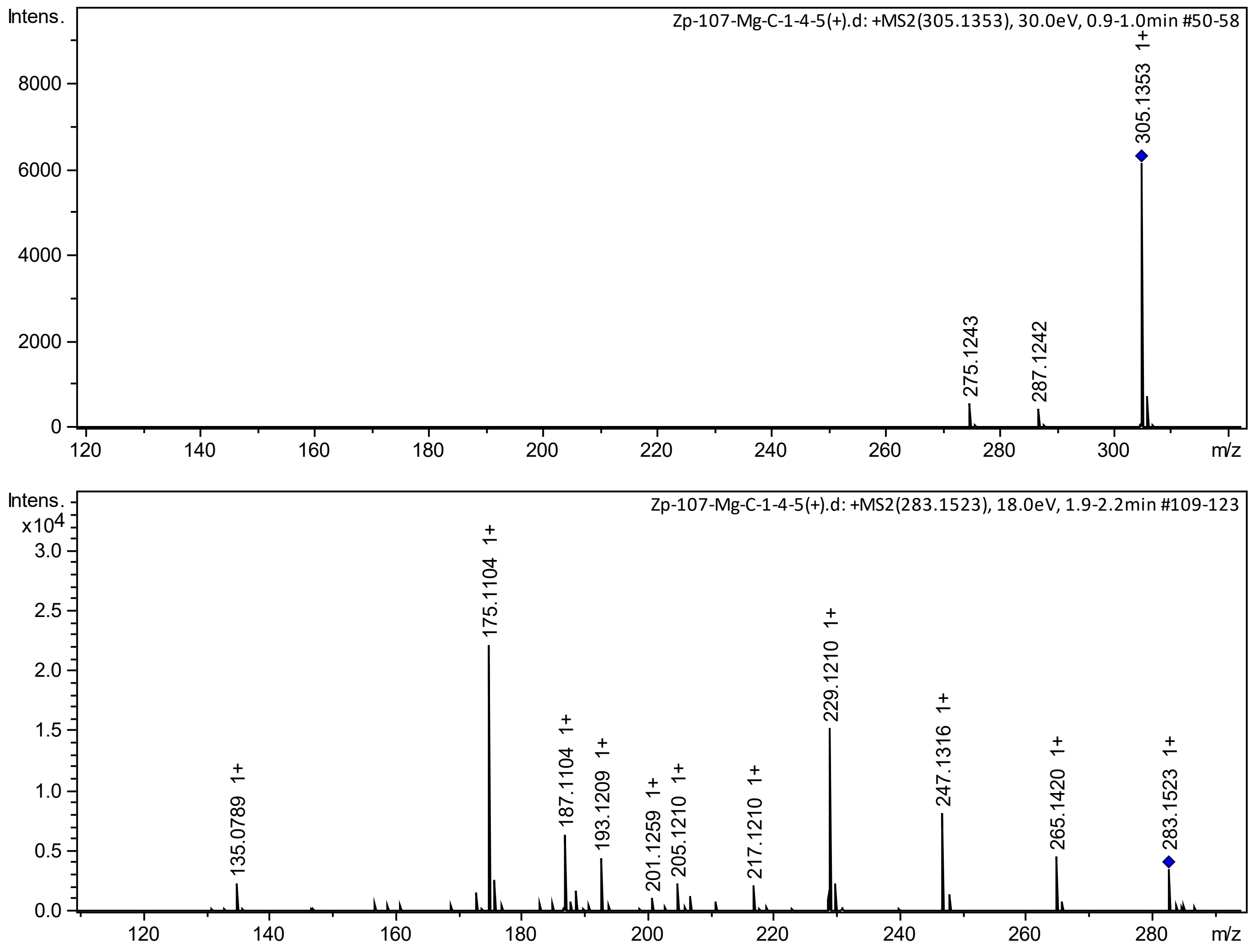
Zp-107-Mg-C-1-4-5(+).d: +MS2(305.1353), 30.0eV, 0.9-1.0min #50-58
Intens.
275.1243
287.1242
305.1353 1+
m/z
Zp-107-Mg-C-1-4-5(+).d: +MS2(283.1523), 18.0eV, 1.9-2.2min #109-123
Intens.
x10^4
135.0789 1+
175.1104 1+
187.1104 1+
193.1209 1+
201.1259 1+
205.1210 1+
217.1210 1+
229.1210 1+
247.1316 1+
265.1420 1+
283.1523 1+
m/z

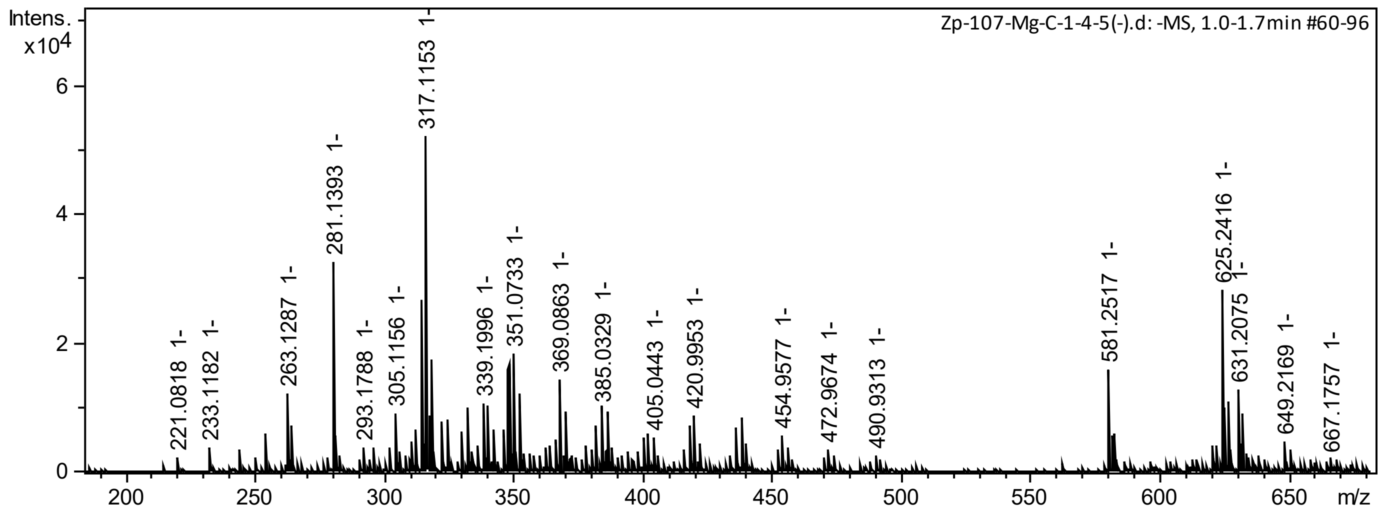

	meas	calc	Δ (ppm)
$[M-H]^-$	281,1393	281,1394	0,4
$[M+H]^+$	283,1541	283,1540	-0,4
$[M+Na]^+$	305,1367	305,1359	-2,6

Figure S36. ^{1}H NMR spectrum of **4** measured at 700 MHz in $CDCl_3$

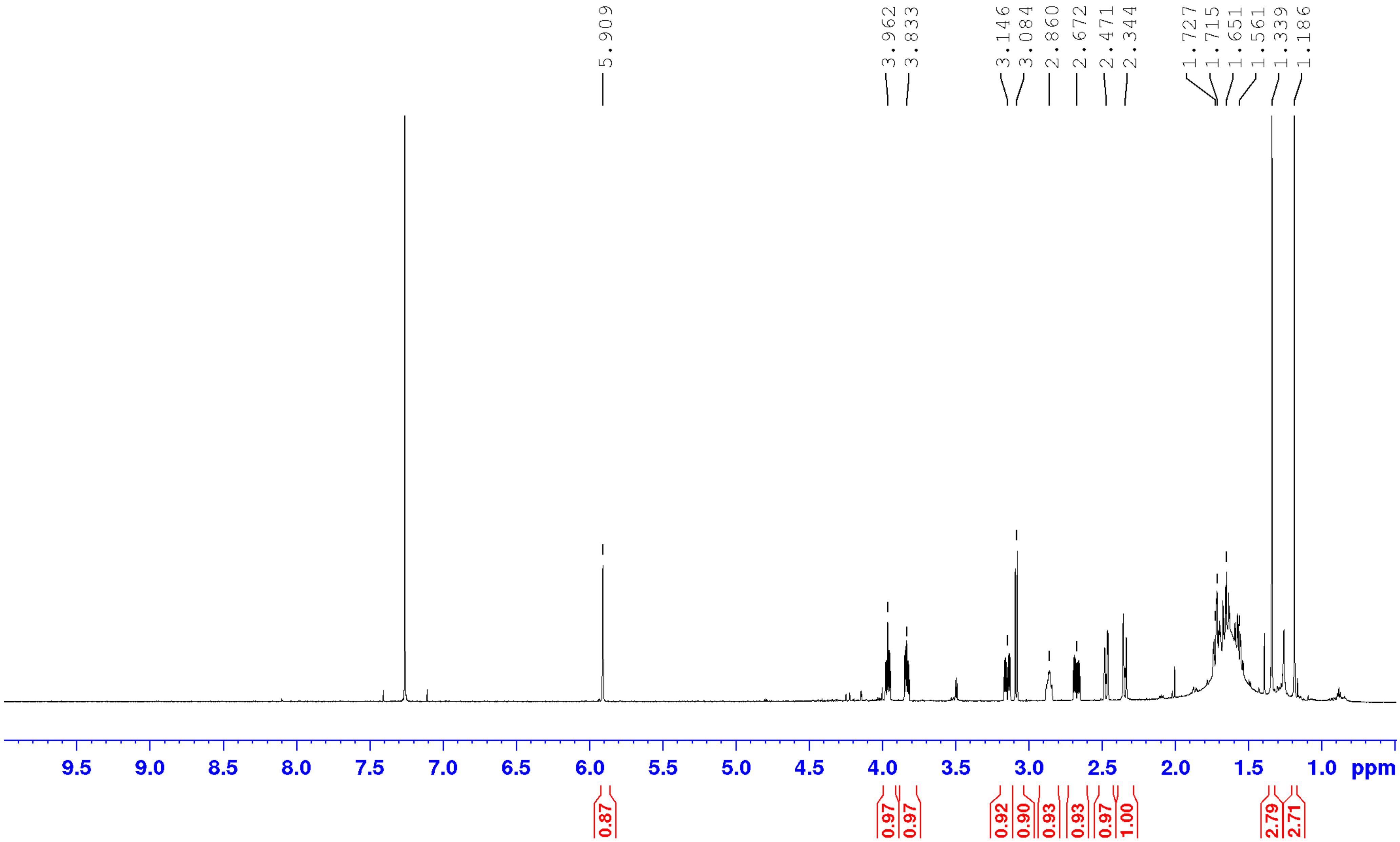

Figure S37. ^{13}C NMR spectrum of **4** measured at 176 MHz in $CDCl_3$

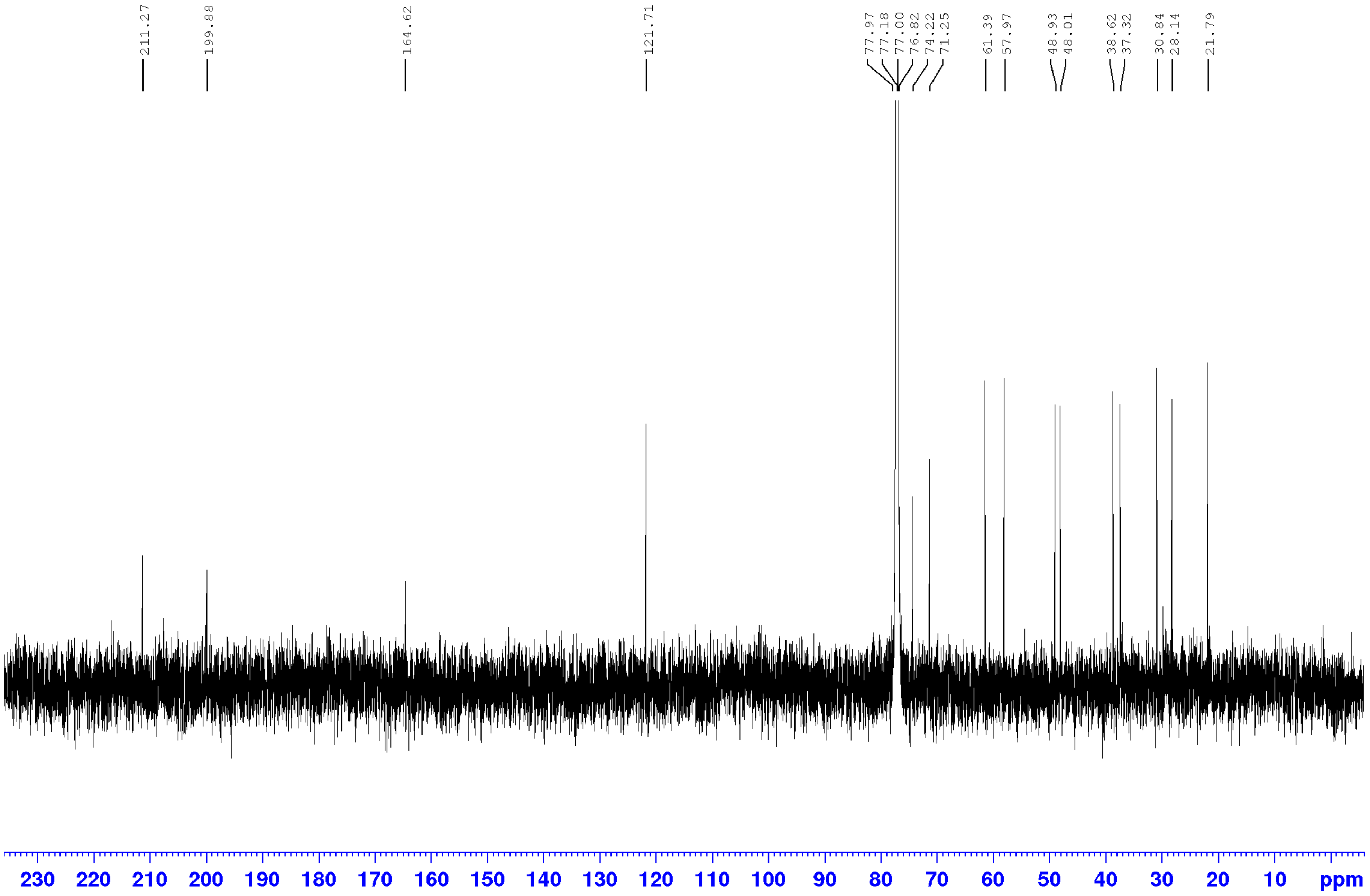

Figure S38. DEPT-135 spectrum of **4** measured at 176 MHz in $CDCl_3$

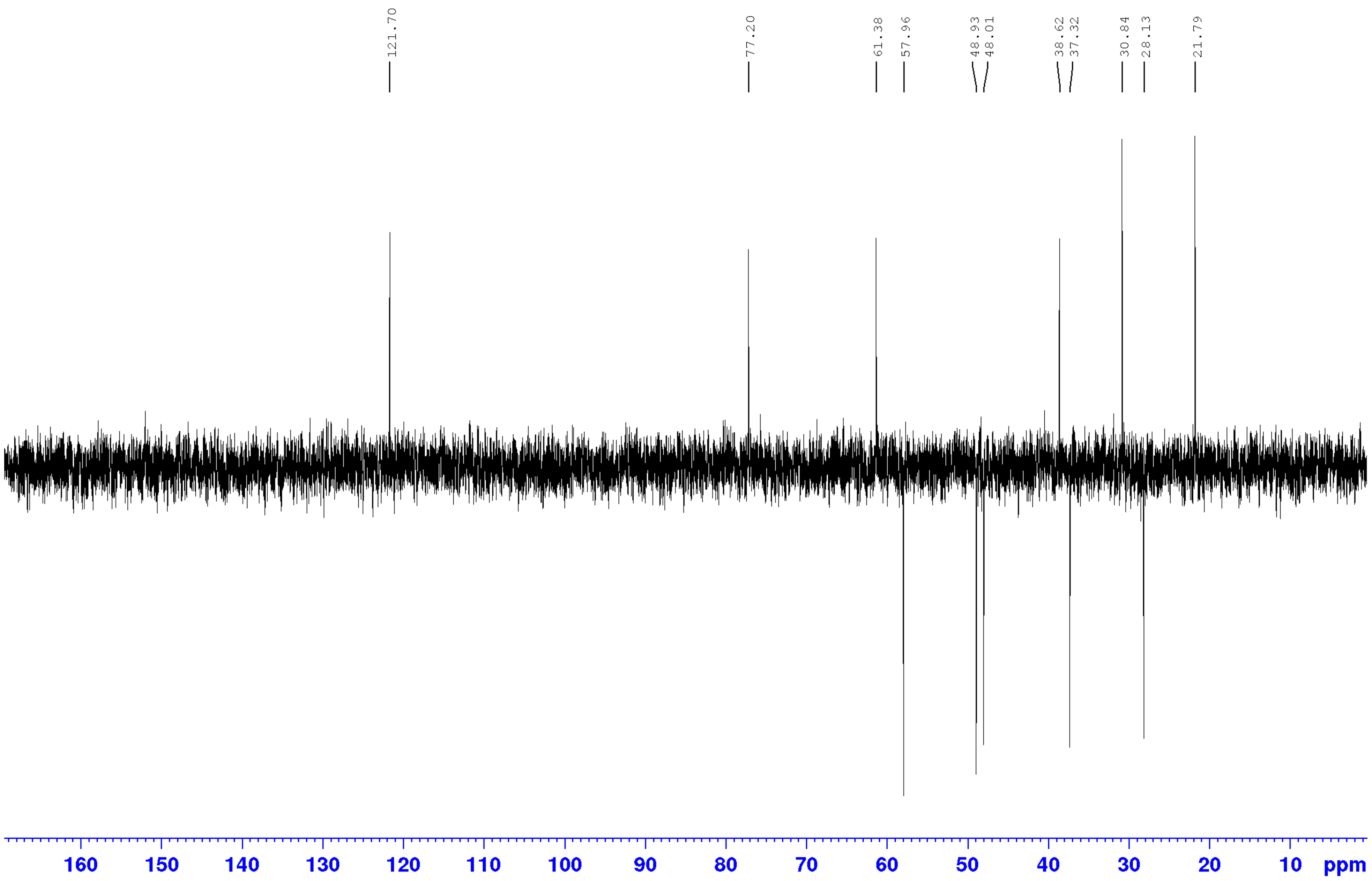

Figure S39. HSQC spectrum of **4** measured in $CDCl_3$

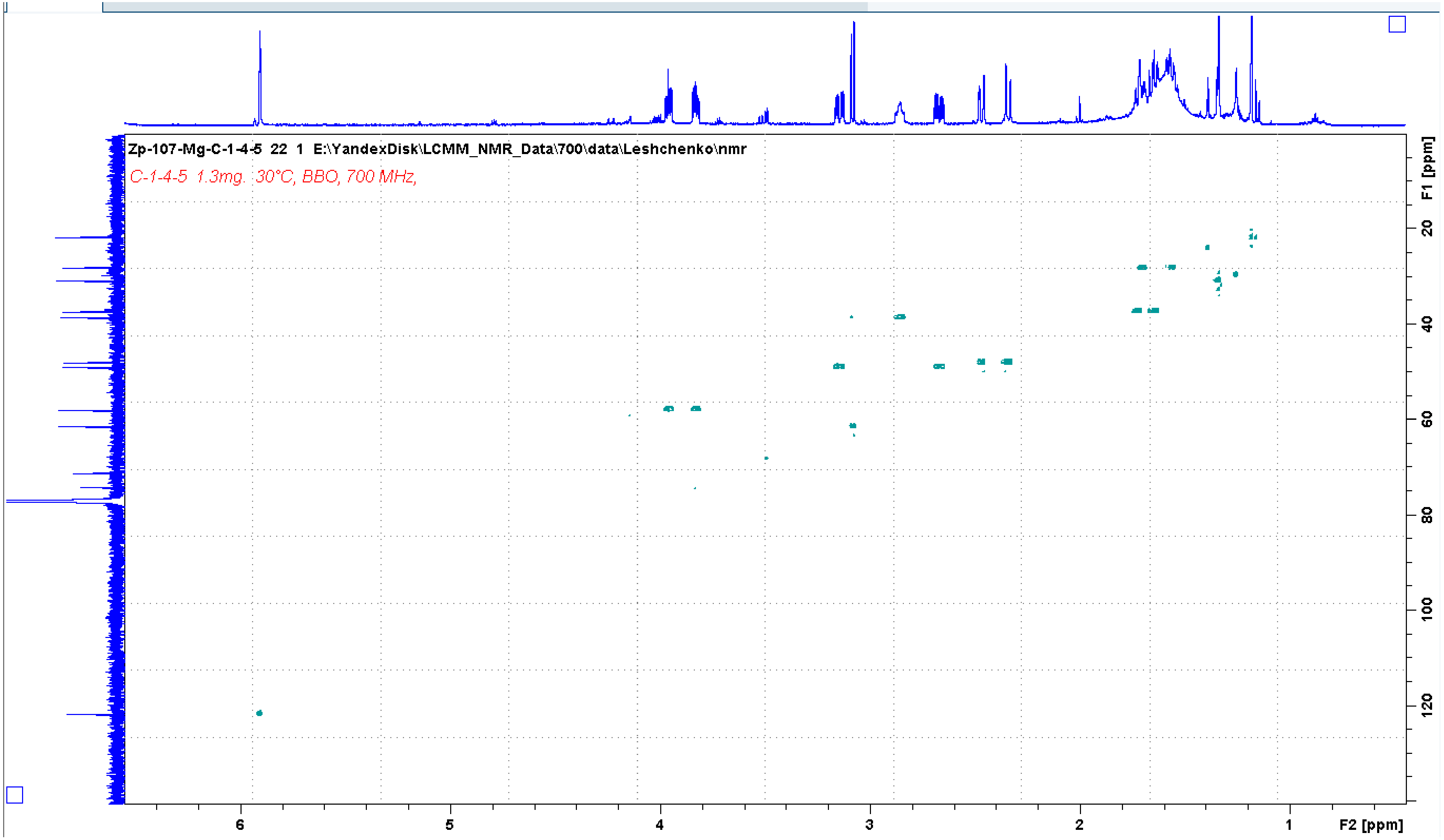

Figure S40. COSY spectrum of **4** measured in $CDCl_3$

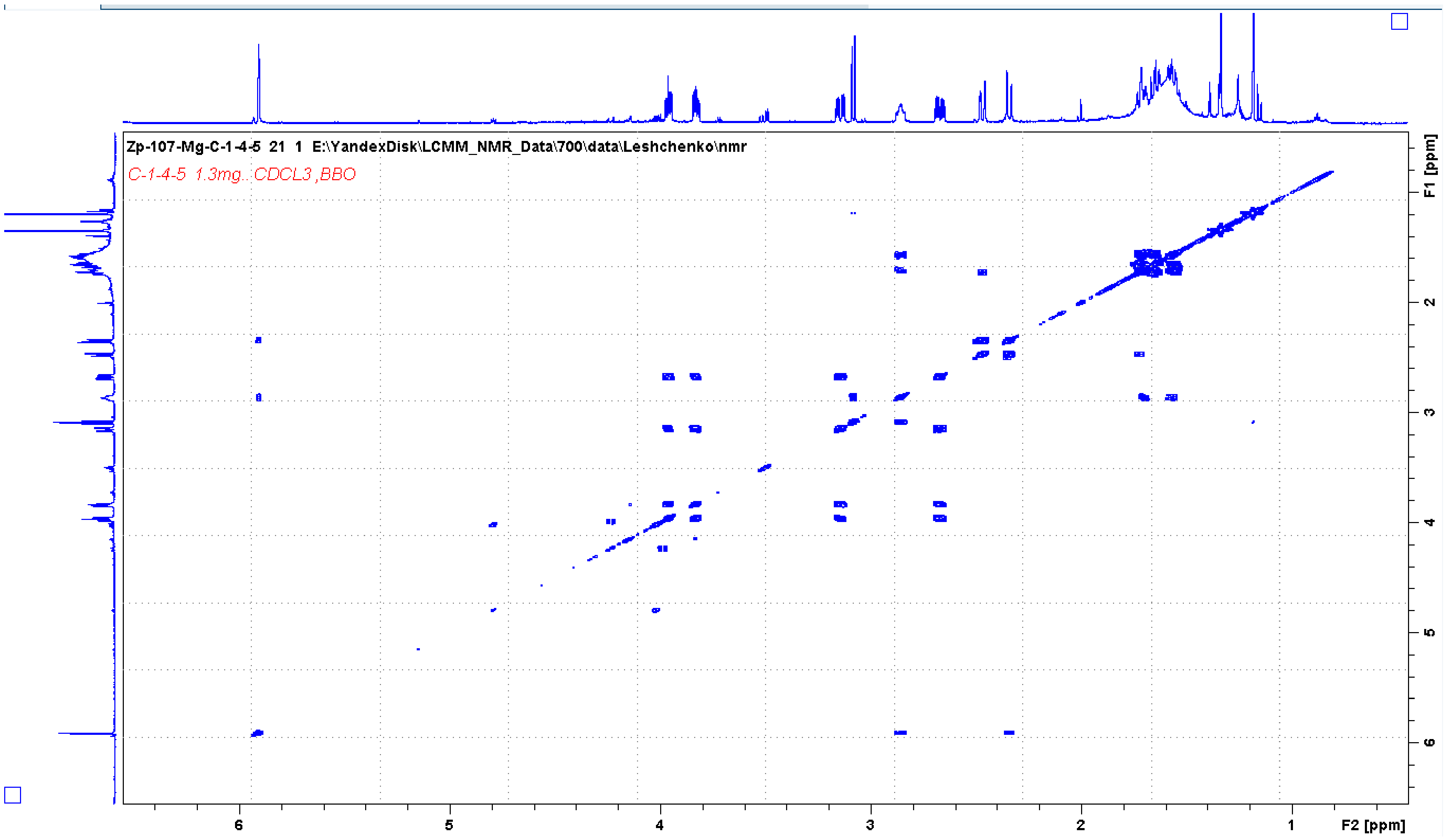

Figure S41. HMBC spectrum of **4** measured in $CDCl_3$

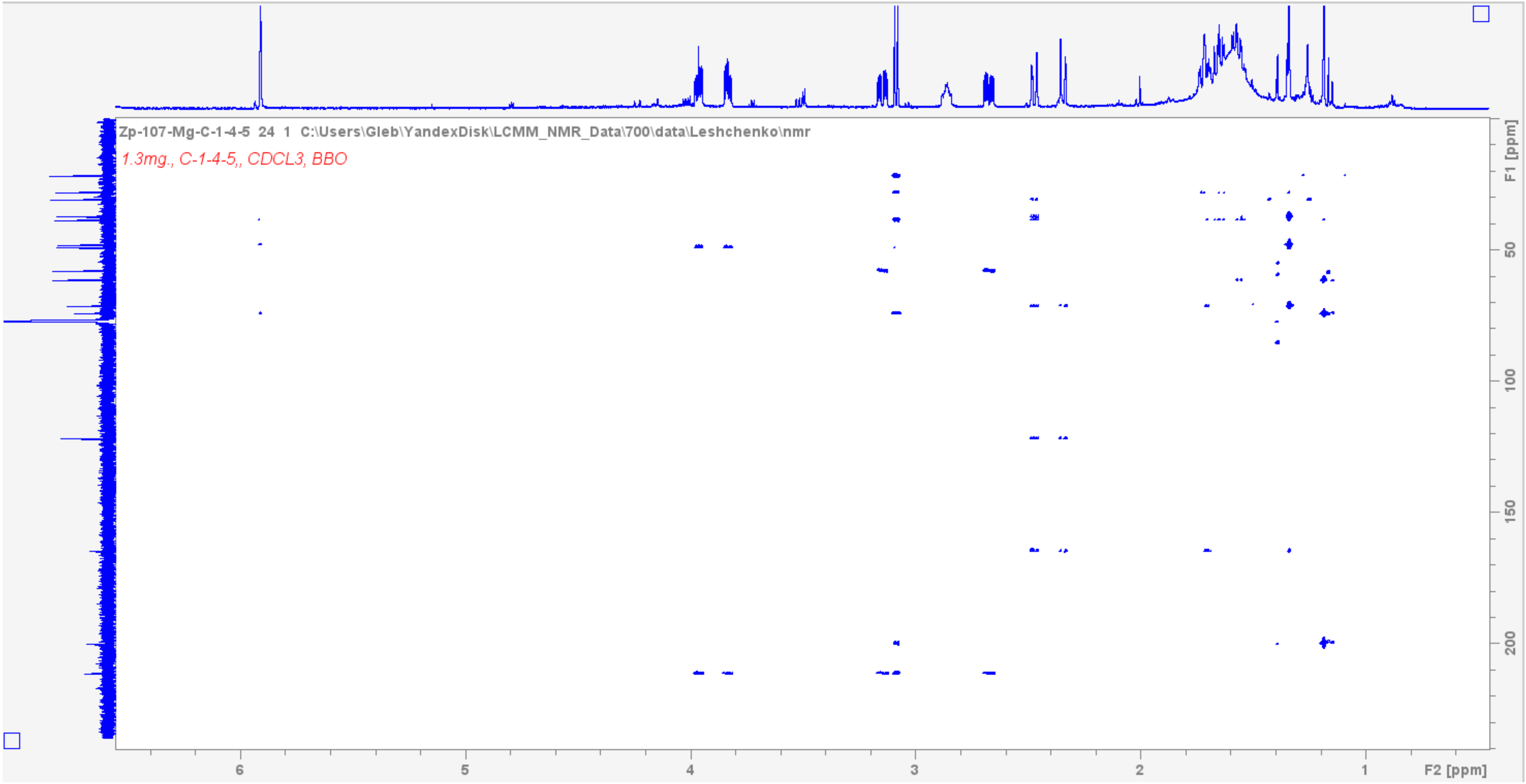

Figure S42. ROESY spectrum of **4** measured in $CDCl_3$

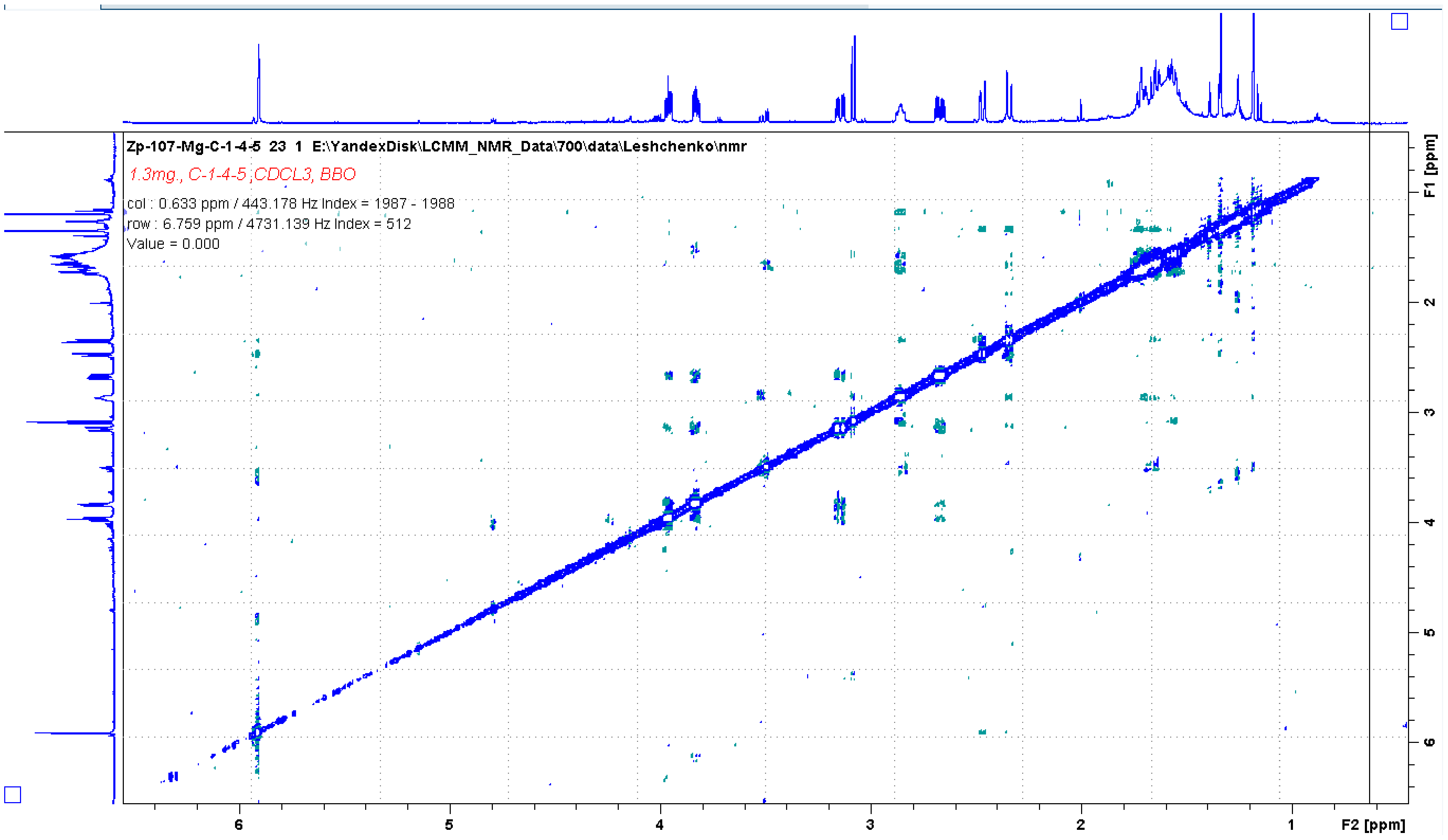

Figure S43. UV spectrum of **4** measured in MeOH

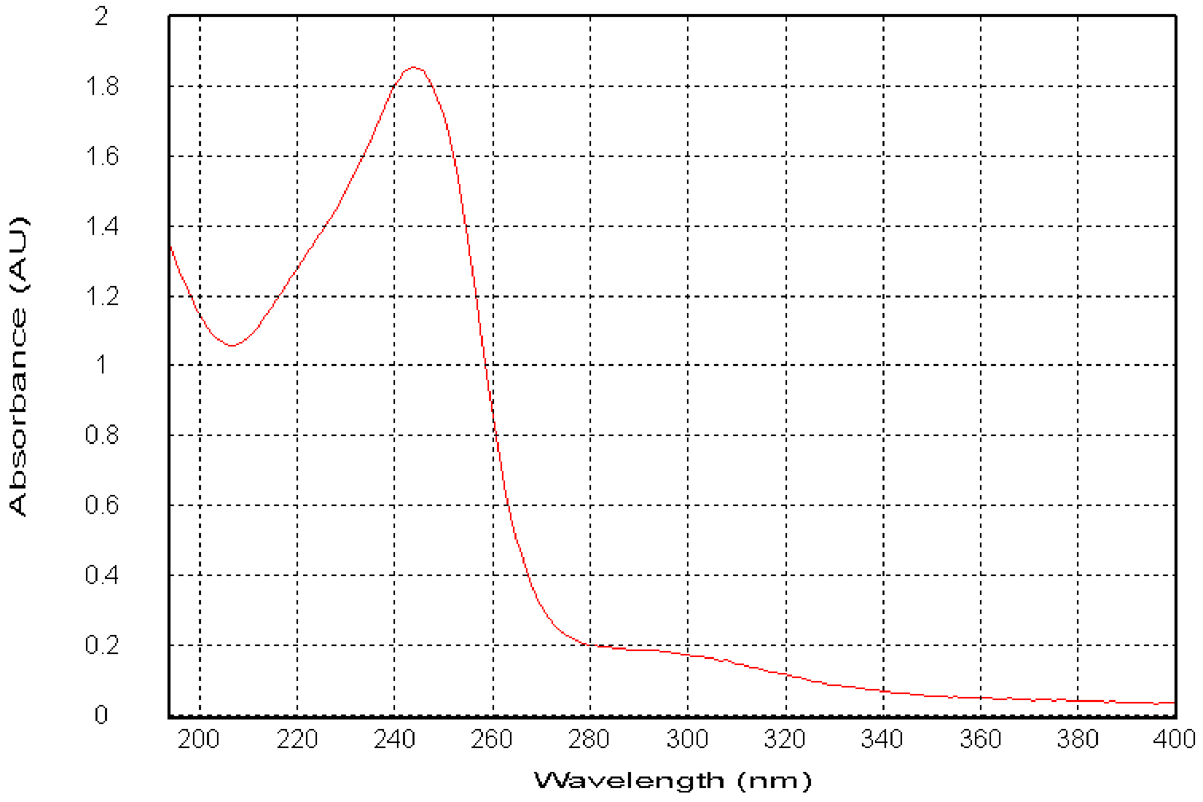

Figure S44. CD spectrum of **4** measured in MeOH

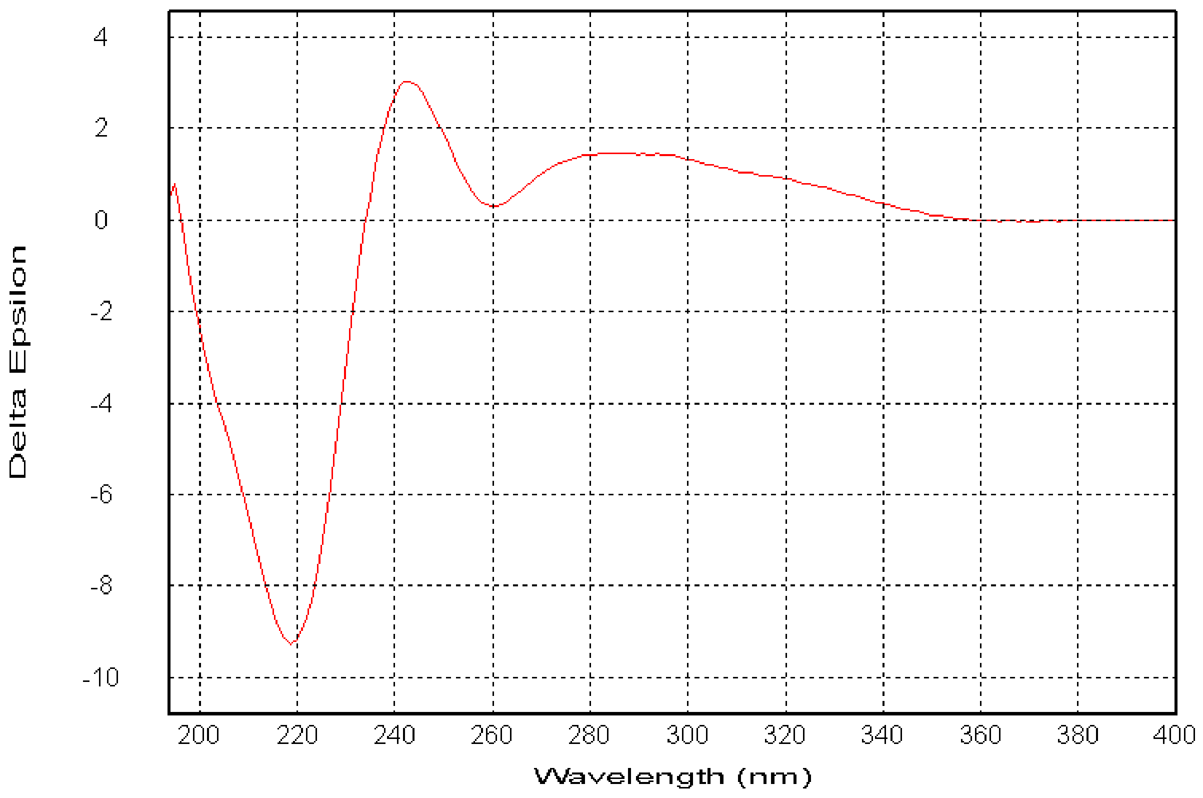

Figure S45. (**A**) Key COSY, (**B**) HMBC and (**C**) ROESY correlations of **5**

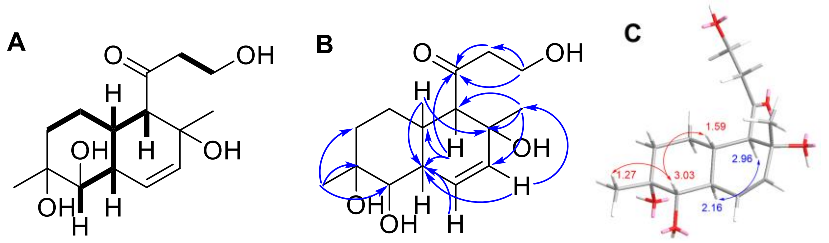

Figure S46. HRESIMS for **5**

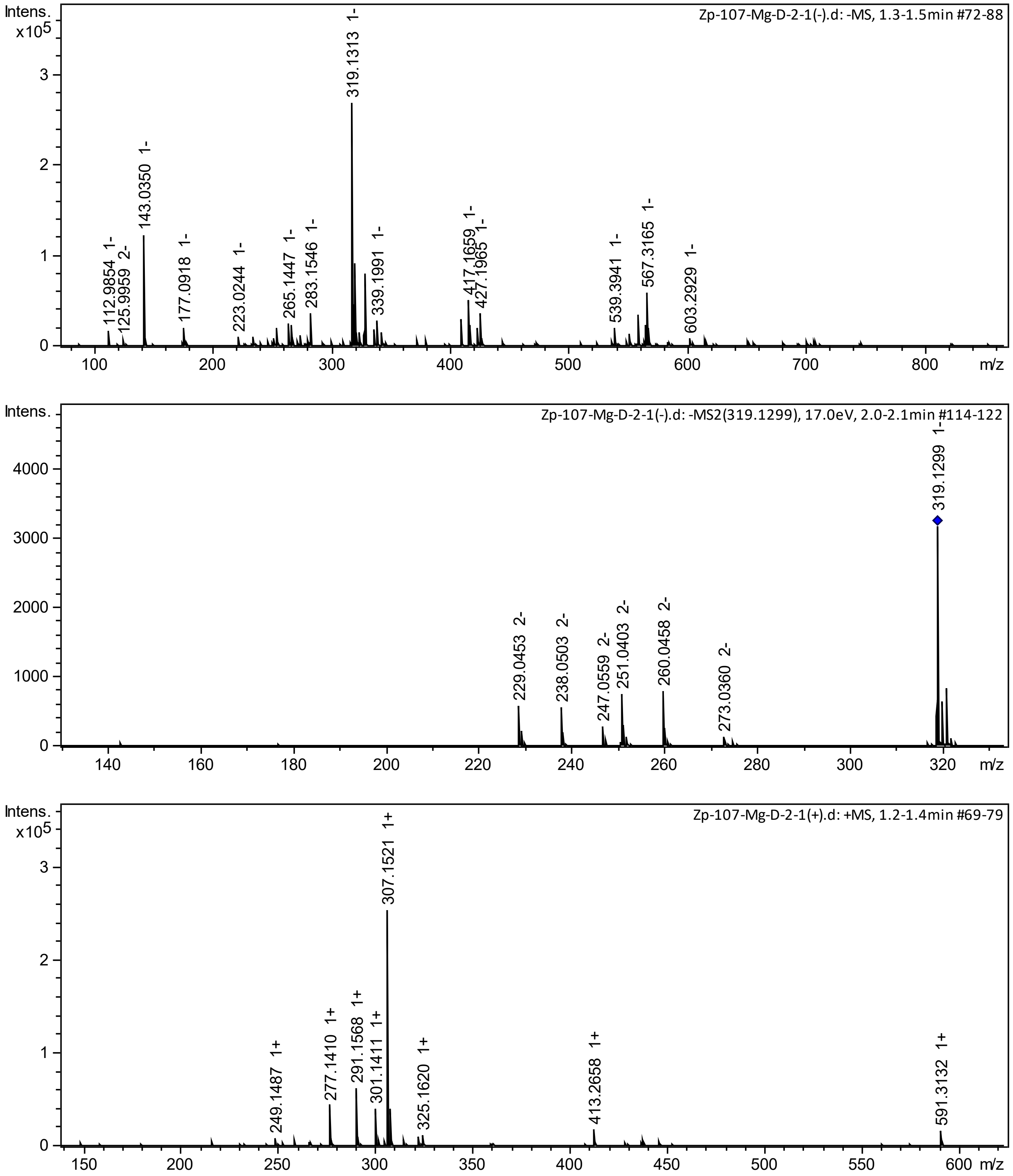

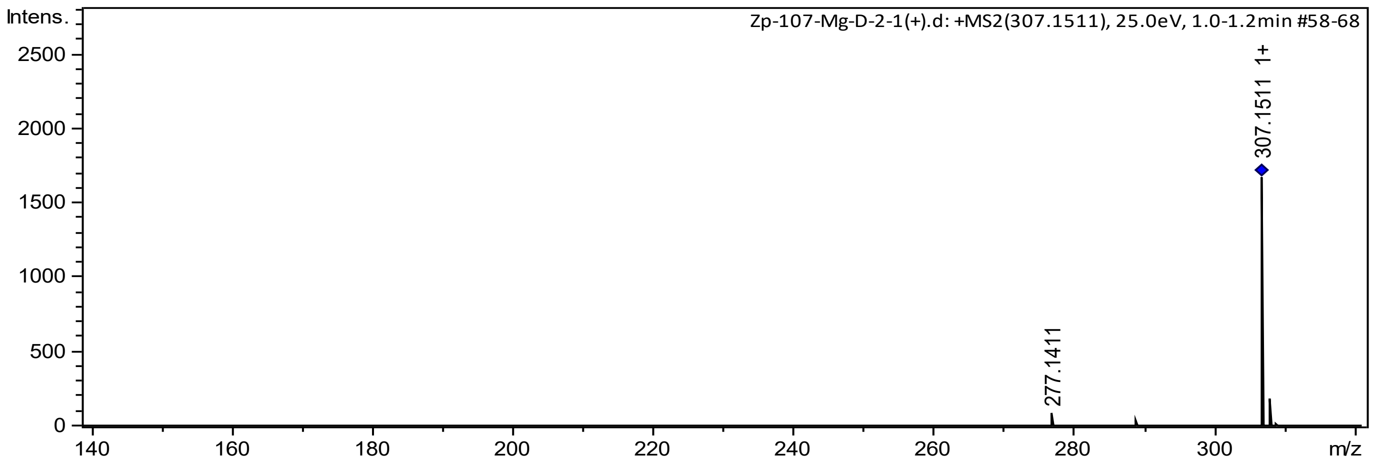

	meas	calc	Δ (ppm)
[M-H]$^-$	283,1546	283,1551	1,8
[M+Cl]$^-$	319,1313	319,1318	1,6
[M+Na]$^+$	307,1521	307,1516	-1,6

Figure S47. ^{1}H NMR spectrum of **5** measured at 700 MHz in $CDCl_3$

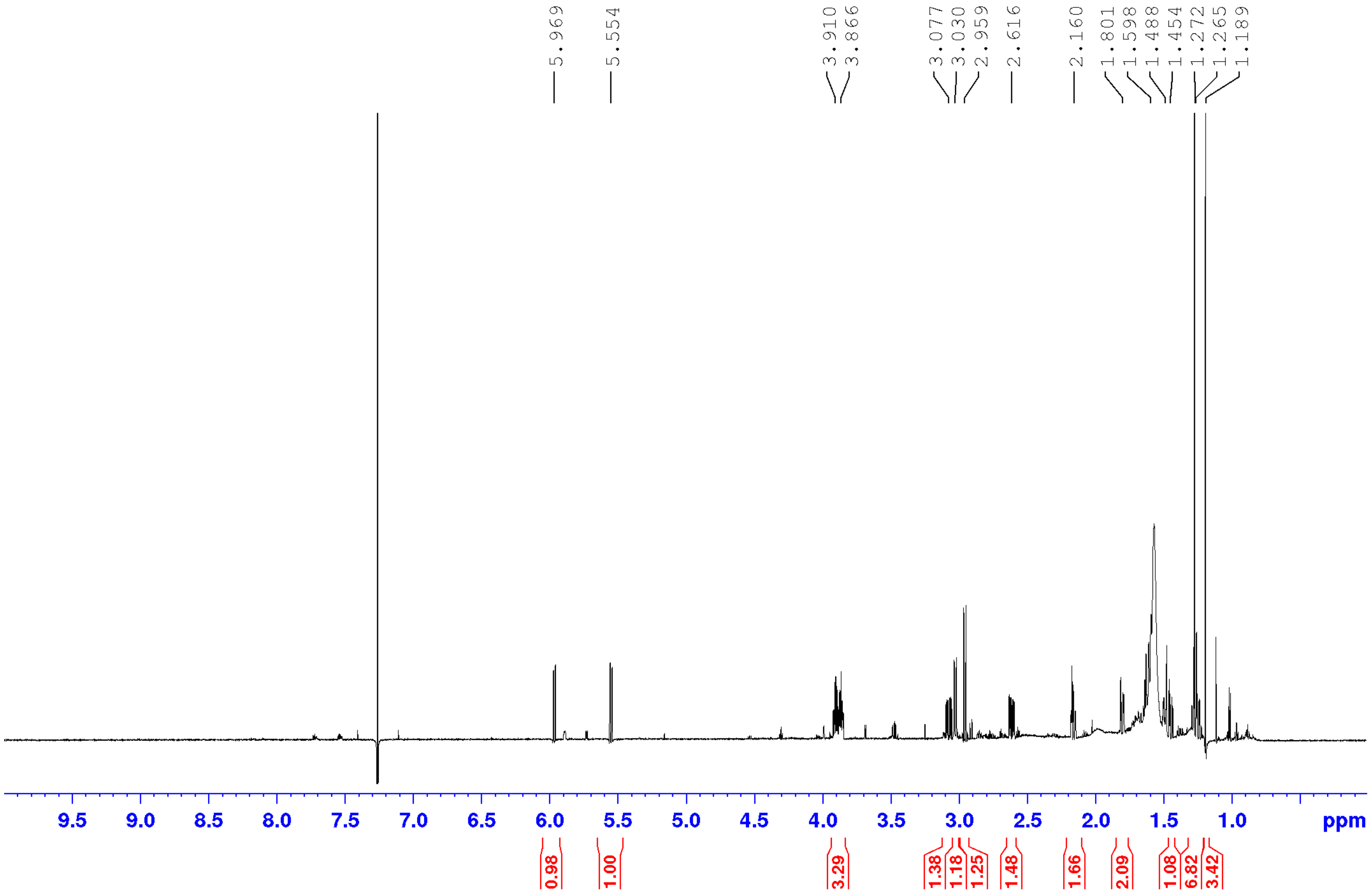

Figure S48. ^{13}C NMR spectrum of **5** measured at 176 MHz in $CDCl_3$

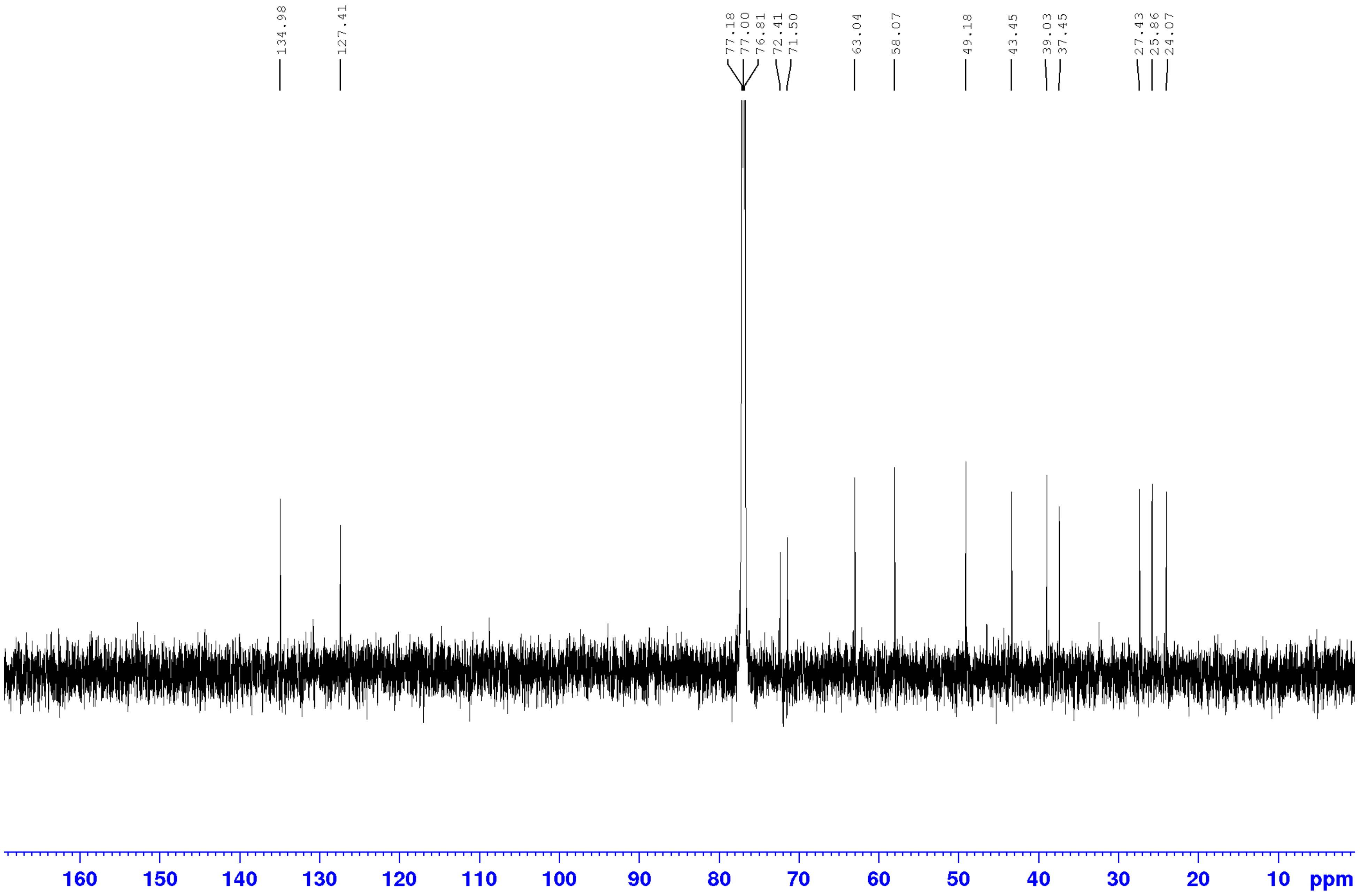

Figure S49. DEPT-135 spectrum of **5** measured at 176 MHz in $CDCl_3$

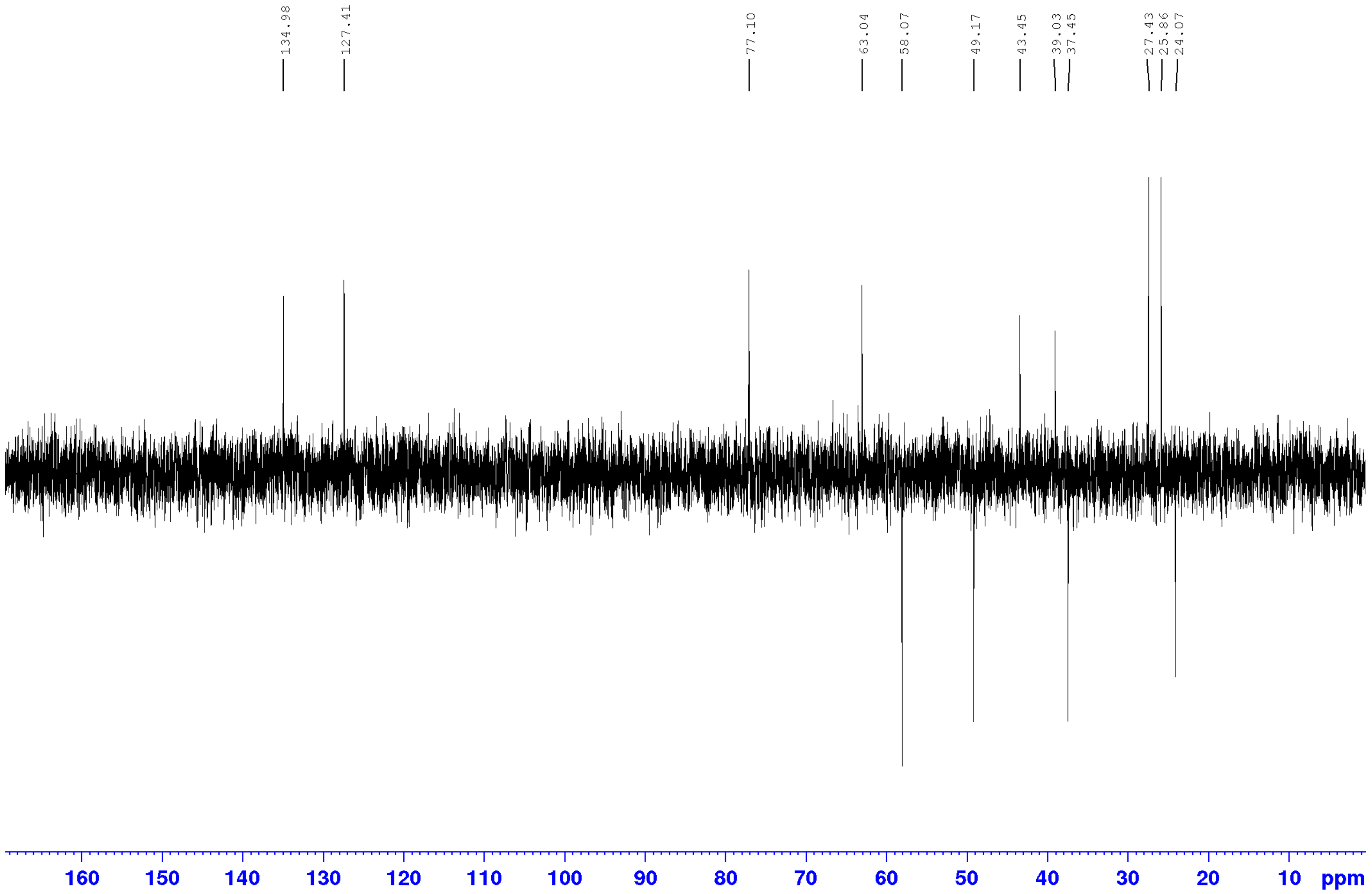

Figure S50. HSQC spectrum of **5** measured in $CDCl_3$

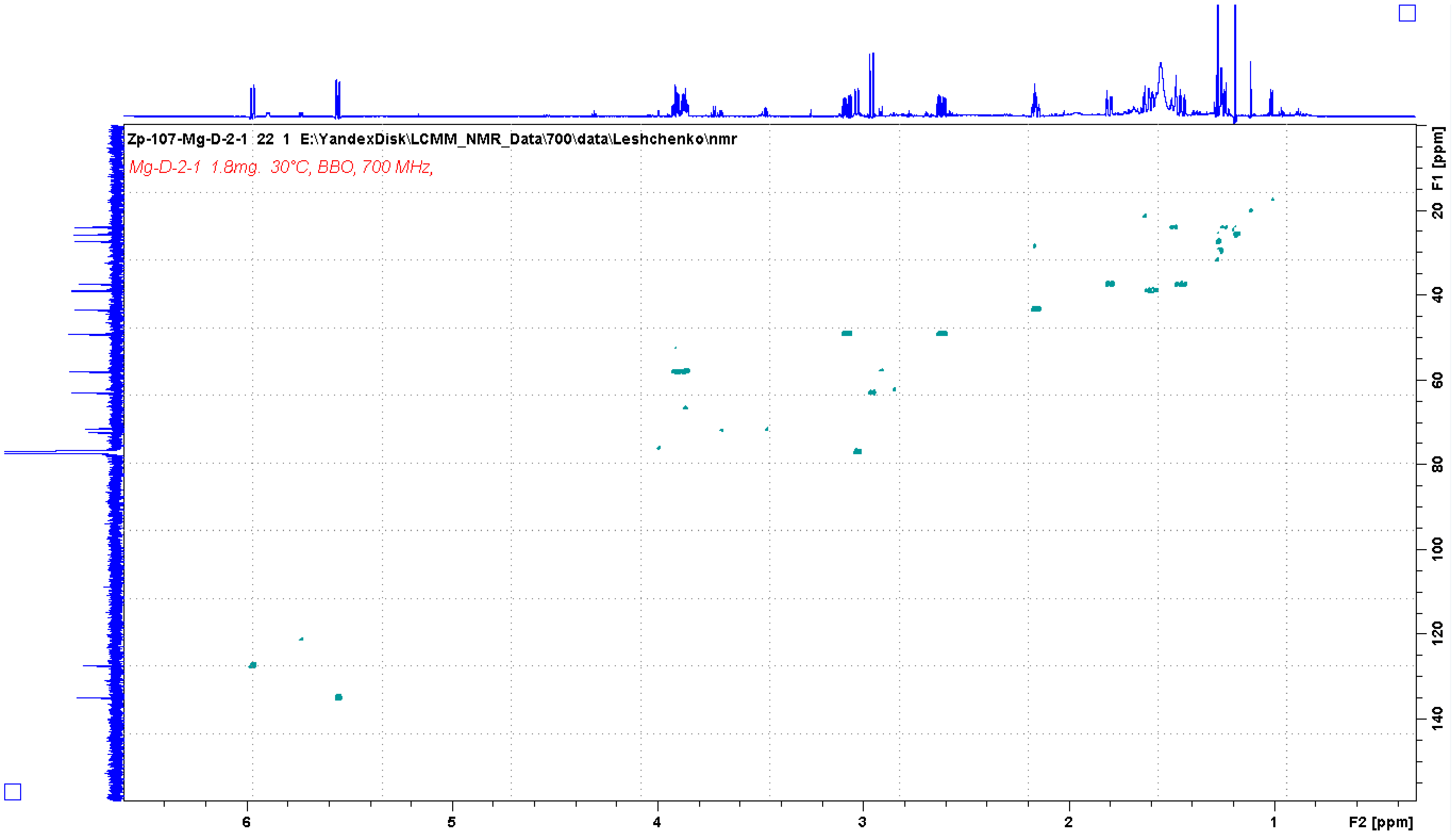

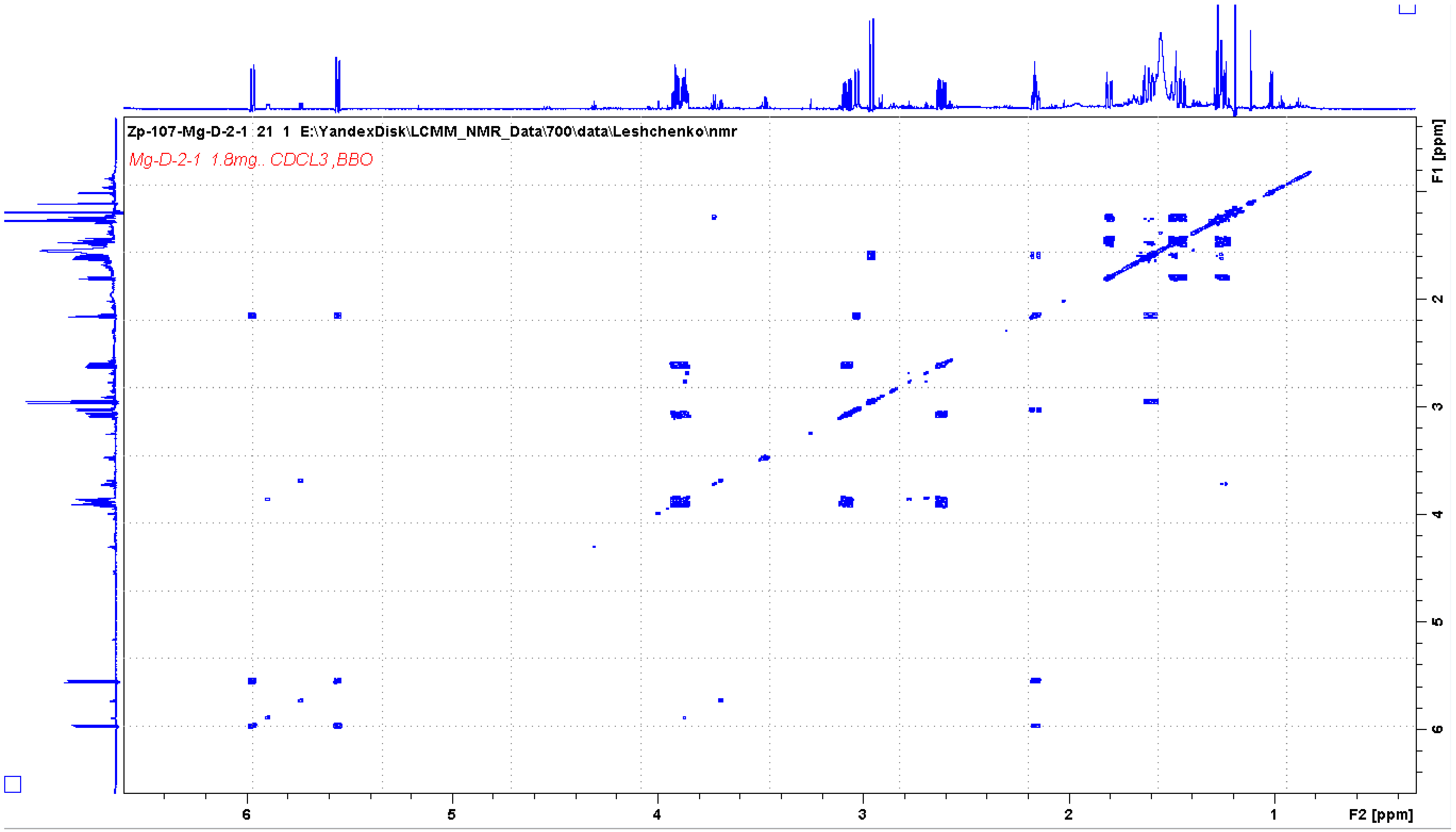

Figure S51. COSY spectrum of **5** measured in $CDCl_3$

Figure S52. HMBC spectrum of **5** measured in $CDCl_3$

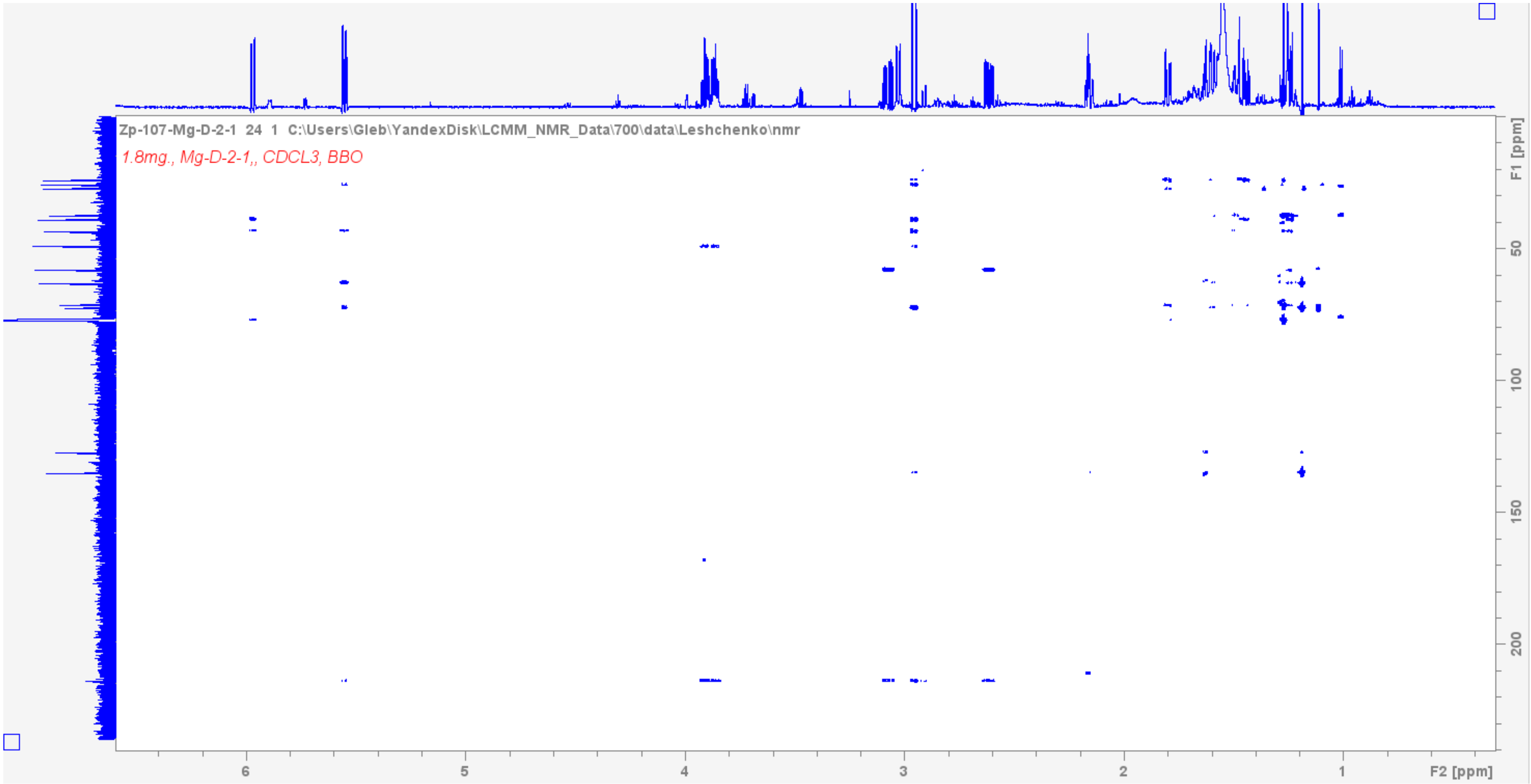

Figure S53. ROESY spectrum of **5** measured in $CDCl_3$

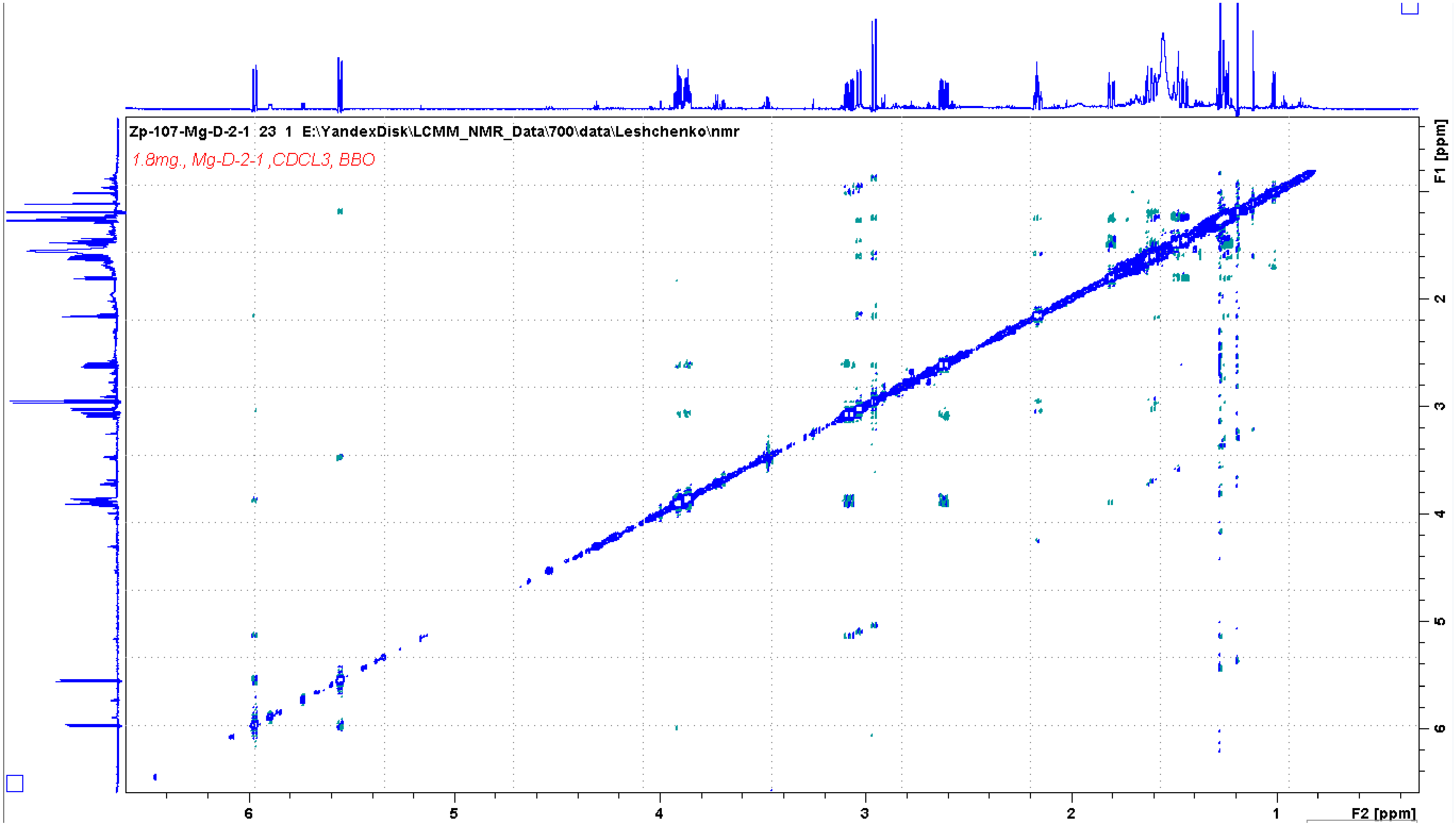

Figure S54. UV spectrum of **5** measured in $CDCl_3$

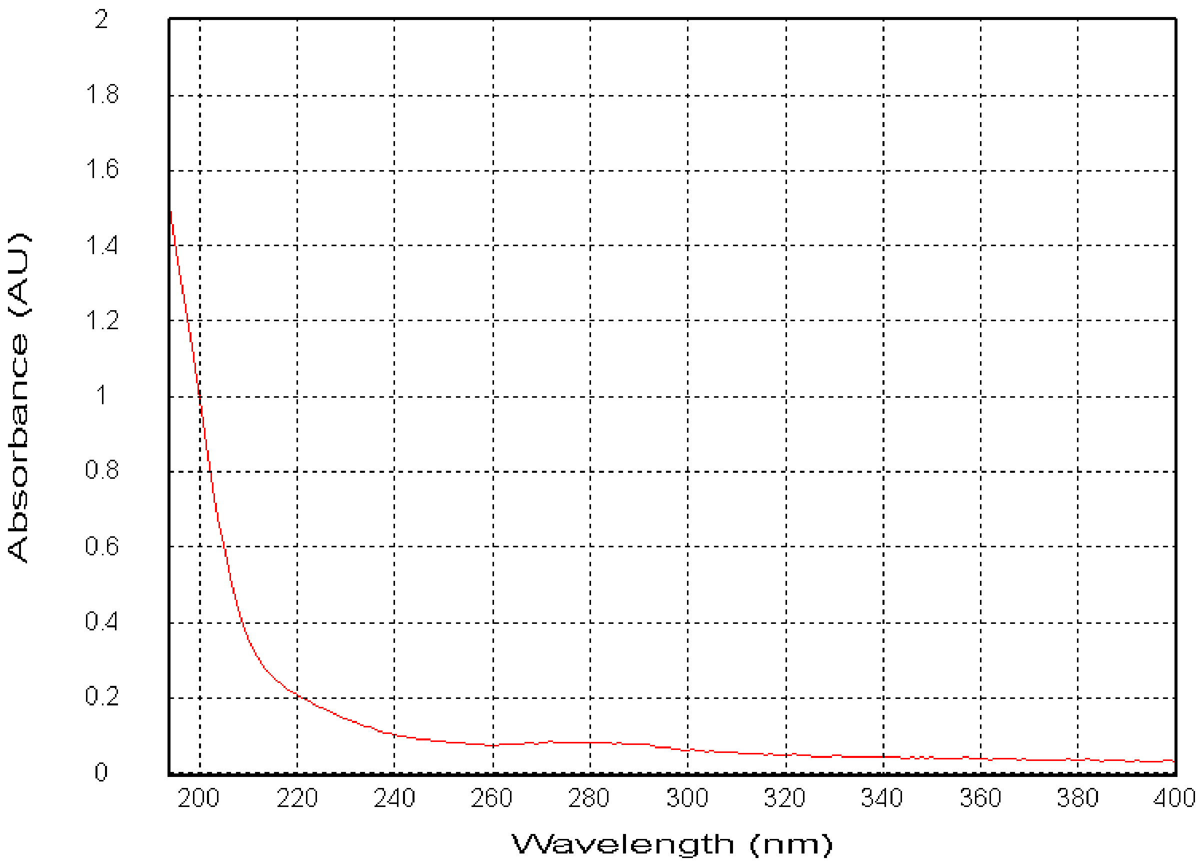

Figure S55. CD spectrum of **5** measured in $CDCl_3$

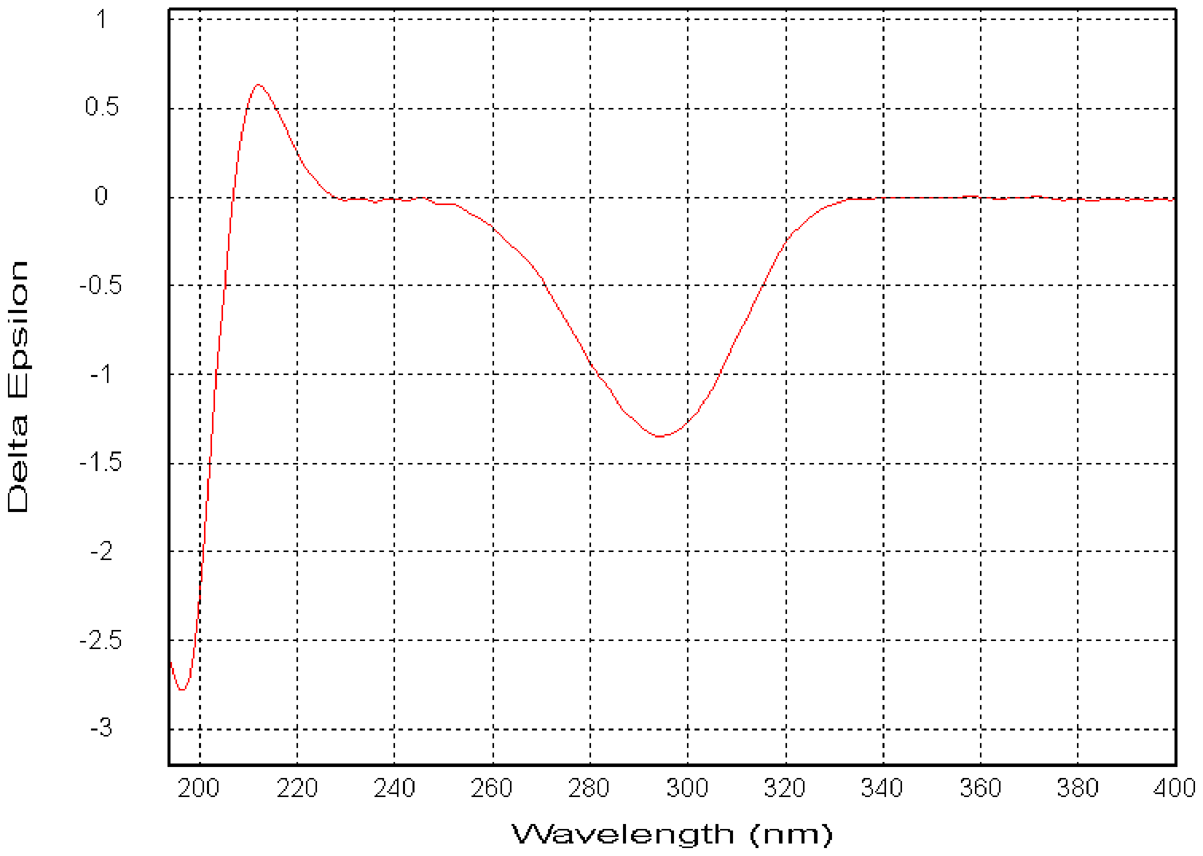

Figure S56. (**A**) Key COSY, (**B**) HMBC and (**C**) ROESY correlations of **6**

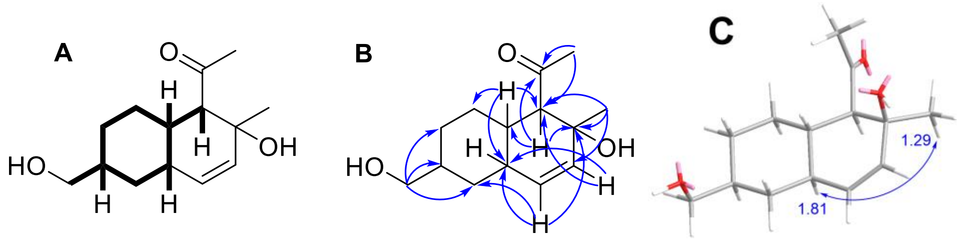

Figure S57. HRESIMS for **6**

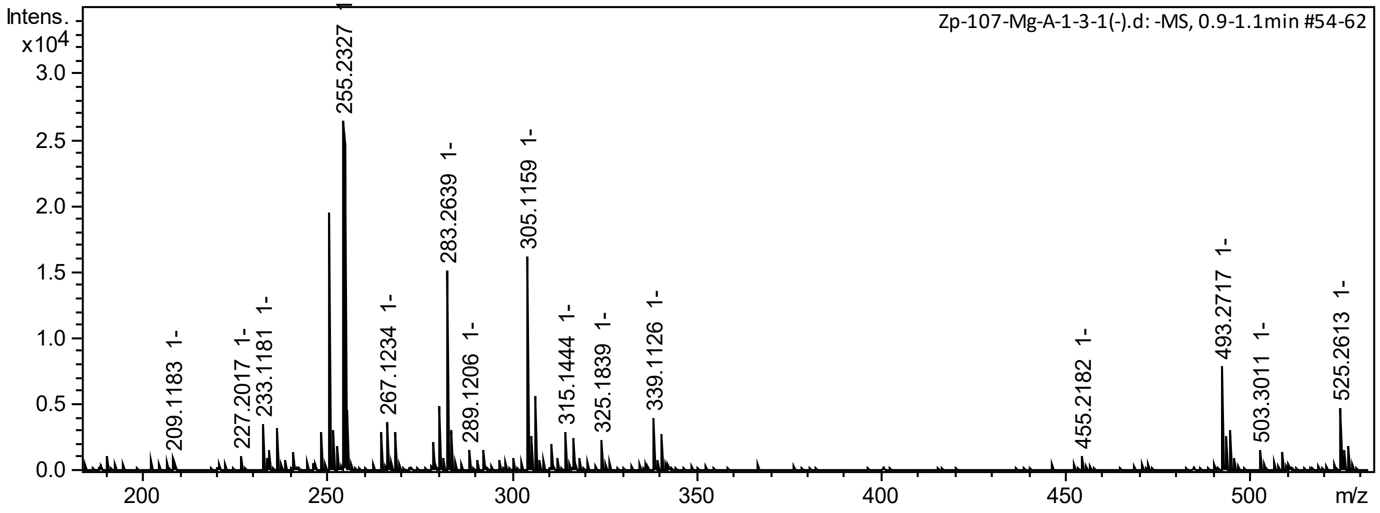

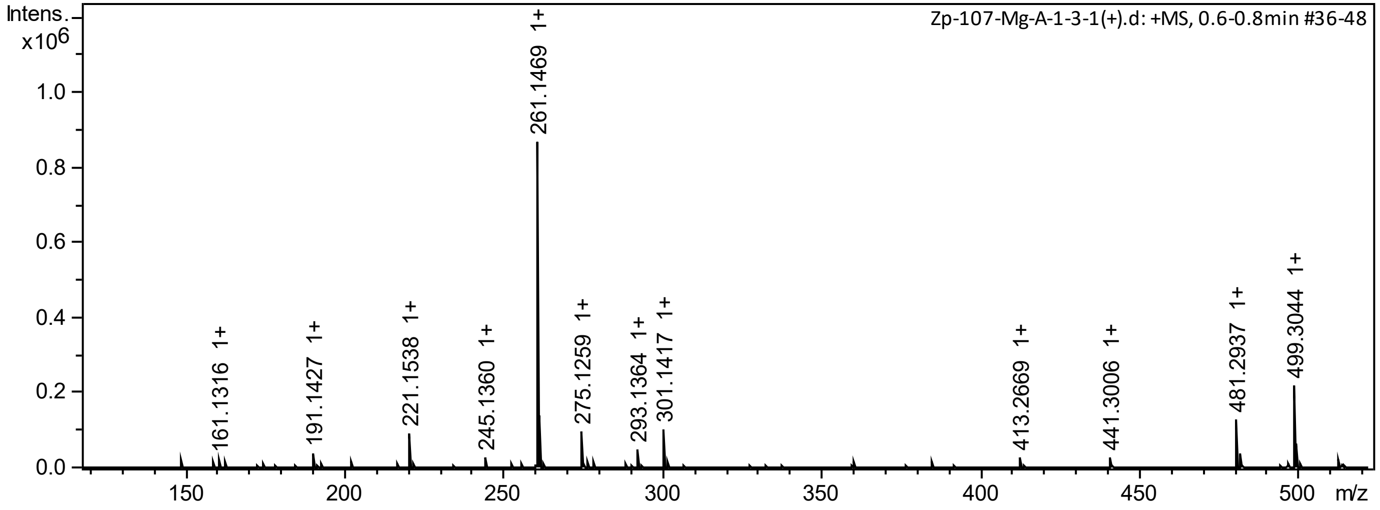

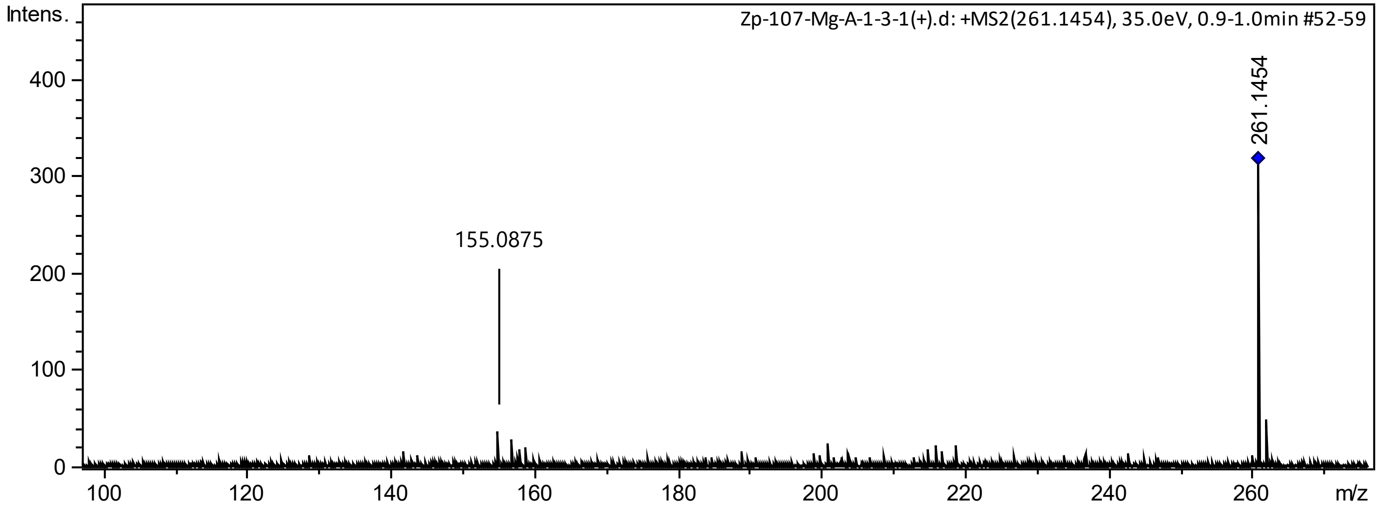

	meas	calc	Δ (ppm)
$[M-H]^-$	237,1493	237,1496	1,3
$[M+Na]^+$	261,1469	261,1461	-3,1

Figure S58. ^{1}H NMR spectrum of **6** measured at 700 MHz in $CDCl_3$

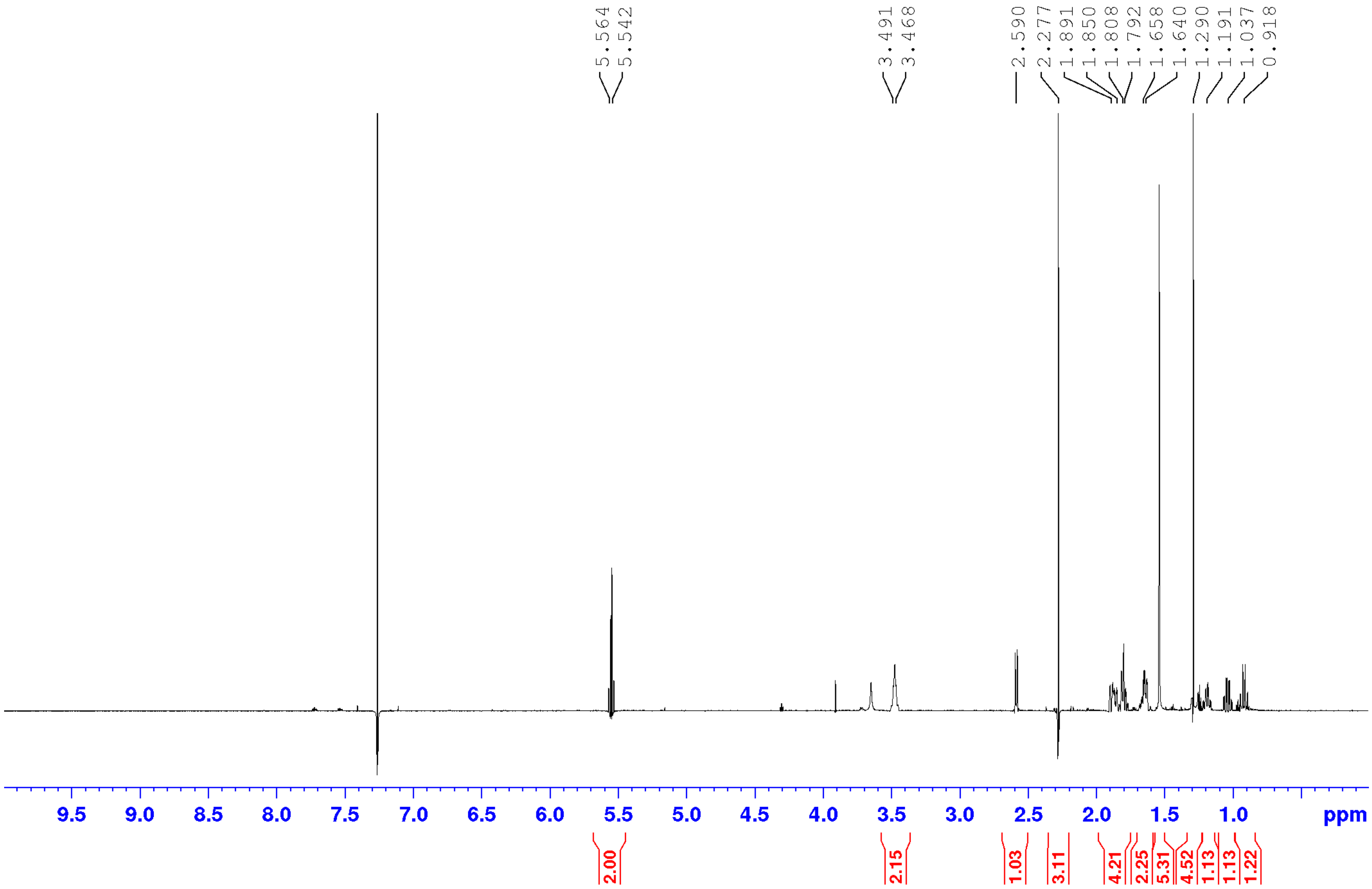

Figure S59. ^{13}C NMR spectrum of **6** measured at 176 MHz in $CDCl_3$

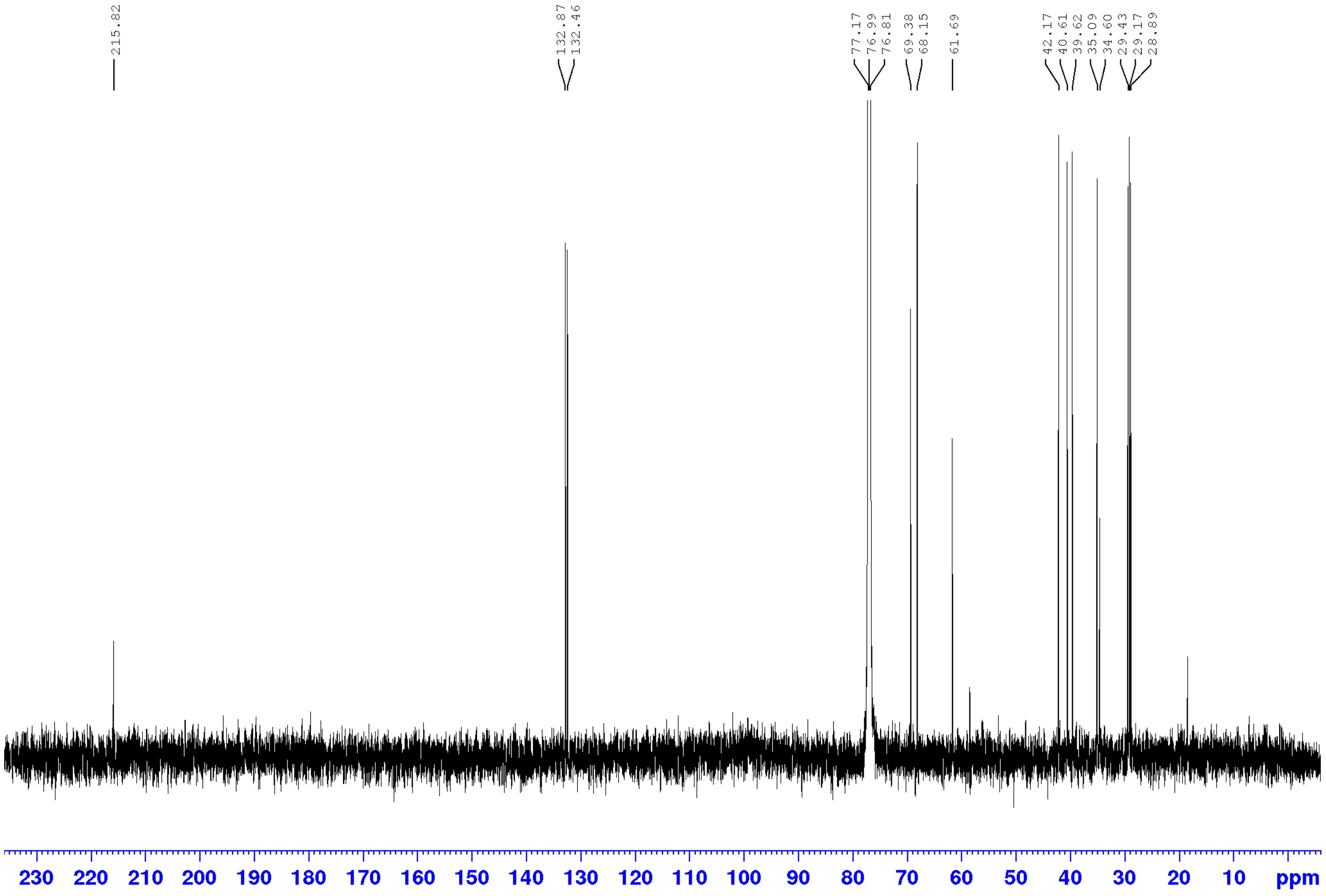

Figure S60. DEPT-135 spectrum of **6** measured at 176 MHz in $CDCl_3$

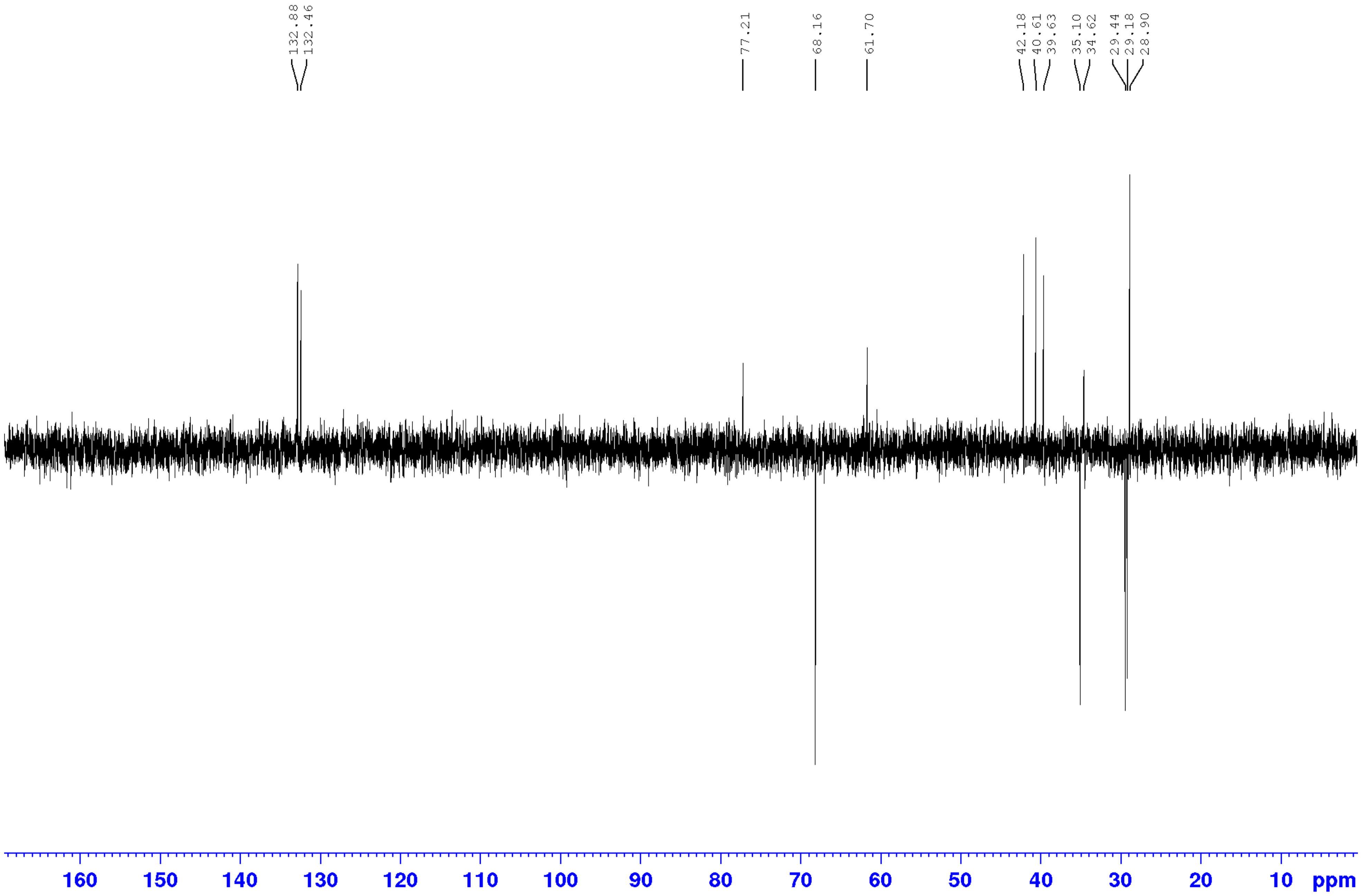

Figure S61. HSQC spectrum of **6** measured in $CDCl_3$

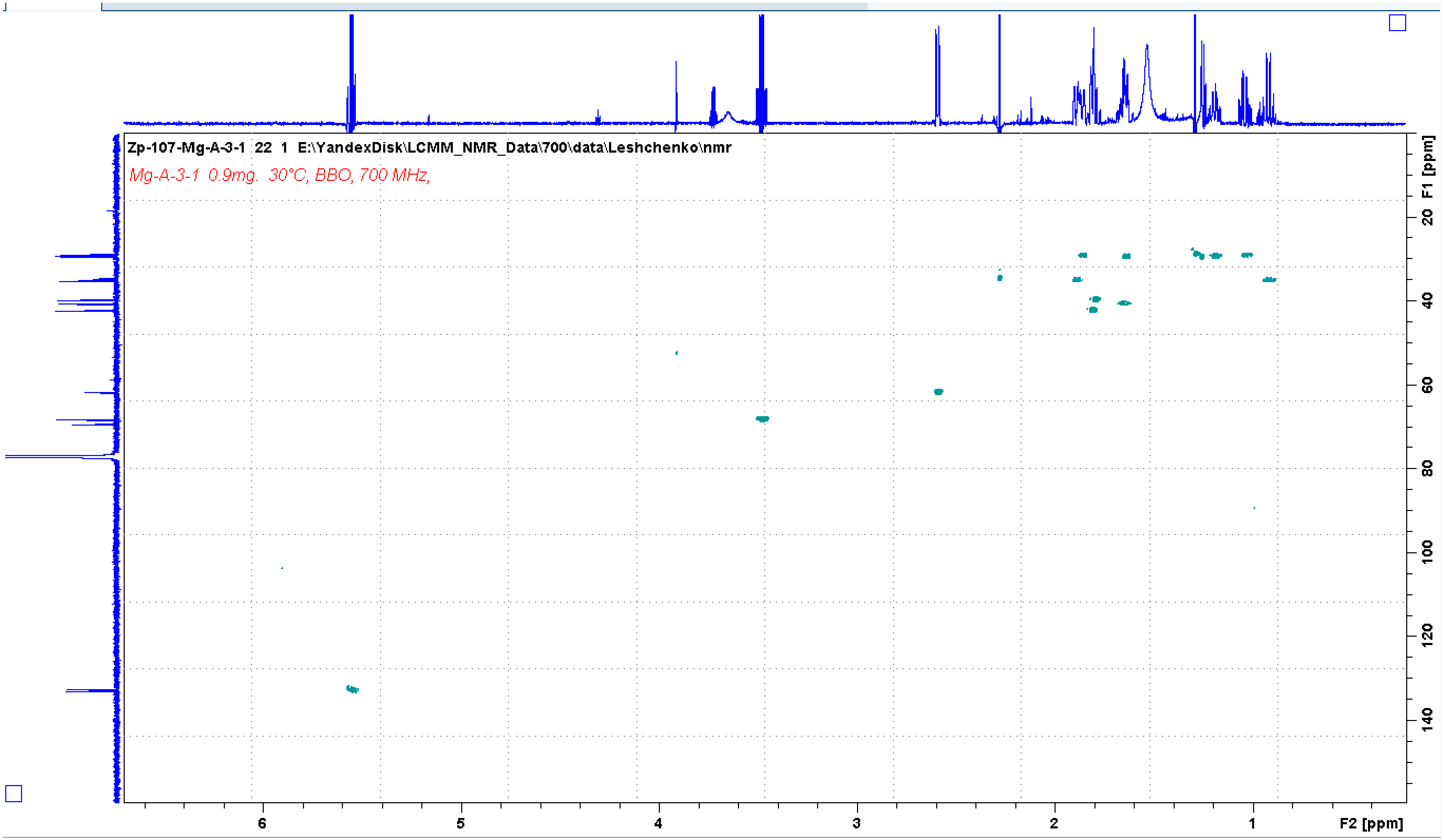

Figure S62. COSY spectrum of **6** measured in $CDCl_3$

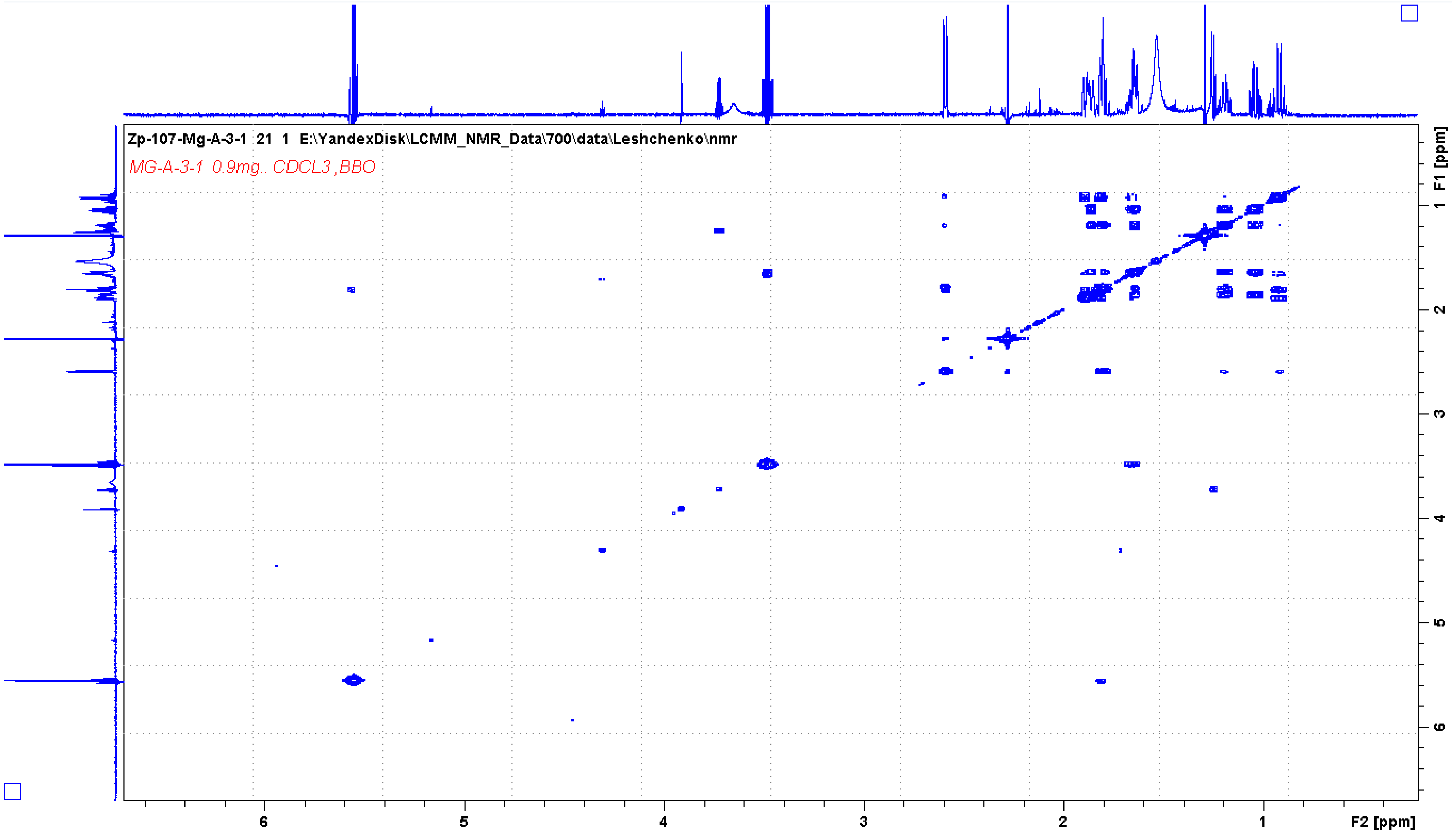

Figure S63. HMBC spectrum of **6** measured in $CDCl_3$

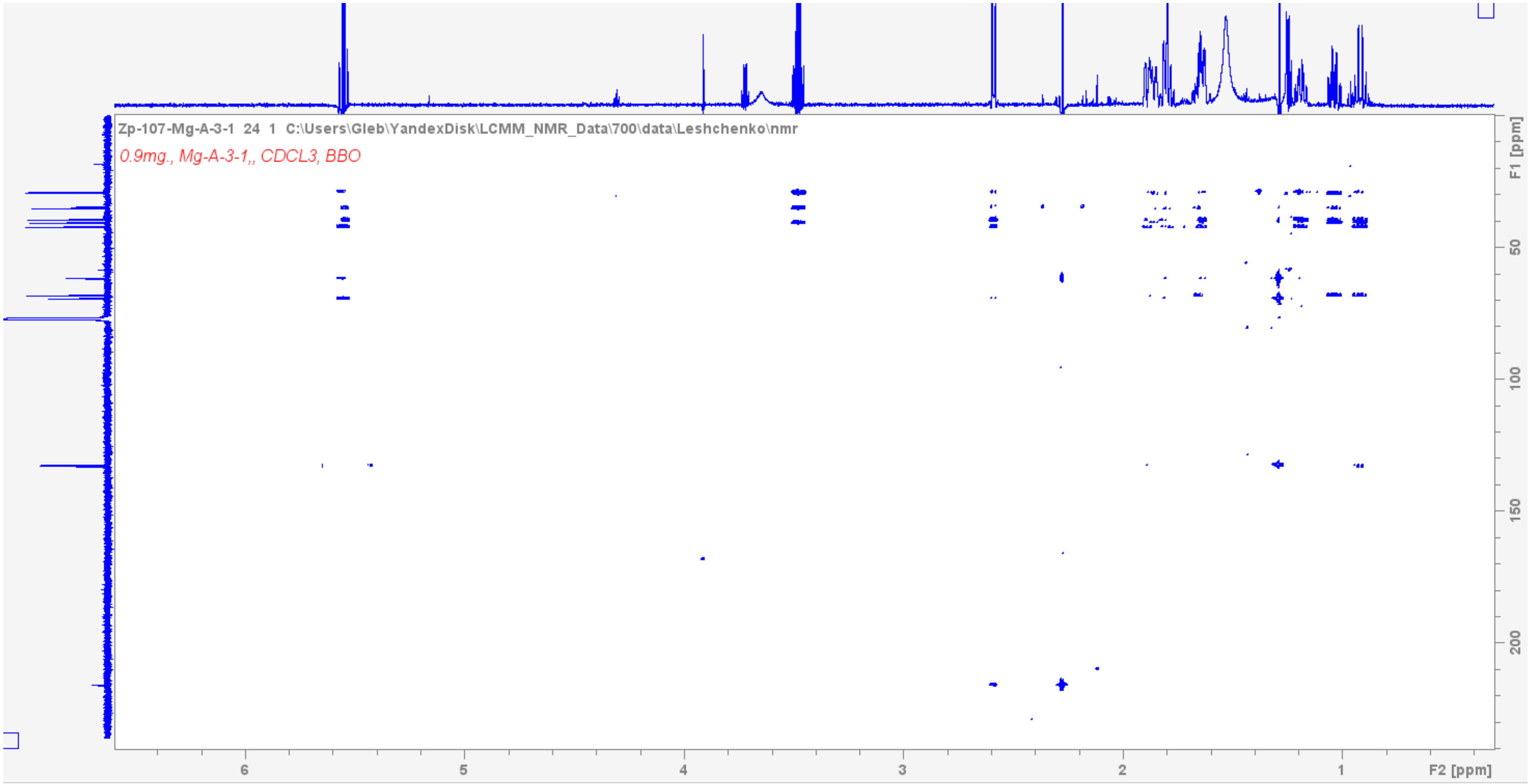

Figure S64. ROESY spectrum of **6** measured in $CDCl_3$

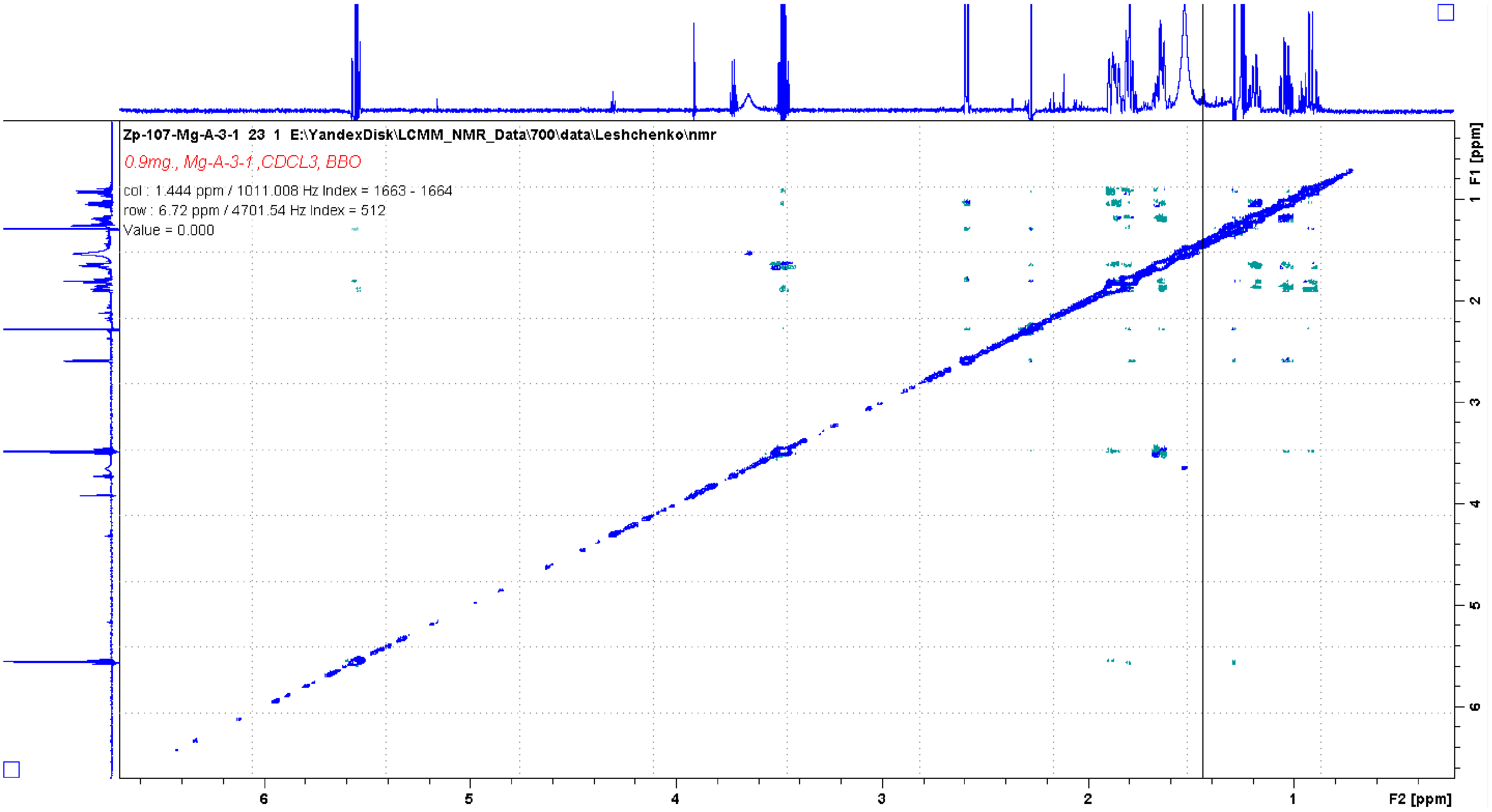

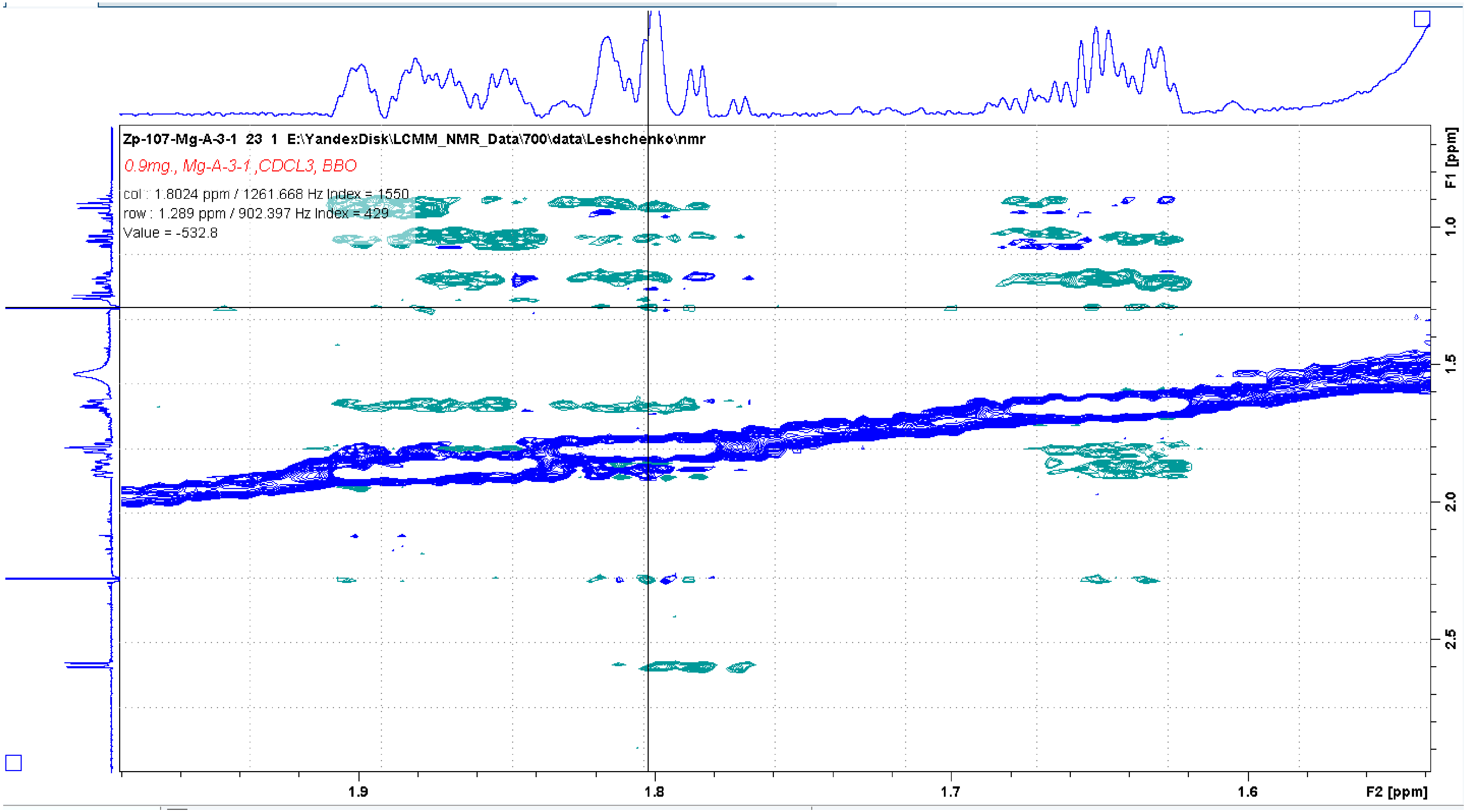

Zp-107-Mg-A-3-1 23 1 E:\YandexDisk\LCMM_NMR_Data\700\data\Leshchenko\nmr
0.9mg., Mg-A-3-1 ,CDCL3, BBO
col : 1.8024 ppm / 1261.668 Hz Index = 1550
row : 1.289 ppm / 902.397 Hz Index = 429
Value = -532.8
F1 [ppm]
1.0
1.5
2.0
2.5
1.9
1.8
1.7
1.6
F2 [ppm]

Figure S65. UV spectrum of **6** measured in MeOH

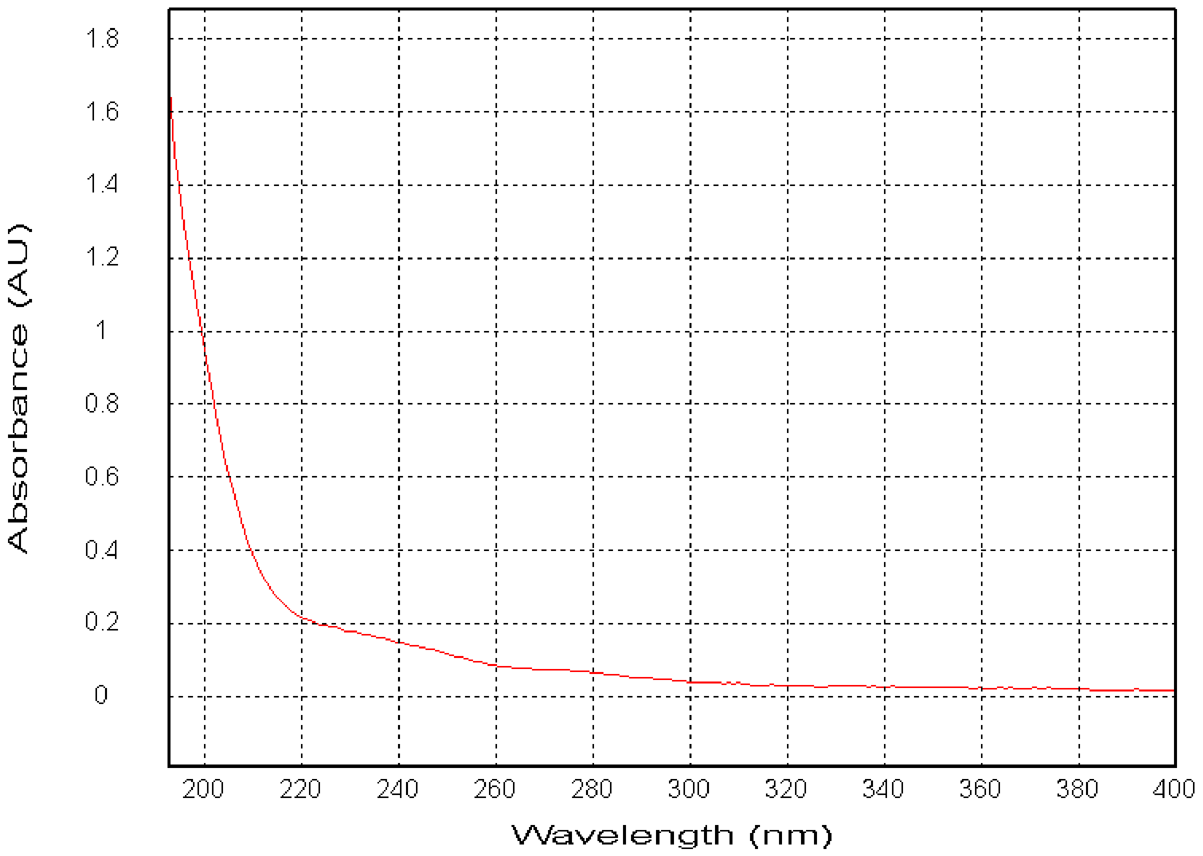

Figure S66. CD spectrum of **6** measured in MeOH

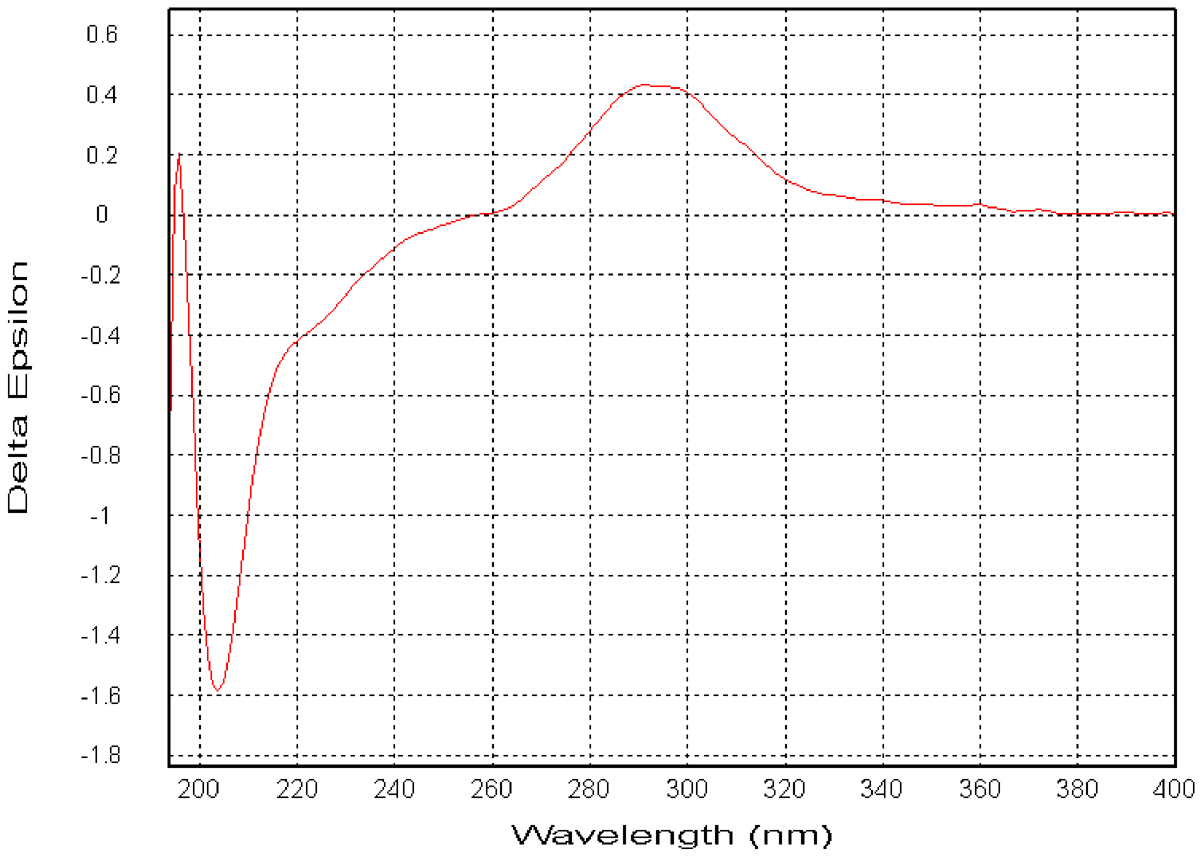

Figure S67. (**A**) Key COSY, (**B**) HMBC and (**C**) ROESY correlations of **7**

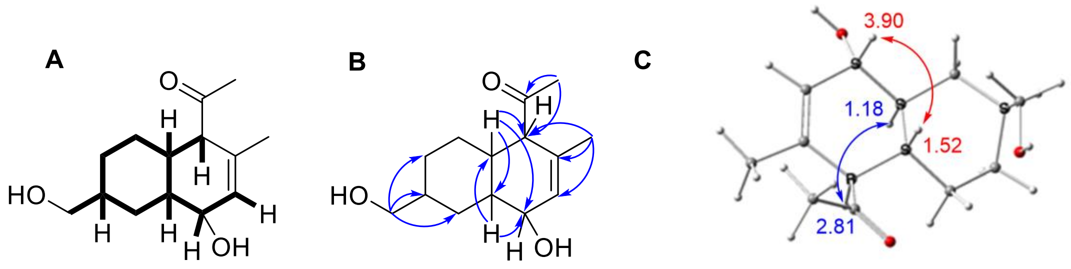

Figure S68. HRESIMS for **7**

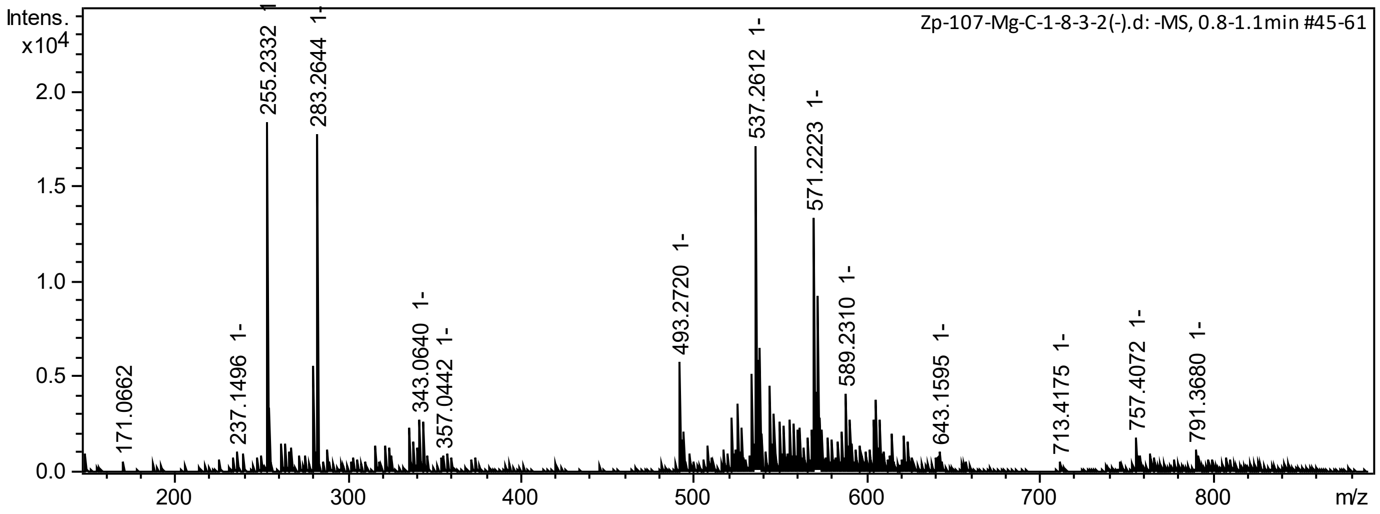

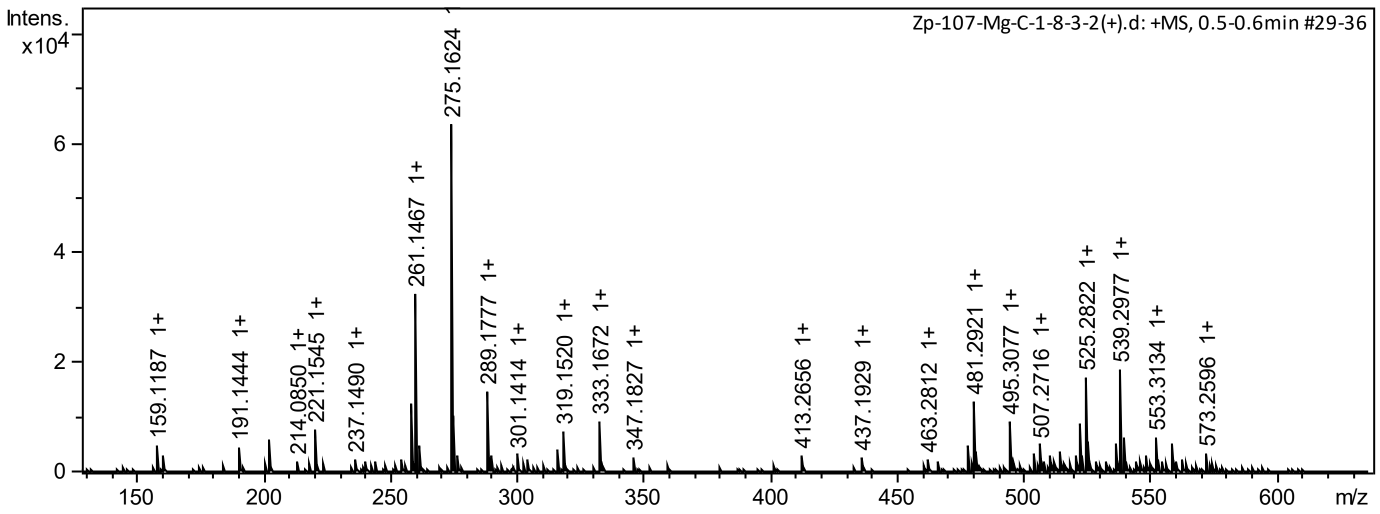

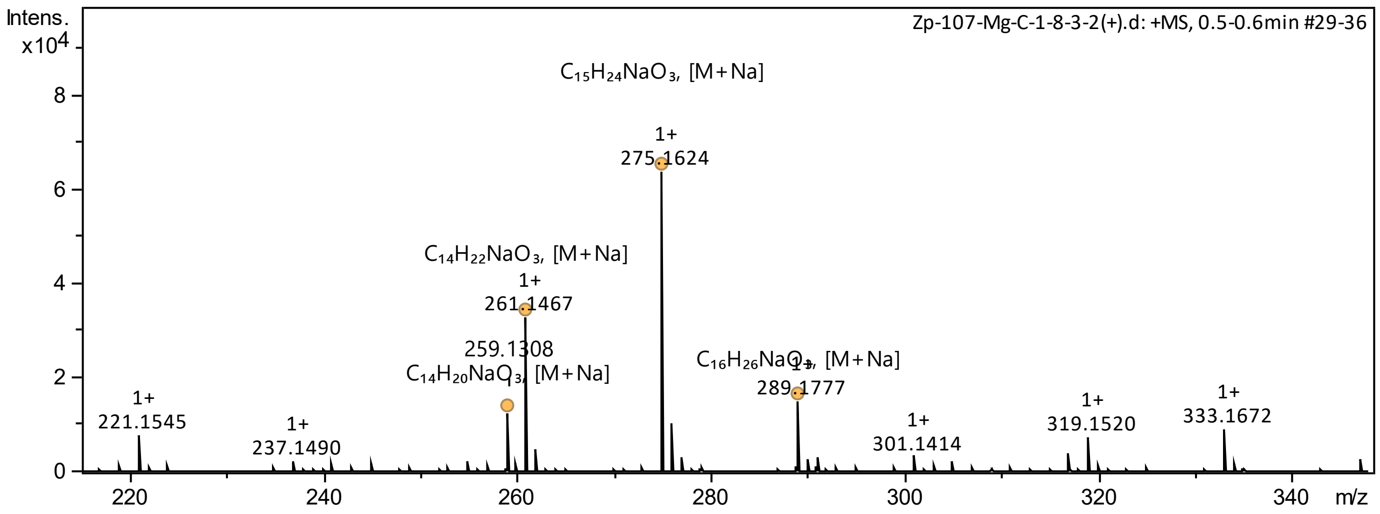

	meas	calc	Δ (ppm)
$[M+Na]^+$	261.1467	261.1461	2.23

Figure S69. ^{1}H NMR spectrum of **7** measured at 700 MHz in $CDCl_3$

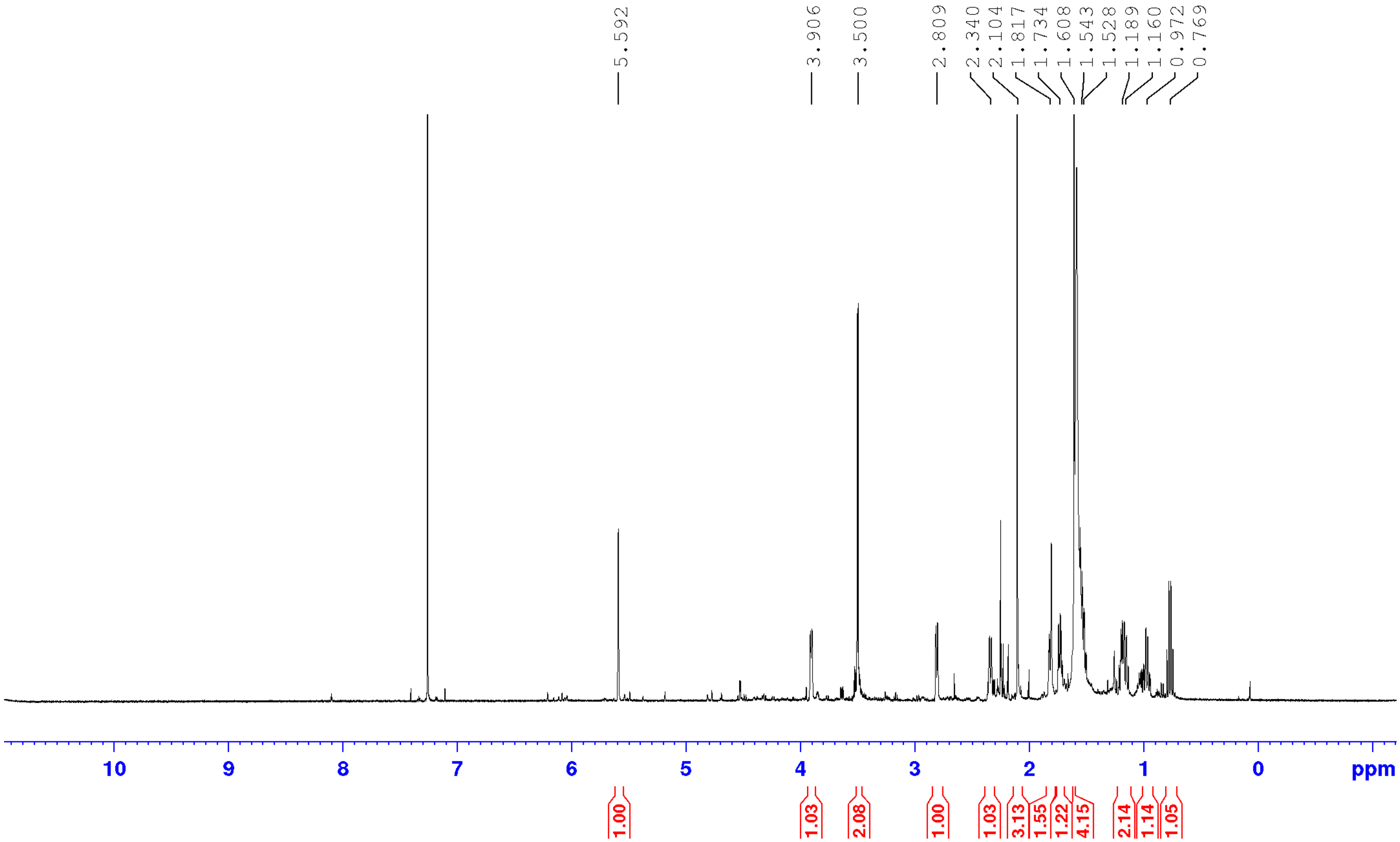

Figure S70. ^{13}C NMR spectrum of **7** measured at 176 MHz in $CDCl_3$

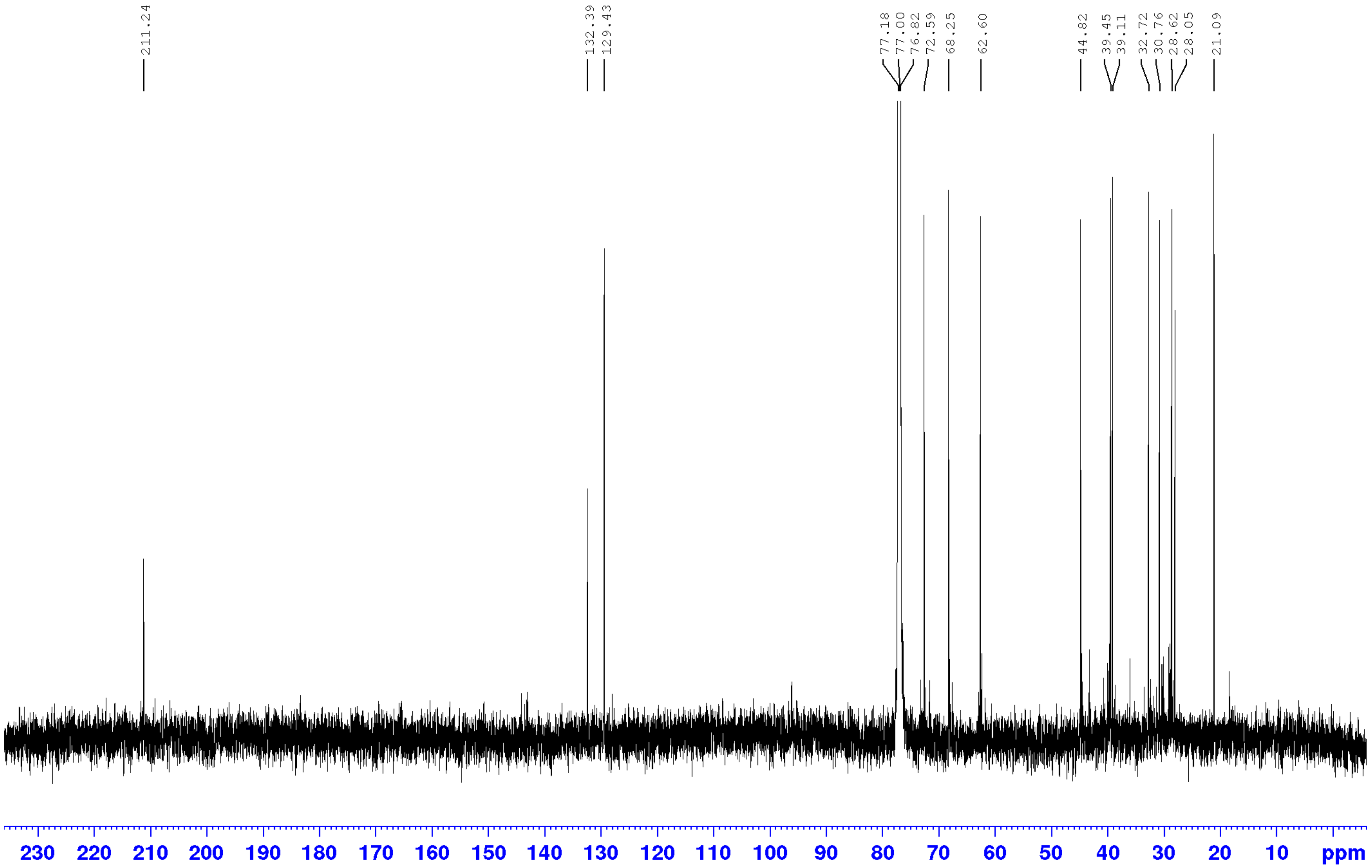

Figure S71. DEPT-135 spectrum of **7** measured at 176 MHz in $CDCl_3$

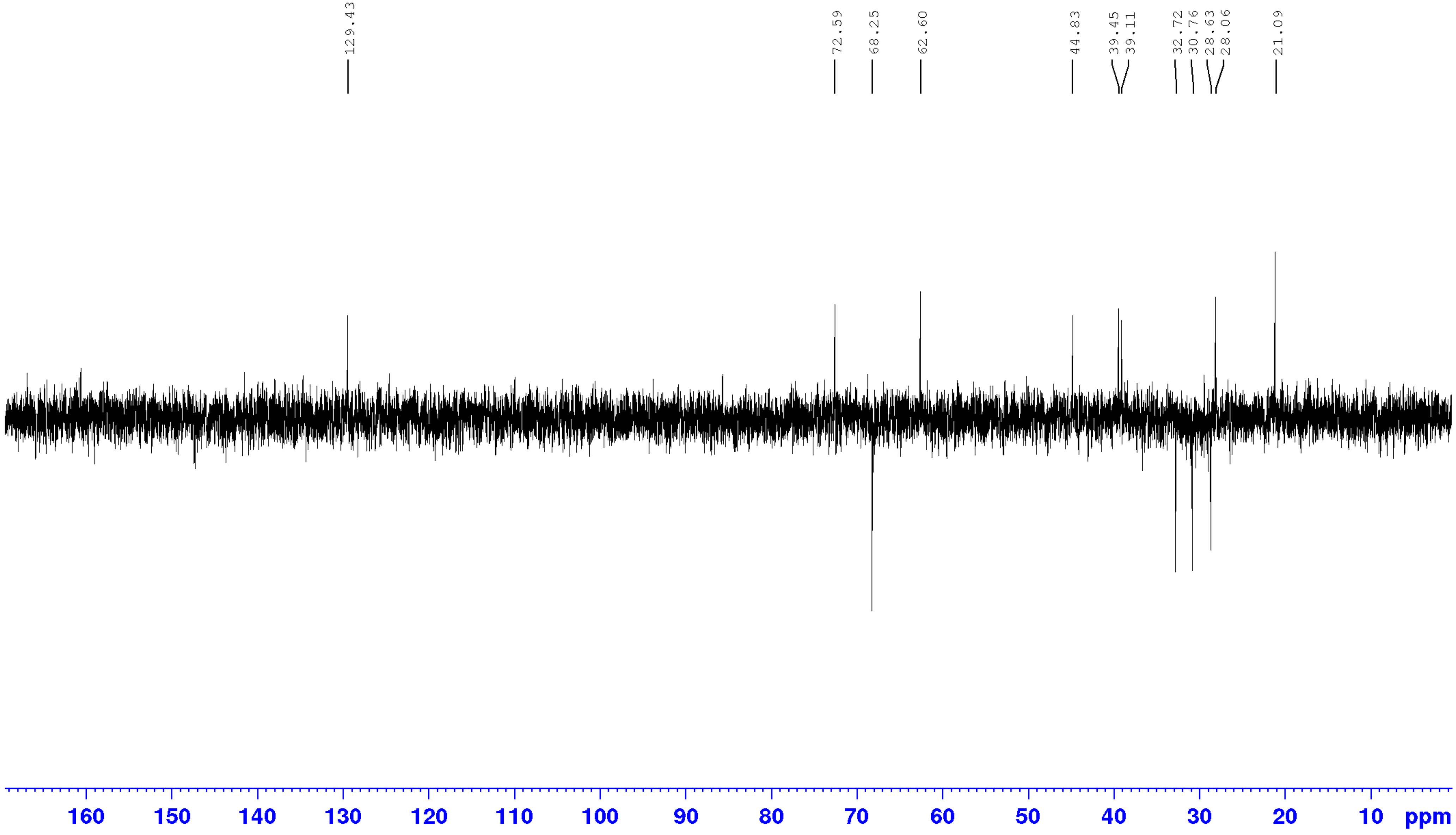

Figure S72. HSQC spectrum of **7** measured in $CDCl_3$

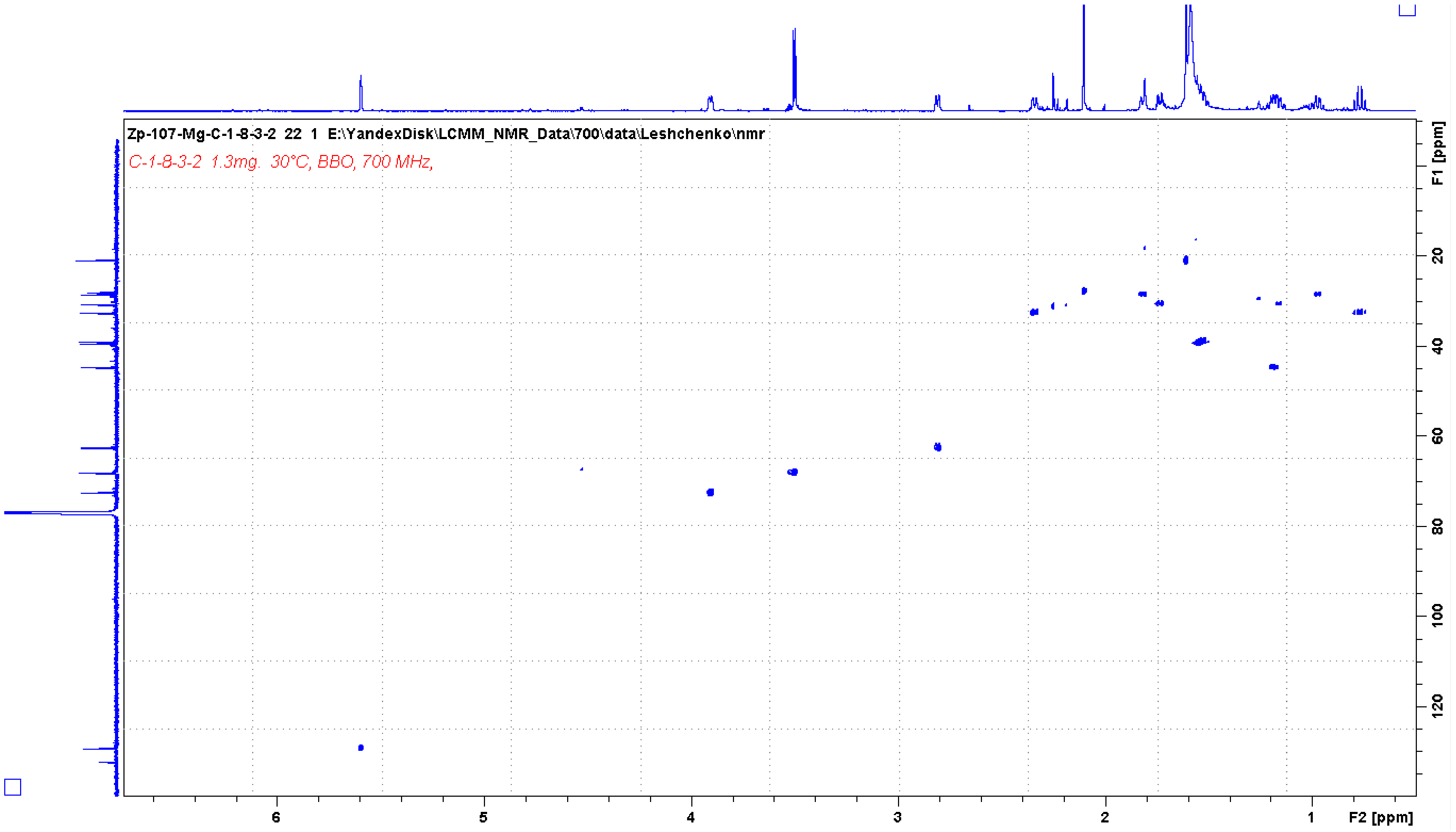

Figure S72. COSY spectrum of **7** measured in $CDCl_3$

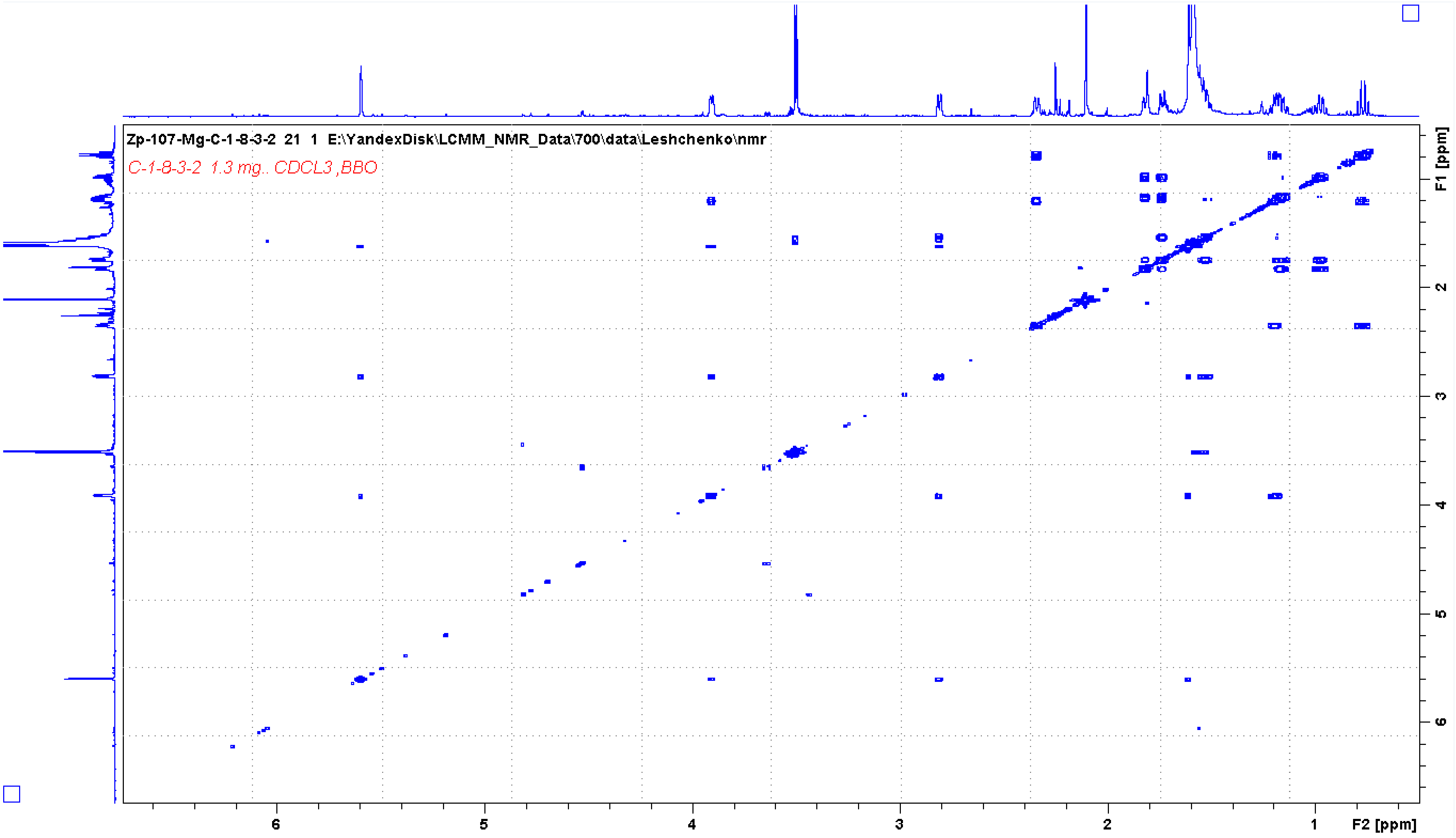

Figure S74. HMBC spectrum of **7** measured in $CDCl_3$

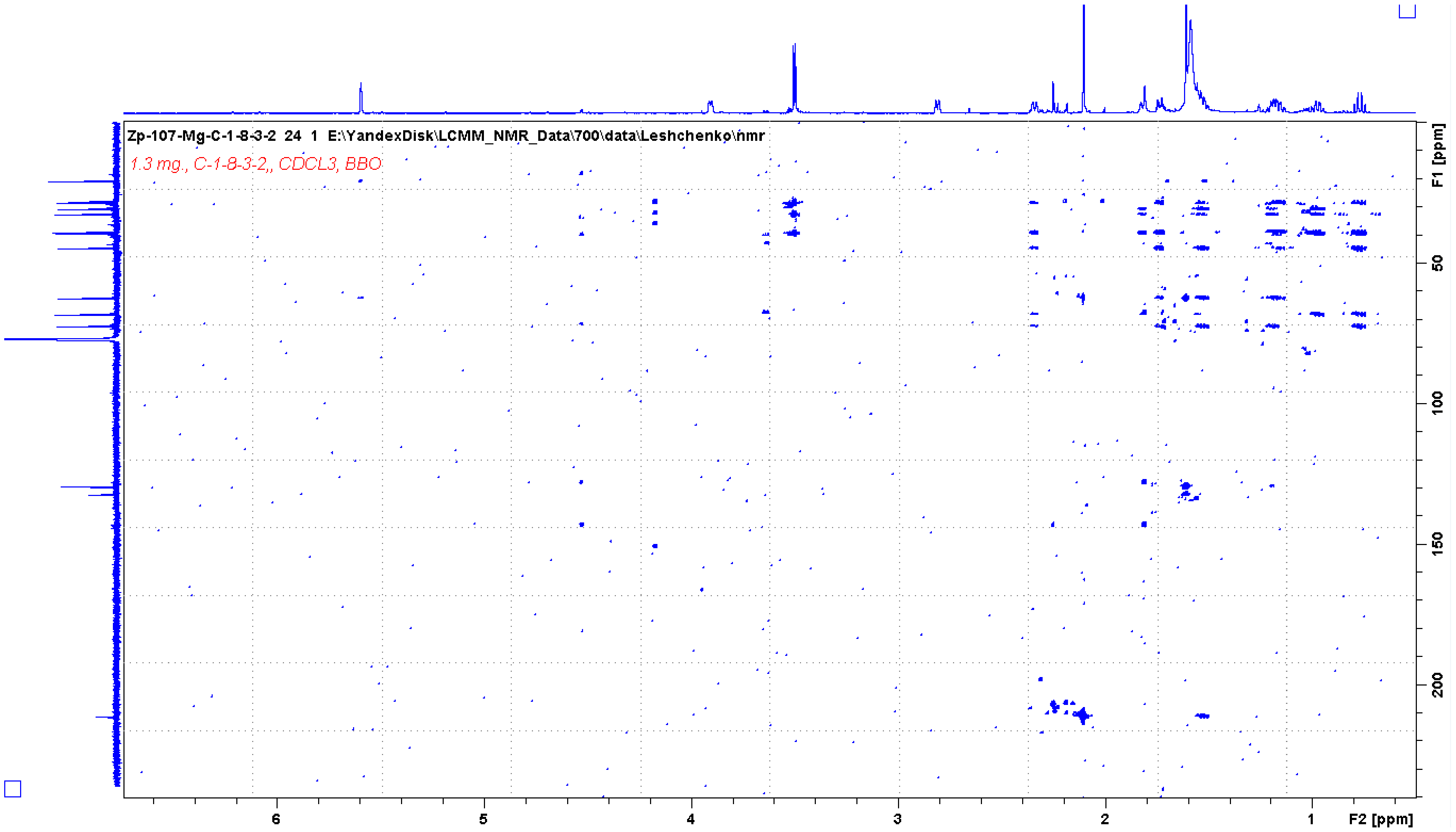

Figure S75. ROESY spectrum of **7** measured in $CDCl_3$

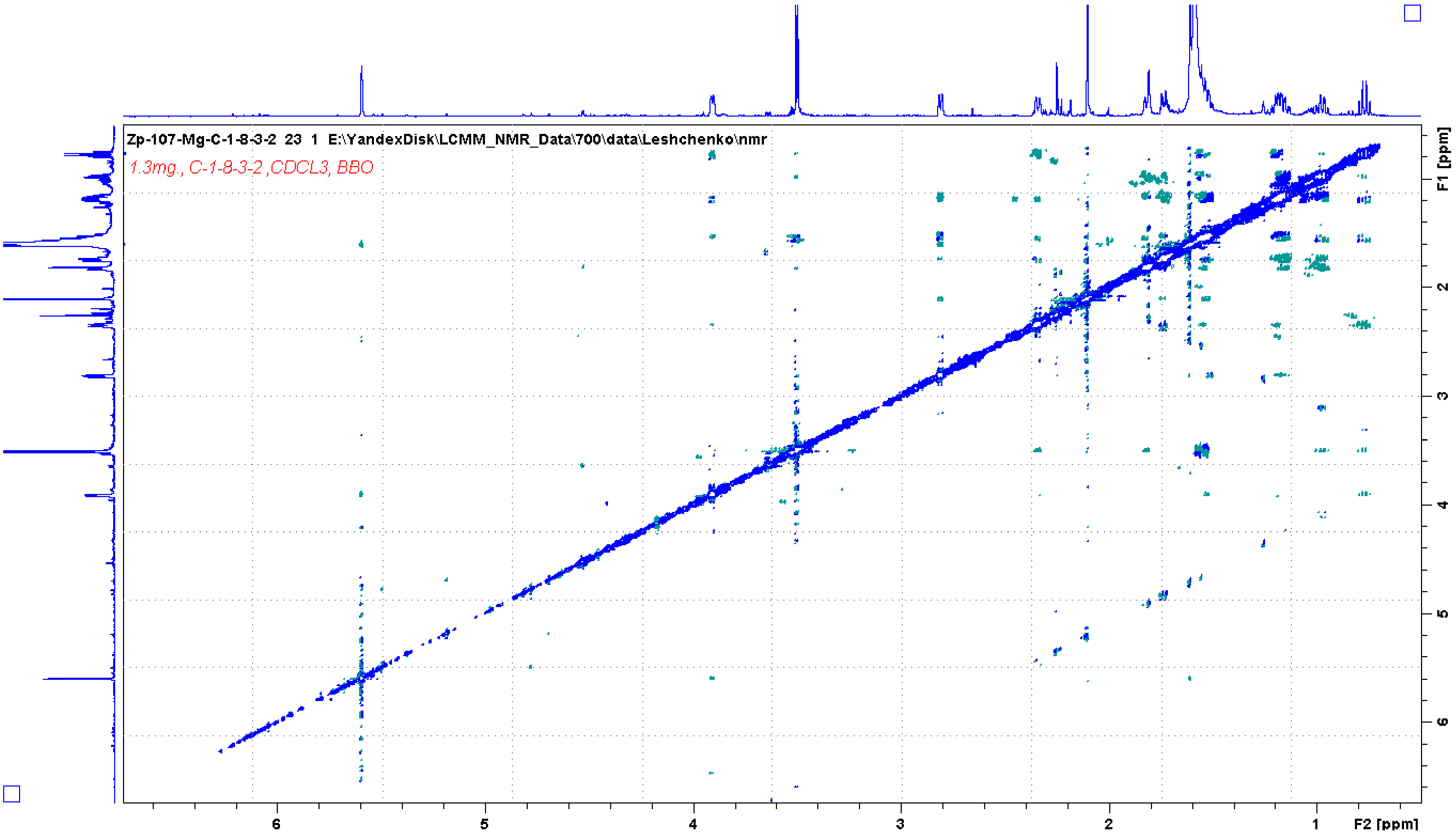

Figure S76. (**A**) Key COSY, (**B**) HMBC and (**C**) ROESY correlations of **8**

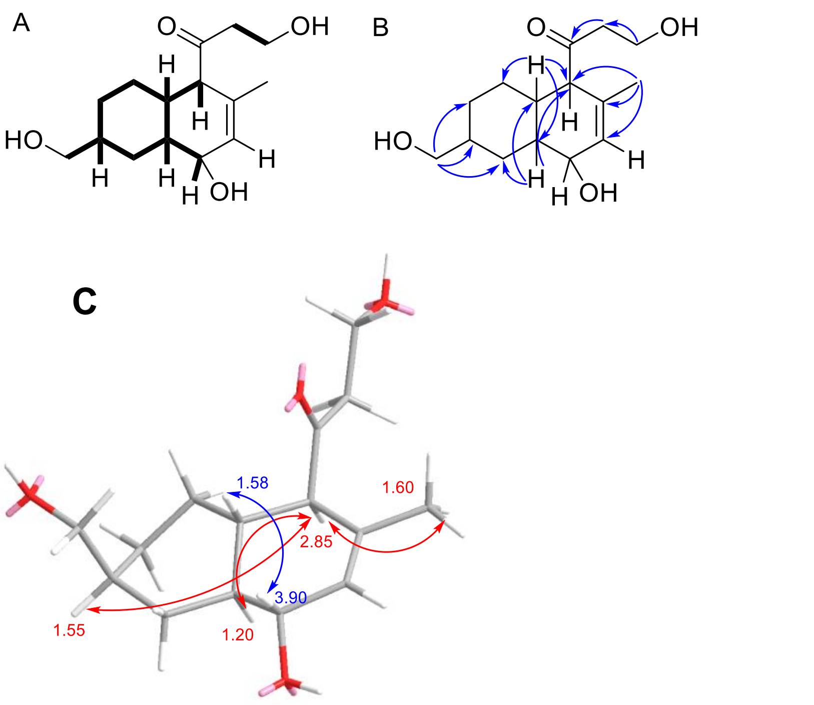

Figure S77. HRESIMS for **8**

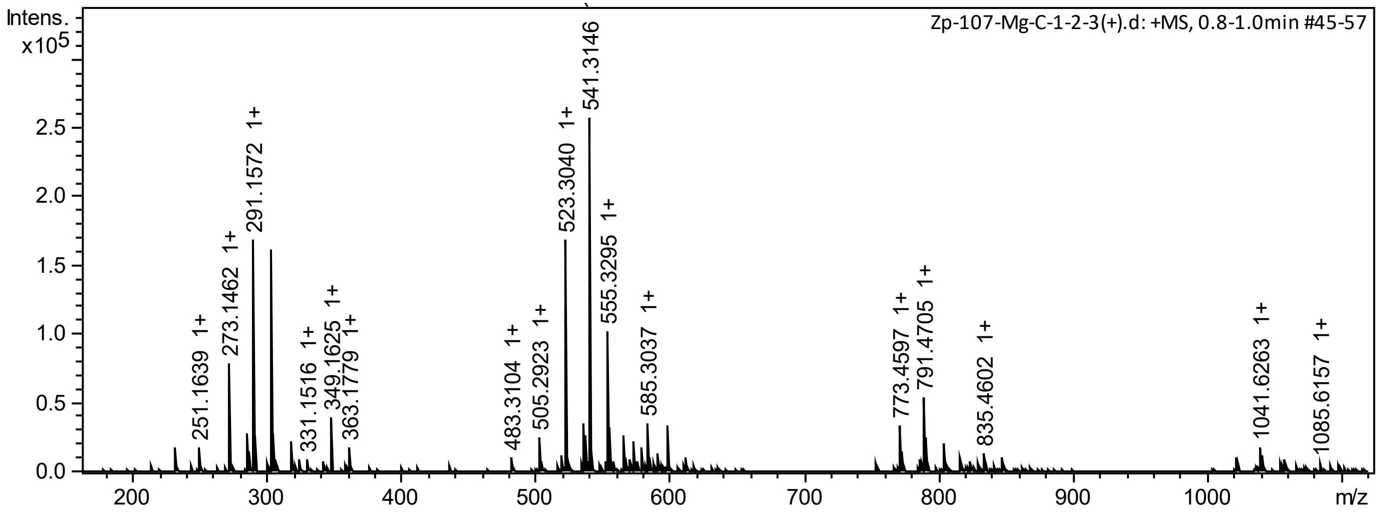

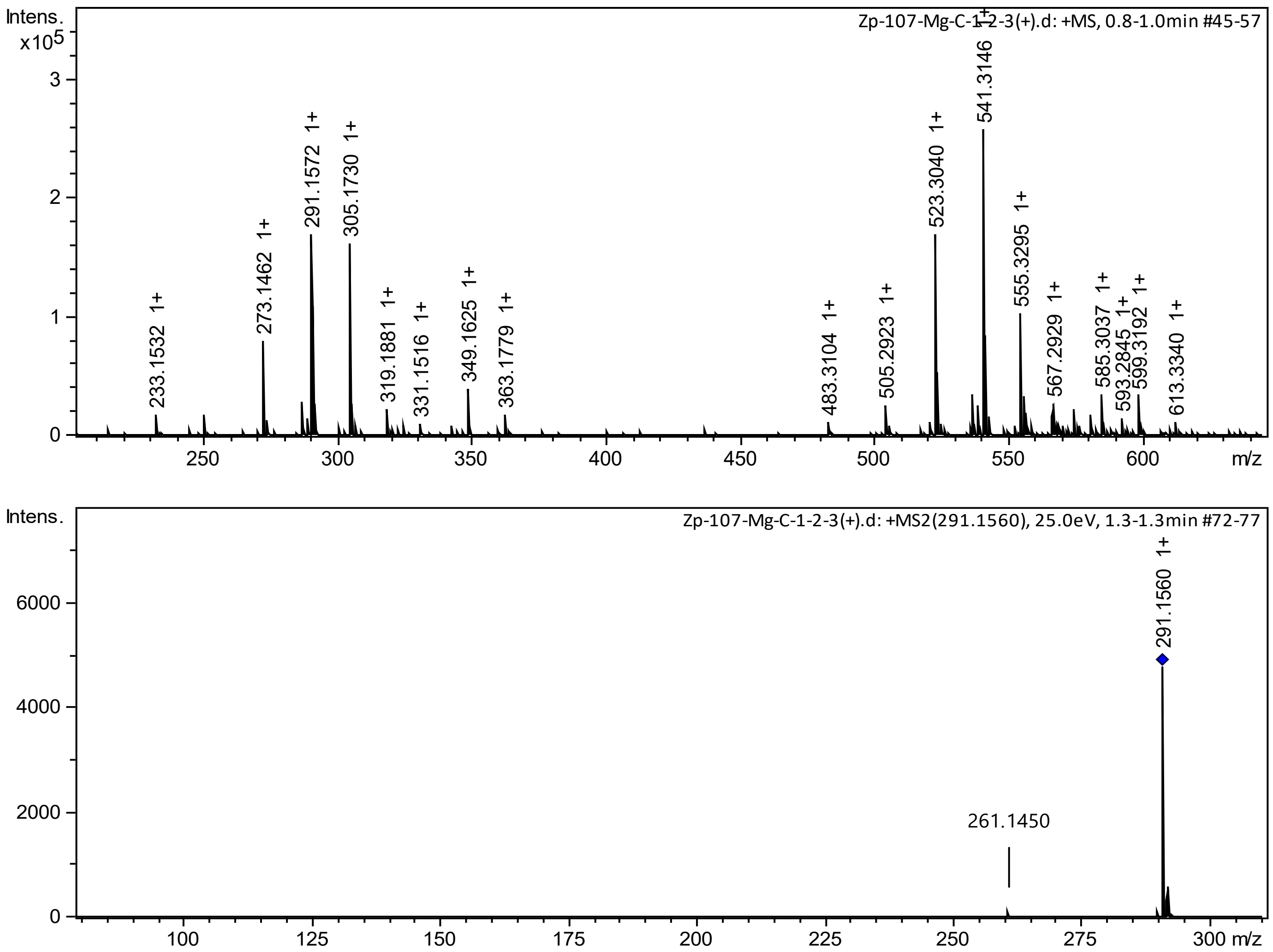
Zp-107-Mg-C-1-2-3(+).d: +MS, 0.8-1.0min #45-57
Intens. x10^5
233.1532 1+
273.1462 1+
291.1572 1+
305.1730 1+
319.1881 1+
331.1516 1+
349.1625 1+
363.1779 1+
483.3104 1+
505.2923 1+
523.3040 1+
541.3146 1+
555.3295 1+
567.2929 1+
585.3037 1+
593.2845 1+
599.3192 1+
613.3340 1+
m/z
Zp-107-Mg-C-1-2-3(+).d: +MS2(291.1560), 25.0eV, 1.3-1.3min #72-77
Intens.
291.1560 1+
261.1450
m/z

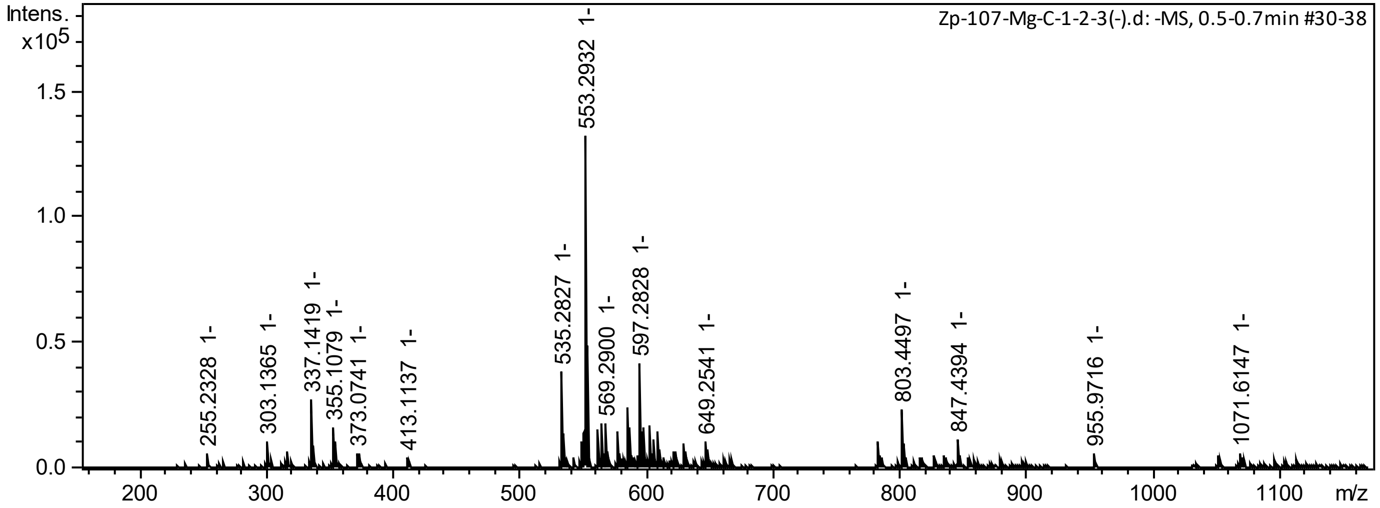

	meas	calc	Δ (ppm)
$[M-H]^-$	267,1596	267,1602	2,2
$[M+Cl]^-$	303,1365	303,1369	1,3
$[M+Na]^+$	291,1572	291,1567	-1,7

Figure S78. ^{1}H NMR spectrum of **8** measured at 700 MHz in $CDCl_3$

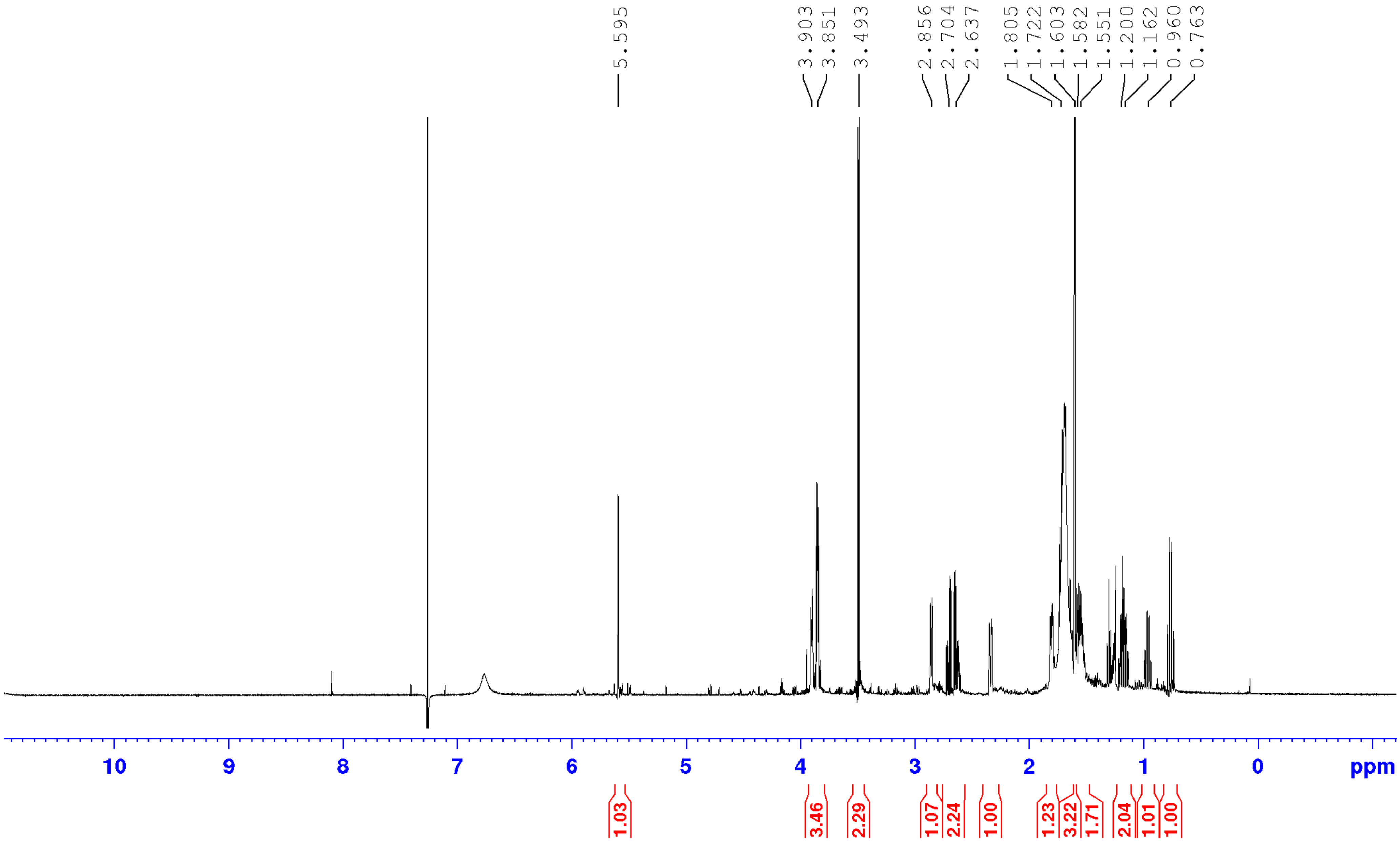

Figure S79. ^{13}C NMR spectrum of **8** measured at 176 MHz in $CDCl_3$

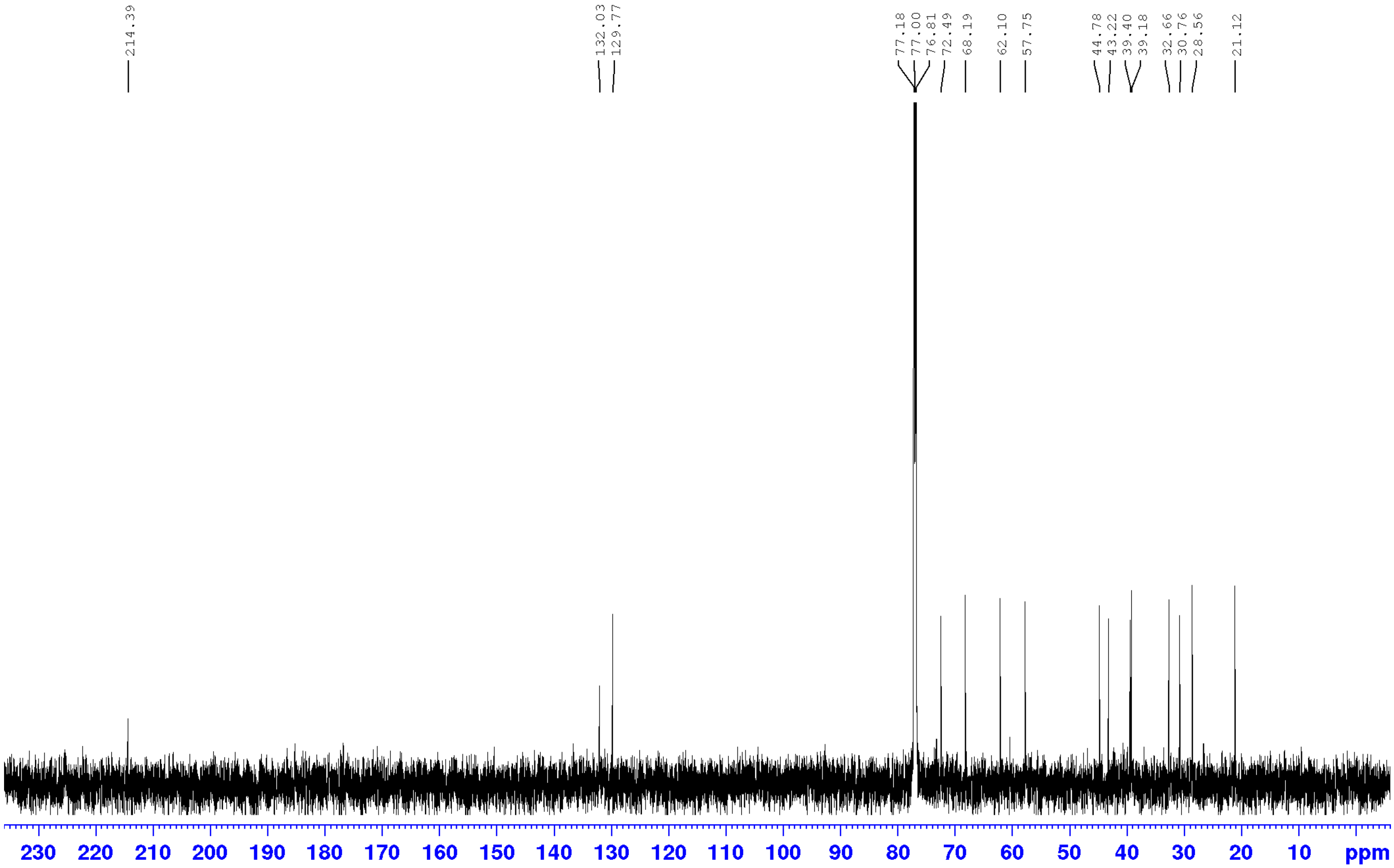

Figure S80. DEPT-135 spectrum of **8** measured at 176 MHz in $CDCl_3$

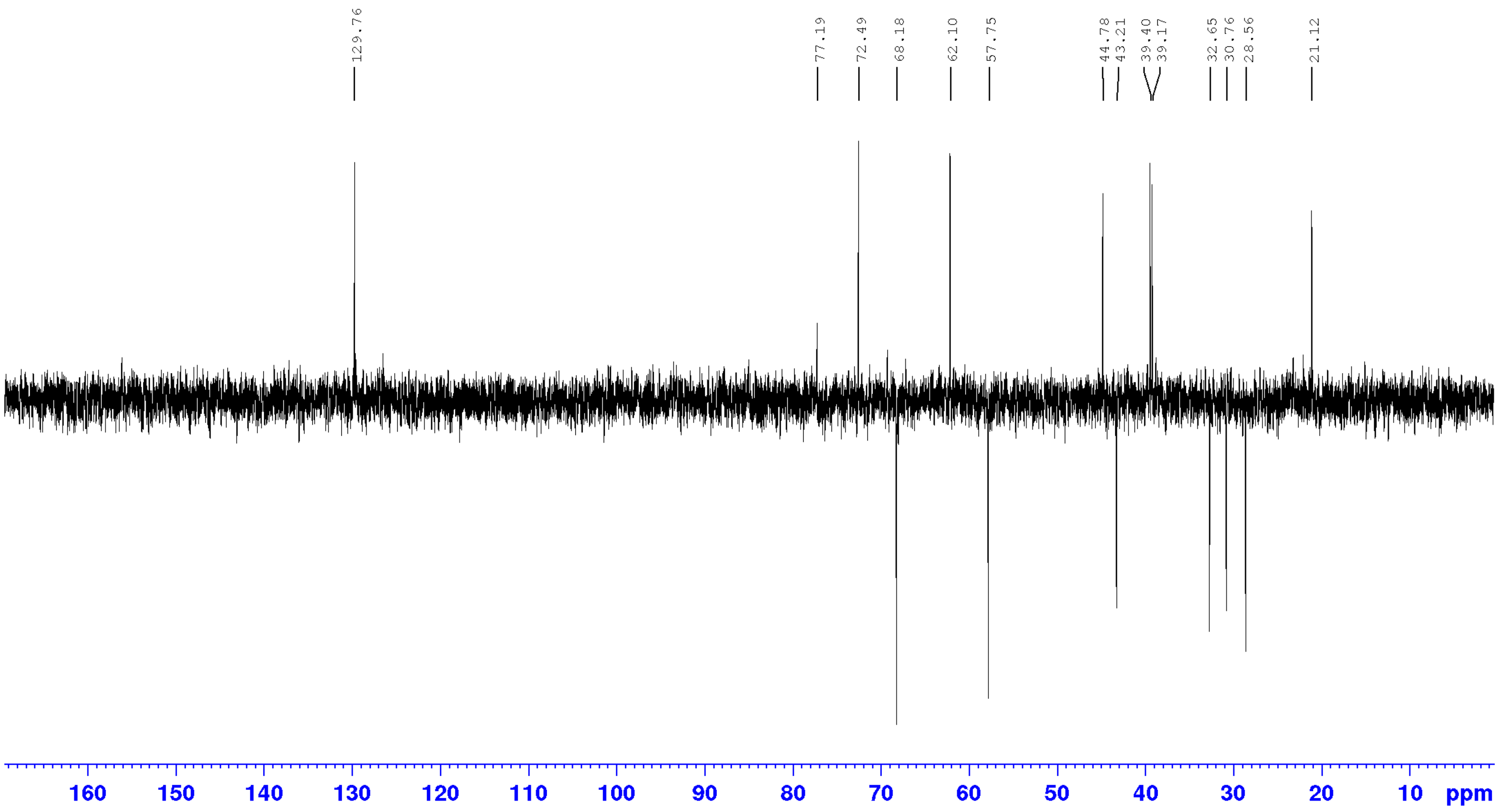

Figure S81. HSQC spectrum of **8** measured in $CDCl_3$

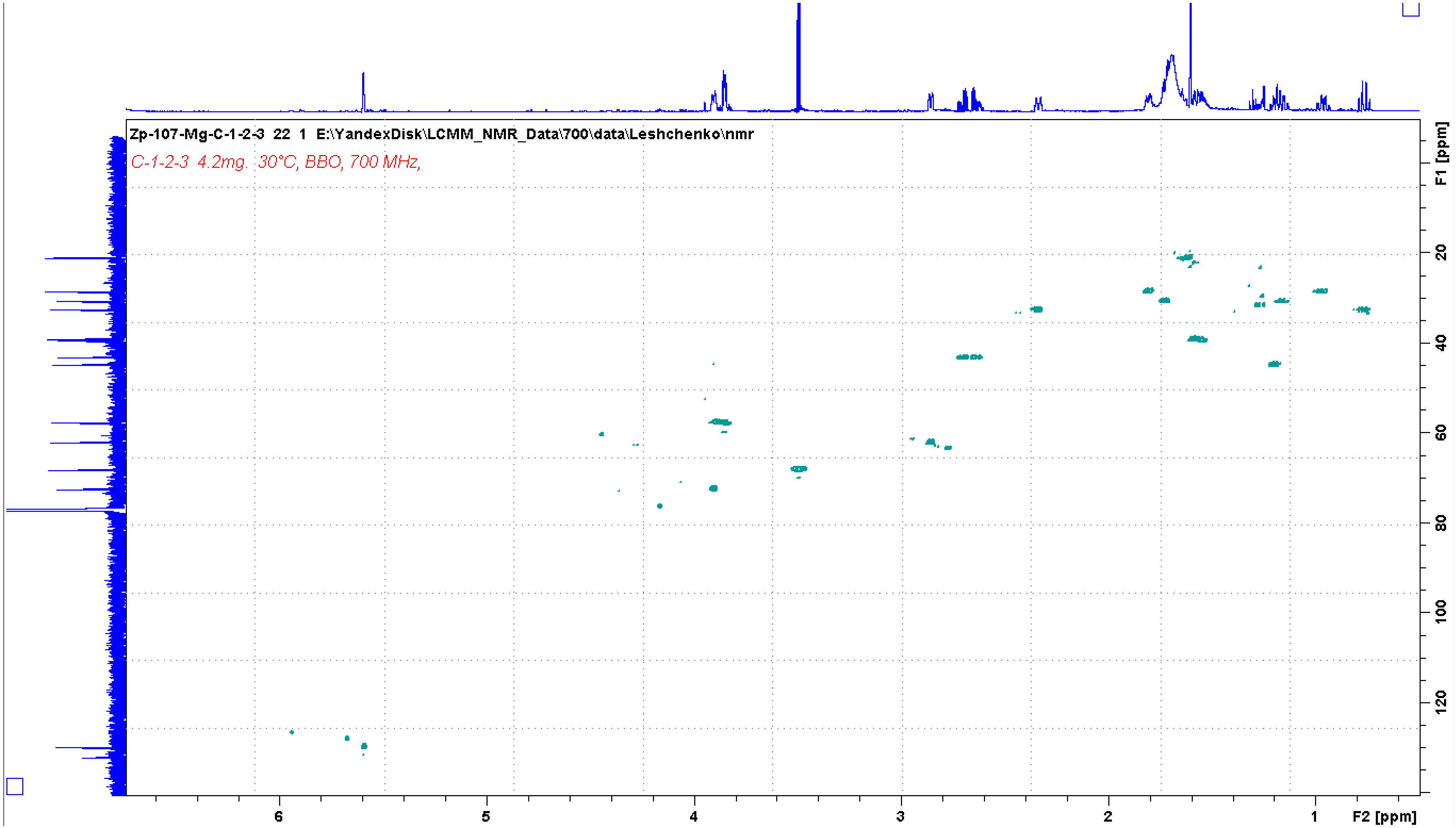

Figure S82. COSY spectrum of **8** measured in $CDCl_3$

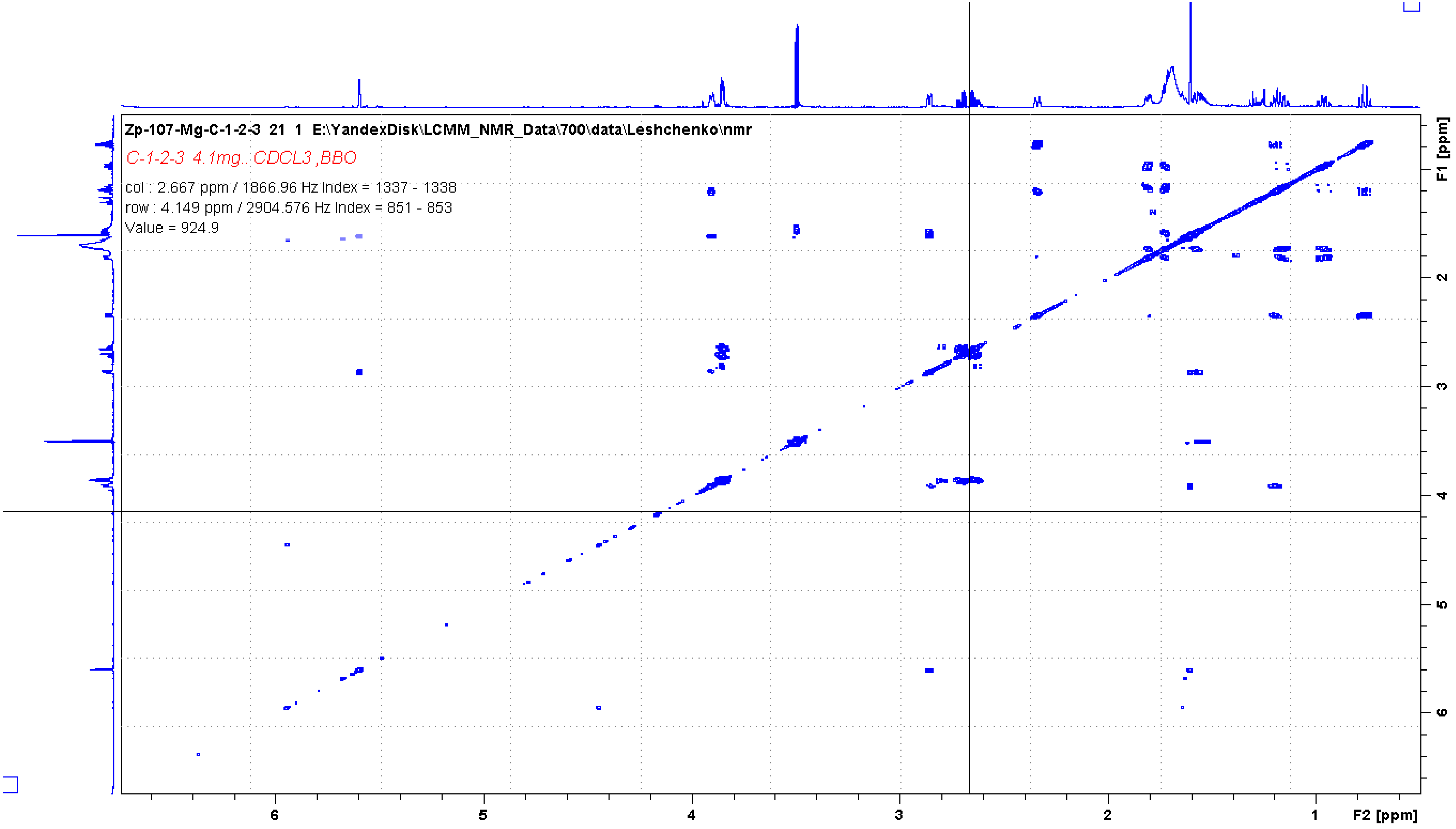

Figure S83. HMBC spectrum of **8** measured in $CDCl_3$

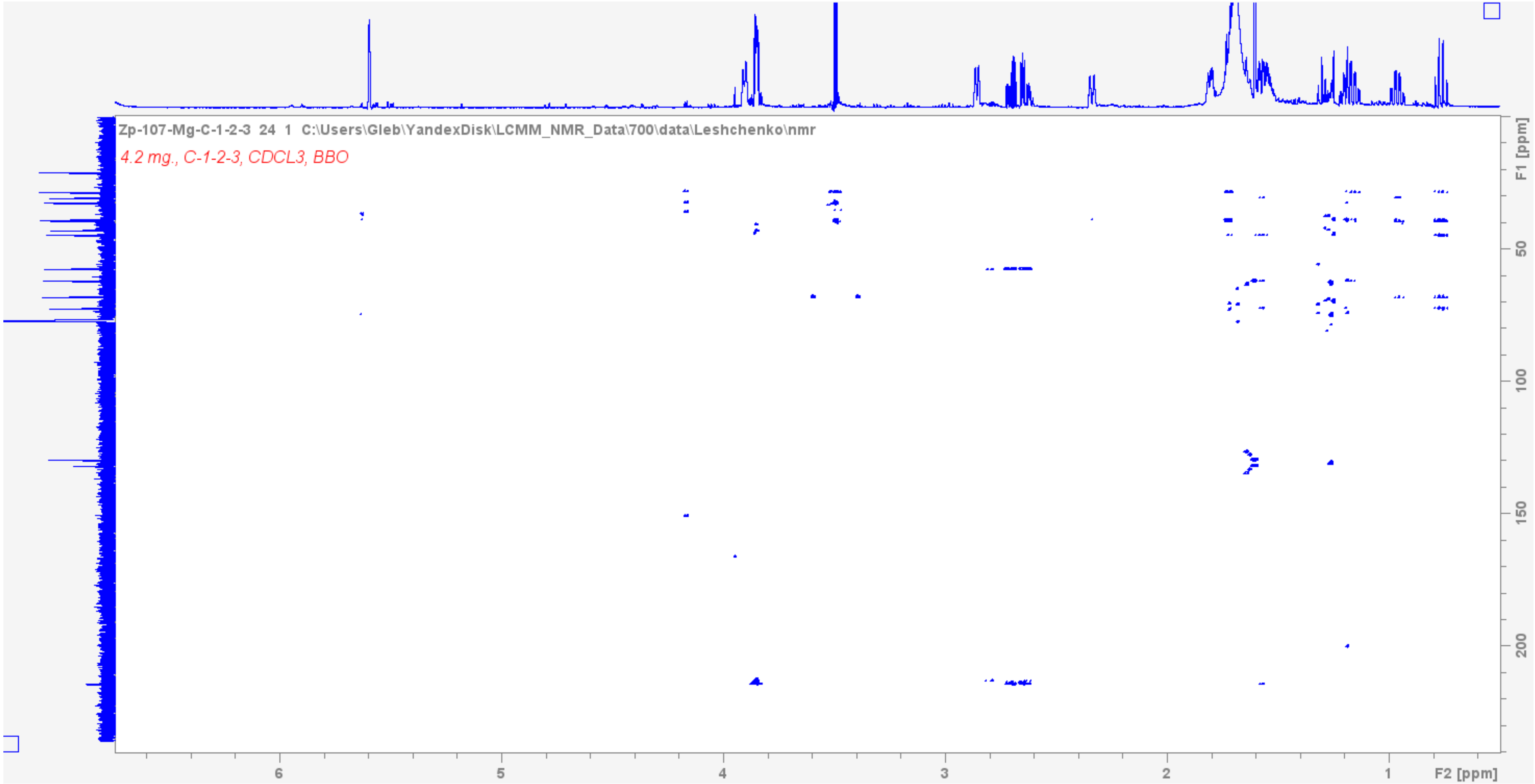

Figure S84. ROESY spectrum of **8** measured in $CDCl_3$

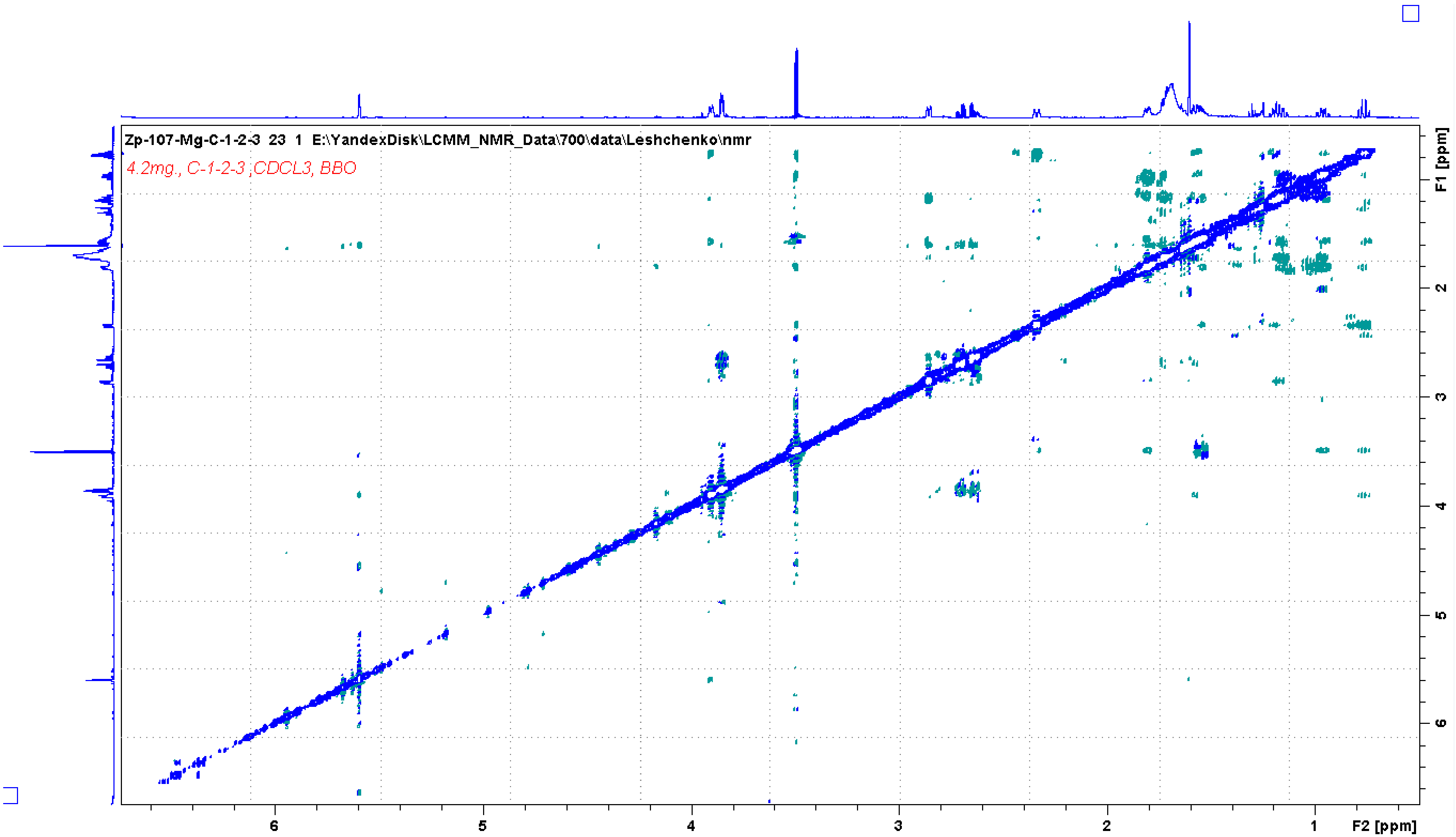

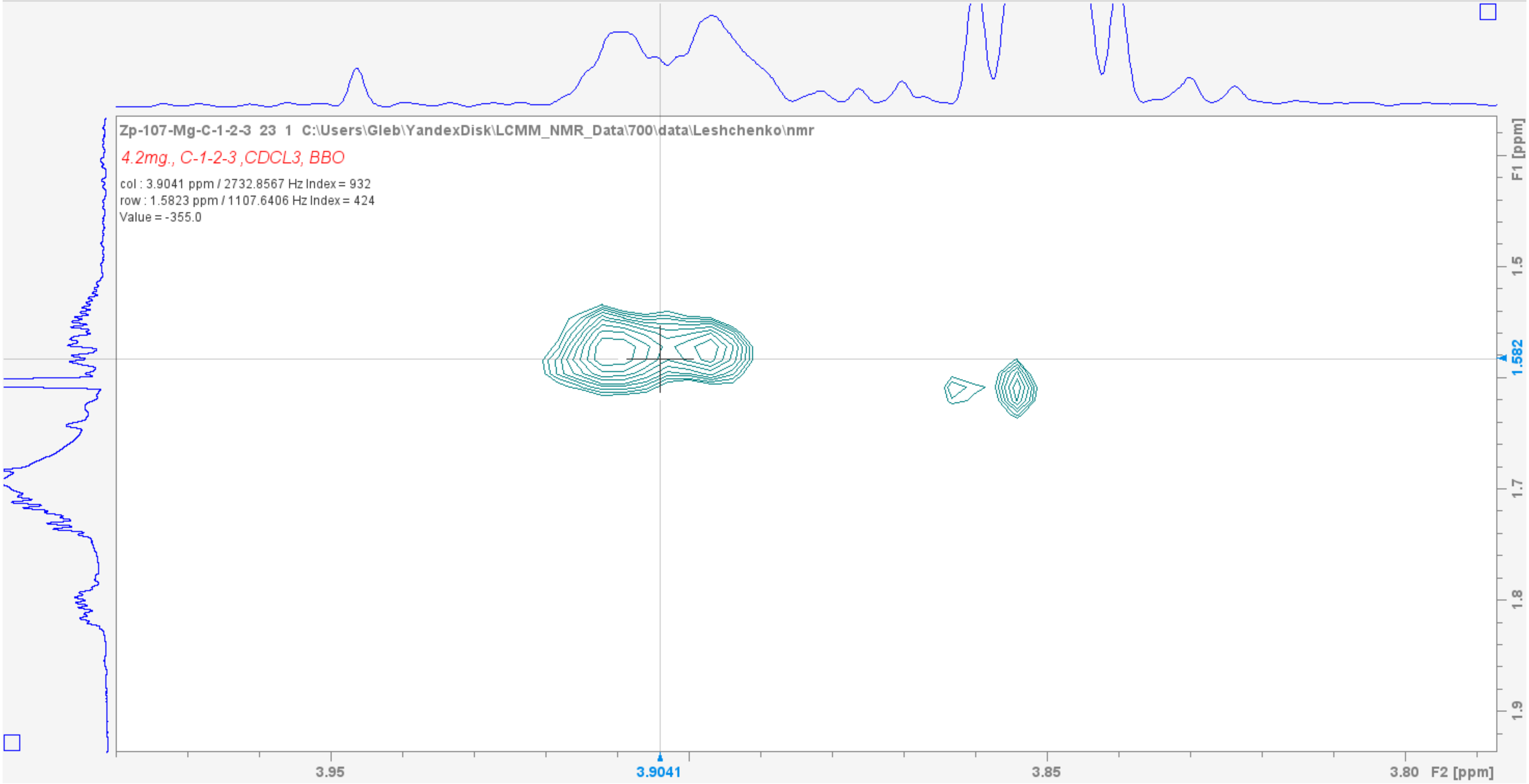

Zp-107-Mg-C-1-2-3 23 1 C:\Users\Gleb\YandexDisk\LCMM_NMR_Data\700\data\Leshchenko\nmr
4.2mg., C-1-2-3 ,CDCL3, BBO
col : 3.9041 ppm / 2732.8567 Hz Index = 932
row : 1.5823 ppm / 1107.6406 Hz Index = 424
Value = -355.0
F1 [ppm]
1.5
1.582
1.7
1.8
1.9
3.95
3.9041
3.85
3.80
F2 [ppm]

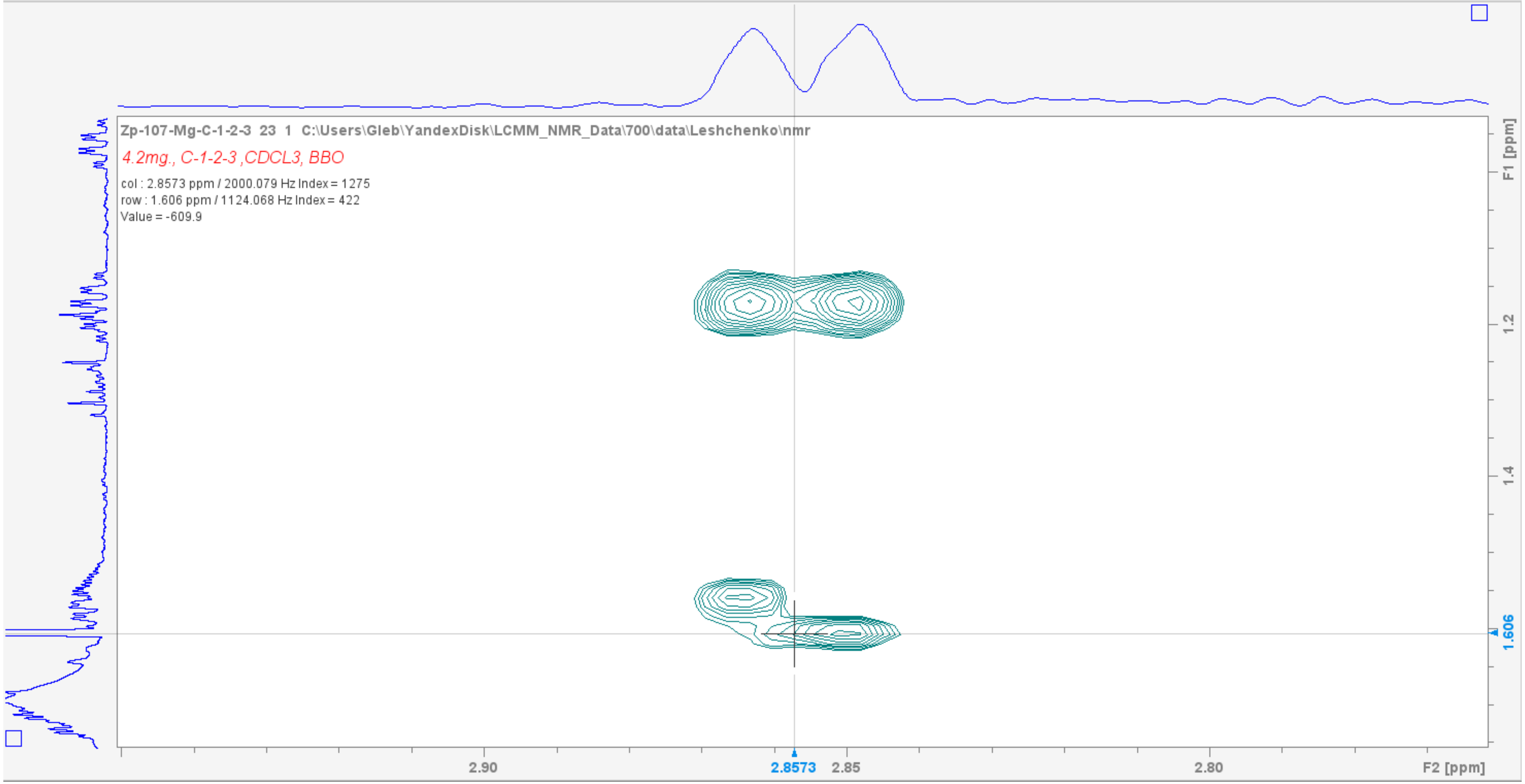

Zp-107-Mg-C-1-2-3 23 1 C:\Users\Gleb\YandexDisk\LCMM_NMR_Data\700\data\Leshchenko\nmr
4.2mg., C-1-2-3 ,CDCL3, BBO
col : 2.8573 ppm / 2000.079 Hz Index = 1275
row : 1.606 ppm / 1124.068 Hz Index = 422
Value = -609.9
F1 [ppm]
1.2
1.4
1.606
2.90
2.8573
2.85
2.80
F2 [ppm]

Figure S85. (A) Key COSY and (B) HMBC correlations of **9**

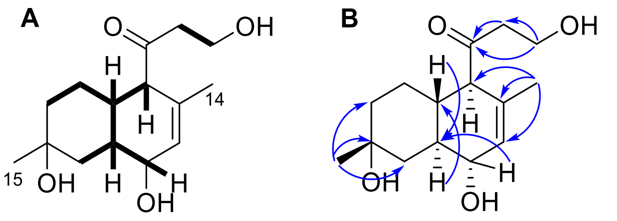

Figure S86. HRESIMS for **9**

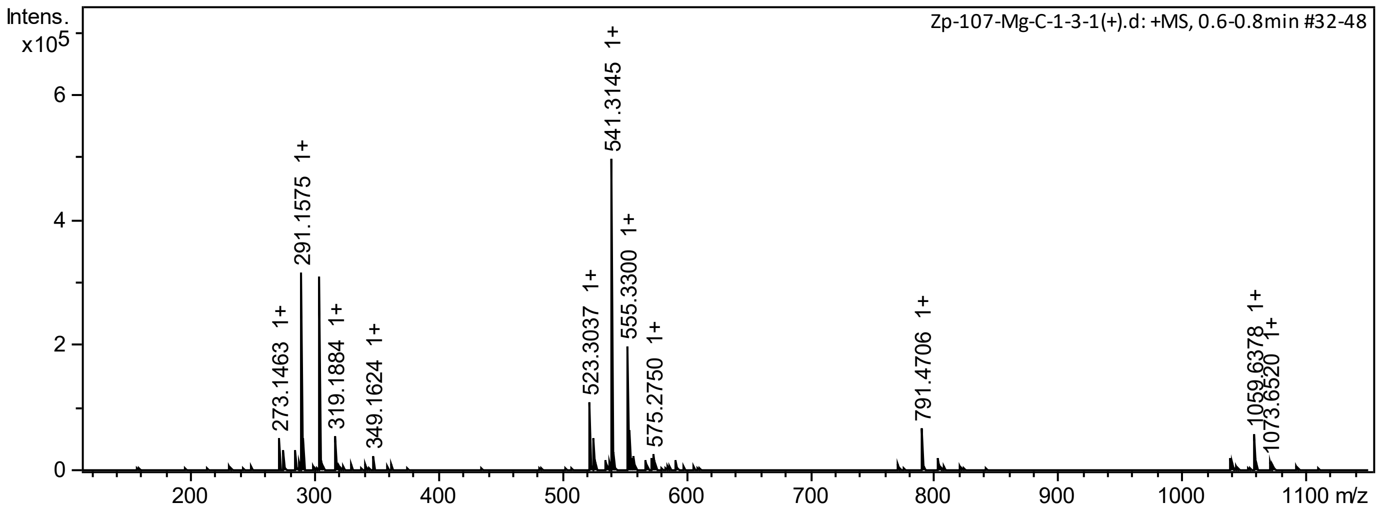

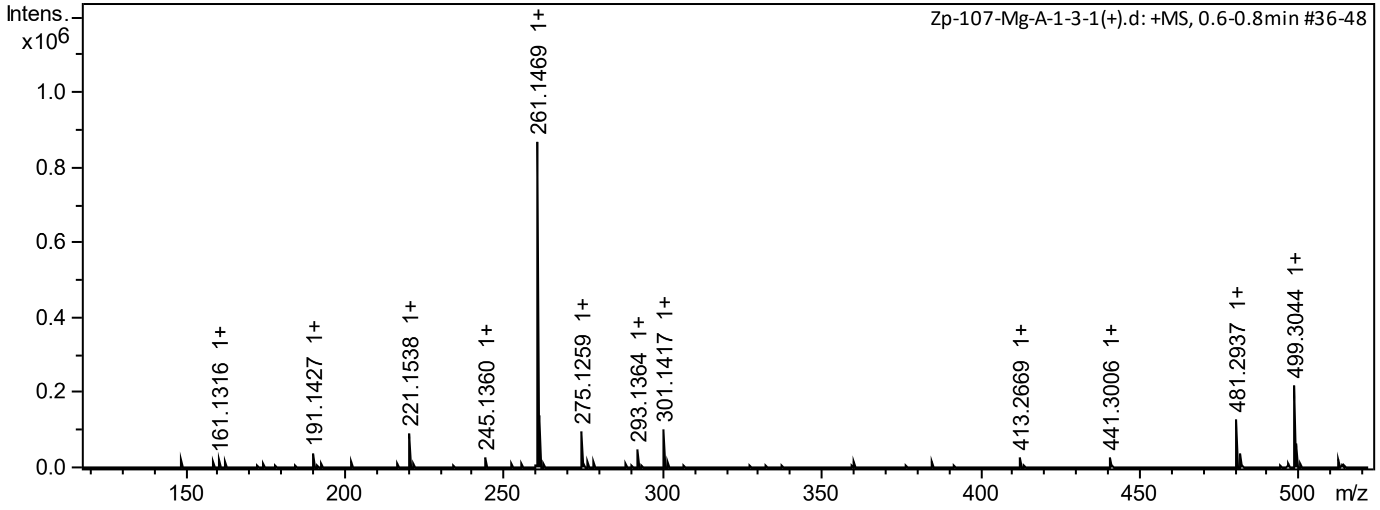

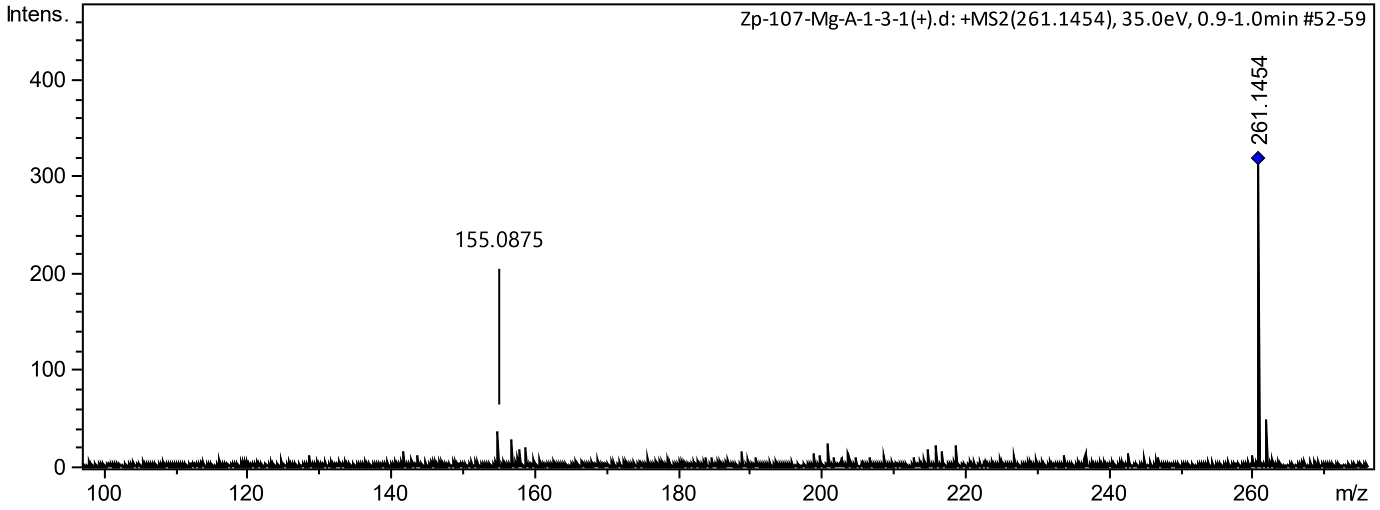

	meas	calc	Δ (ppm)
$[M-H]^-$	237,1493	237,1496	1,3
$[M+Na]^+$	261,1469	261,1461	-3,1

Figure S87. ^{1}H NMR spectrum of **9** measured at 700 MHz in $CDCl_3$

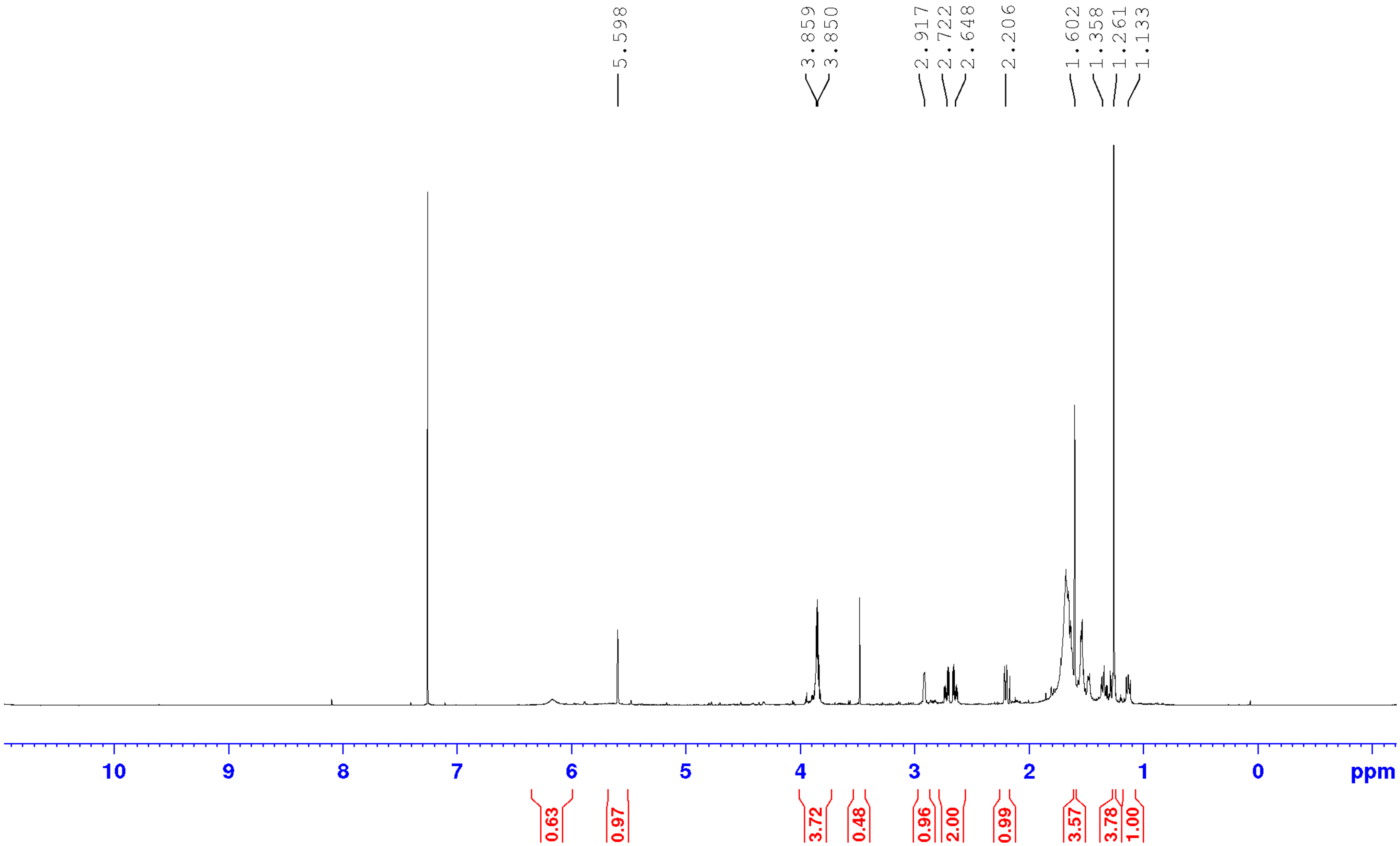

Figure S88. ^{13}C NMR spectrum of **9** measured at 176 MHz in $CDCl_3$

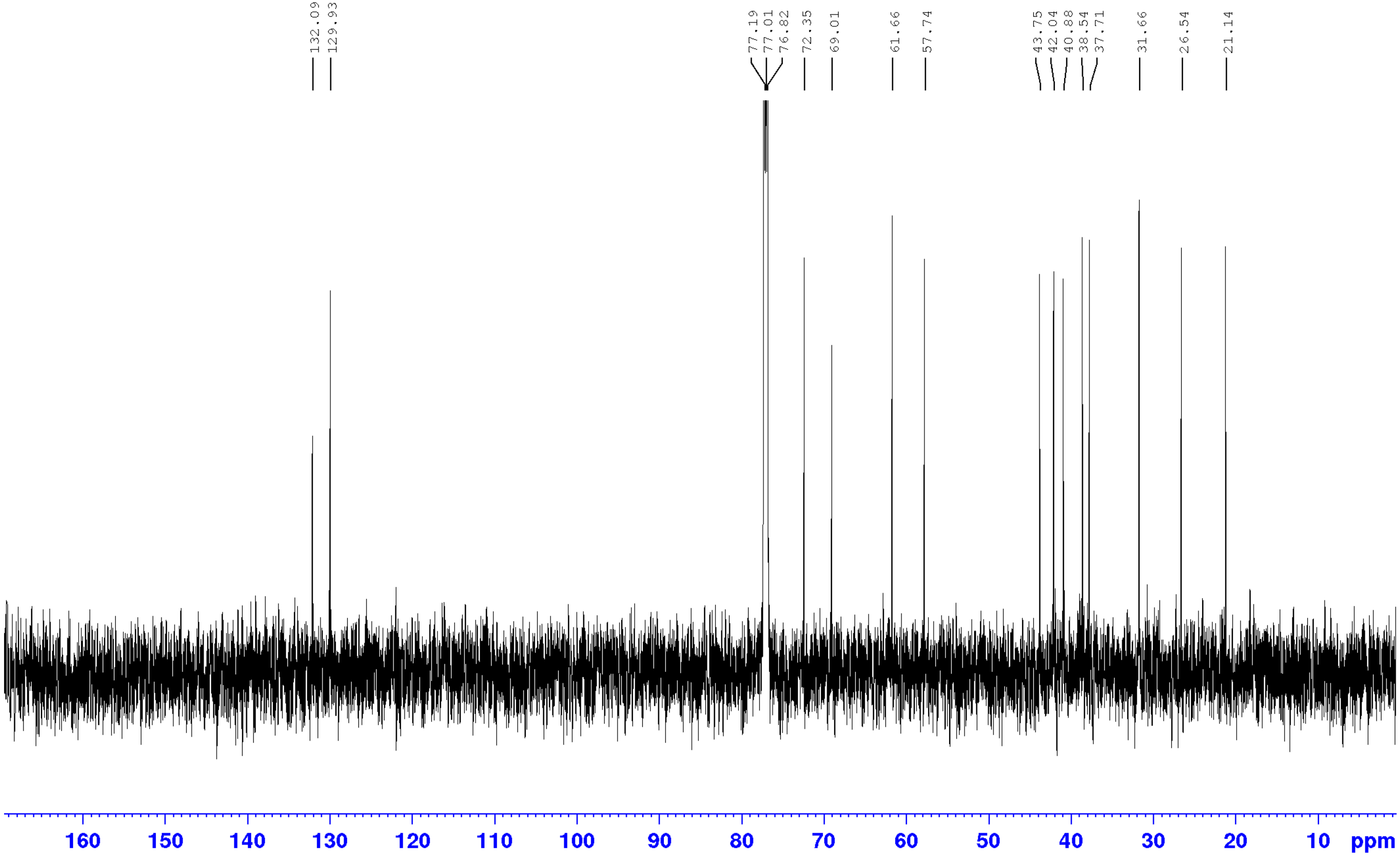

Figure S89. DEPT-135 spectrum of **9** measured at 176 MHz in $CDCl_3$

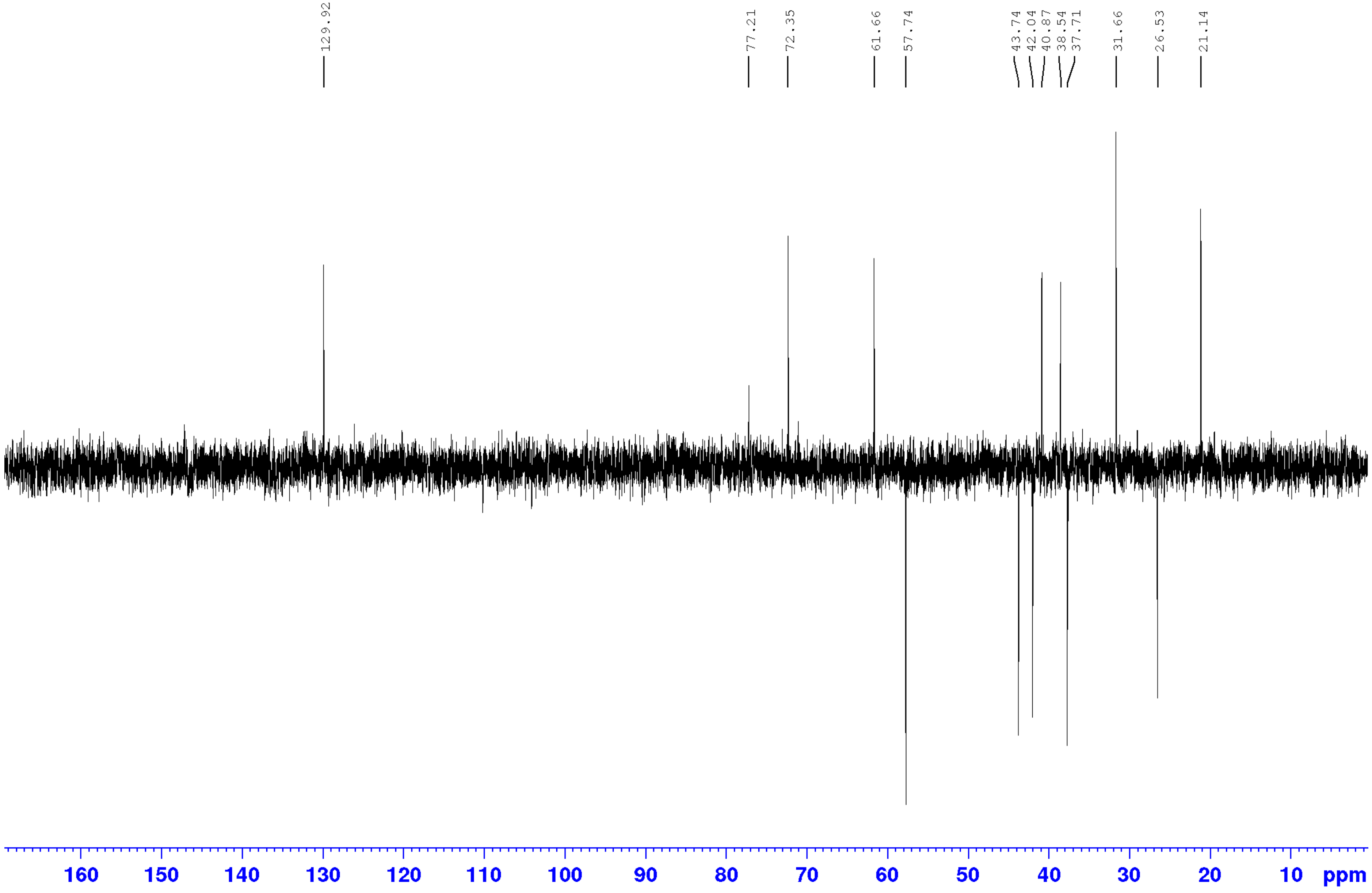

Figure S90. HSQC spectrum of **9** measured in $CDCl_3$

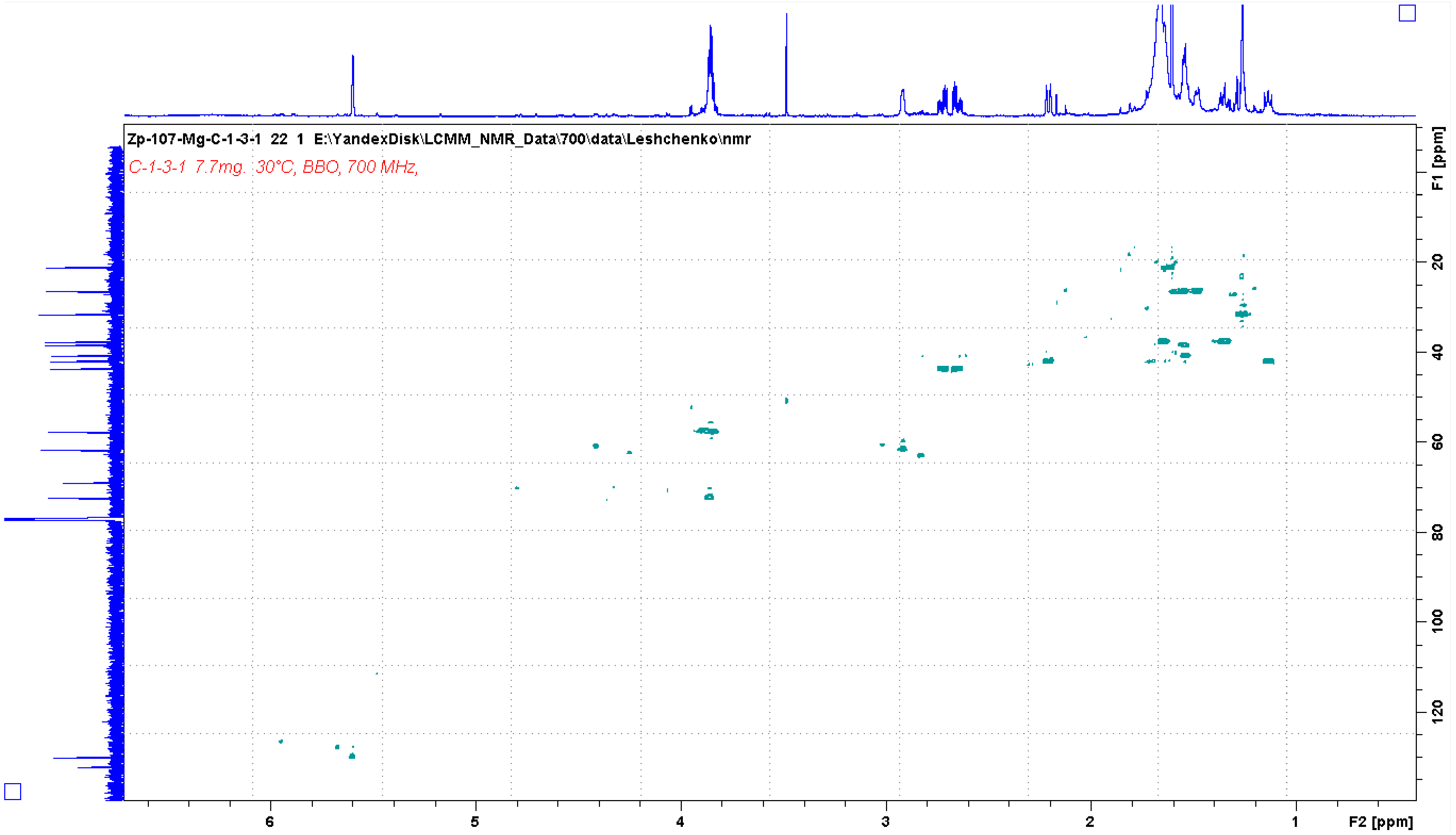

Figure S91. COSY spectrum of **9** measured in $CDCl_3$

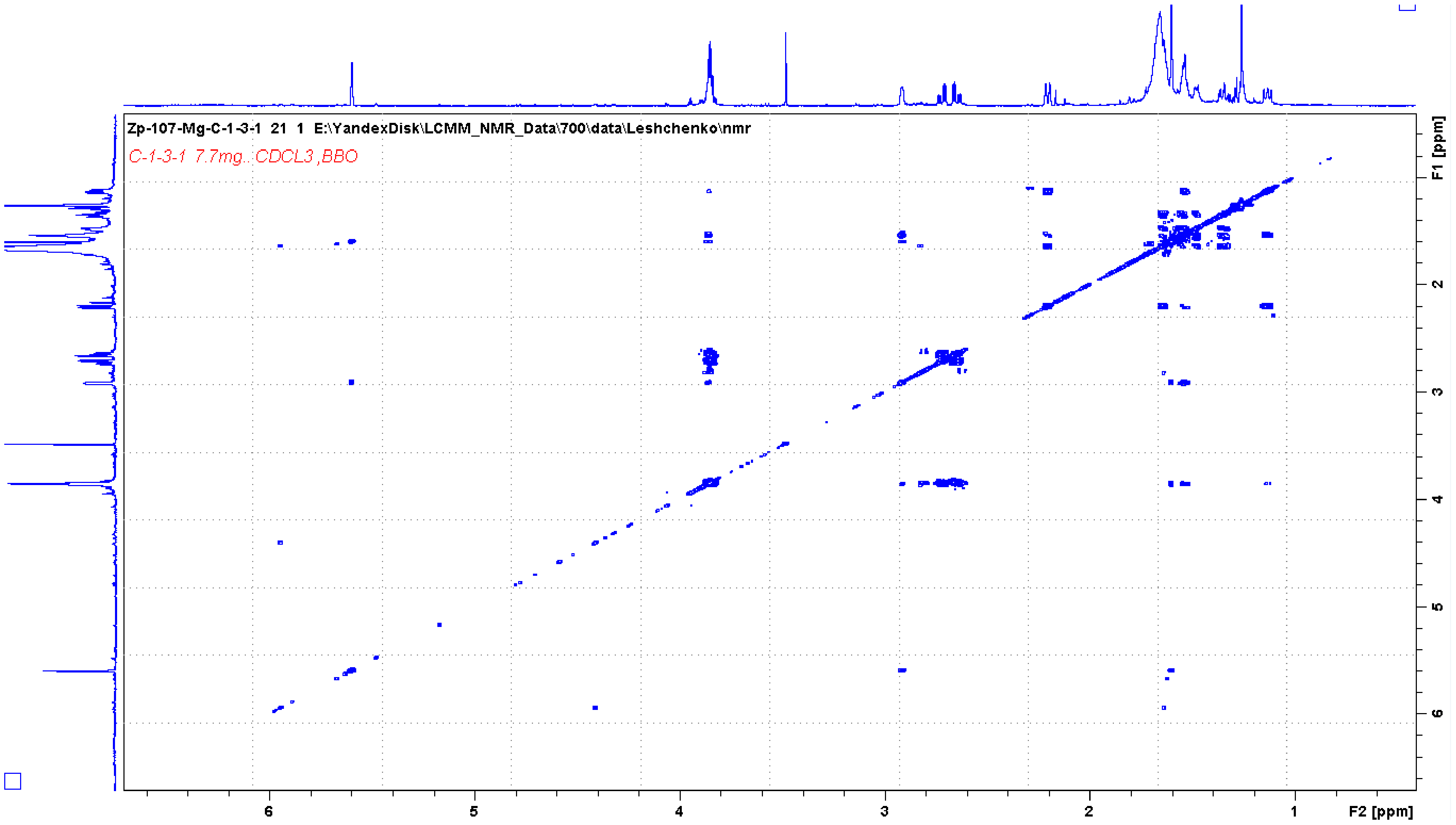

Figure S92. HMBC spectrum of **9** measured in $CDCl_3$

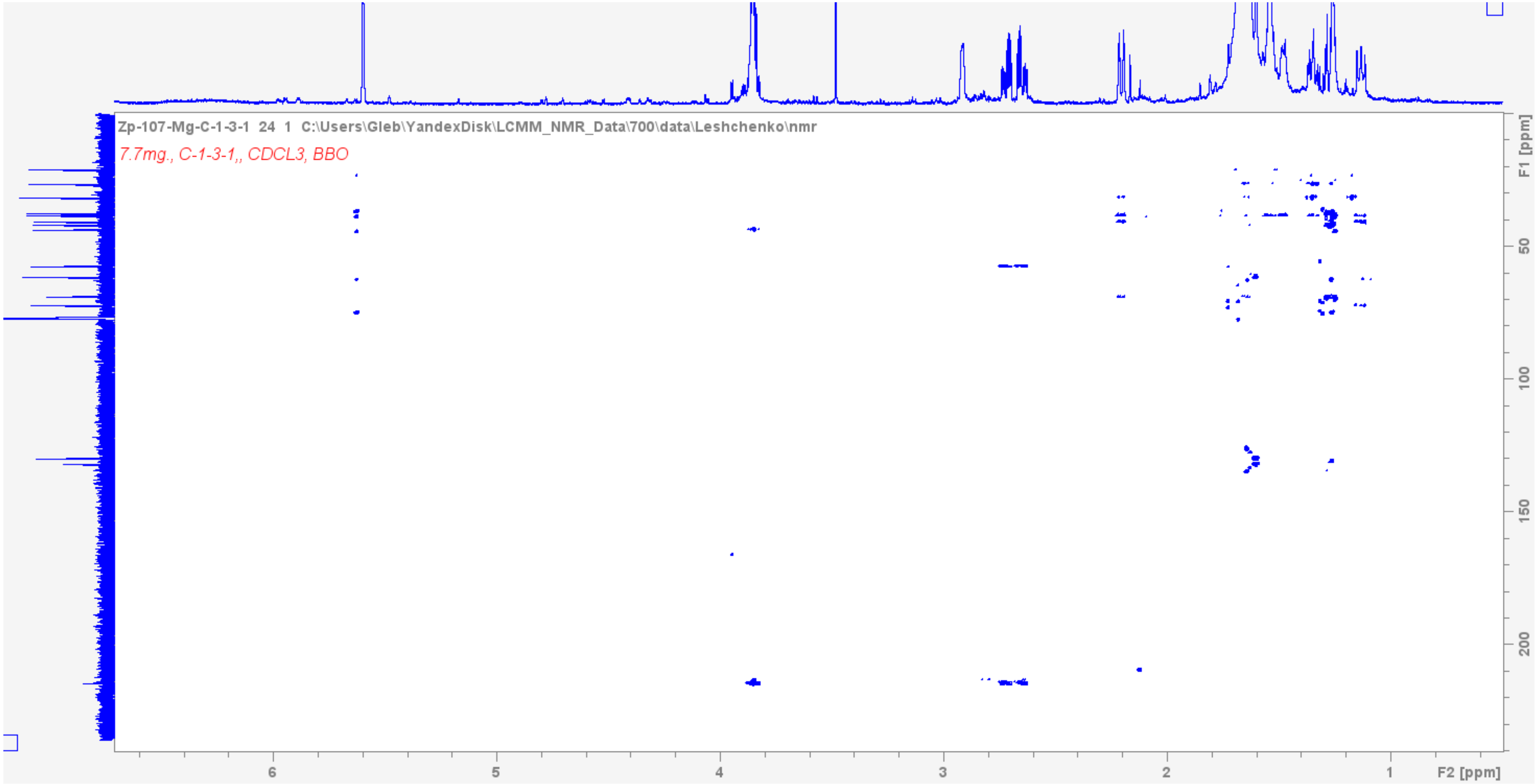

Figure S93. ROESY spectrum of **9** measured in $CDCl_3$

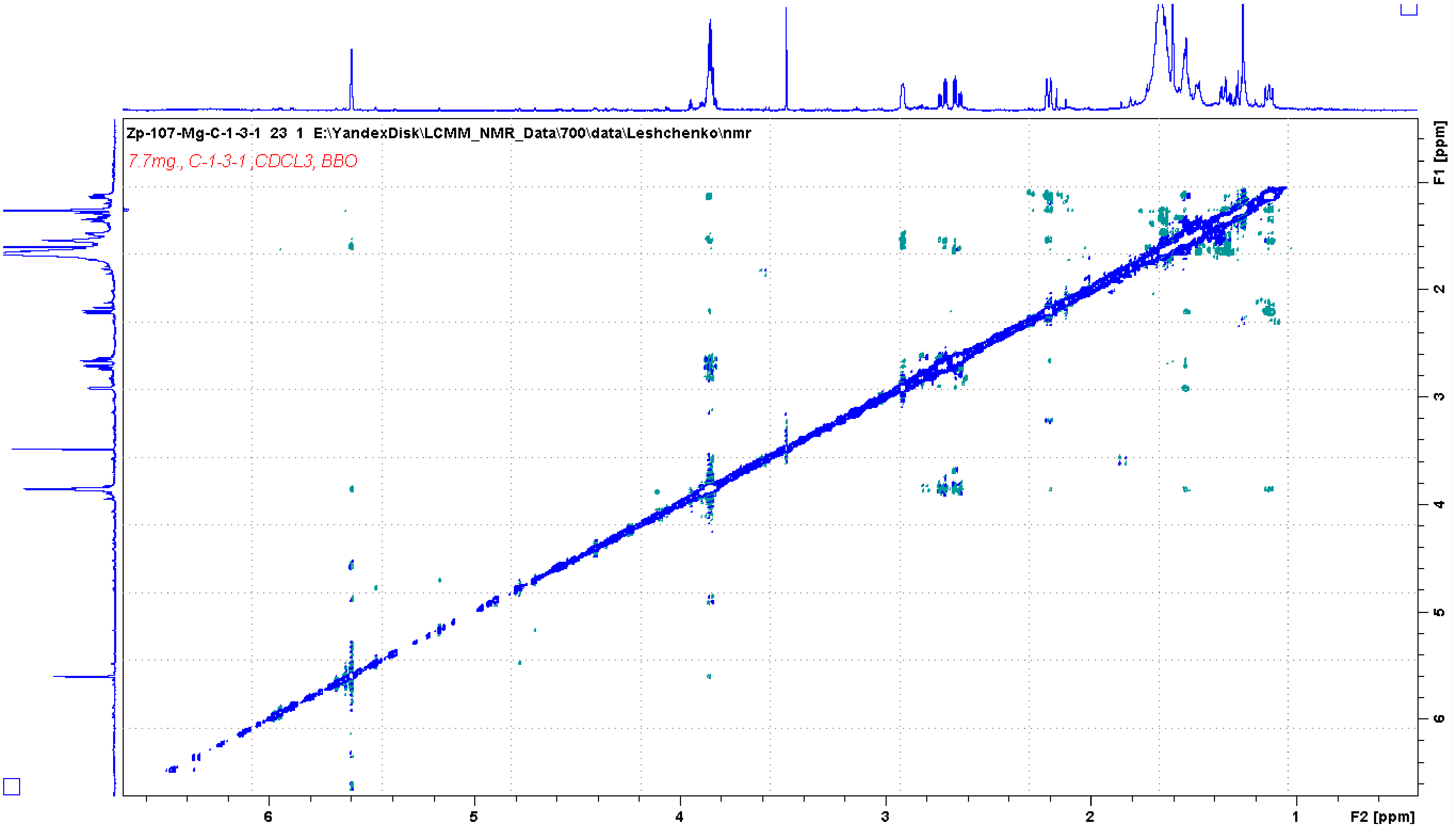

Figure S94. UV spectrum of **9** measured in MeOH

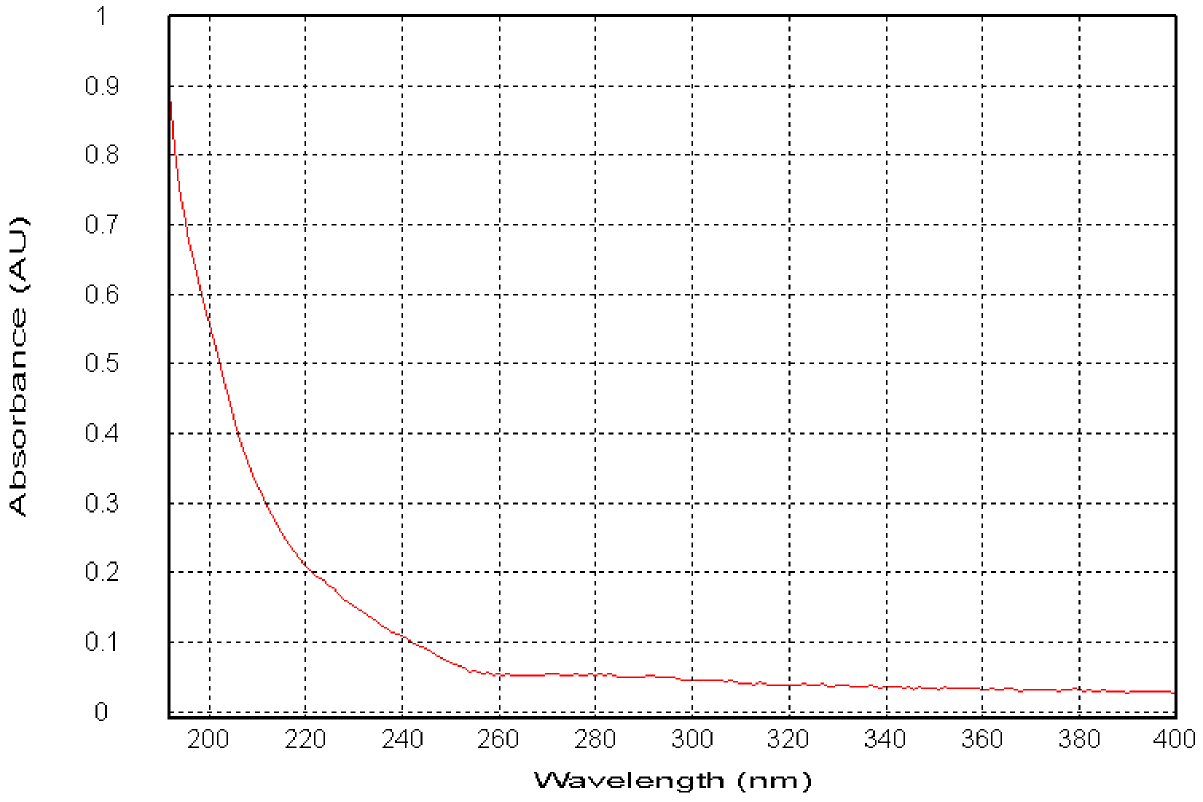

Figure S95. CD spectrum of **9** measured in MeOH

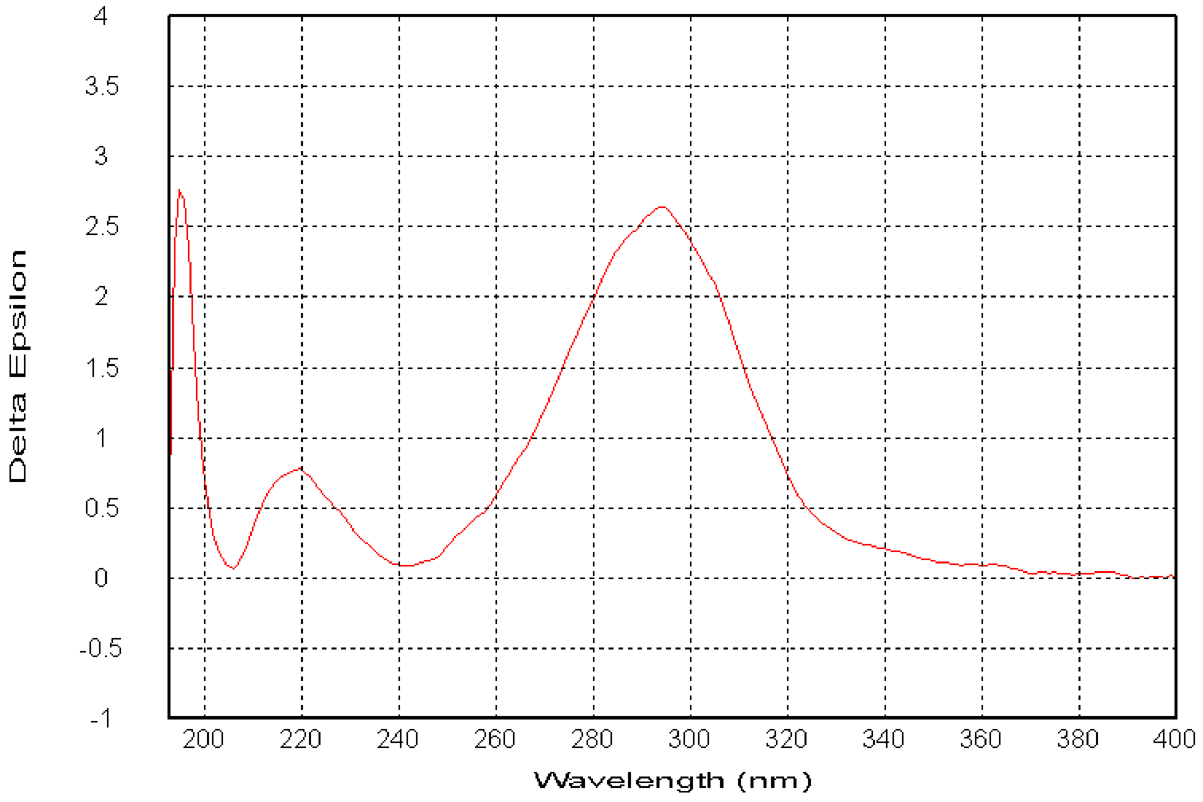

Figure S96. (A) Key COSY and (B) HMBC correlations of **10**

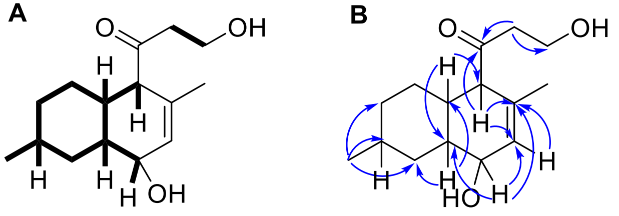

Figure S97. HRESIMS for **10**

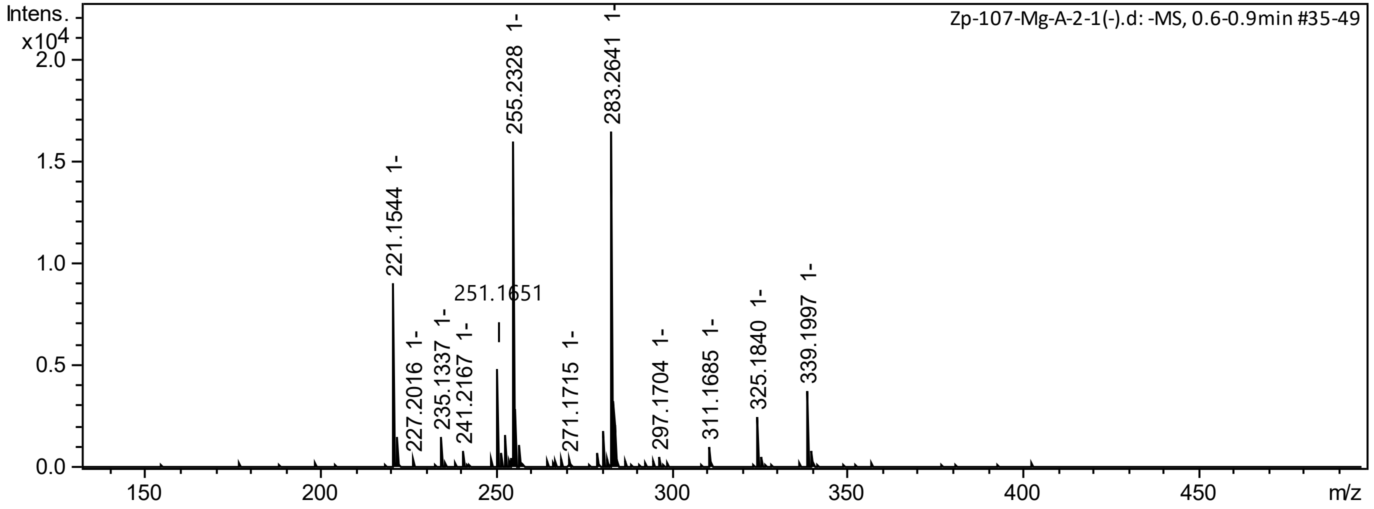

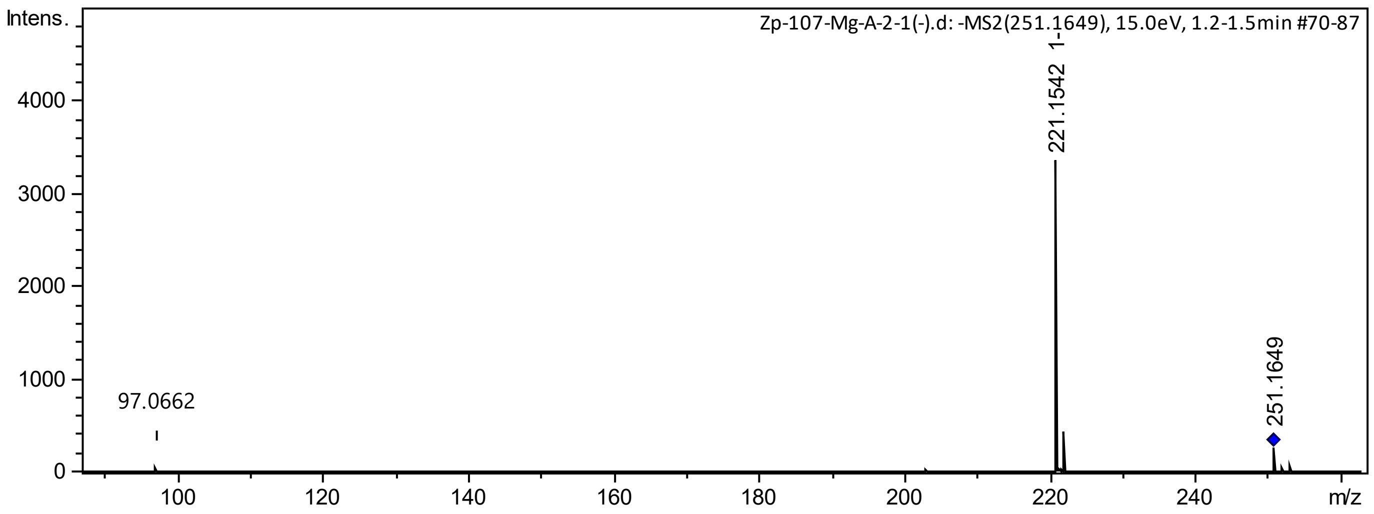
Intens.
Zp-107-Mg-A-2-1(-).d: -MS2(251.1649), 15.0eV, 1.2-1.5min #70-87
221.1542 1-
251.1649
97.0662
m/z

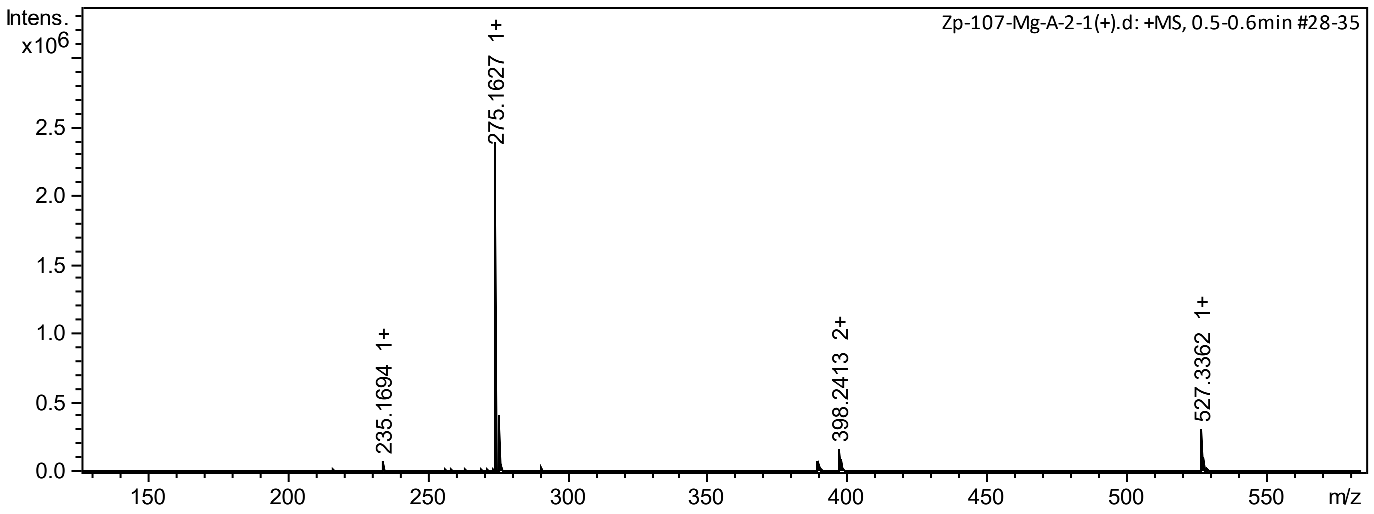
Intens.
x10^6
Zp-107-Mg-A-2-1(+).d: +MS, 0.5-0.6min #28-35
275.1627 1+
235.1694 1+
398.2413 2+
527.3362 1+
m/z

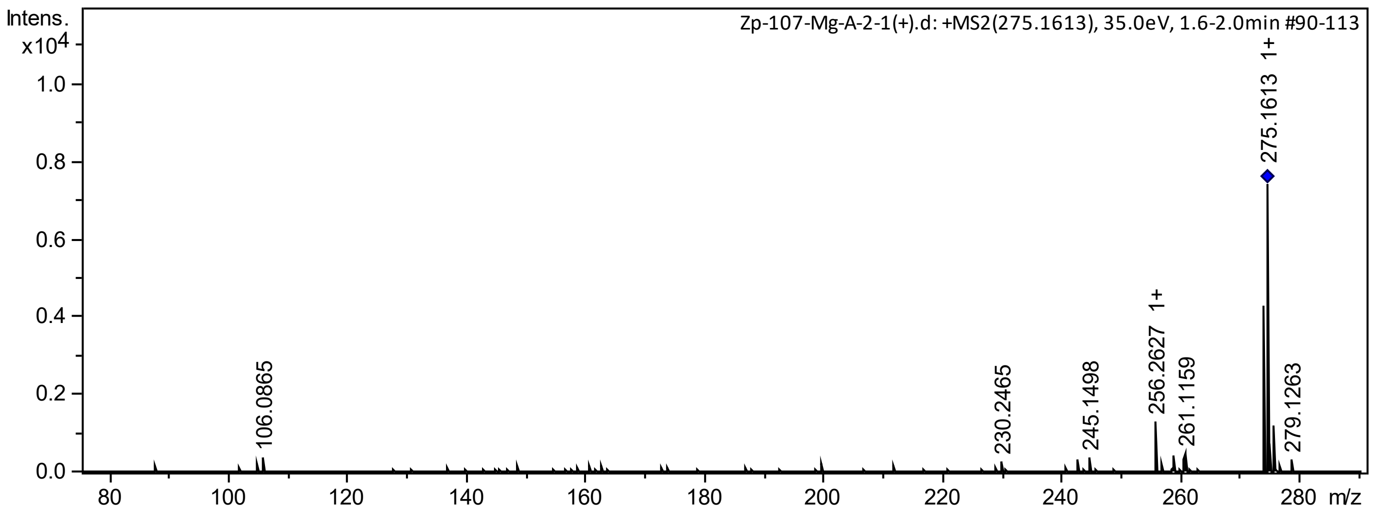

	meas	calc	Δ (ppm)
$[M-H]^-$	251,1651	251,1653	0,8
$[M+Na]^+$	275,1627	275,1618	-3,3

Figure S98. ^{1}H NMR spectrum of **10** measured at 700 MHz in $CDCl_3$

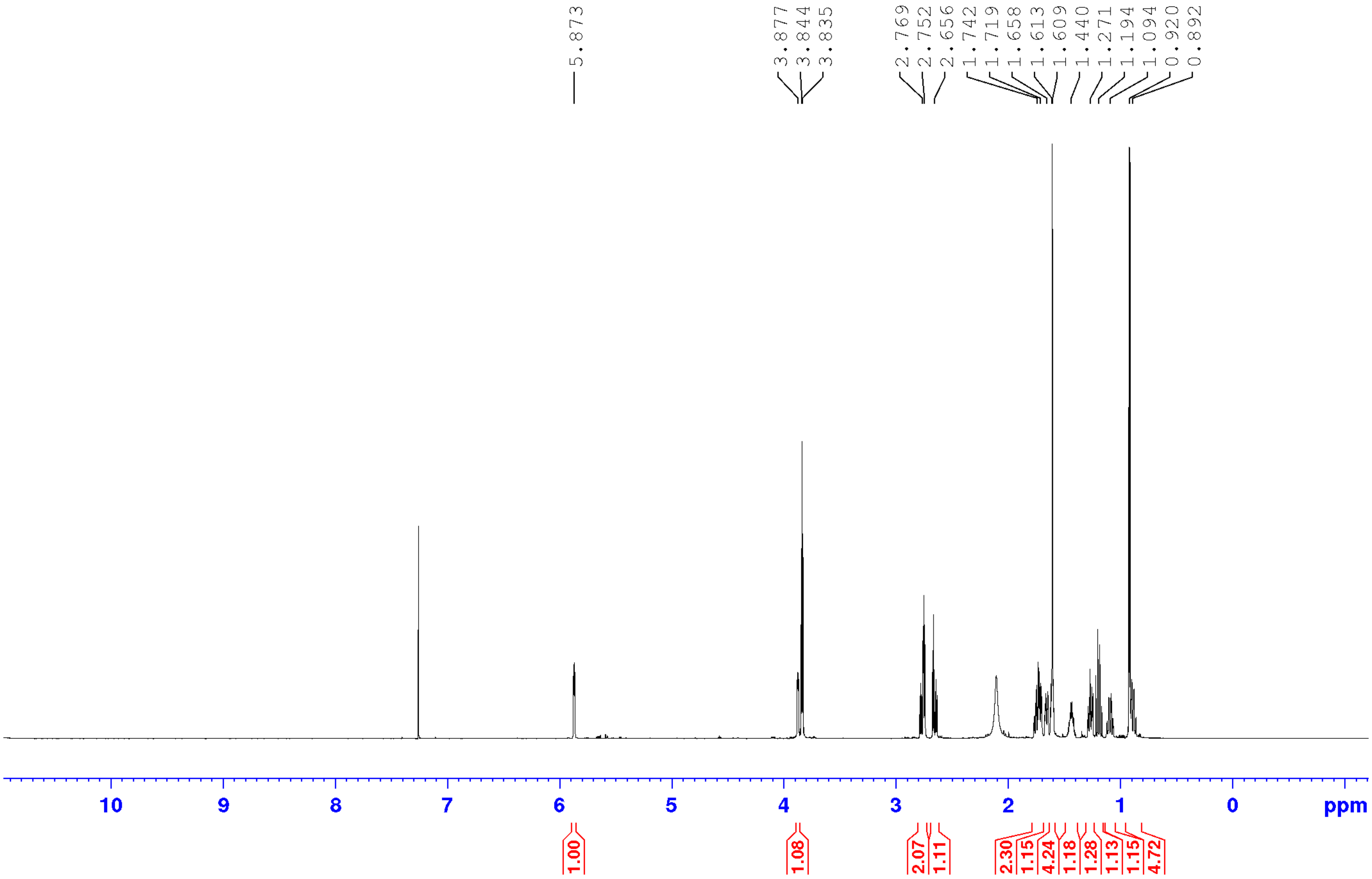

Figure S99. ^{13}C NMR spectrum of **10** measured at 176 MHz in $CDCl_3$

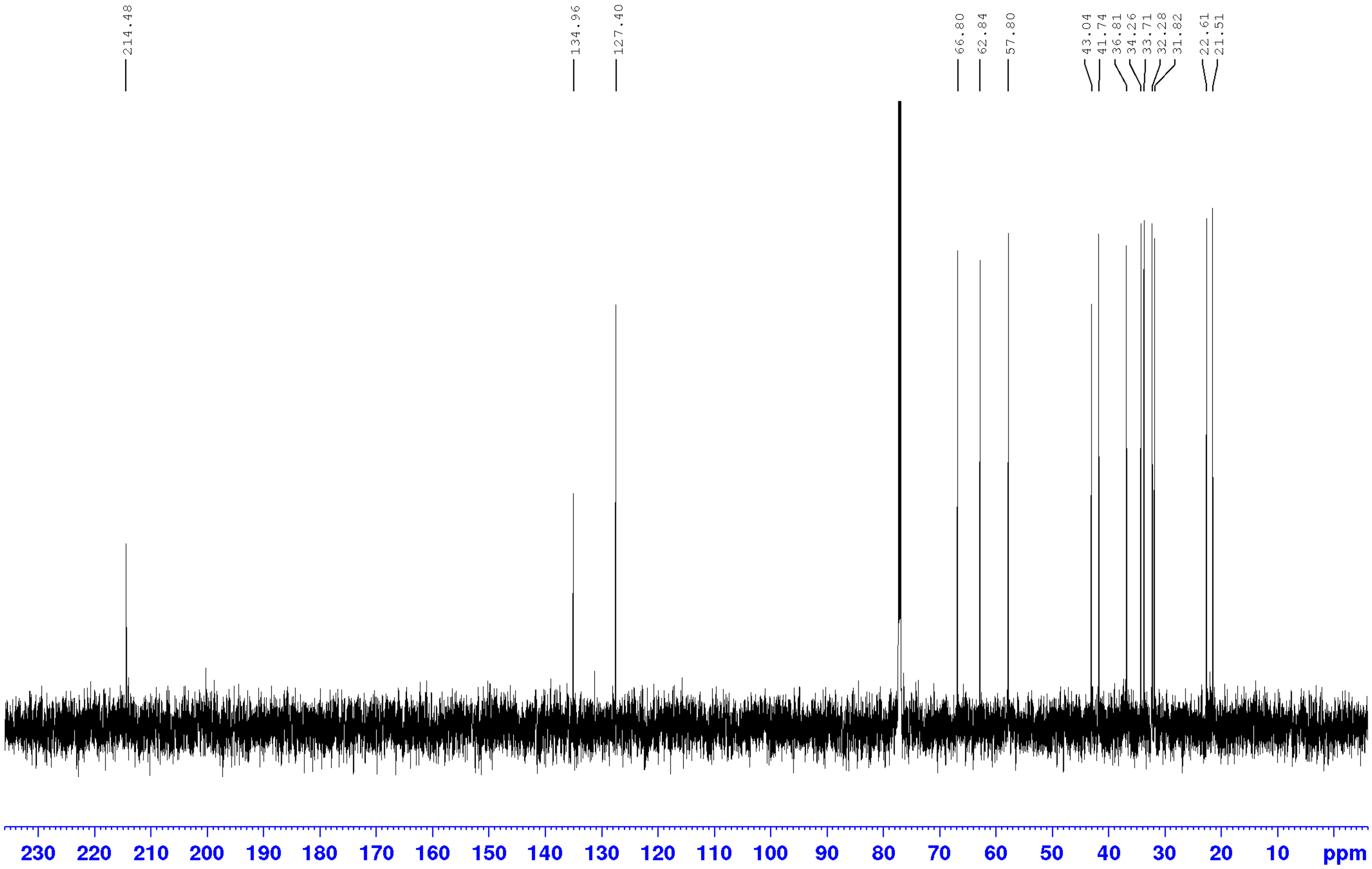

Figure S100. DEPT-135 spectrum of **10** measured at 176 MHz in $CDCl_3$

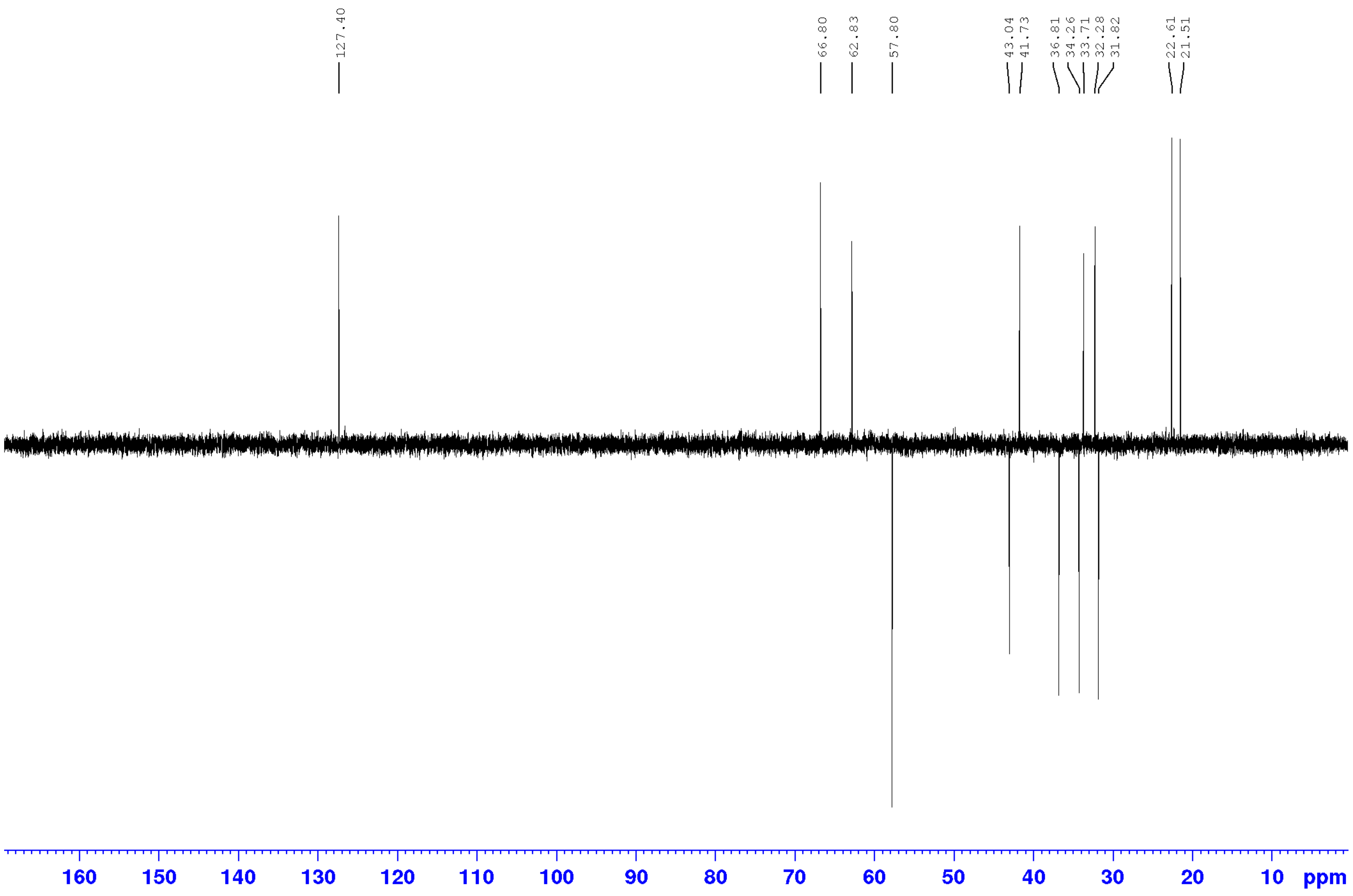

Figure S101. HSQC spectrum of **10** measured in $CDCl_3$

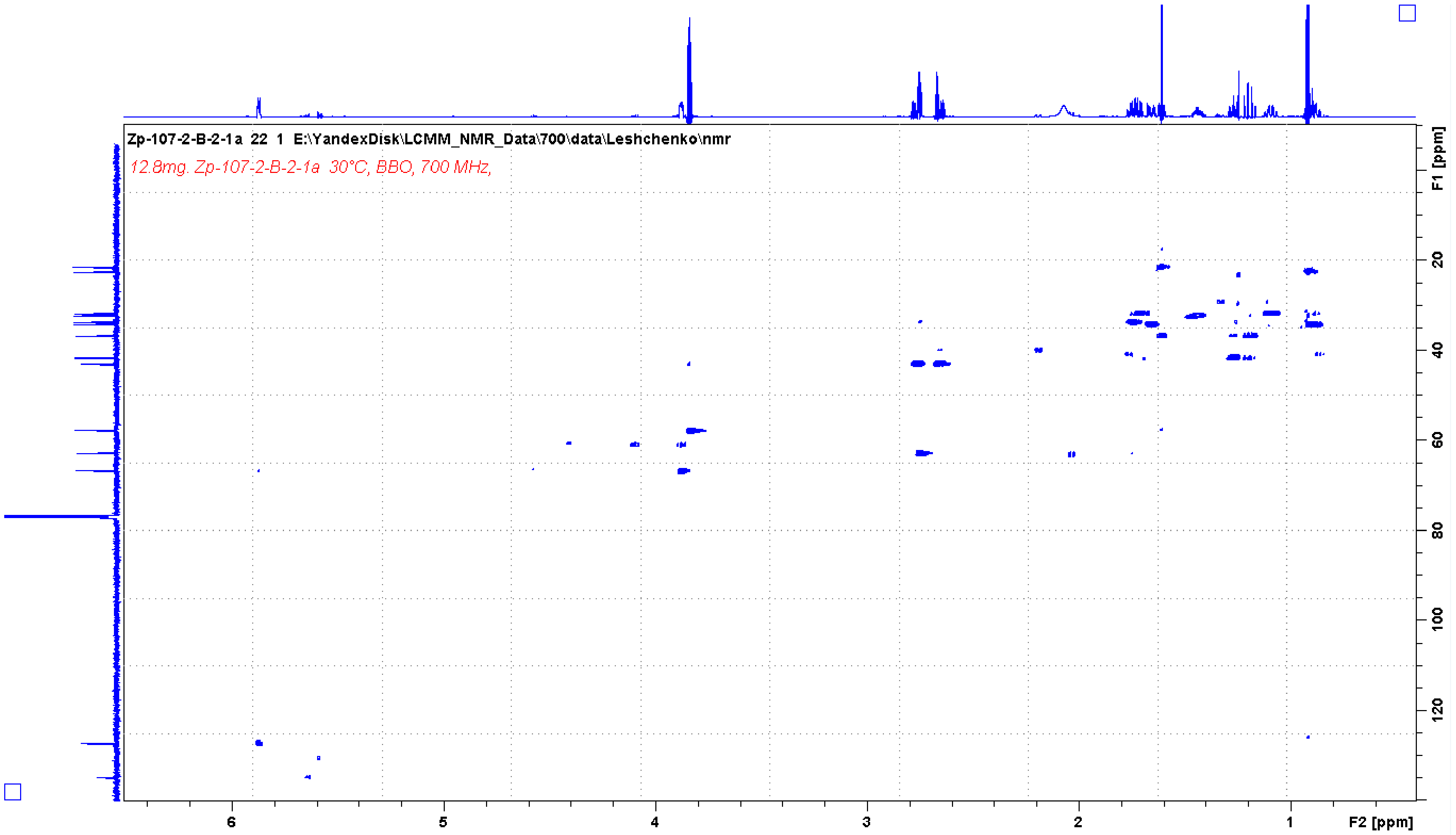

Figure S102. COSY spectrum of **10** measured in $CDCl_3$

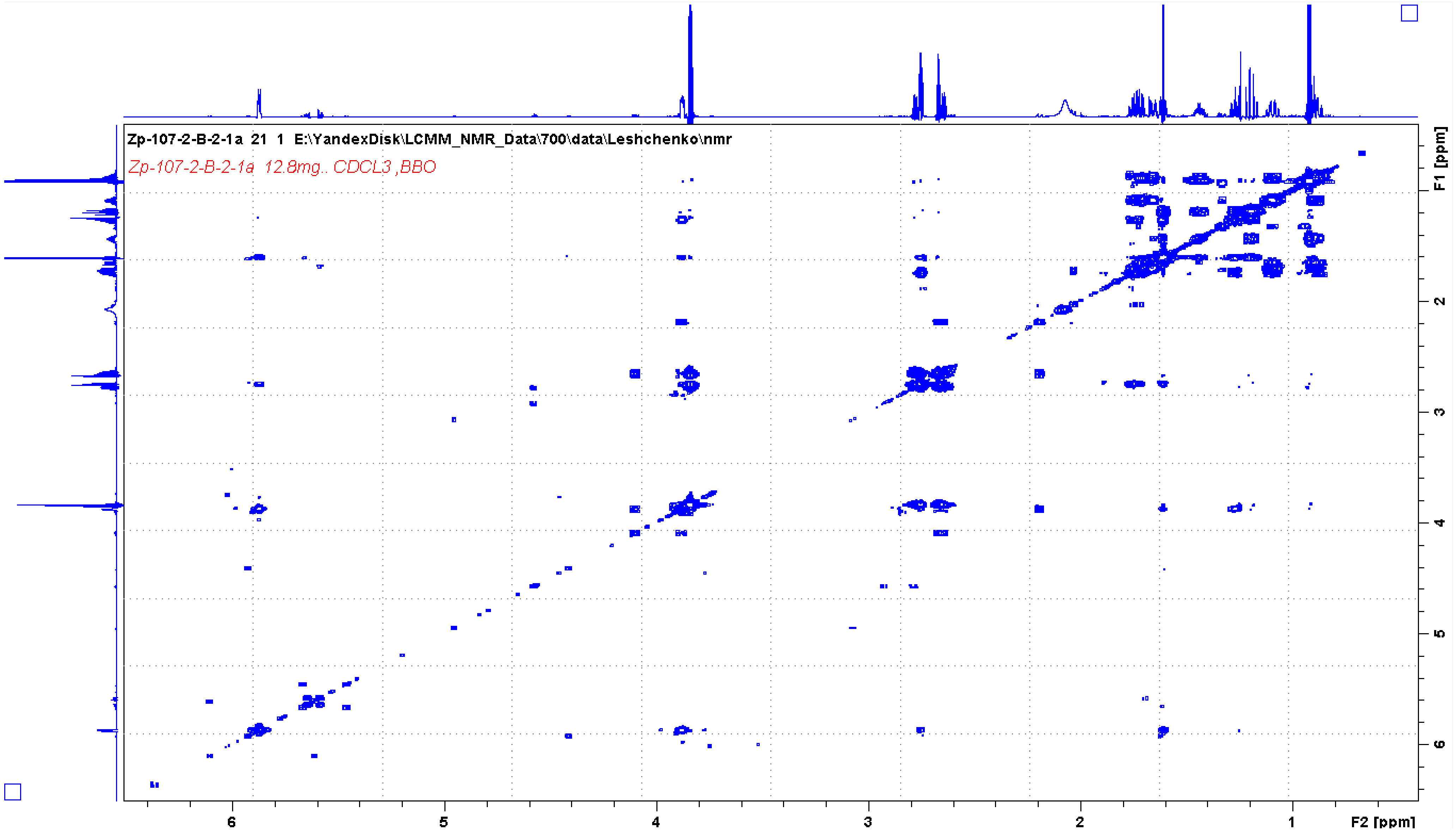

Figure S103. HMBC spectrum of **10** measured in $CDCl_3$

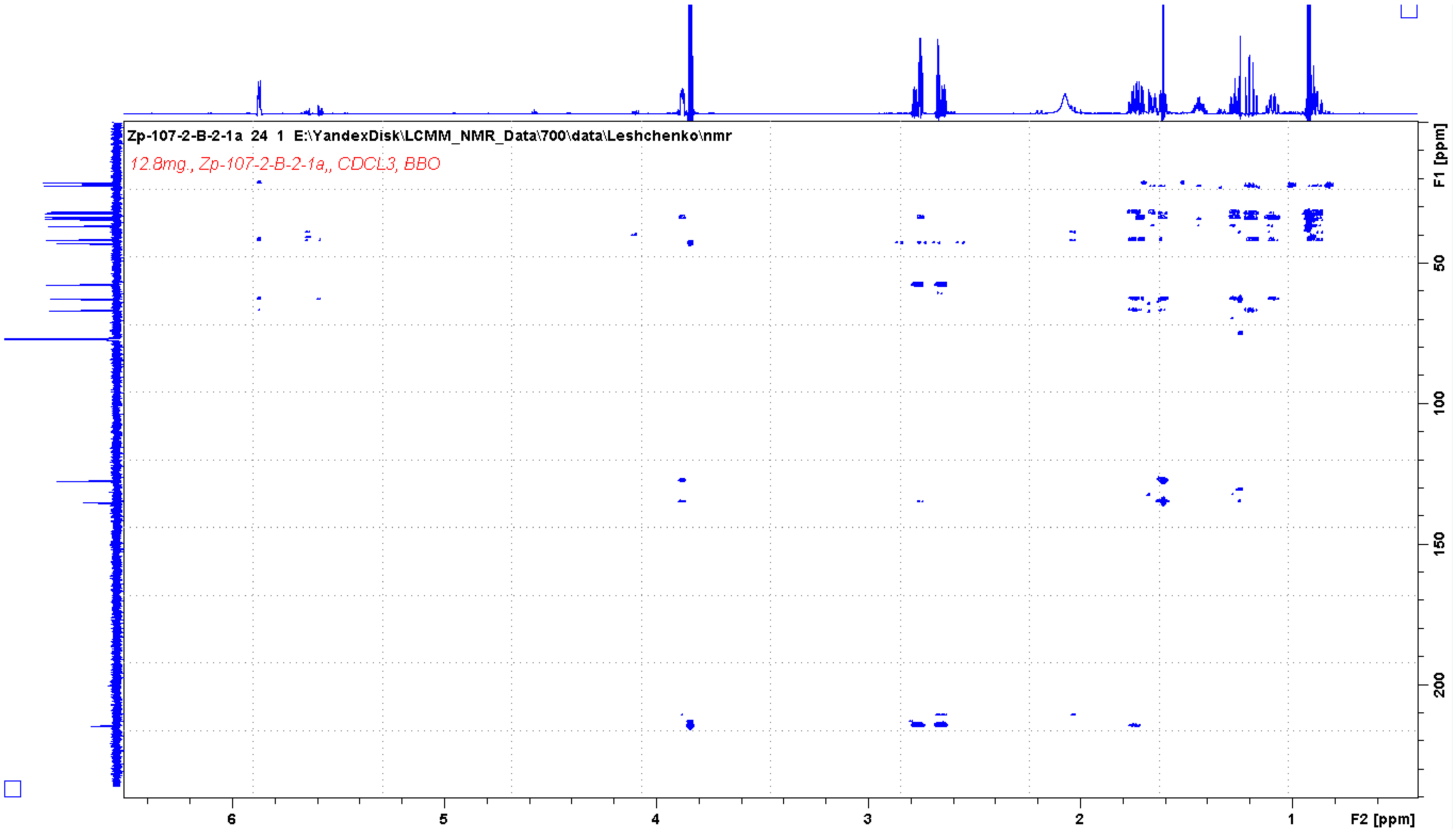

Figure S104. ROESY spectrum of **10** measured in $CDCl_3$

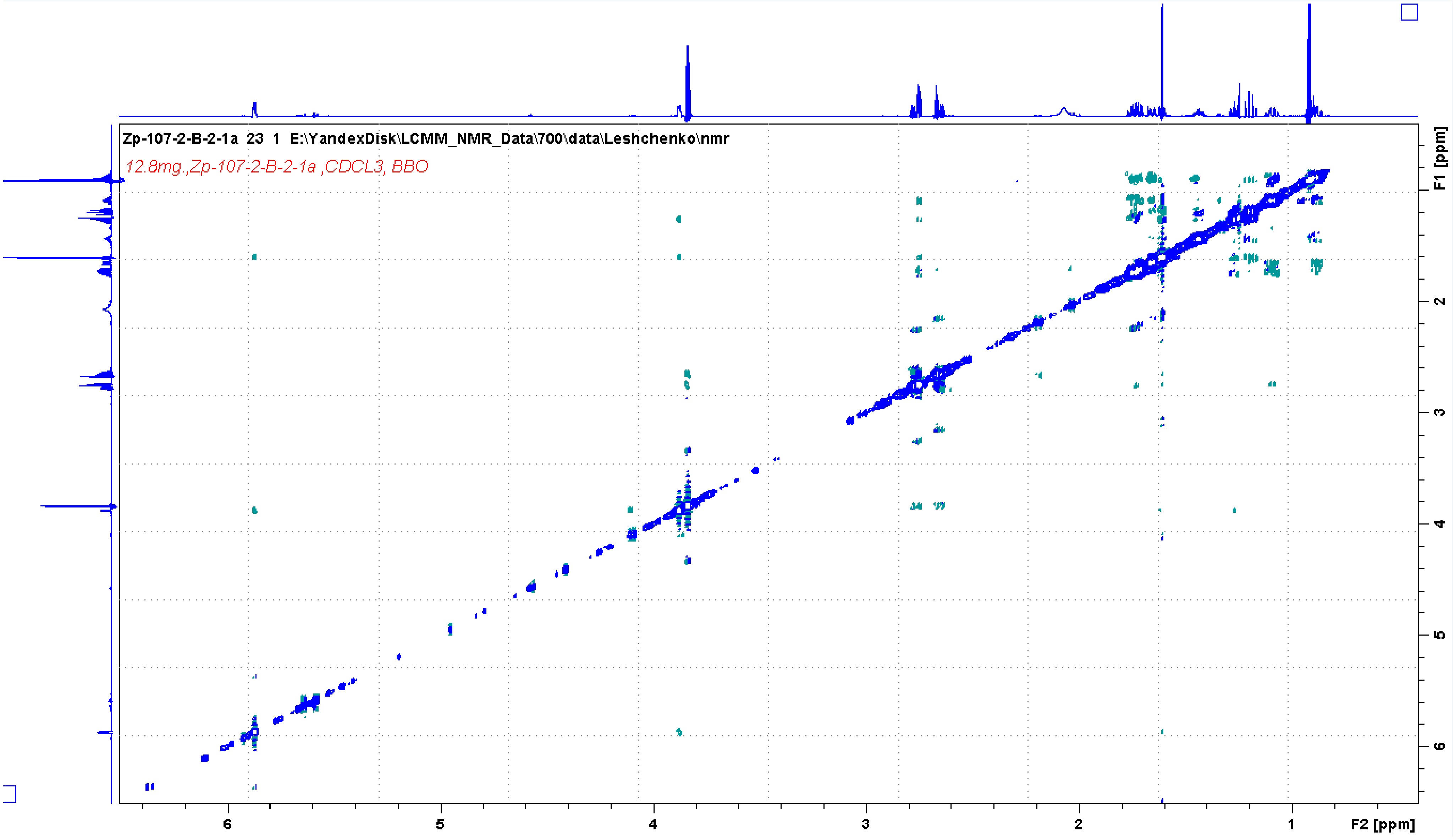

Figure S105. UV spectrum of **10** measured in MeOH

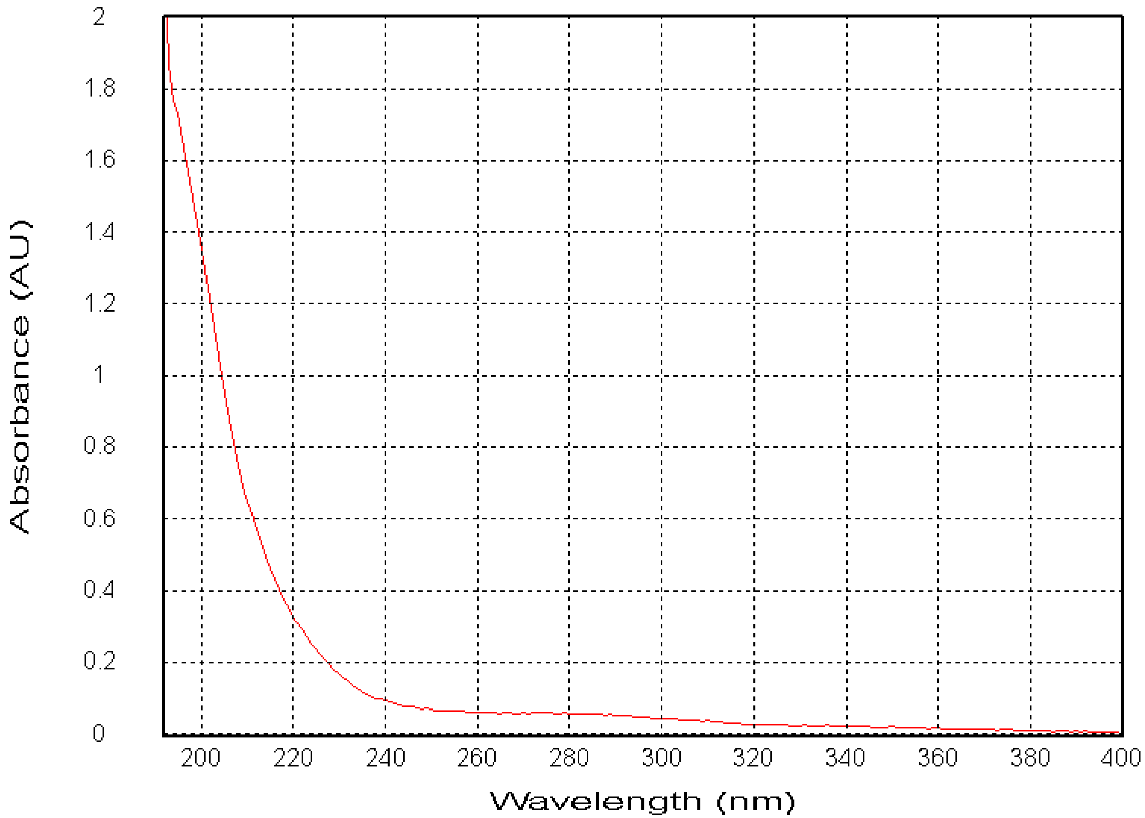

Figure S106. CD spectrum of **10** measured in MeOH

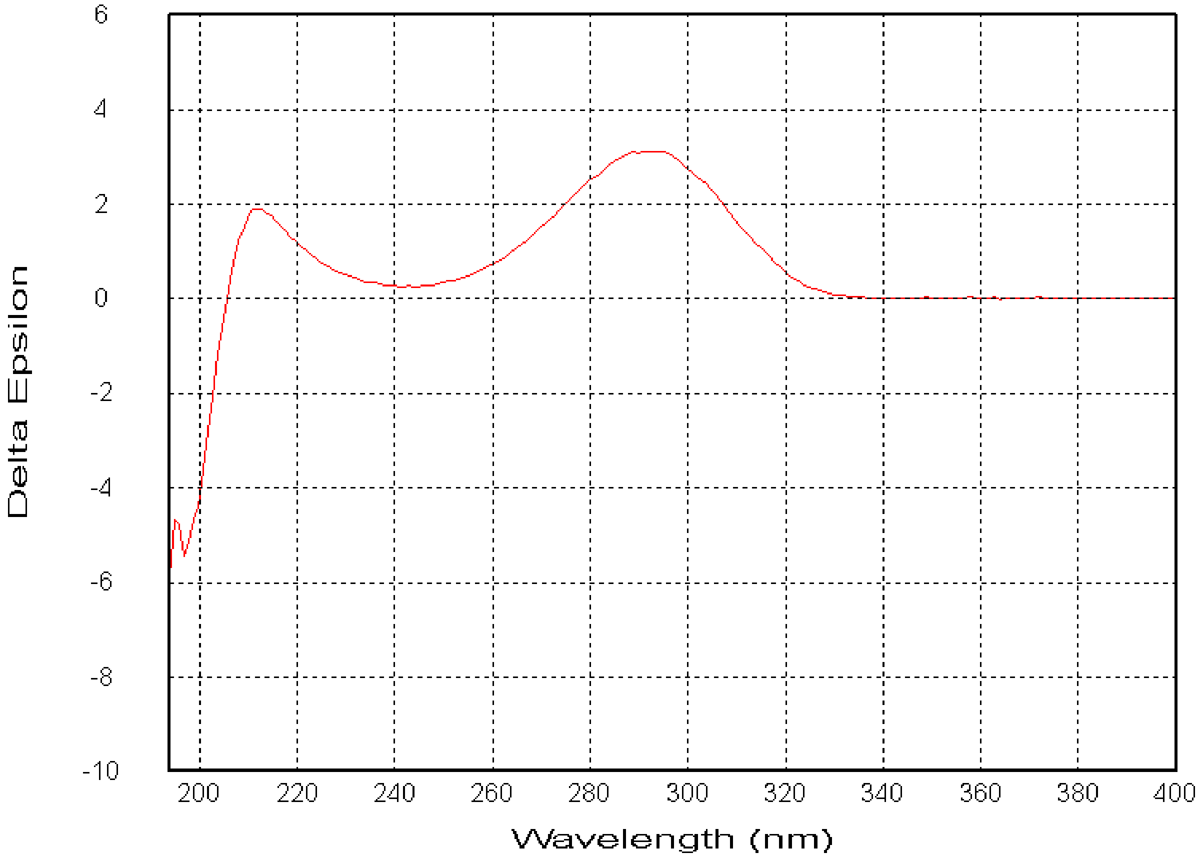

Figure S107. (**A**) Key COSY, (**B**) HMBC and (**C**) ROESY correlations of **11**

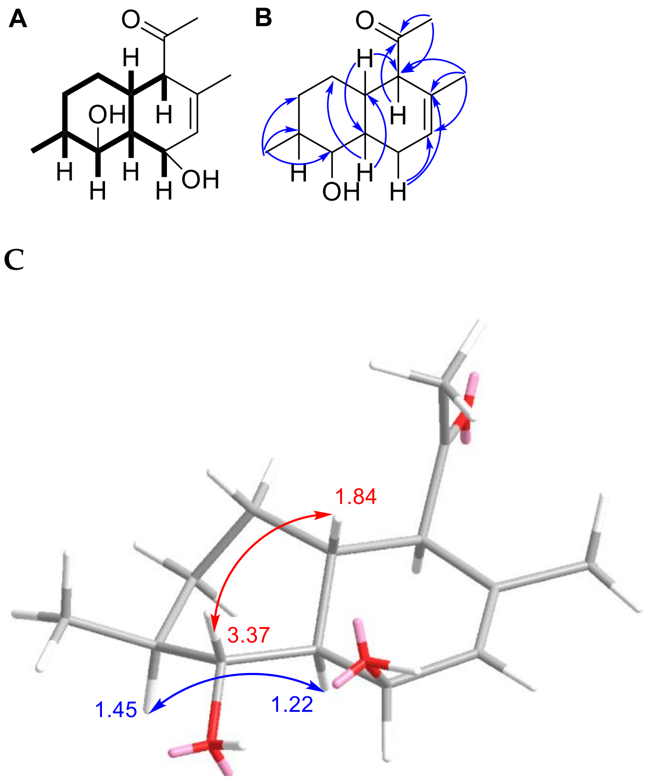

Figure S108. HRESIMS for **11**

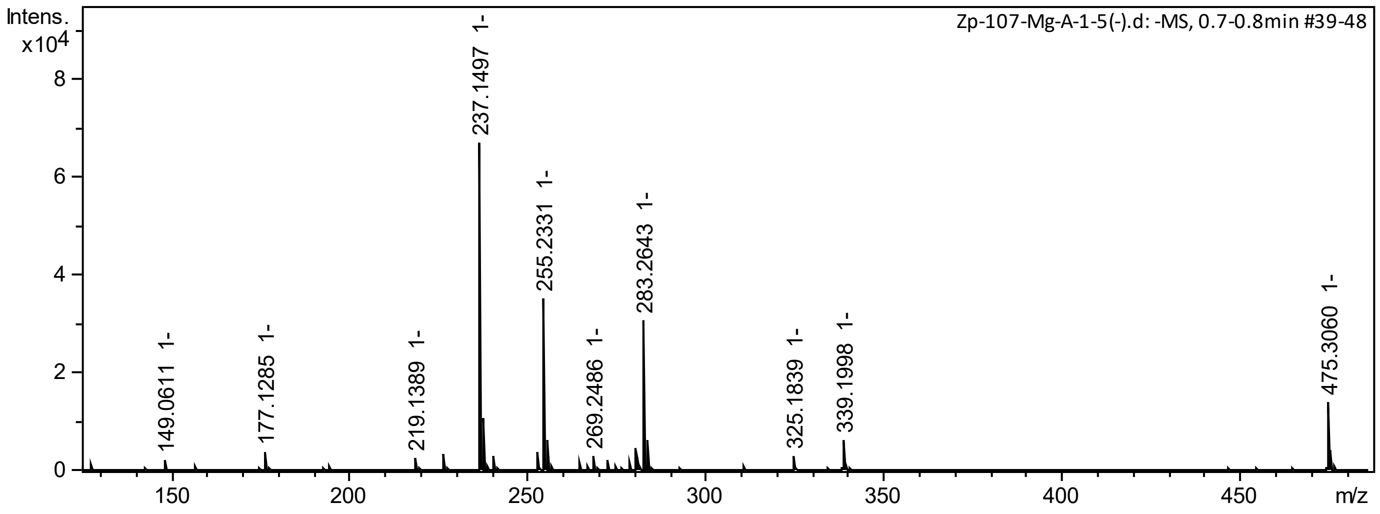

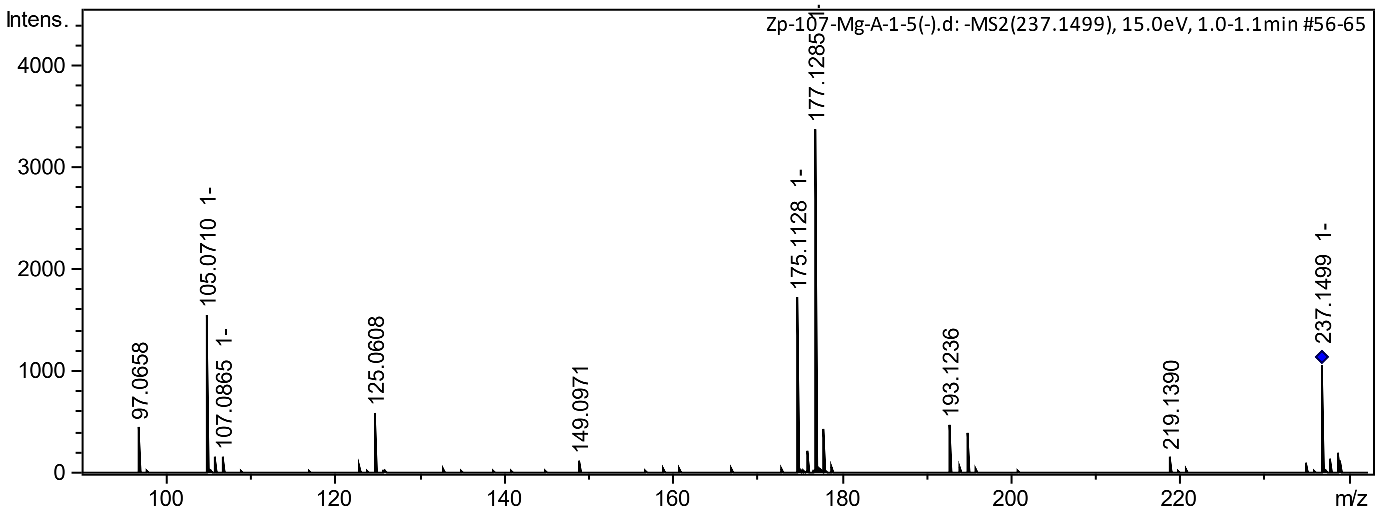

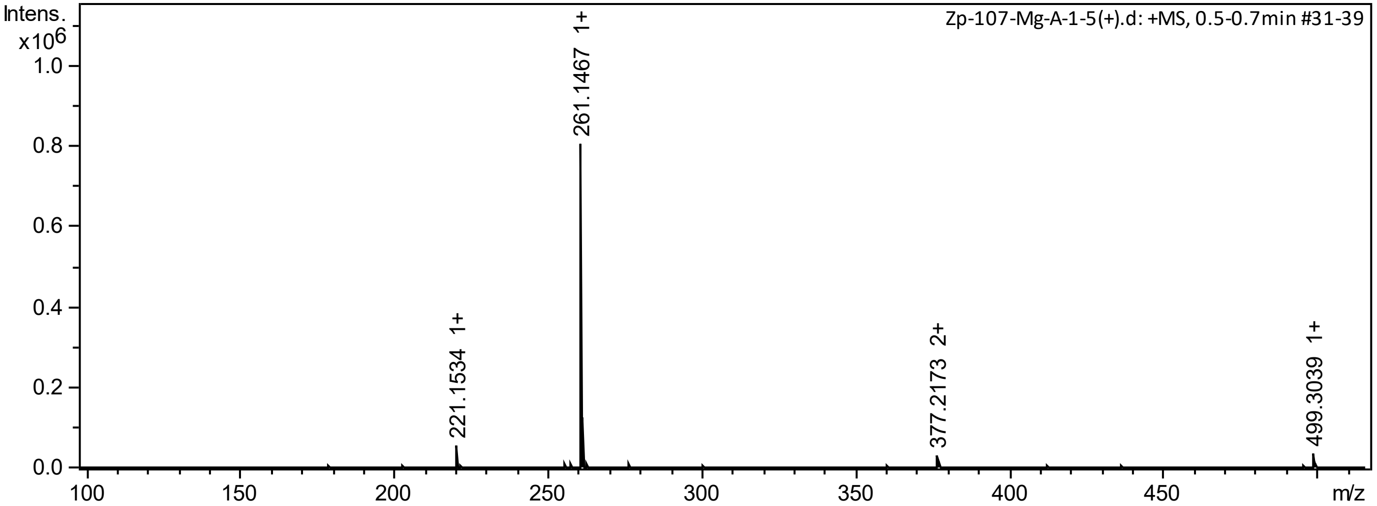

	meas	calc	Δ (ppm)
$[M\text{-}H]^-$	237,1497	237,1496	-0,4
$[M+Na]^+$	261,1467	261,1461	-2,3

Figure S109. ^{1}H NMR spectrum of **11** measured at 700 MHz in $CDCl_3$

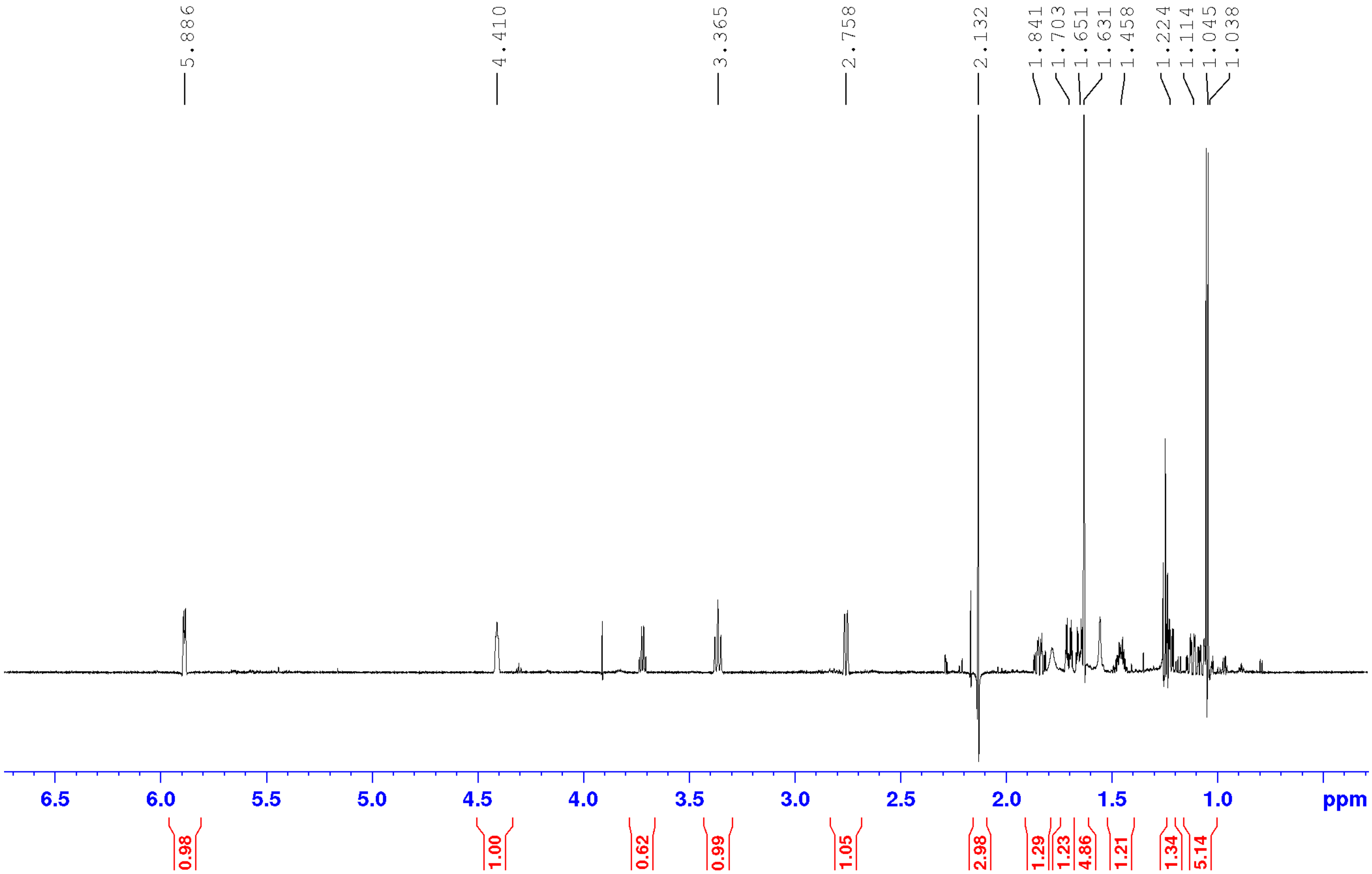

Figure S110. ^{13}C NMR spectrum of **11** measured at 176 MHz in $CDCl_3$

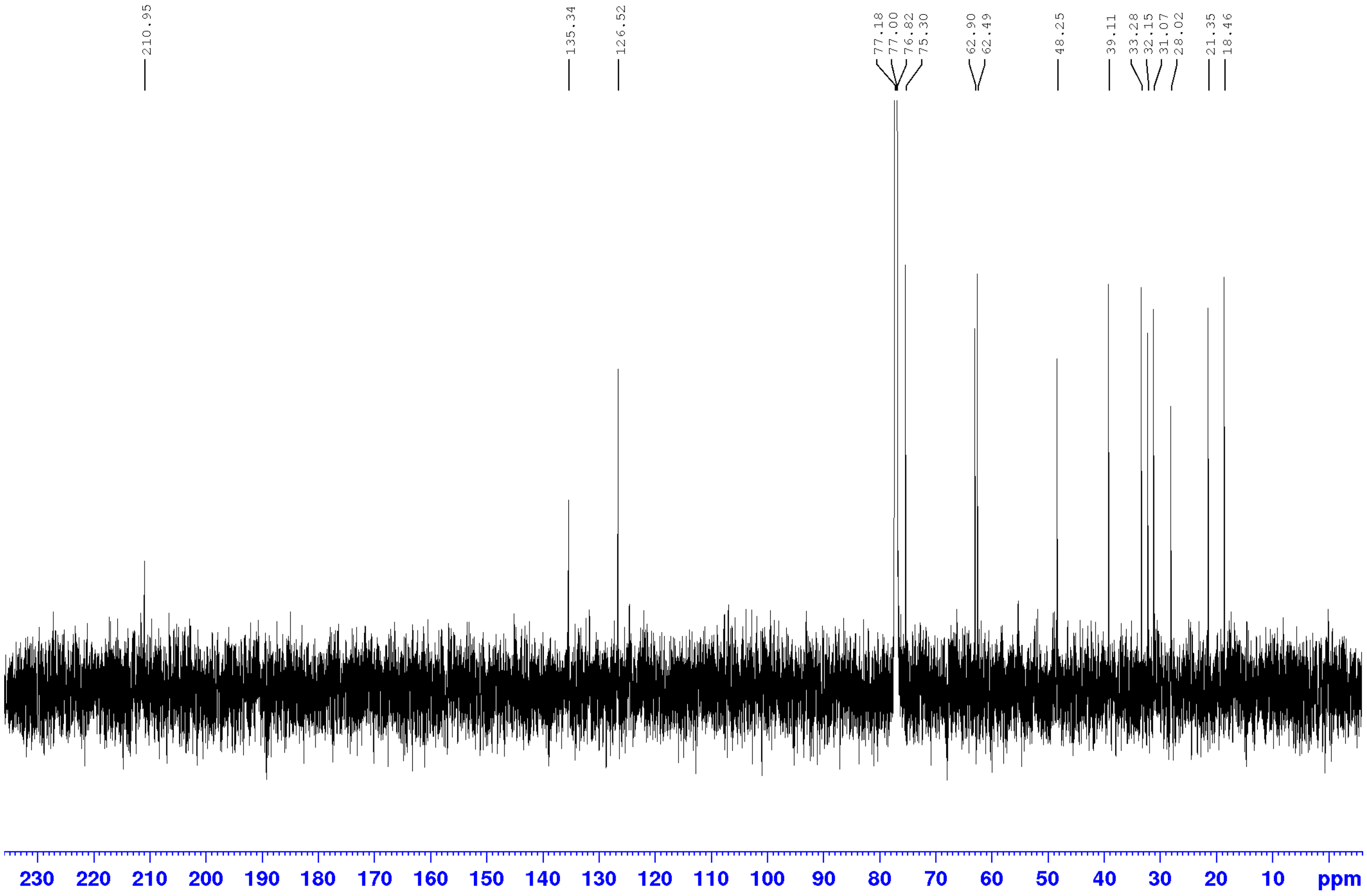

Figure S111. DEPT-135 spectrum of **11** measured at 176 MHz in $CDCl_3$

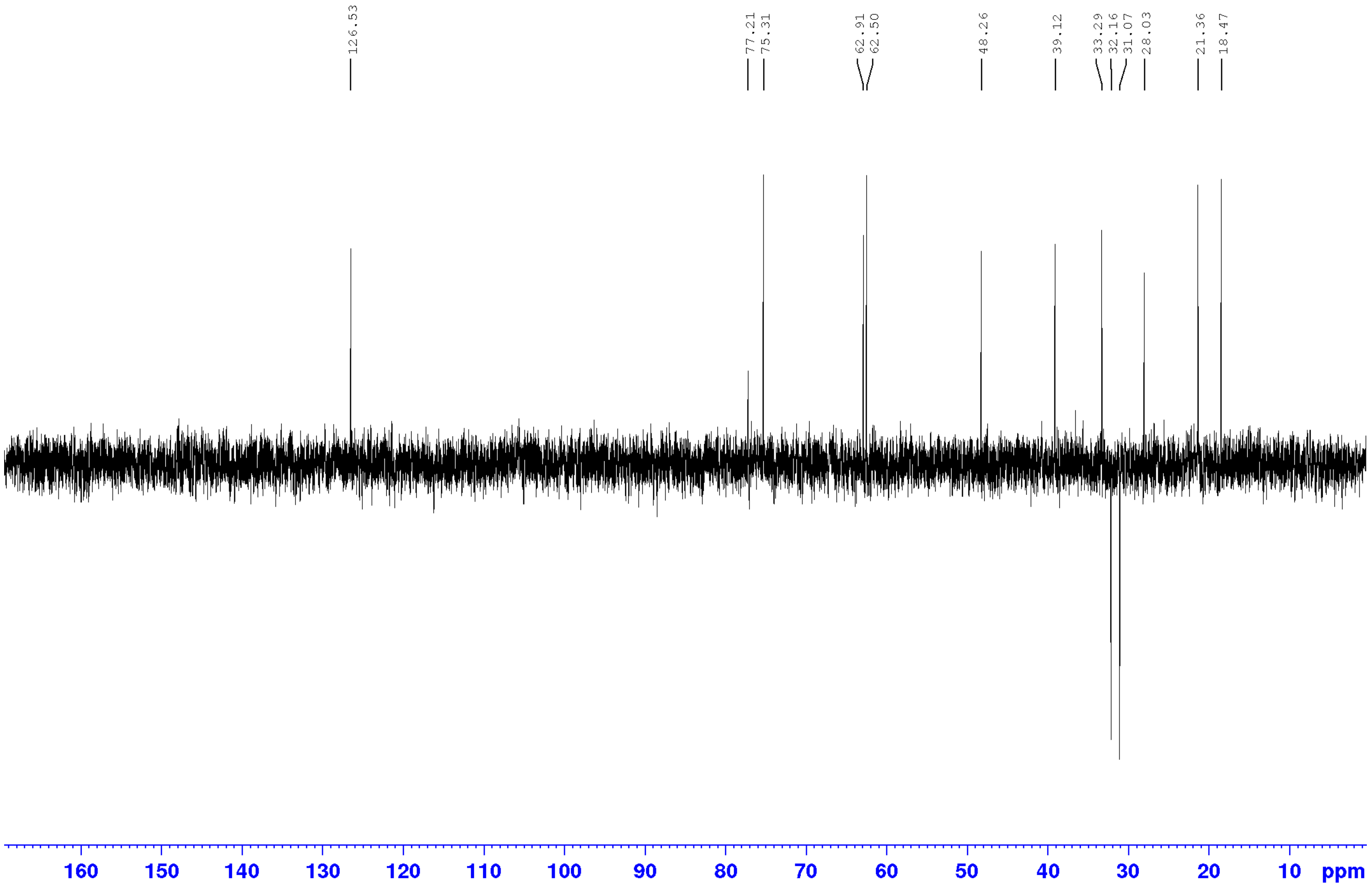

Figure S112. HSQC spectrum of **11** measured in $CDCl_3$

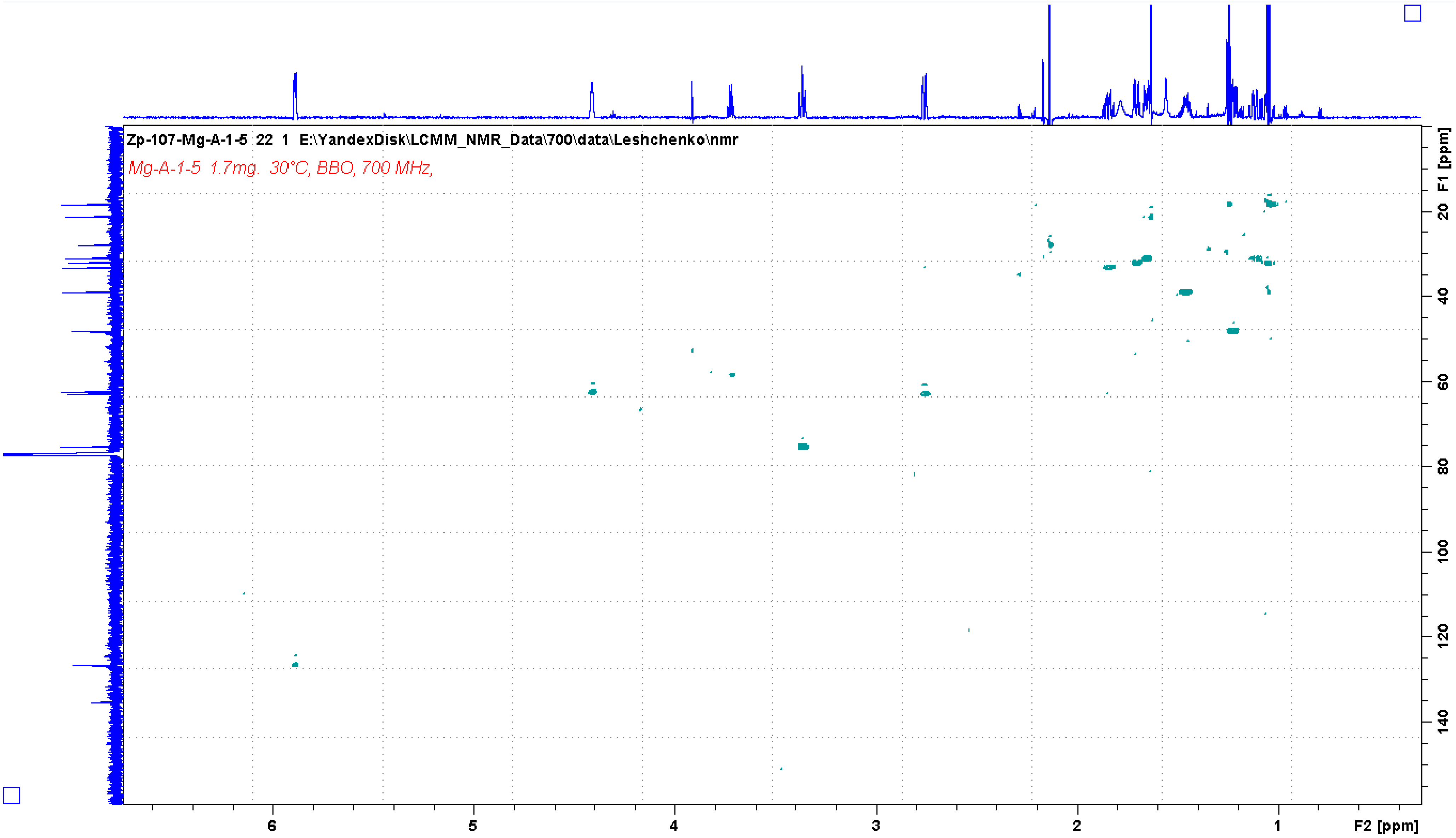

Figure S113. COSY spectrum of **11** measured in $CDCl_3$

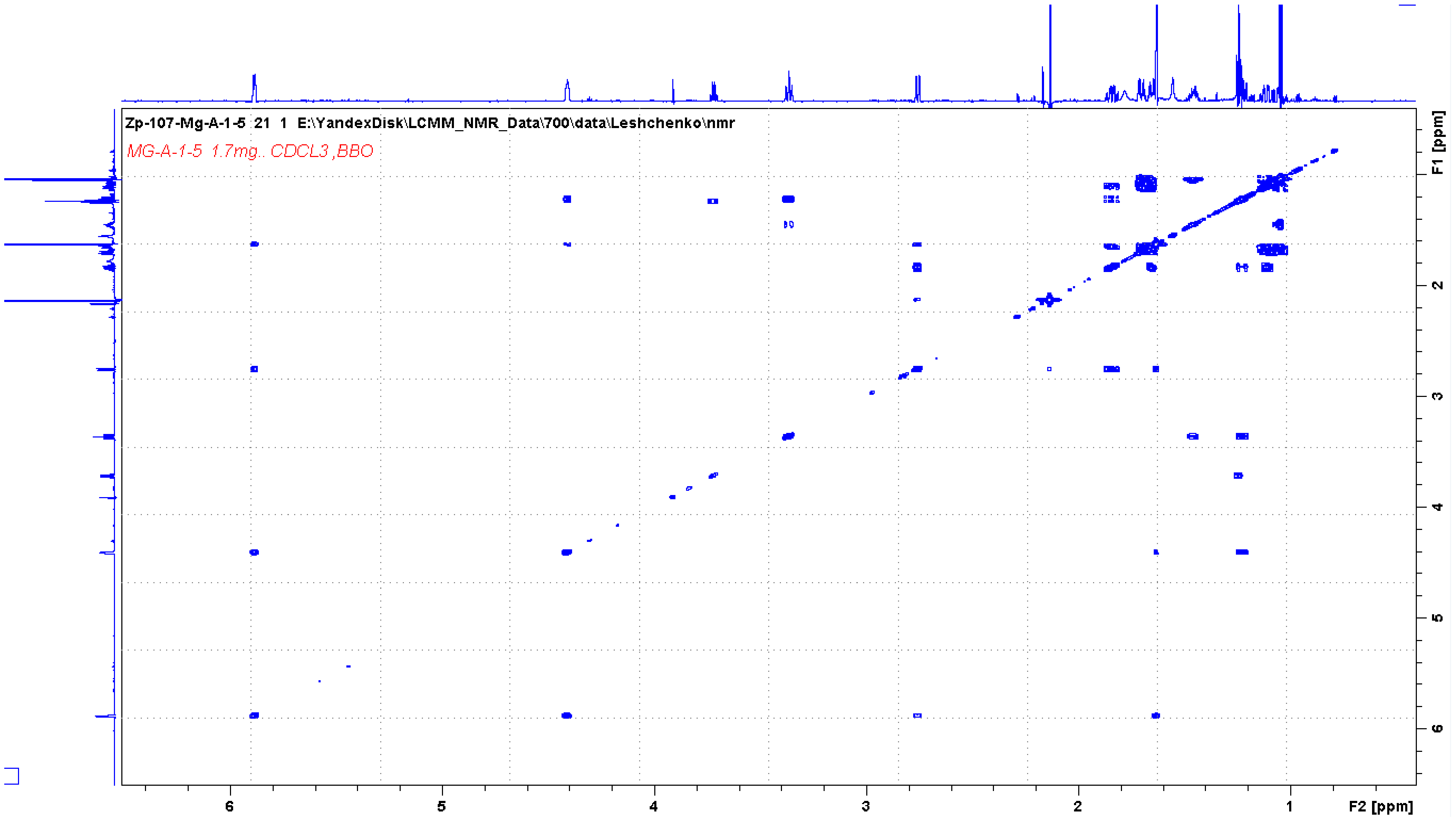

Figure S114. HMBC spectrum of **11** measured in $CDCl_3$

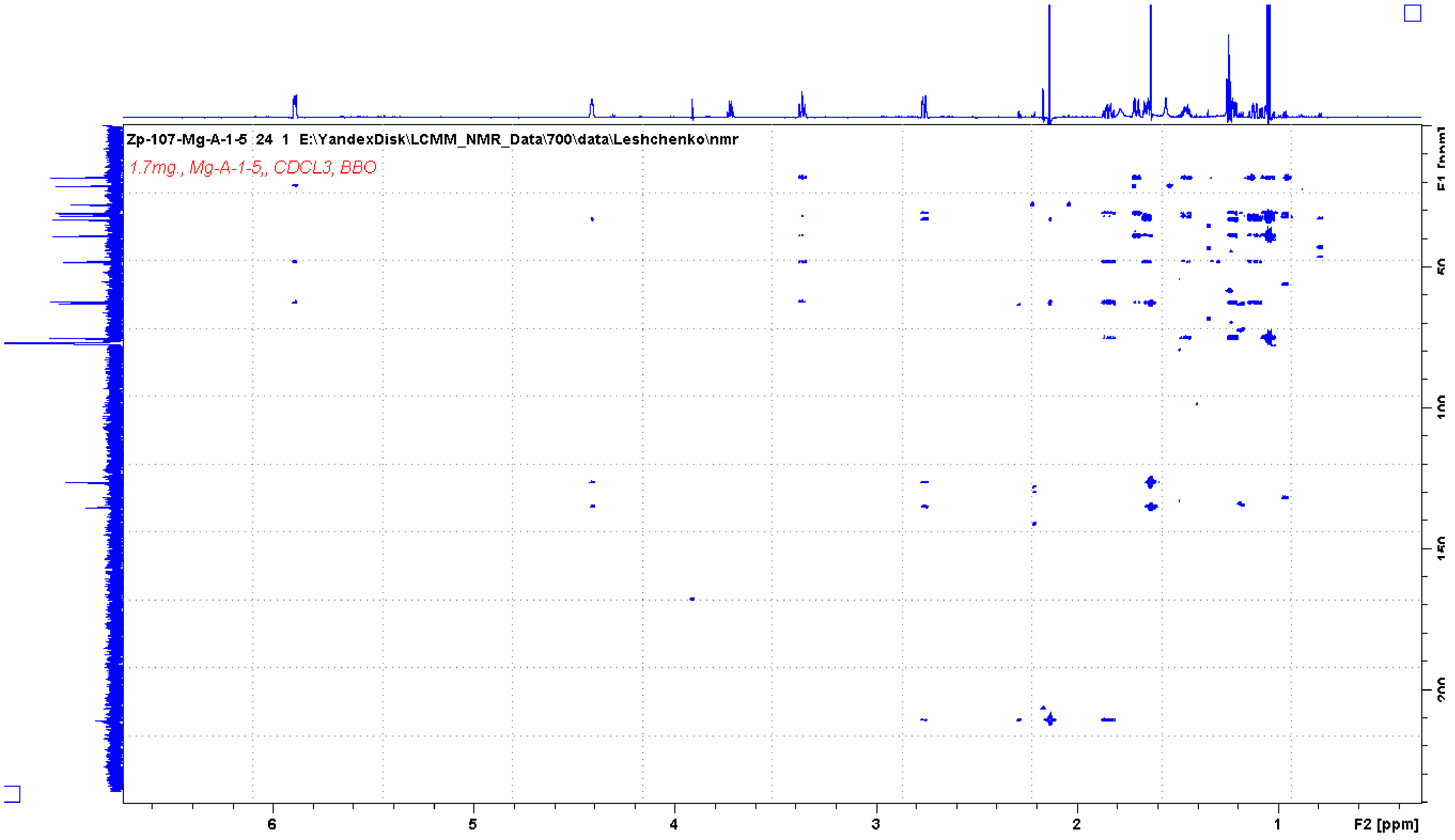

Figure S115. ROESY spectrum of **11** measured in $CDCl_3$

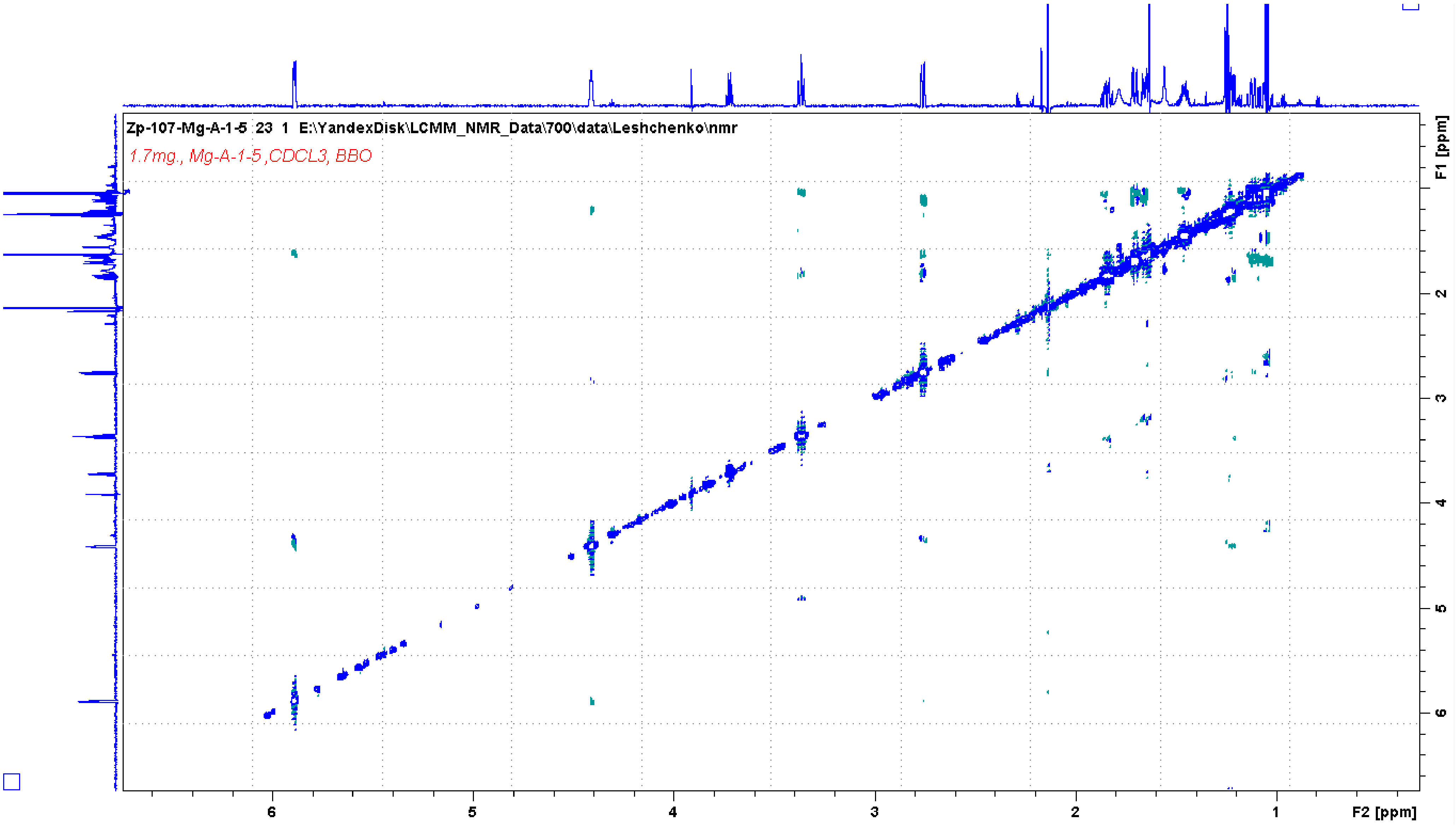

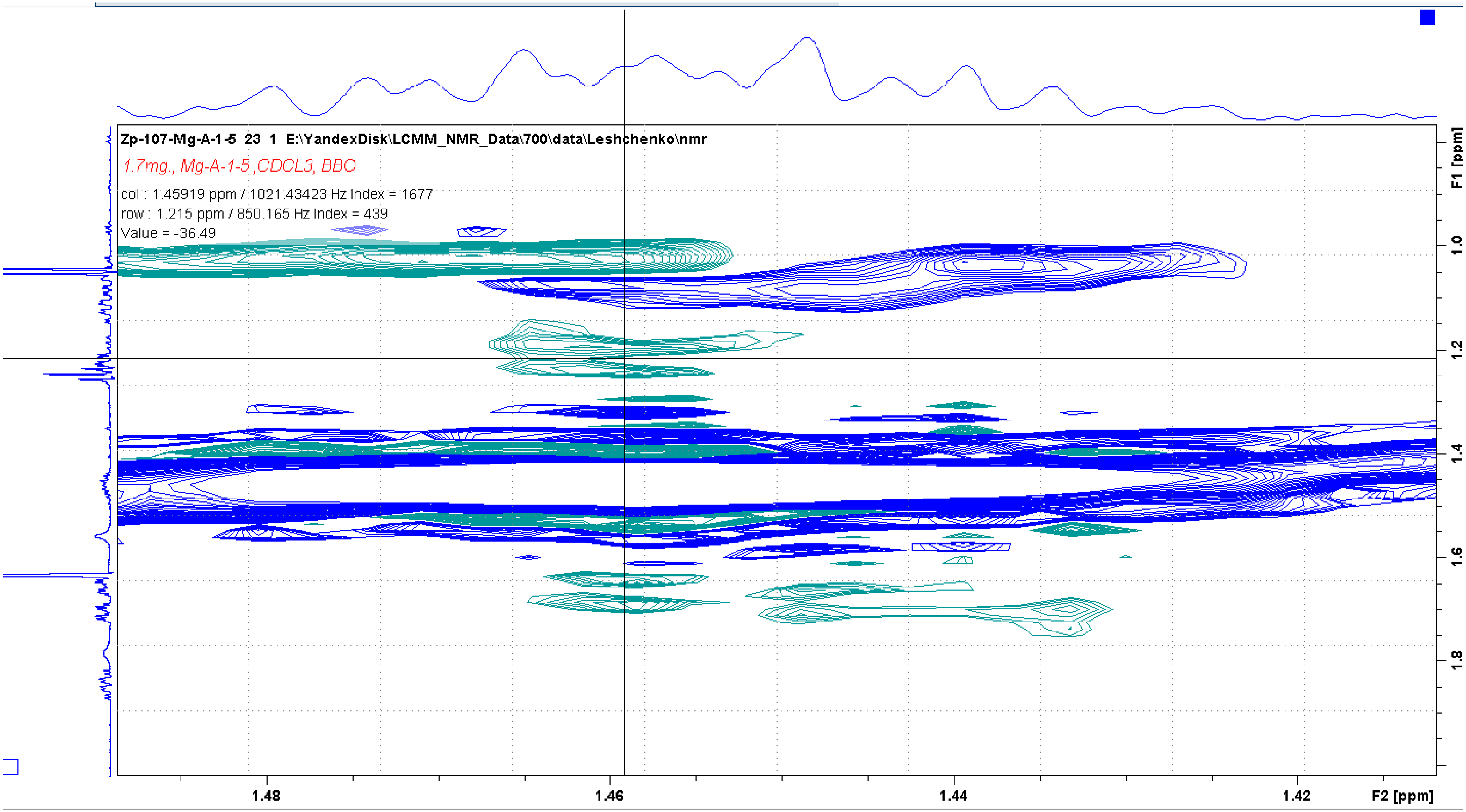

Zp-107-Mg-A-1-5 23 1 E:\YandexDisk\LCMM_NMR_Data\700\data\Leshchenko\nmr
1.7mg., Mg-A-1-5 ,CDCL3, BBO
col : 1.45919 ppm / 1021.43423 Hz Index = 1677
row : 1.215 ppm / 850.165 Hz Index = 439
Value = -36.49
F1 [ppm]
1.0
1.2
1.4
1.6
1.8
1.48
1.46
1.44
1.42
F2 [ppm]

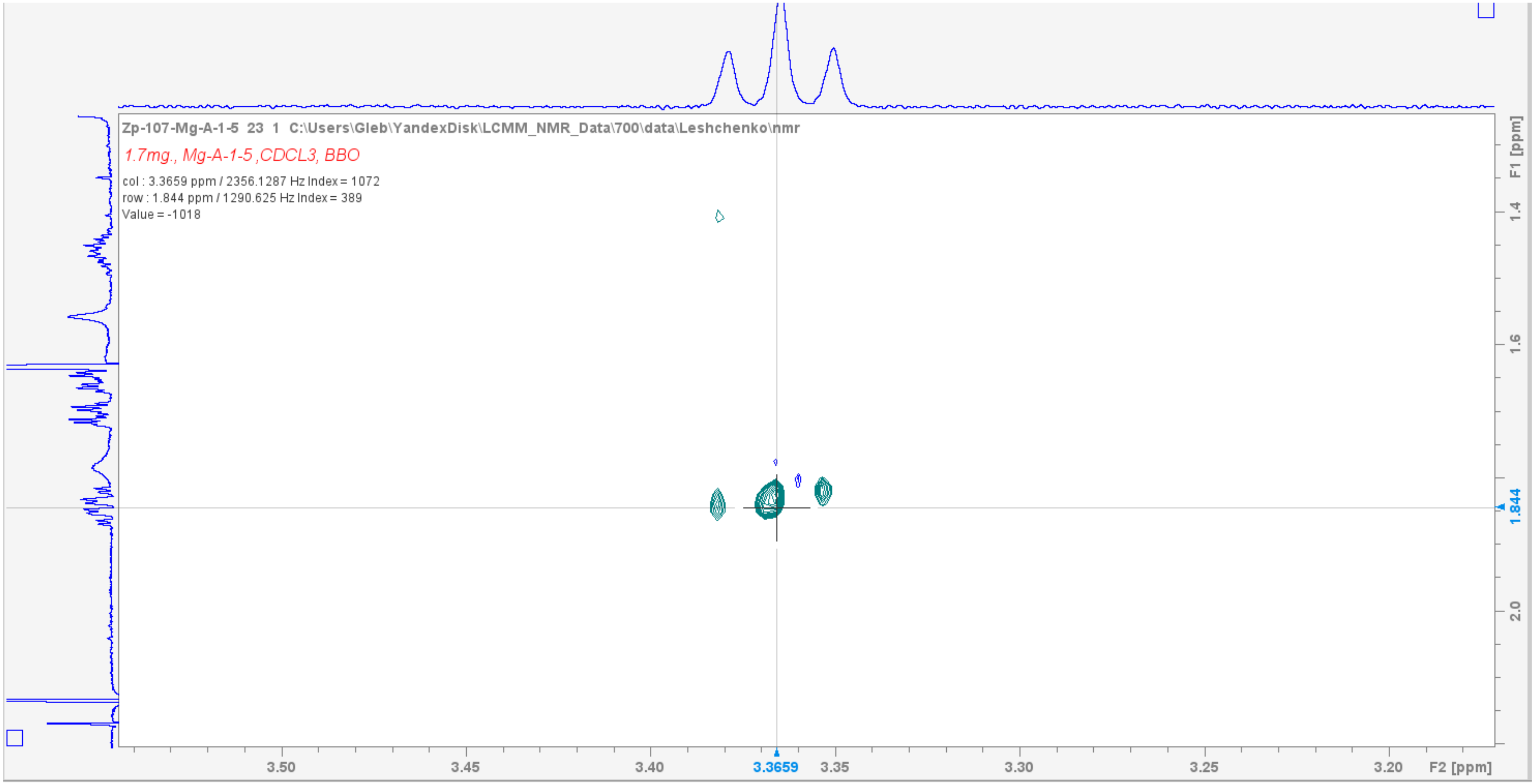
Zp-107-Mg-A-1-5 23 1 C:\Users\Gleb\YandexDisk\LCMM_NMR_Data\700\data\Leshchenko\nmr
1.7mg., Mg-A-1-5 ,CDCL3, BBO
col : 3.3659 ppm / 2356.1287 Hz Index = 1072
row : 1.844 ppm / 1290.625 Hz Index = 389
Value = -1018
F1 [ppm]
1.4
1.6
1.844
2.0
3.50
3.45
3.40
3.3659
3.35
3.30
3.25
3.20
F2 [ppm]

Figure S116. (**A**) Key COSY, (**B**) HMBC and (**C**) ROESY correlations of **12**

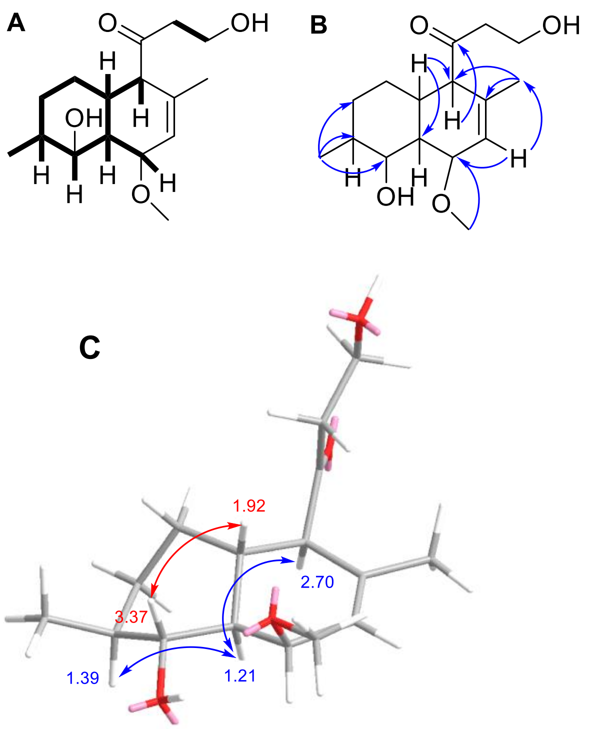

Figure S117. HRESIMS for **12**

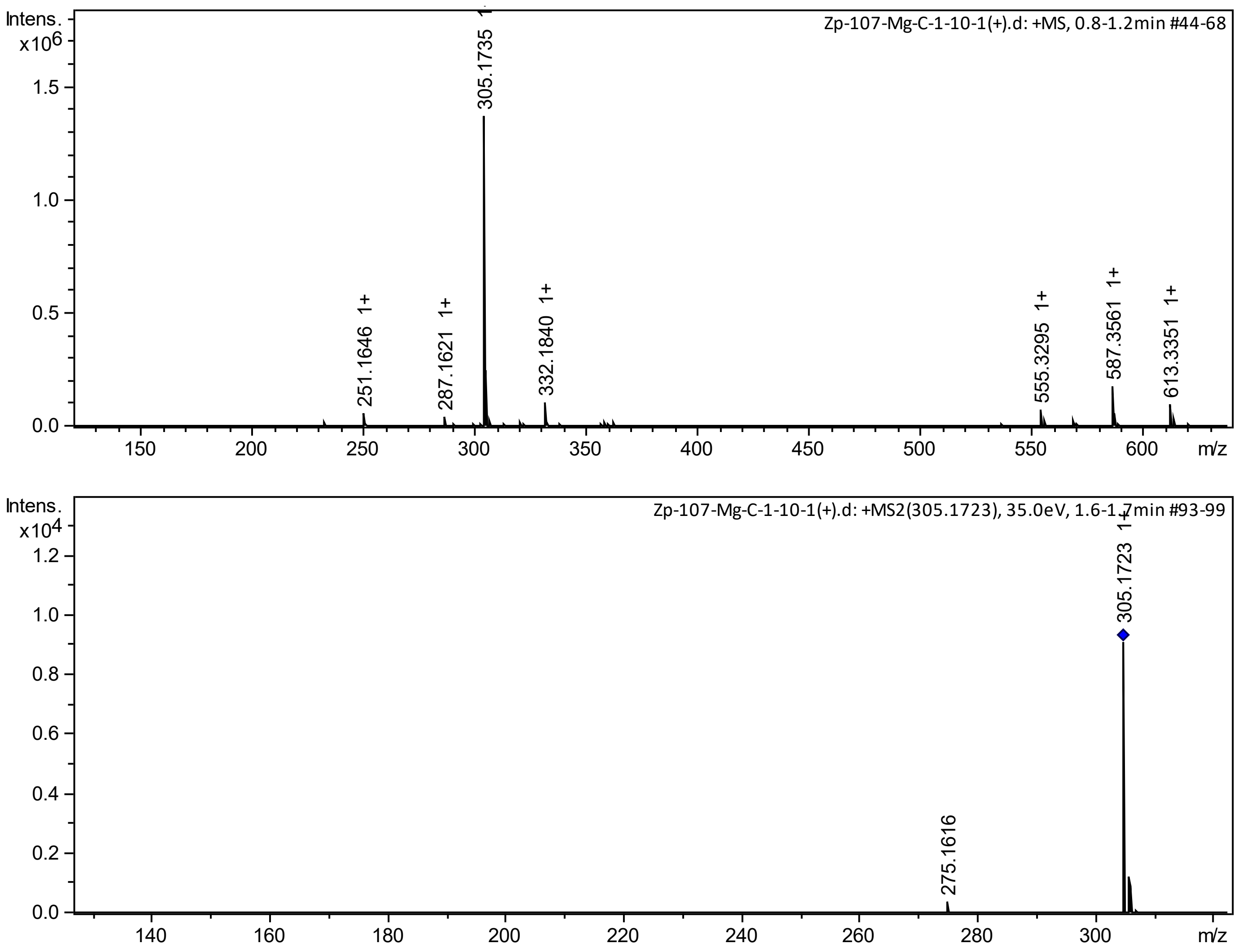

	meas	calc	Δ (ppm)
$[M+Na]^+$	305,1735	305,1723	-3,9

Figure S118. ^{1}H NMR spectrum of **12** measured at 700 MHz in $CDCl_3$

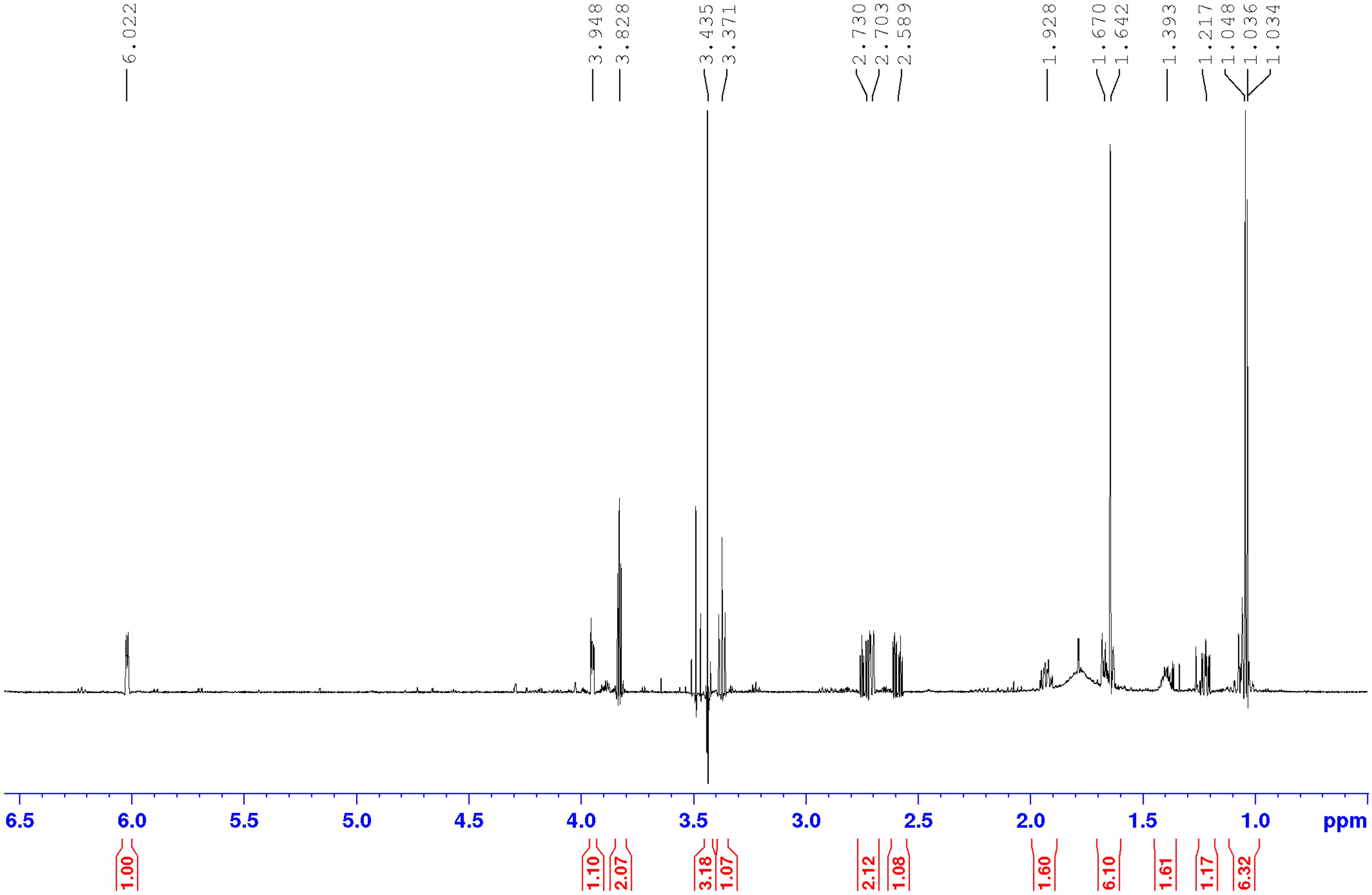

Figure S119. ^{13}C NMR spectrum of **12** measured at 176 MHz in $CDCl_3$

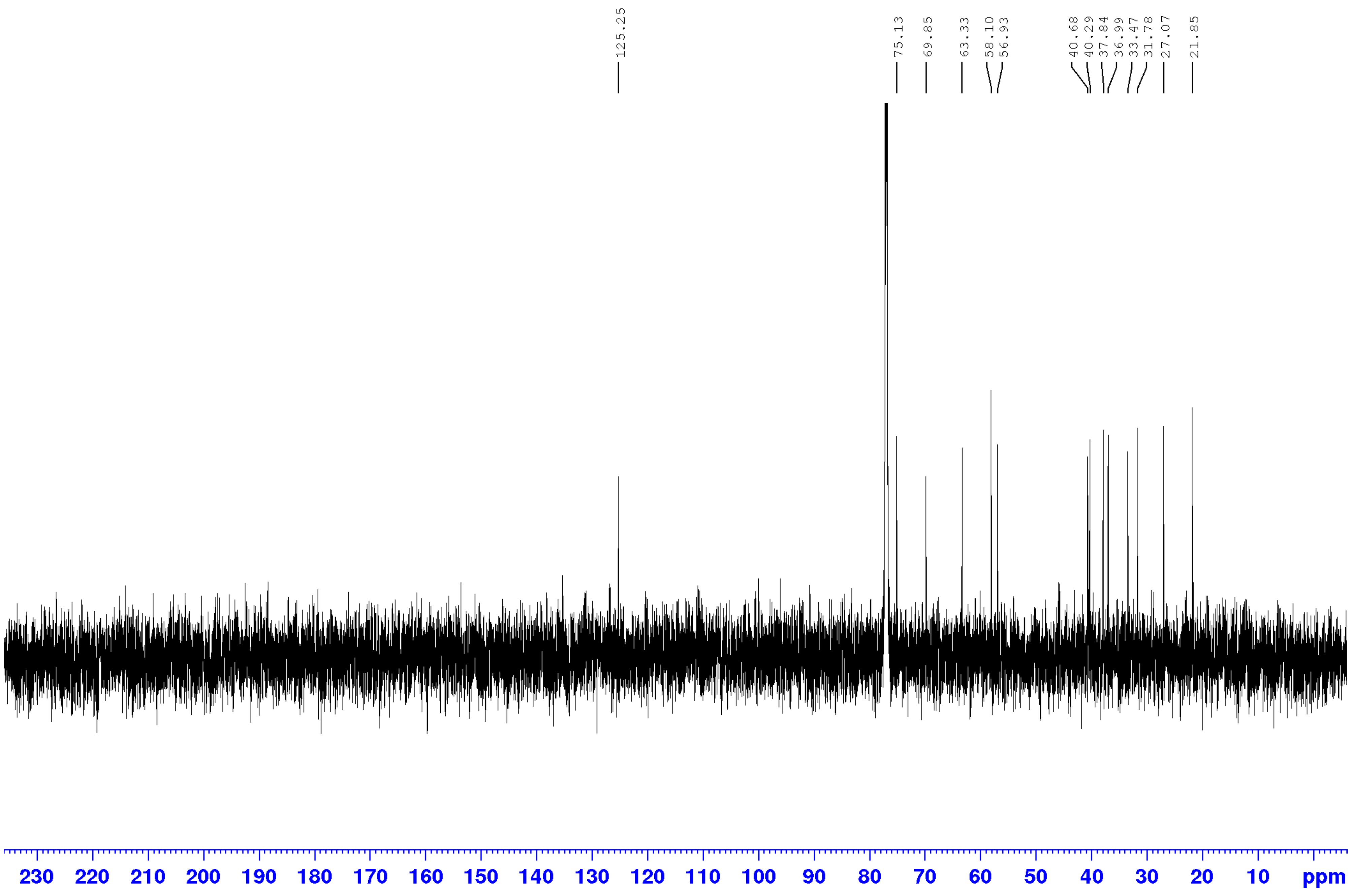

Figure S120. DEPT-135 spectrum of **12** measured at 176 MHz in $CDCl_3$

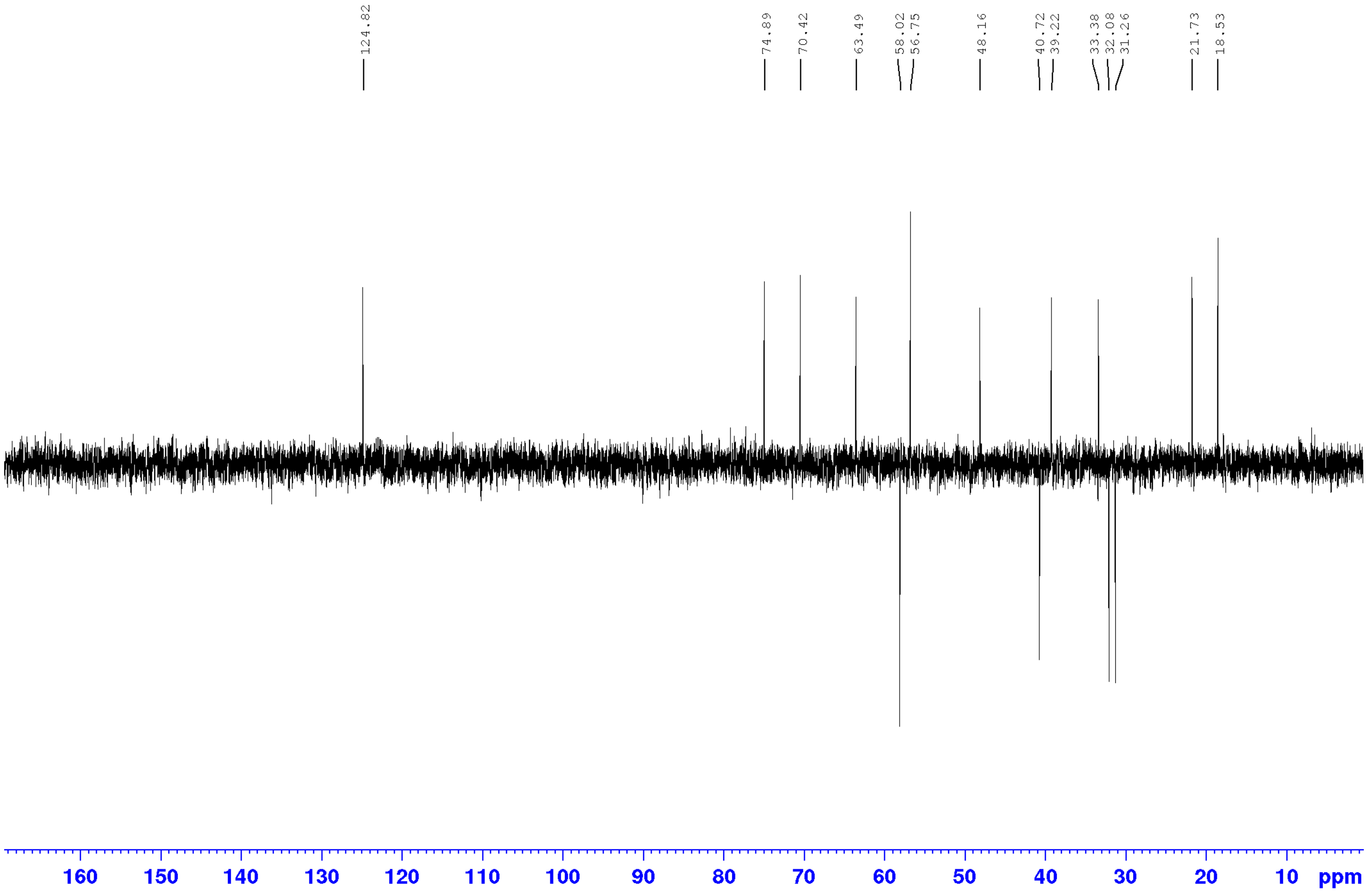

Figure S121. HSQC spectrum of **12** measured in $CDCl_3$

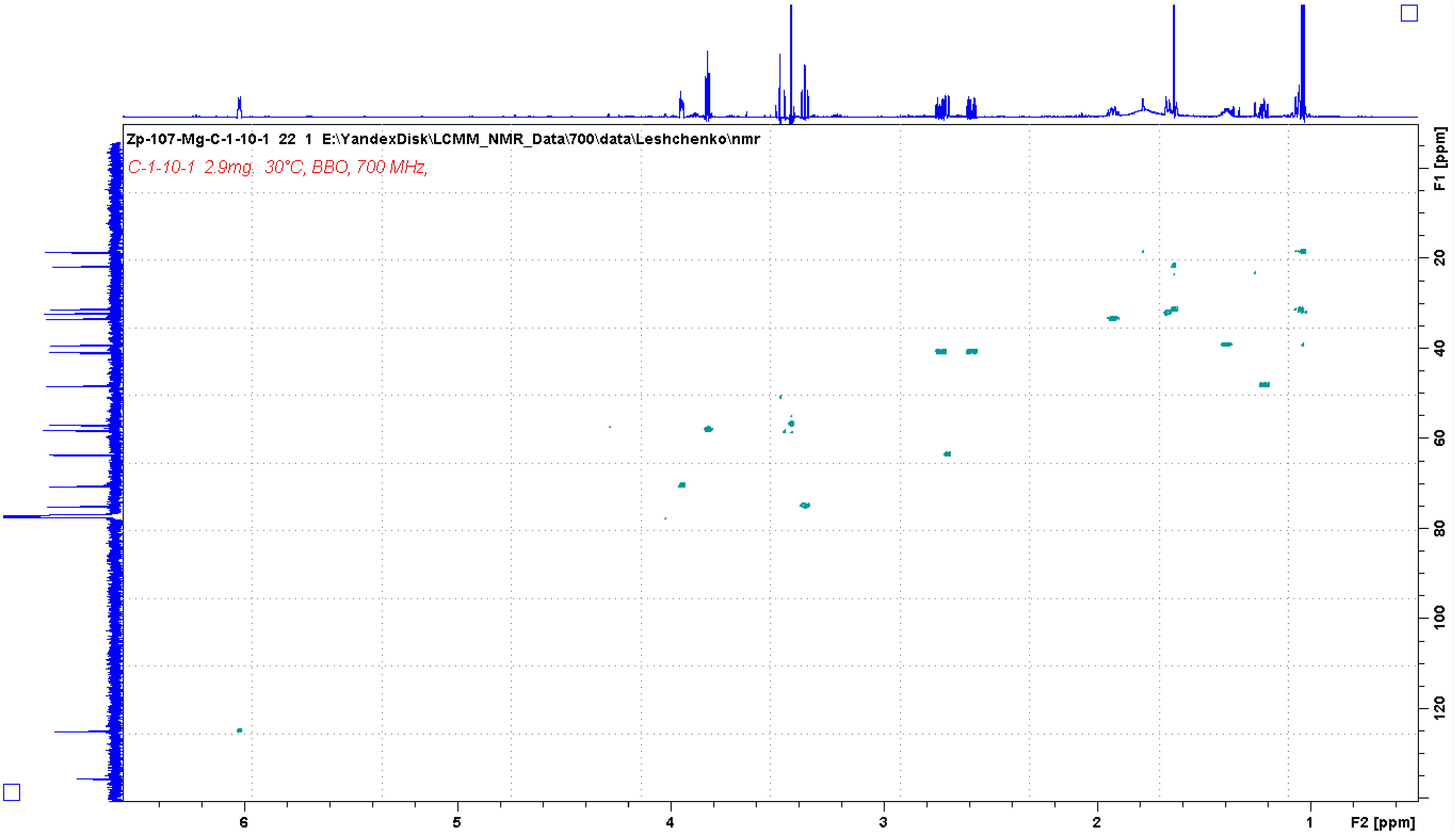

Figure S122. COSY spectrum of **12** measured in $CDCl_3$

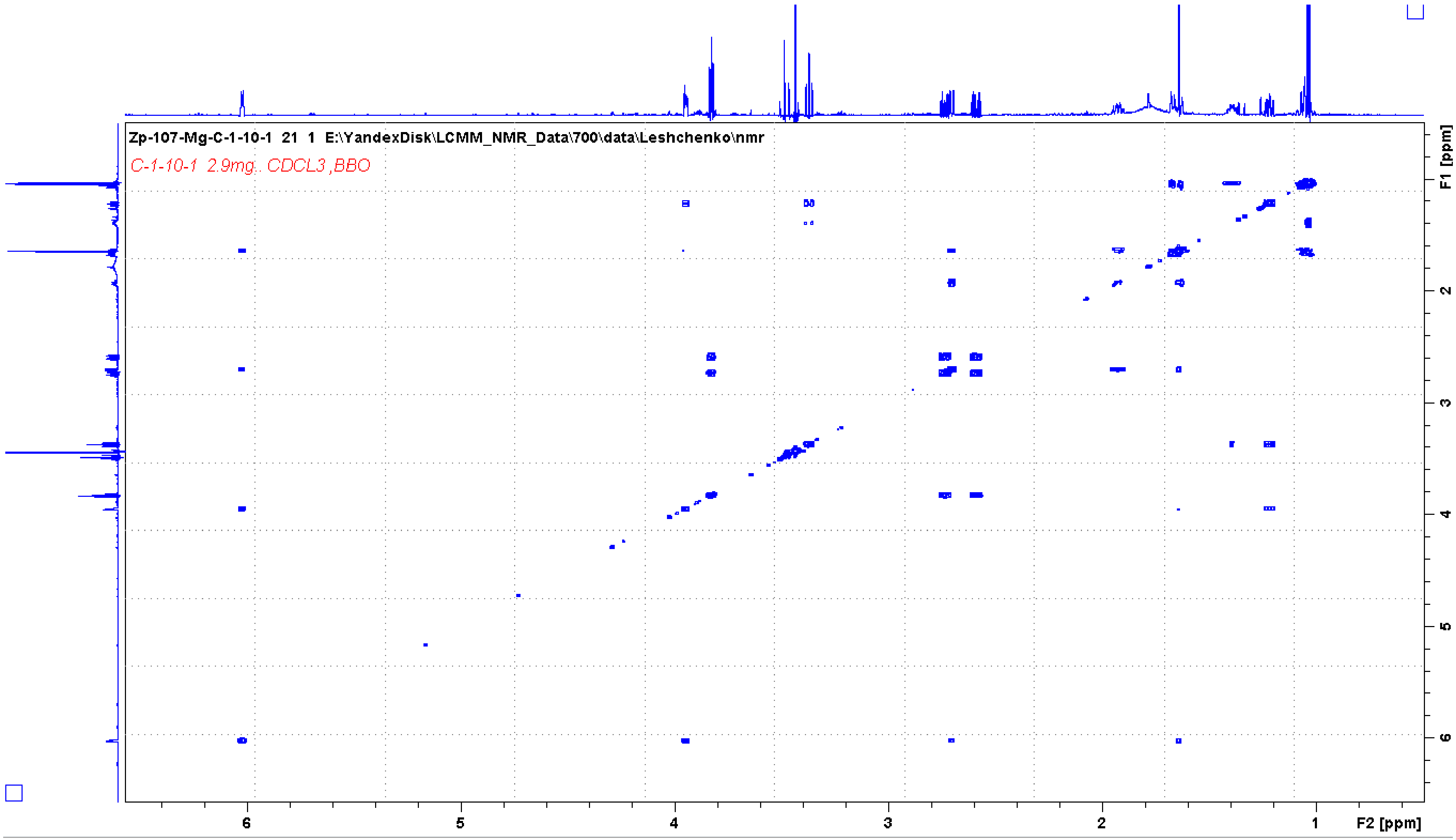

Figure S123. HMBC spectrum of **12** measured in $CDCl_3$

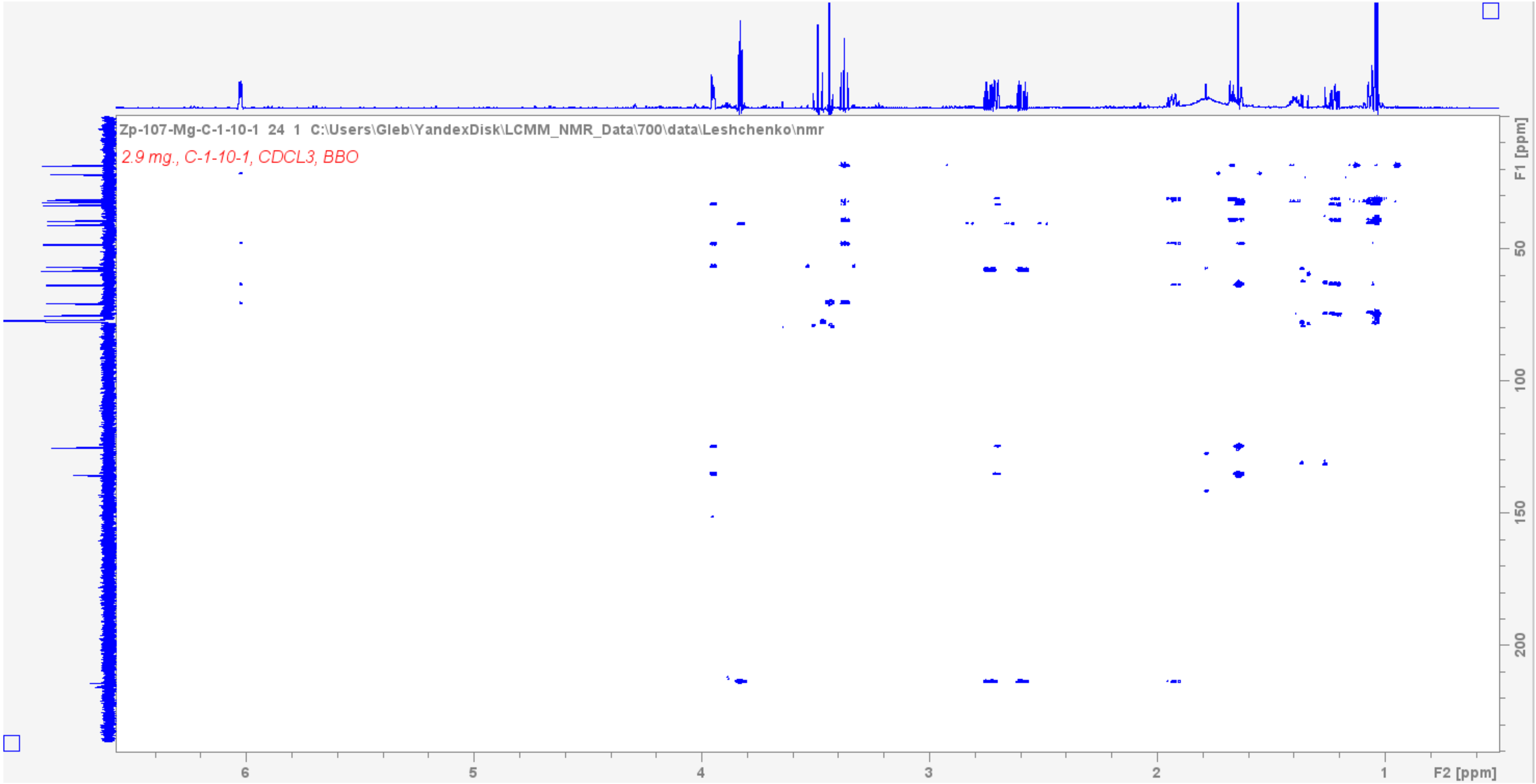

Figure S124. ROESY spectrum of **12** measured in $CDCl_3$

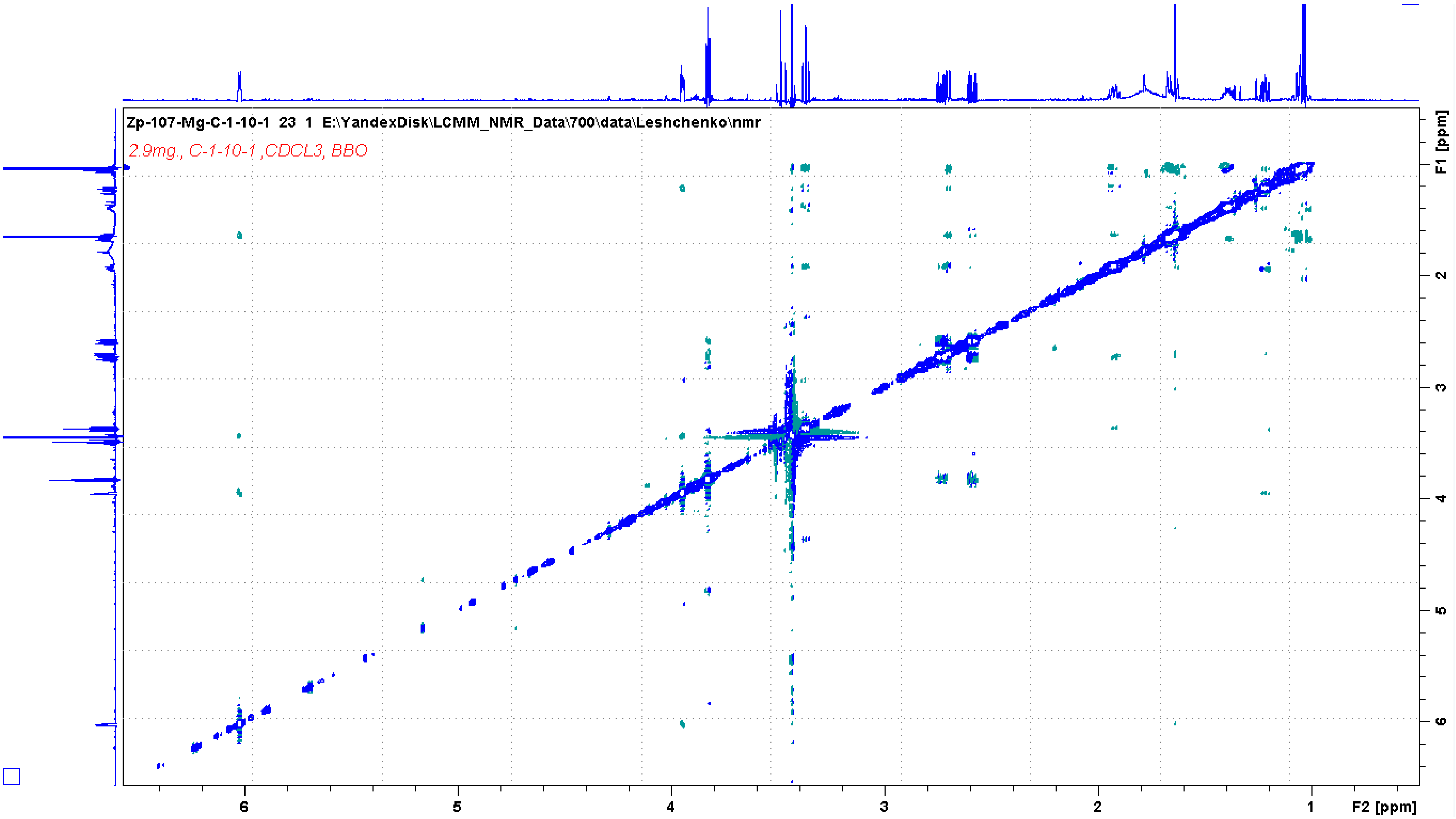

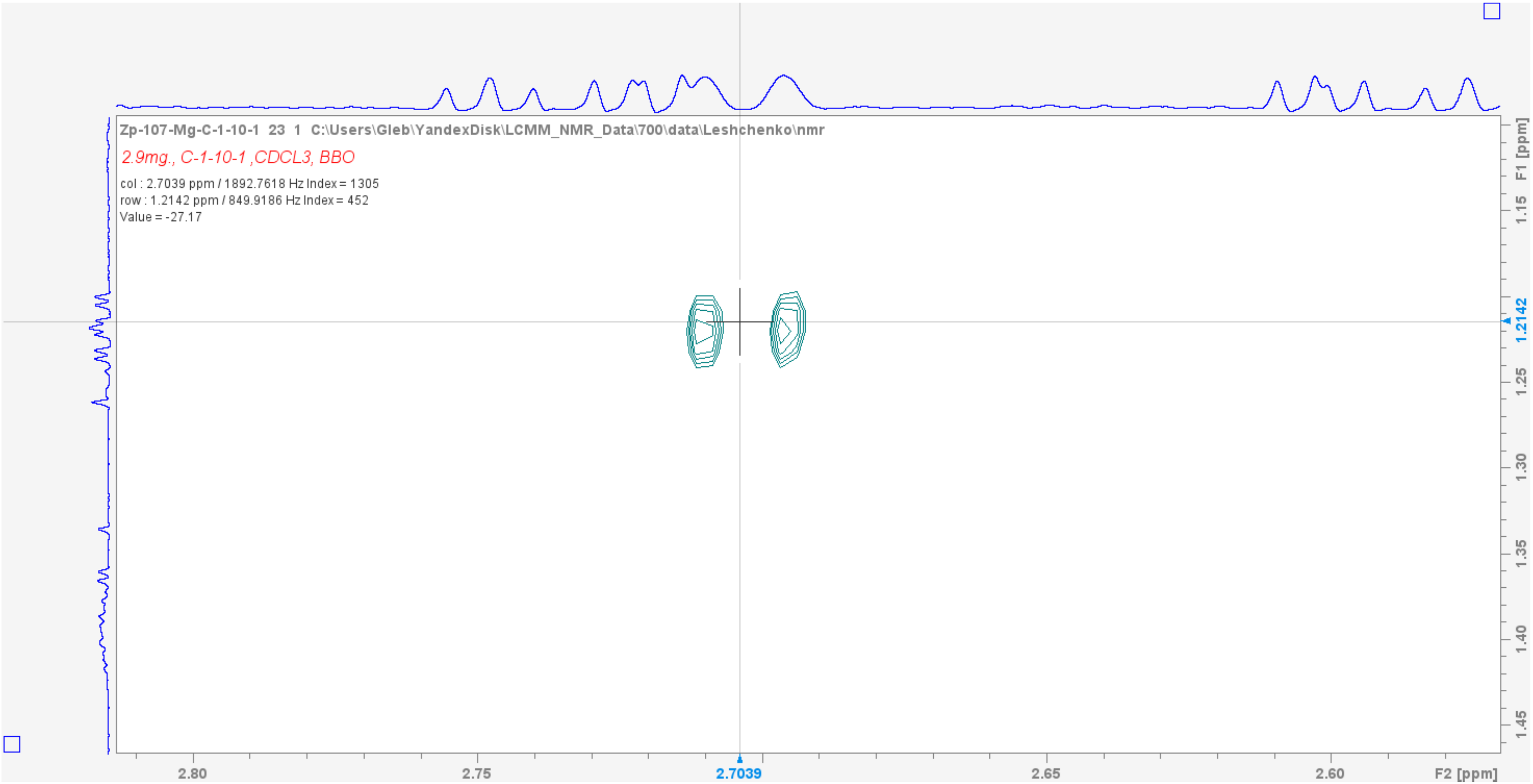

Zp-107-Mg-C-1-10-1 23 1 C:\Users\Gleb\YandexDisk\LCMM_NMR_Data\700\data\Leshchenko\nmr
2.9mg., C-1-10-1 ,CDCL3, BBO
col : 2.7039 ppm / 1892.7618 Hz Index = 1305
row : 1.2142 ppm / 849.9186 Hz Index = 452
Value = -27.17
2.80
2.75
2.7039
2.65
2.60
F2 [ppm]
F1 [ppm]
1.15
1.2142
1.25
1.30
1.35
1.40
1.45

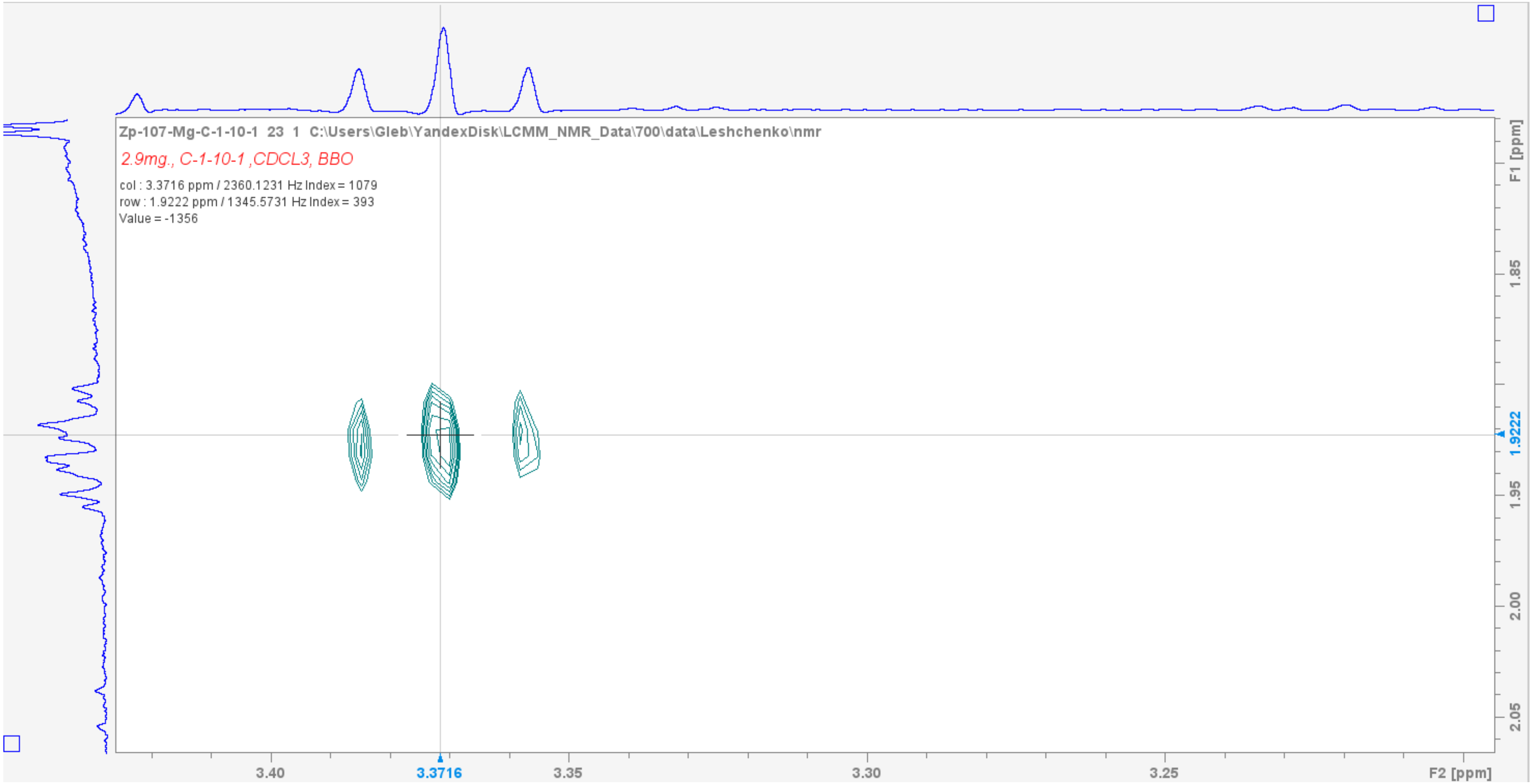
Zp-107-Mg-C-1-10-1 23 1 C:\Users\Gleb\YandexDisk\LCMM_NMR_Data\700\data\Leshchenko\nmr
2.9mg., C-1-10-1 ,CDCL3, BBO
col : 3.3716 ppm / 2360.1231 Hz Index = 1079
row : 1.9222 ppm / 1345.5731 Hz Index = 393
Value = -1356
3.40
3.3716
3.35
3.30
3.25
F2 [ppm]
F1 [ppm]
1.85
1.9222
1.95
2.00
2.05

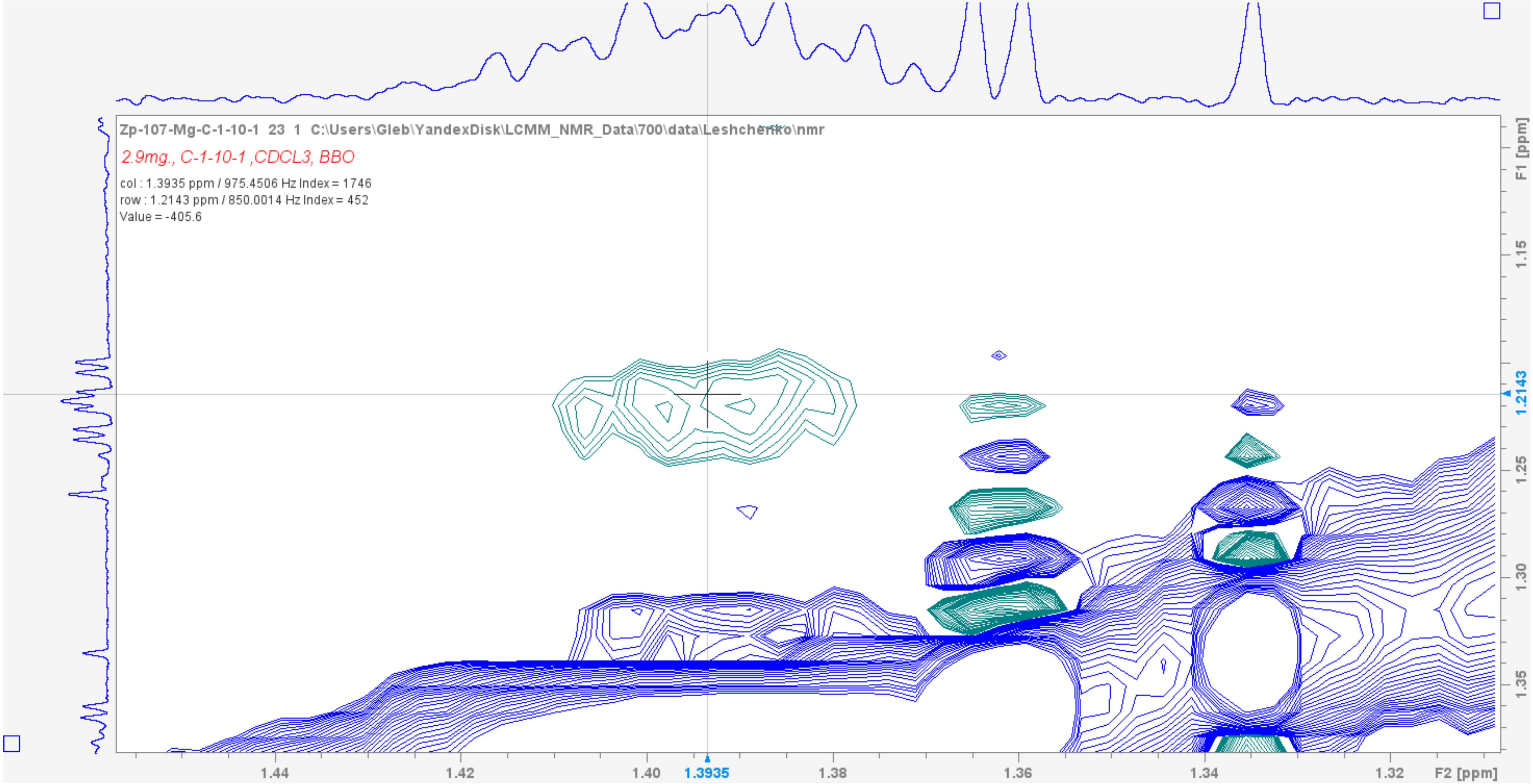

Zp-107-Mg-C-1-10-1 23 1 C:\Users\Gleb\YandexDisk\LCMM_NMR_Data\700\data\Leshchenko\nmr
2.9mg., C-1-10-1 ,CDCL3, BBO
col : 1.3935 ppm / 975.4506 Hz Index = 1746
row : 1.2143 ppm / 850.0014 Hz Index = 452
Value = -405.6
1.44
1.42
1.40
1.3935
1.38
1.36
1.34
1.32
F2 [ppm]
F1 [ppm]
1.15
1.2143
1.25
1.30
1.35

Figure S125. UV spectrum of **12** measured in MeOH

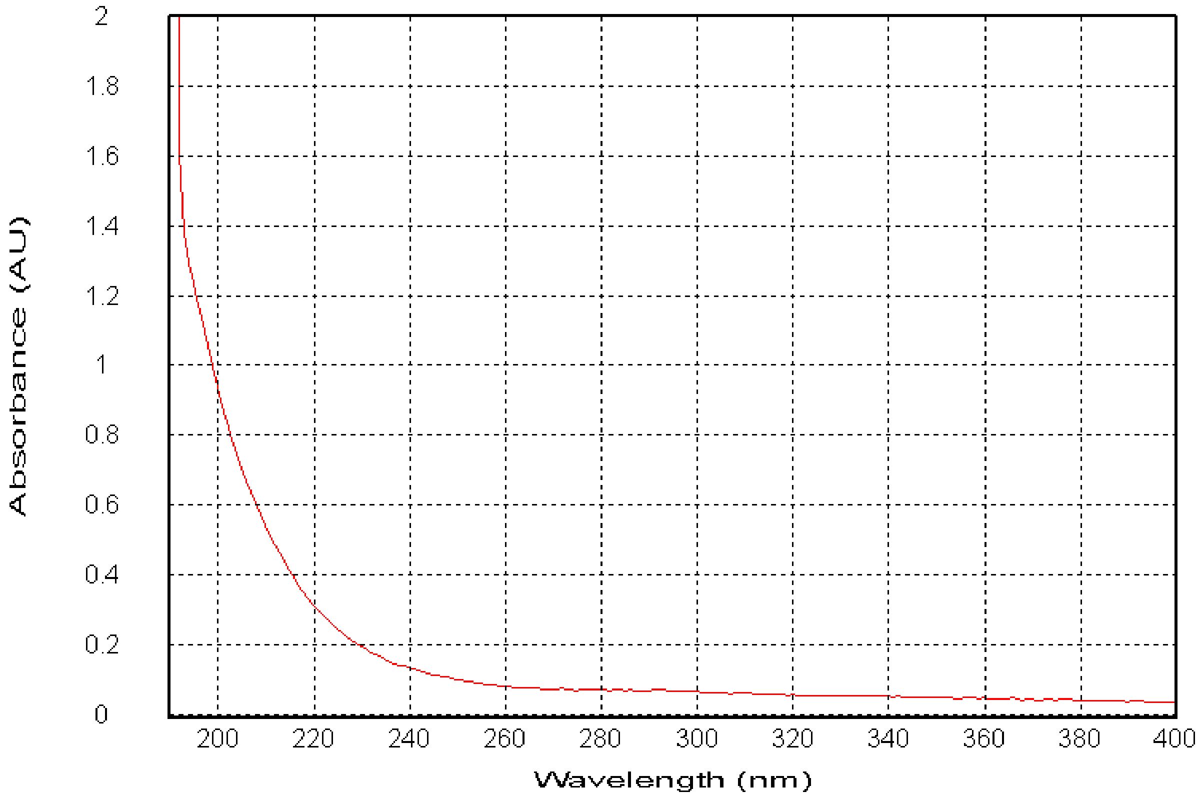

Figure S126. CD spectrum of **12** measured in MeOH

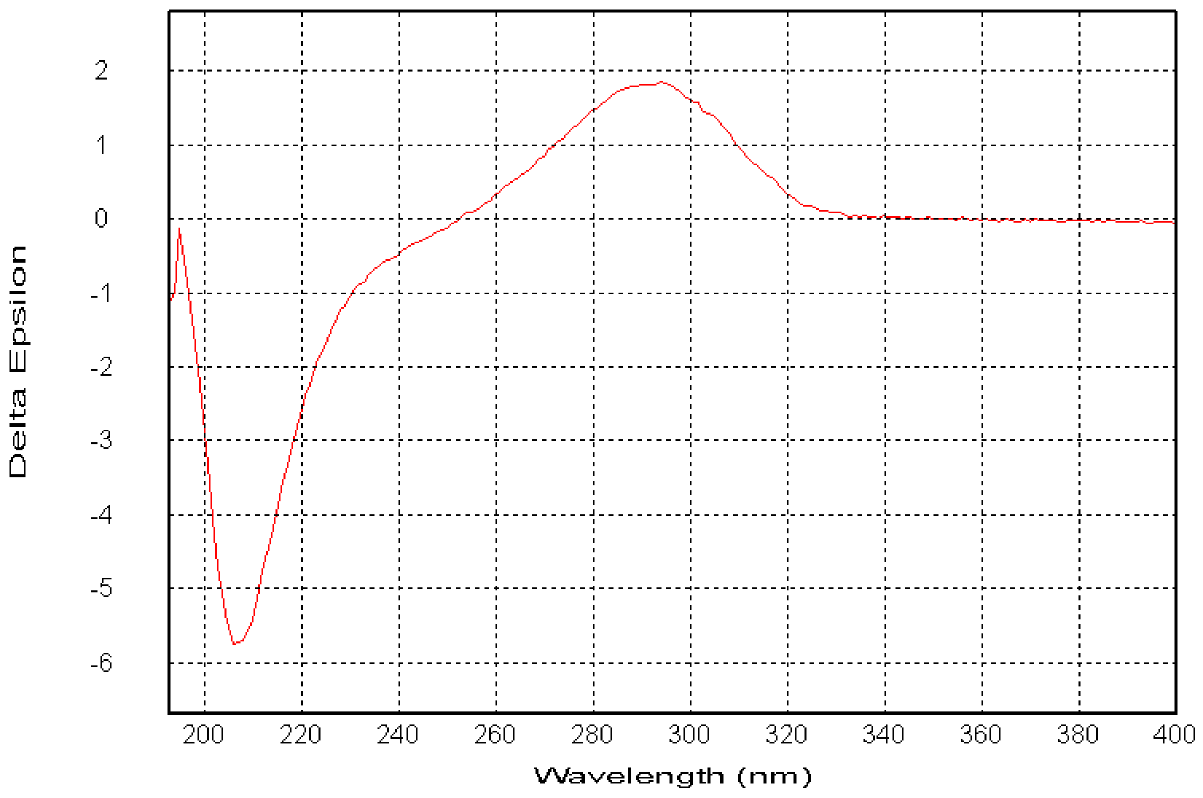

Figure S127. (**A**) Key COSY, (**B**) HMBC and (**C**) ROESY correlations of **13**

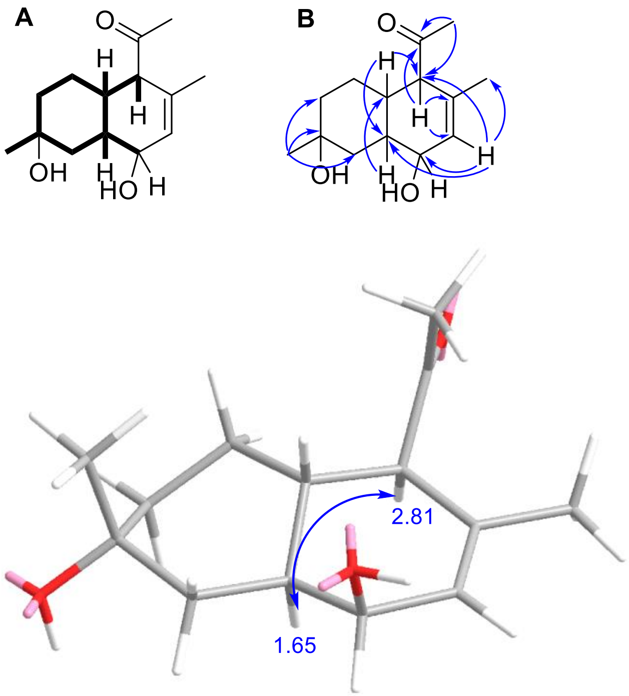

Figure S128. HRESIMS for **13**

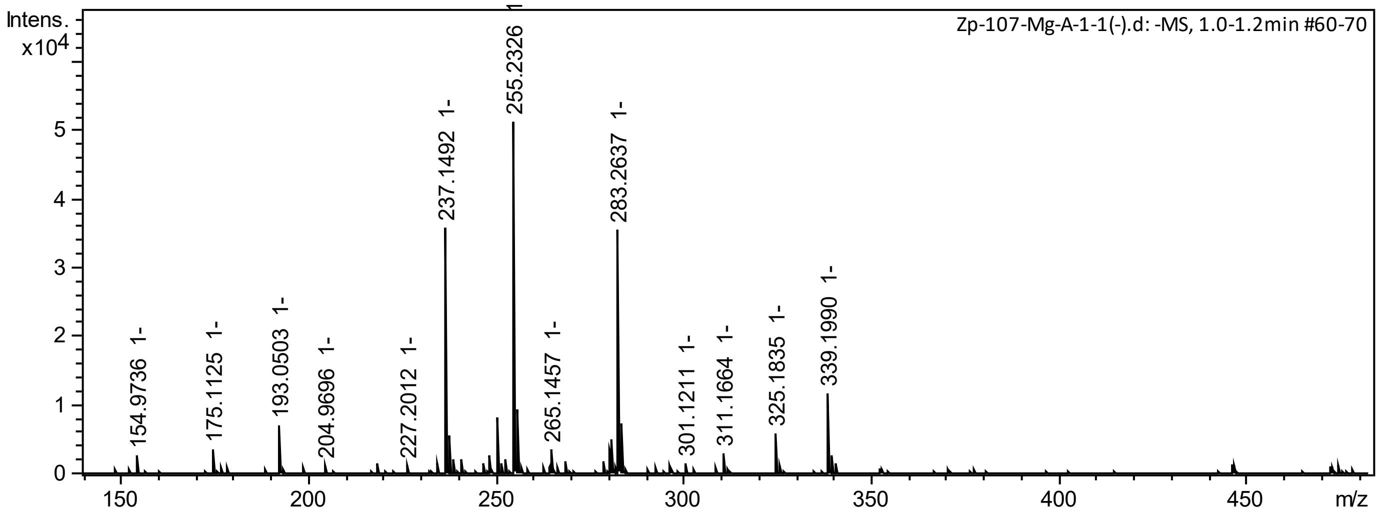

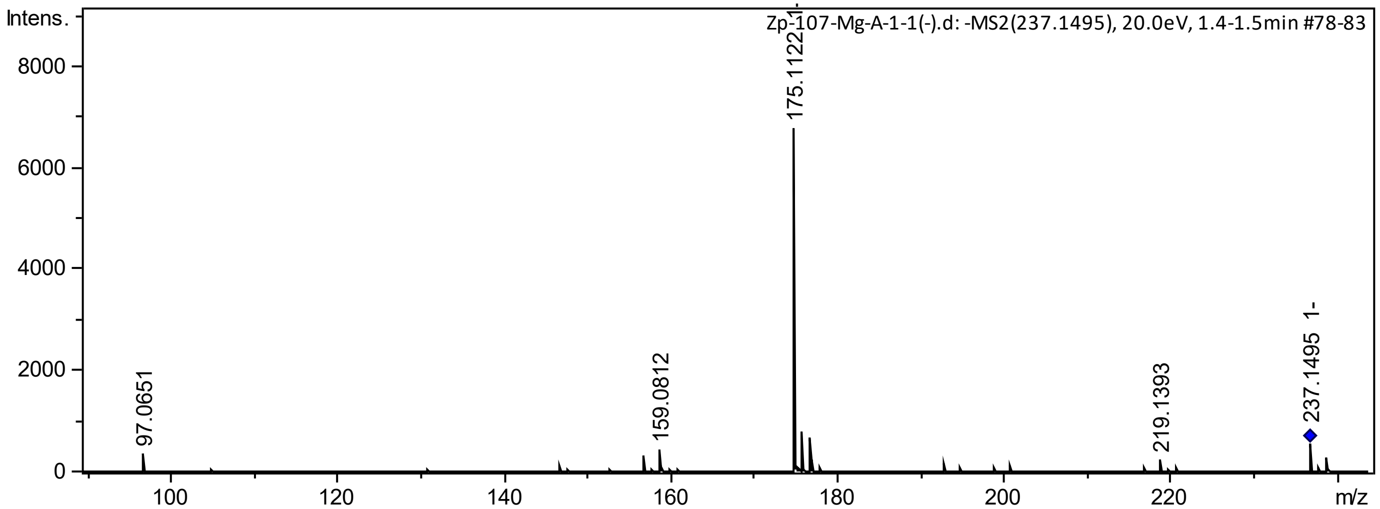
Intens.
Zp-107-Mg-A-1-1(-).d: -MS2(237.1495), 20.0eV, 1.4-1.5min #78-83
8000
6000
4000
2000
0
175.1122 1-
97.0651
159.0812
219.1393
237.1495 1-
100
120
140
160
180
200
220
m/z

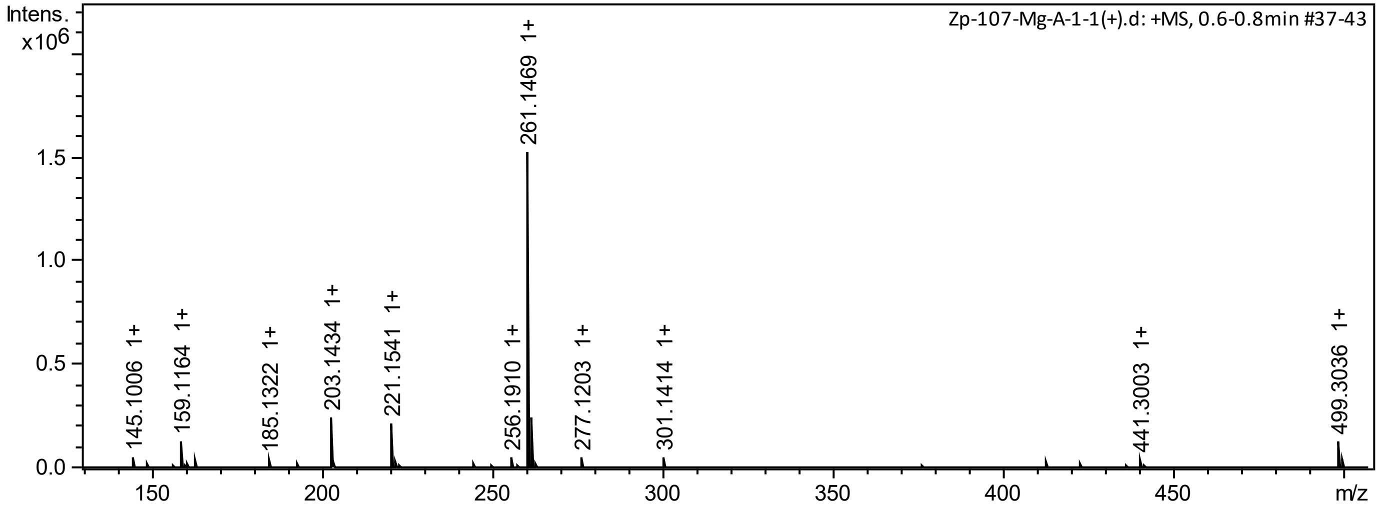
Intens.
x10^6
Zp-107-Mg-A-1-1(+).d: +MS, 0.6-0.8min #37-43
1.5
1.0
0.5
0.0
261.1469 1+
145.1006 1+
159.1164 1+
185.1322 1+
203.1434 1+
221.1541 1+
256.1910 1+
277.1203 1+
301.1414 1+
441.3003 1+
499.3036 1+
150
200
250
300
350
400
450
m/z

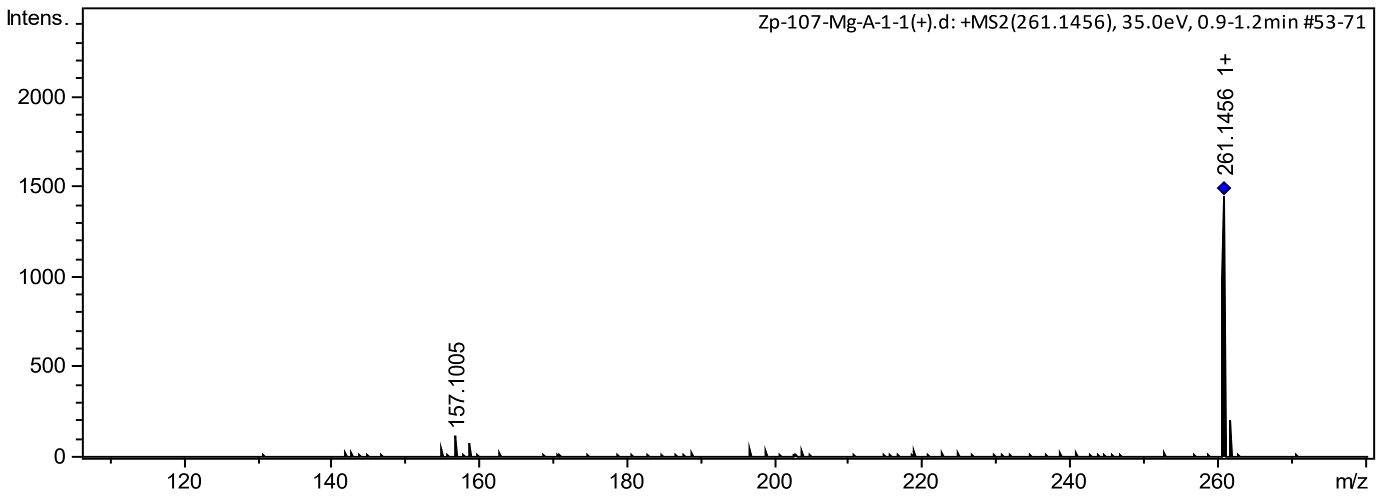

	meas	calc	Δ (ppm)
$[M-H]^-$	237,1492	237,1496	1,7
$[M+Na]^+$	261,1469	261,1461	-3,1

Figure S129. ^1H NMR spectrum of **13** measured at 700 MHz in $CDCl_3$

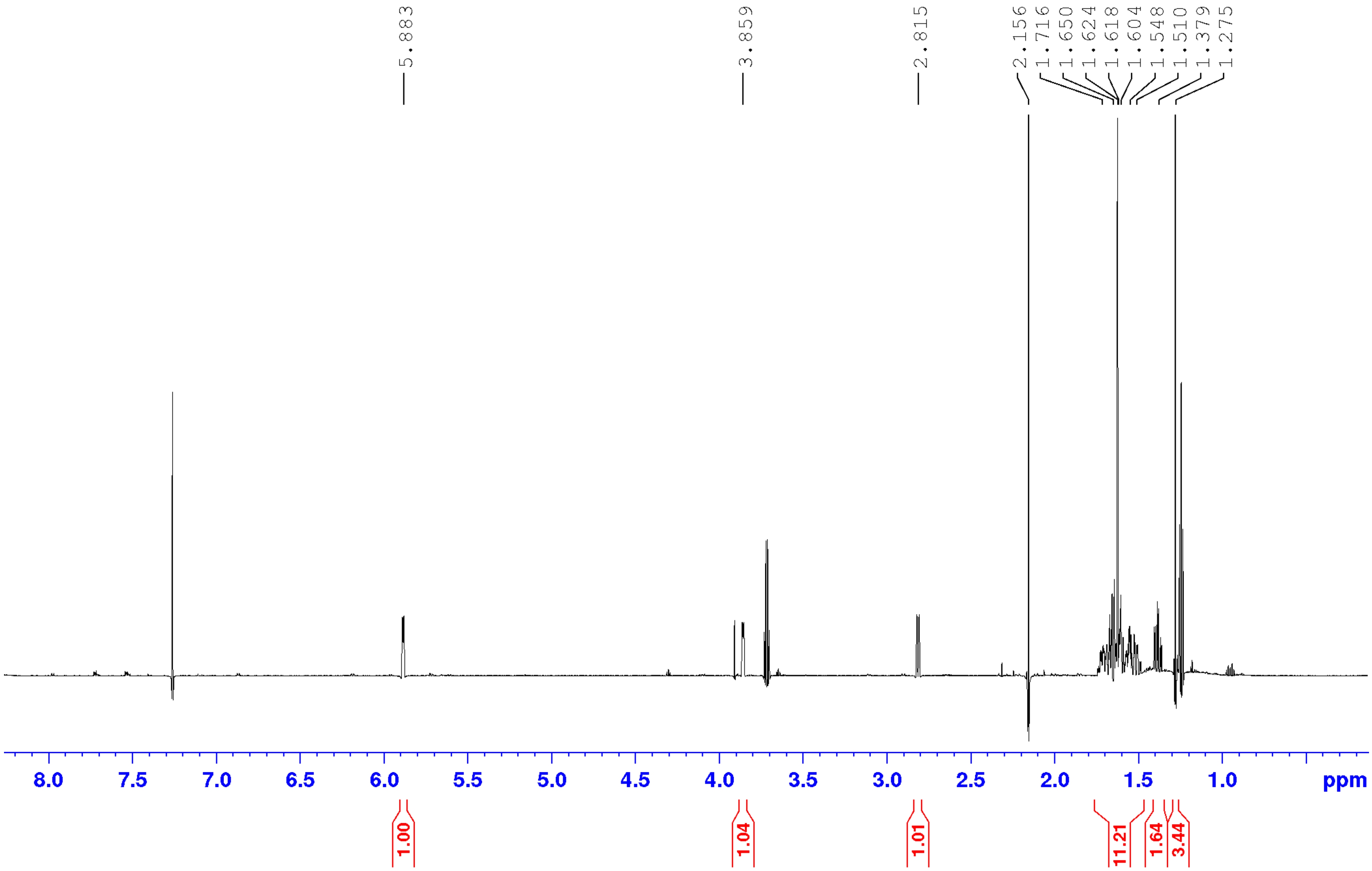

Figure S130. ^{13}C NMR spectrum of **13** measured at 176 MHz in $CDCl_3$

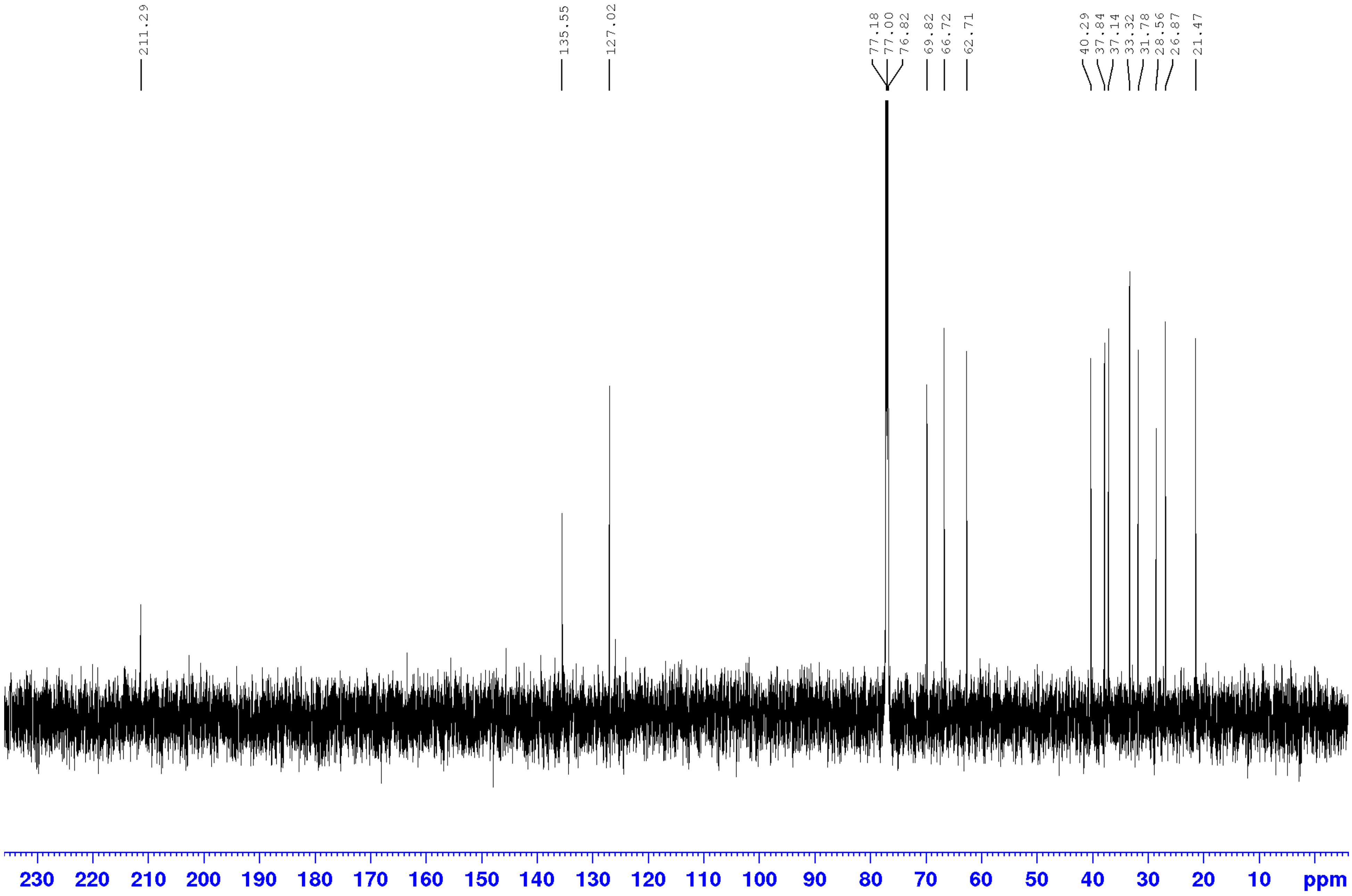

Figure S131. DEPT-135 spectrum of **13** measured at 176 MHz in $CDCl_3$

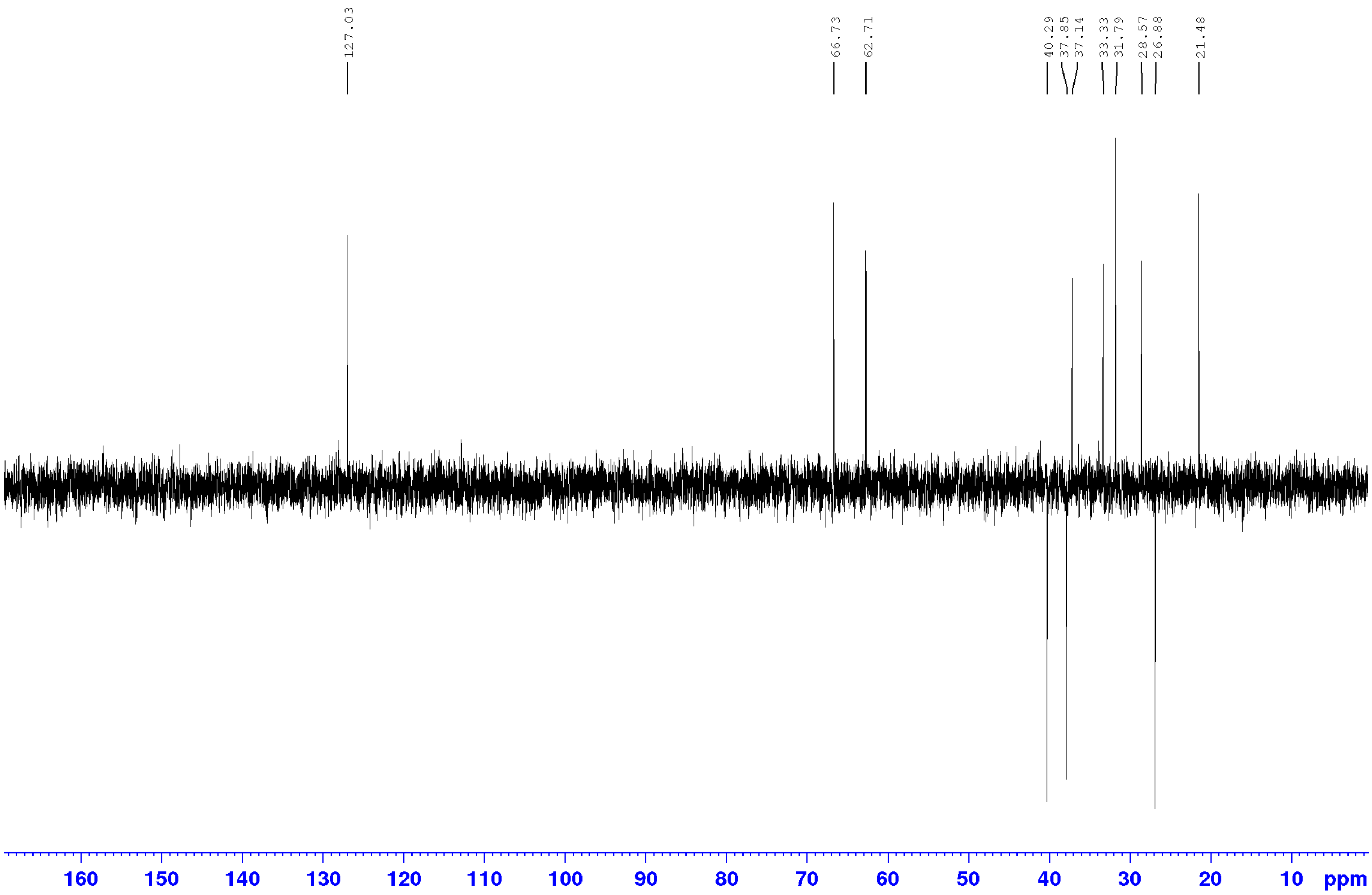

Figure S132. HSQC spectrum of **13** measured in $CDCl_3$

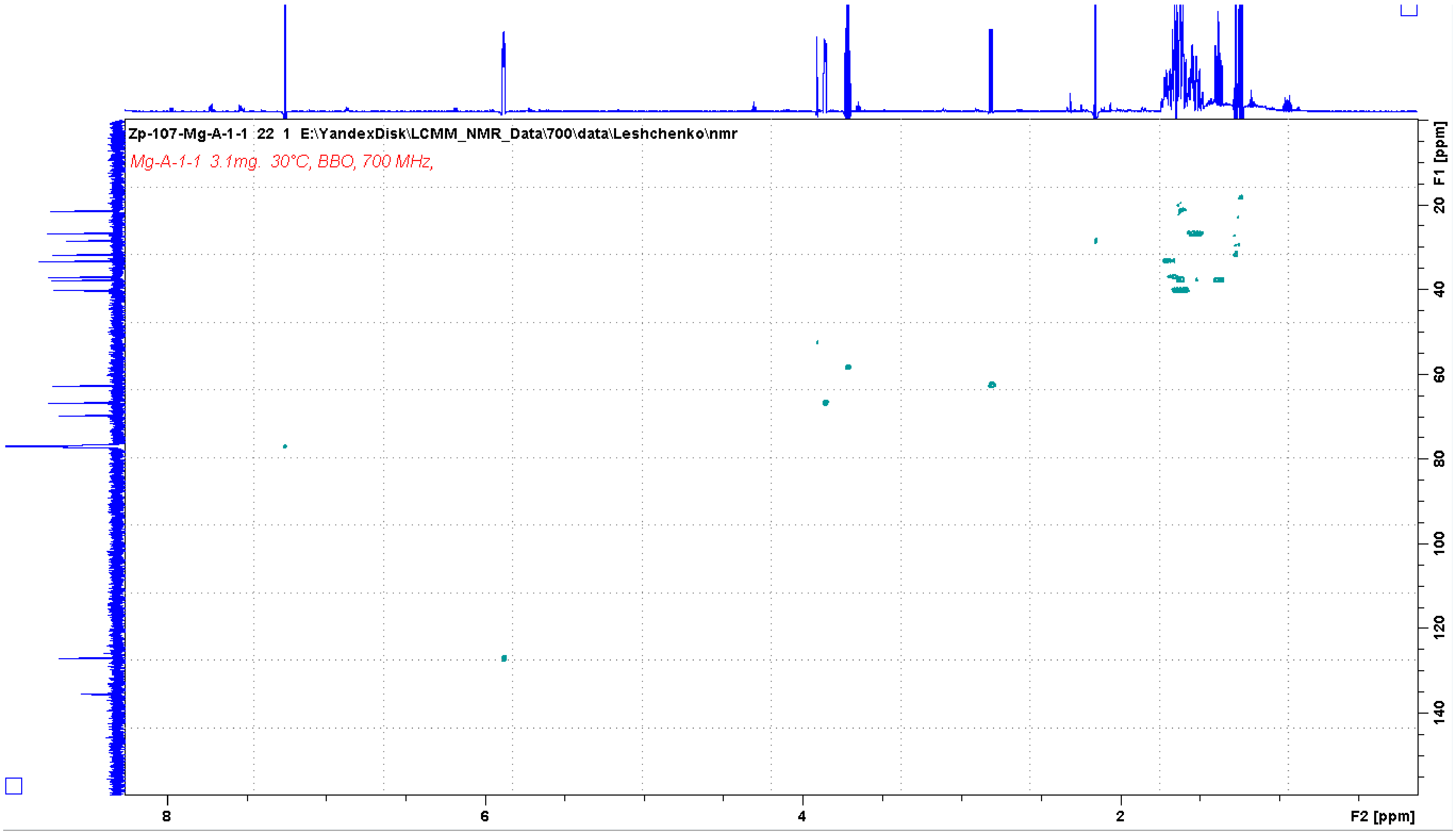

Figure S133. COSY spectrum of **13** measured in $CDCl_3$

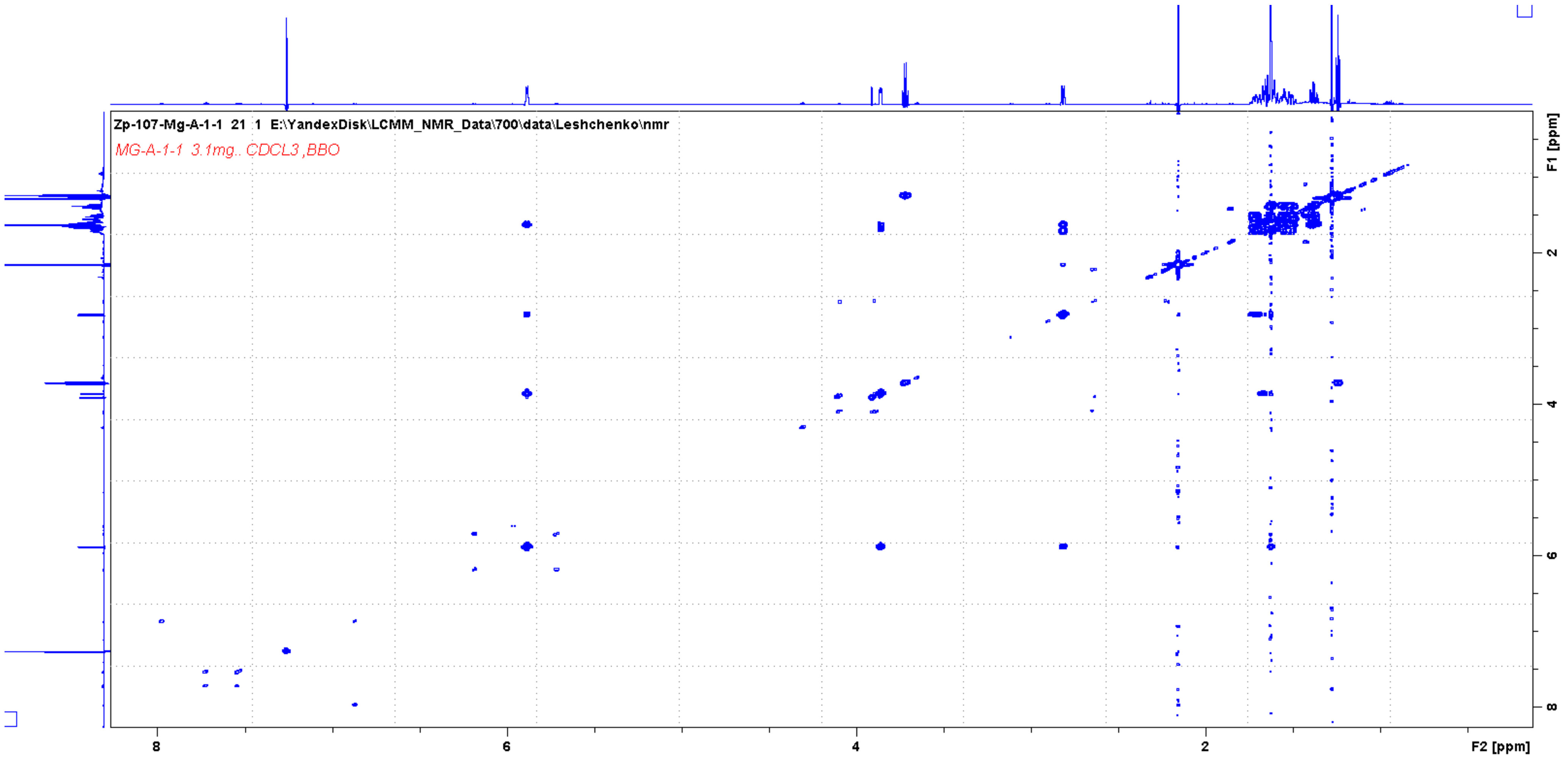

Figure S134. HMBC spectrum of **13** measured in $CDCl_3$

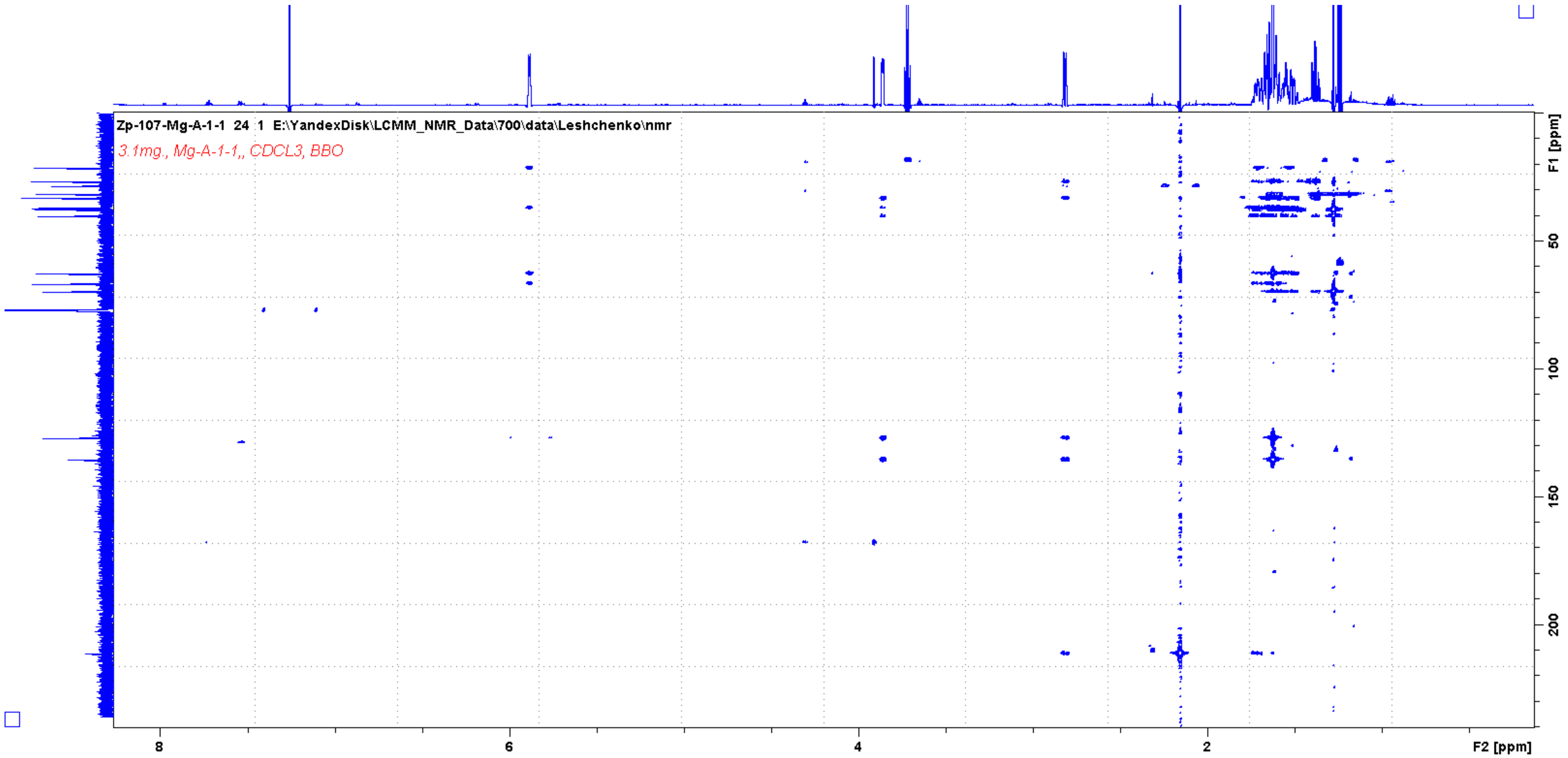

Figure S135. ROESY spectrum of **13** measured in $CDCl_3$

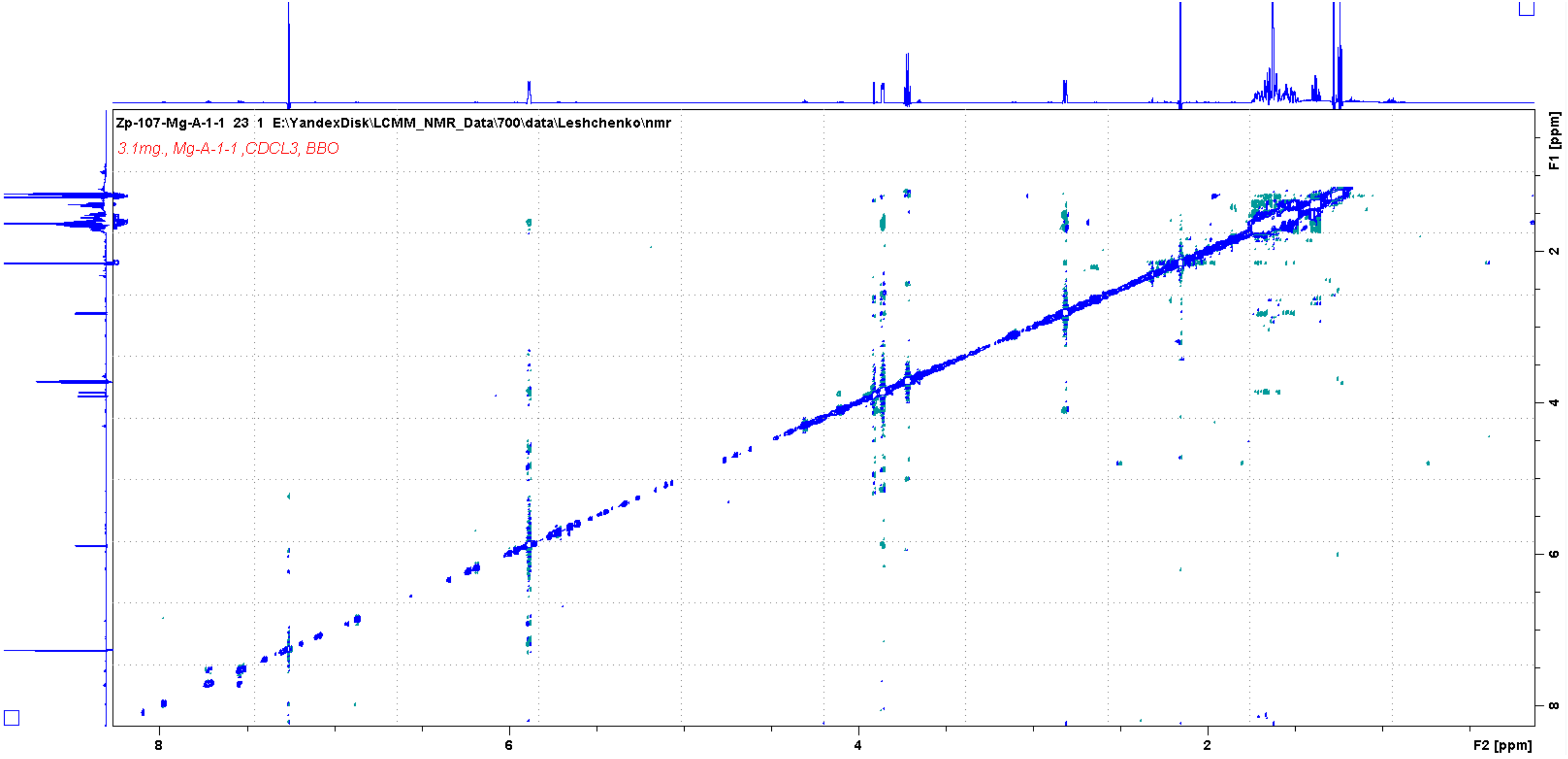

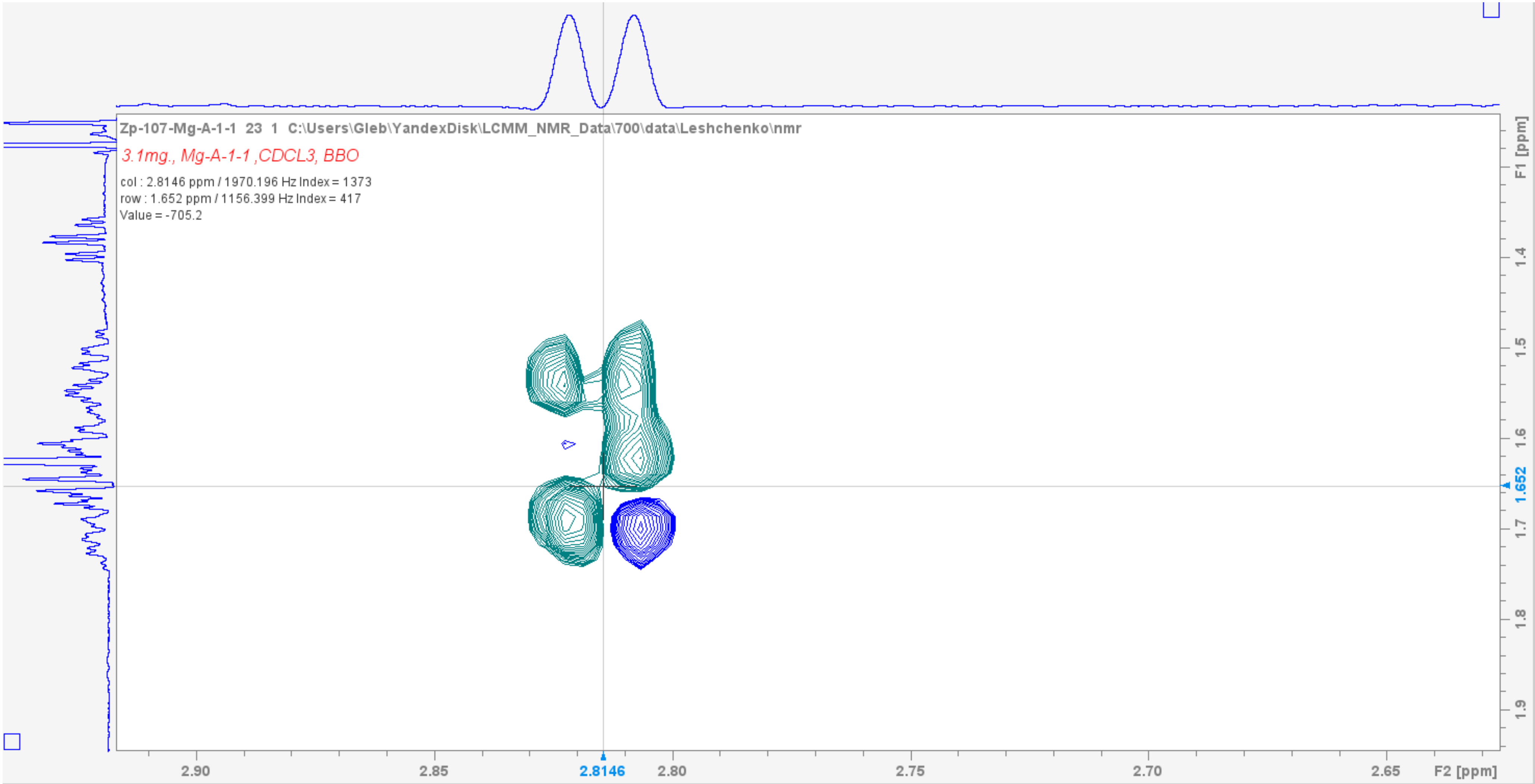

Zp-107-Mg-A-1-1 23 1 C:\Users\Gleb\YandexDisk\LCMM_NMR_Data\700\data\Leshchenko\nmr
3.1mg., Mg-A-1-1 ,CDCL3, BBO
col : 2.8146 ppm / 1970.196 Hz Index = 1373
row : 1.652 ppm / 1156.399 Hz Index = 417
Value = -705.2
2.90
2.85
2.8146
2.80
2.75
2.70
2.65
F2 [ppm]
F1 [ppm]
1.4
1.5
1.6
1.652
1.7
1.8
1.9

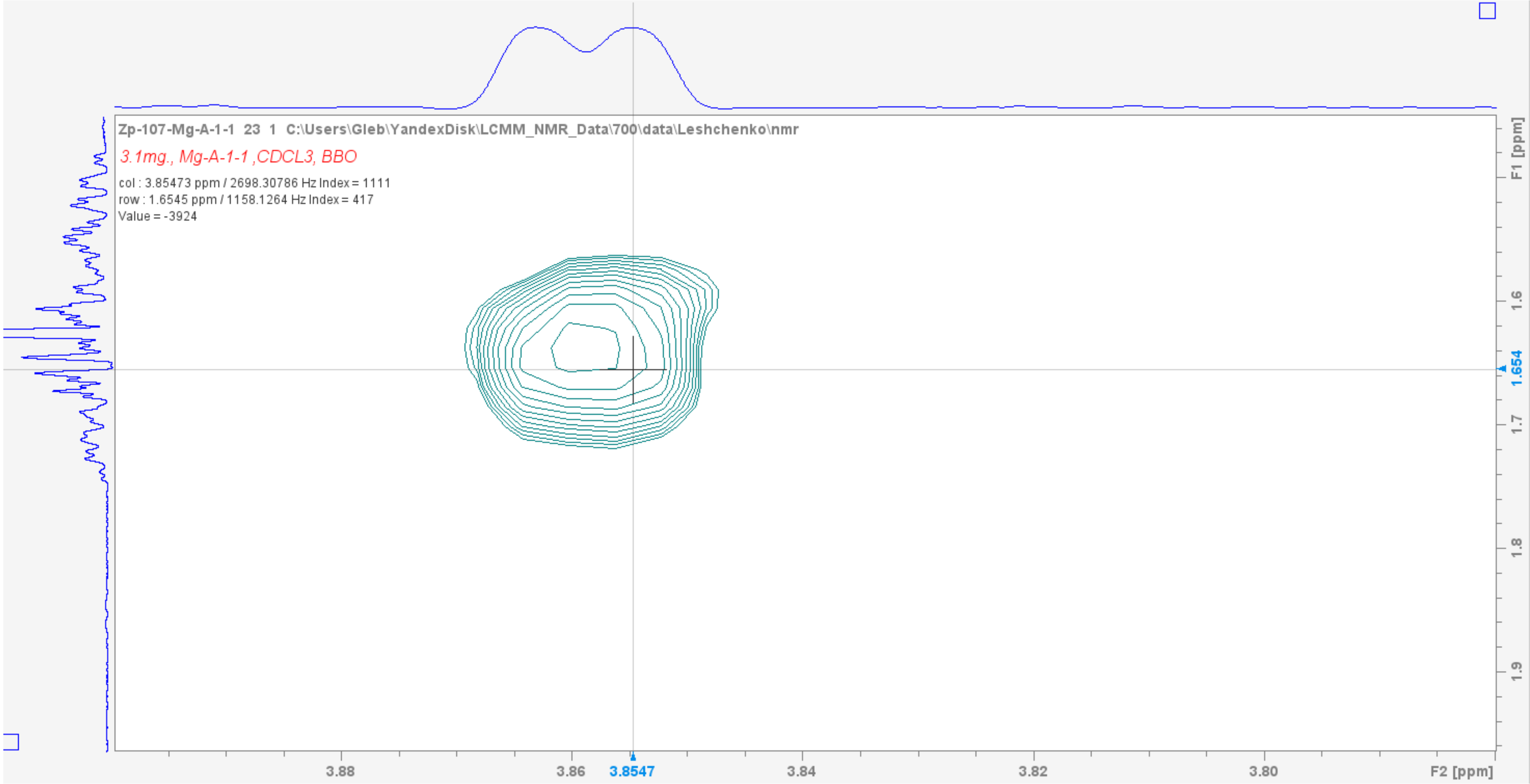

Zp-107-Mg-A-1-1 23 1 C:\Users\Gleb\YandexDisk\LCMM_NMR_Data\700\data\Leshchenko\nmr
3.1mg., Mg-A-1-1 ,CDCL3, BBO
col : 3.85473 ppm / 2698.30786 Hz Index = 1111
row : 1.6545 ppm / 1158.1264 Hz Index = 417
Value = -3924
3.88
3.86
3.8547
3.84
3.82
3.80
F2 [ppm]
F1 [ppm]
1.6
1.654
1.7
1.8
1.9

Figure S136. UV spectrum of **13** measured in MeOH

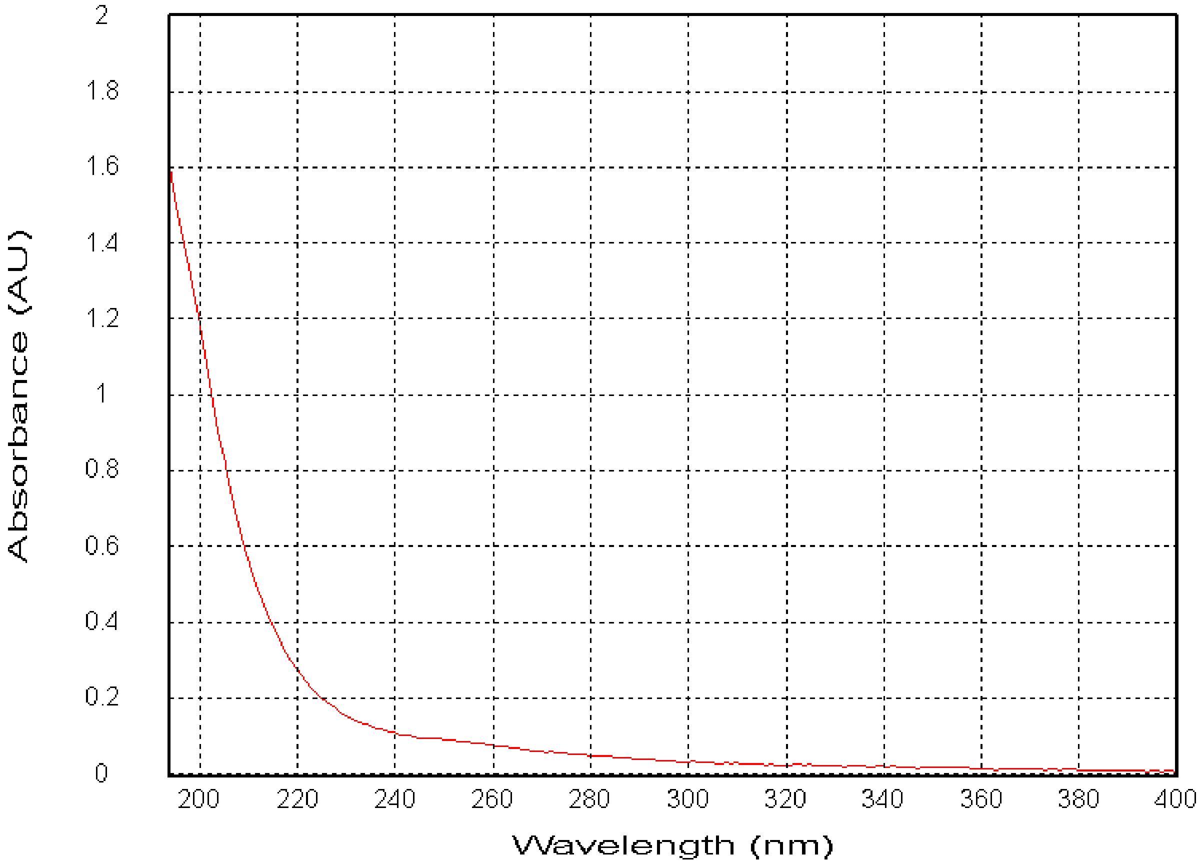

Figure S137. CD spectrum of **13** measured in MeOH

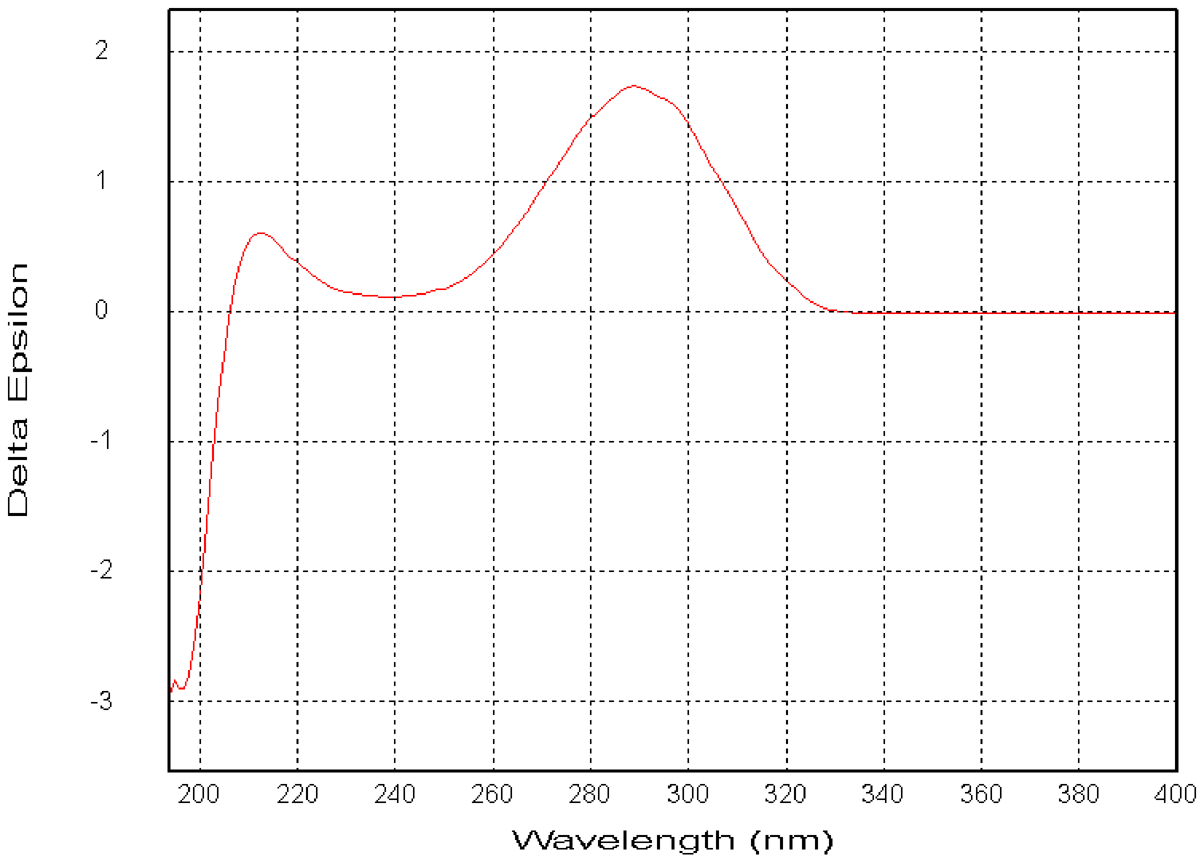

Table S1. Crystal data and structure refinement for **28**

Formula	$C_{15}H_{25}ClO_4 \cdot H_2O$
CCDC Number	2546332
Chemical formula	$C_{15}H_{25}ClO_4 \cdot H_2O$
Formula weight	322.81
Temperature (K)	120(2)
Crystal system, space group	Orthorhombic, $P2_12_12_1$
a, b, c (Å)	a = 7.2622(6), b = 7.7796(7), c = 28.257(3)
V (Å^3); Z	1596.5(2); 26
Density (calculated), g·cm^{-3}	1.343
μ (mm^{-1})	0.258
F(000)	696
Crystal size (mm^3)	0.380 × 0.230 × 0.120
θ range (°)	2.72 to 28.12
Range of h, k and l	-9<=h<=9, -10<=k<=10, -37<=l<=35
Reflections collected/independent	14521/3897 [$R_{(int)}$ = 0.0228]
Coef. of Transmission T_{min}; T_{max}	0.8035; 0.8621
Completeness to theta = 25.242°	99.9 %
Data / restraints / parameters	3897 / 0 / 201
Goof	1.049
Final R indices [I>2sigma(I)]	R_1 = 0.0259, wR_2 = 0.0630
R indices (all data)	R_1 = 0.0279, wR_2 = 0.0639
Absolute structure parameter	0.033(17)
$\Delta\rho_{min}$, $\Delta\rho_{max}$ (e/Å^3)	-0.162, 0.255

Table S2. Bond lengths and angles for **28**

Bond	d, Å	Bond	d, Å
Cl(1)-C(1)	1.8159(17)	O(1)-C(2)	1.4257(19)
O(2)-C(3)	1.4365(19)	O(3)-C(12)	1.208(2)
O(4)-C(14)	1.432(2)	C(1)-C(10)	1.514(2)
C(1)-C(2)	1.535(2)	C(2)-C(3)	1.547(2)
C(3)-C(11)	1.521(2)	C(3)-C(4)	1.546(2)
C(4)-C(12)	1.523(2)	C(4)-C(5)	1.537(2)
C(5)-C(6)	1.530(2)	C(5)-C(10)	1.531(2)
C(6)-C(7)	1.526(2)	C(7)-C(8)	1.524(3)
C(8)-C(9)	1.522(2)	C(8)-C(15)	1.526(2)
C(9)-C(10)	1.528(2)	C(13)-C(14)	1.507(2)
C(12)-C(13)	1.503(2)	O(5)-H(12)	0.93(3)
O(5)-H(11)	0.81(3)		
**Angle**	**ω,°**	**Angle**	**ω,°**
C(10)-C(1)-Cl(1)	111.69(11)	C(2)-C(1)-Cl(1)	110.14(11)
O(1)-C(2)-C(1)	105.70(13)	O(1)-C(2)-C(3)	109.12(13)
C(1)-C(2)-C(3)	114.48(12)	O(2)-C(3)-C(11)	104.26(13)
O(2)-C(3)-C(4)	108.58(13)	C(11)-C(3)-C(4)	114.16(13)
O(2)-C(3)-C(2)	107.91(12)	C(11)-C(3)-C(2)	111.81(14)
C(4)-C(3)-C(2)	109.77(13)	C(12)-C(4)-C(5)	111.01(12)
C(12)-C(4)-C(3)	110.68(13)	C(5)-C(4)-C(3)	113.35(13)
C(6)-C(5)-C(10)	109.67(14)	C(6)-C(5)-C(4)	110.54(13)
C(10)-C(5)-C(4)	109.11(12)	C(7)-C(6)-C(5)	112.65(14)
C(8)-C(7)-C(6)	111.29(14)	C(9)-C(8)-C(7)	109.49(14)
C(9)-C(8)-C(15)	110.91(15)	C(7)-C(8)-C(15)	111.57(15)
C(8)-C(9)-C(10)	111.92(14)	C(1)-C(10)-C(9)	113.07(13)
C(1)-C(10)-C(5)	112.16(13)	C(9)-C(10)-C(5)	112.67(13)
O(3)-C(12)-C(13)	121.42(15)	O(3)-C(12)-C(4)	121.57(15)
C(13)-C(12)-C(4)	116.95(14)	C(12)-C(13)-C(14)	114.09(14)
O(4)-C(14)-C(13)	111.47(14)	H(11)-O(5)-H(12)	111(2)
C(10)-C(1)-C(2)	112.76(13)		

Table S3. Hydrogen bonds for **28**

D-H···A	d(D-H)	d(H···A)	d(D···A)	<(DHA)
O(1)-H(1)···O(2) ^{i}	0.84	1.84	2.6787(16)	171.6
O(2)-H(2)···O(5)^{ii}	0.84	1.94	2.760(2)	164.8
O(4)-H(4)···O(1)^{iii}	0.84	1.97	2.7361(17)	151.8
C(10)-H(10)···O(3)^{iv}	1.00	2.51	3.207(2)	126.7
C(11)-H(11B)···Cl(1)	0.98	2.72	3.2593(18)	115.2
C(13)-H(13A)···Cl(1)^{v}	0.99	2.98	3.8515(17)	147.4
C(13)-H(13B)···O(5)^{ii}	0.99	2.57	3.432(2)	146.0
O(5)-H(11)···O(4)^{vi}	0.81(3)	2.01(3)	2.7931(19)	162(3)
O(5)-H(12)···O(4)^{vii}	0.93(3)	1.94(3)	2.8166(19)	156(2)

Symmetry transformations: (i) x+1/2,-y+3/2,-z+2; (ii) x-1/2,-y+3/2,-z+1; (iii) x-1,y,z; (iv) x+1,y,z; (v) x,y+1,z; (vi) x+1,y-1,z-1; (vii) x+3/2,-y+3/2,-z+1.

Figure S138. A fragment of a layer of stacked and interconnected hydrogen bonds O–H···O, C–H···Cl and C–H···O molecules $C_{15}H_{25}ClO_4$ and H_2O, depicted in two projections: parallel to the plane (011) – **a** and (110) – **b**

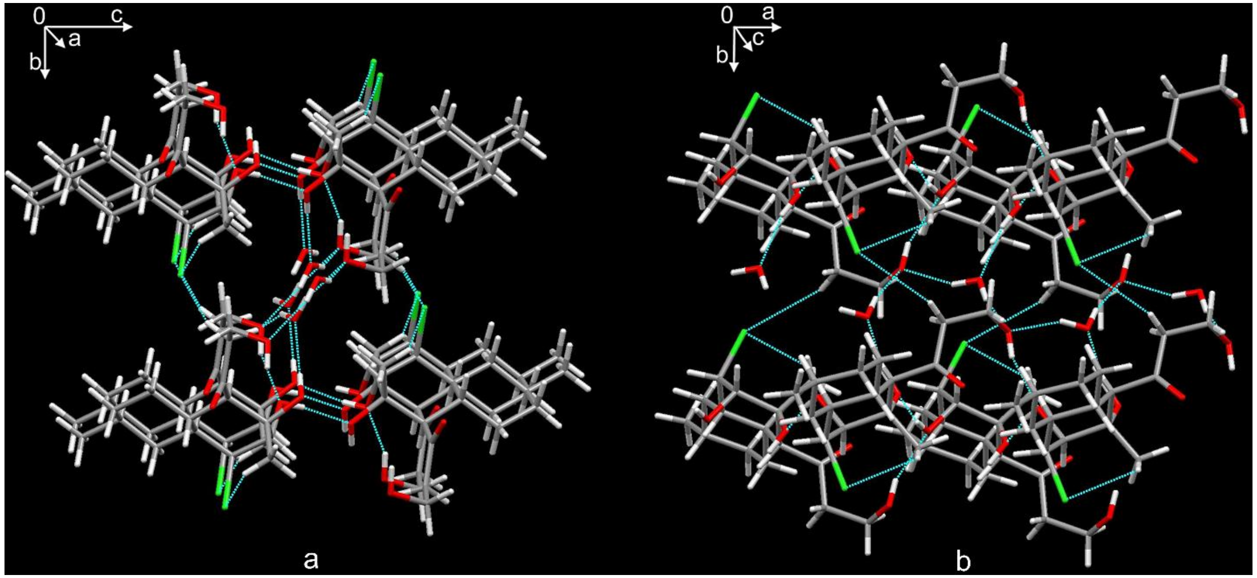

Figure S139. The crystal structure of $C_{15}H_{25}ClO_4 \cdot H_2O$

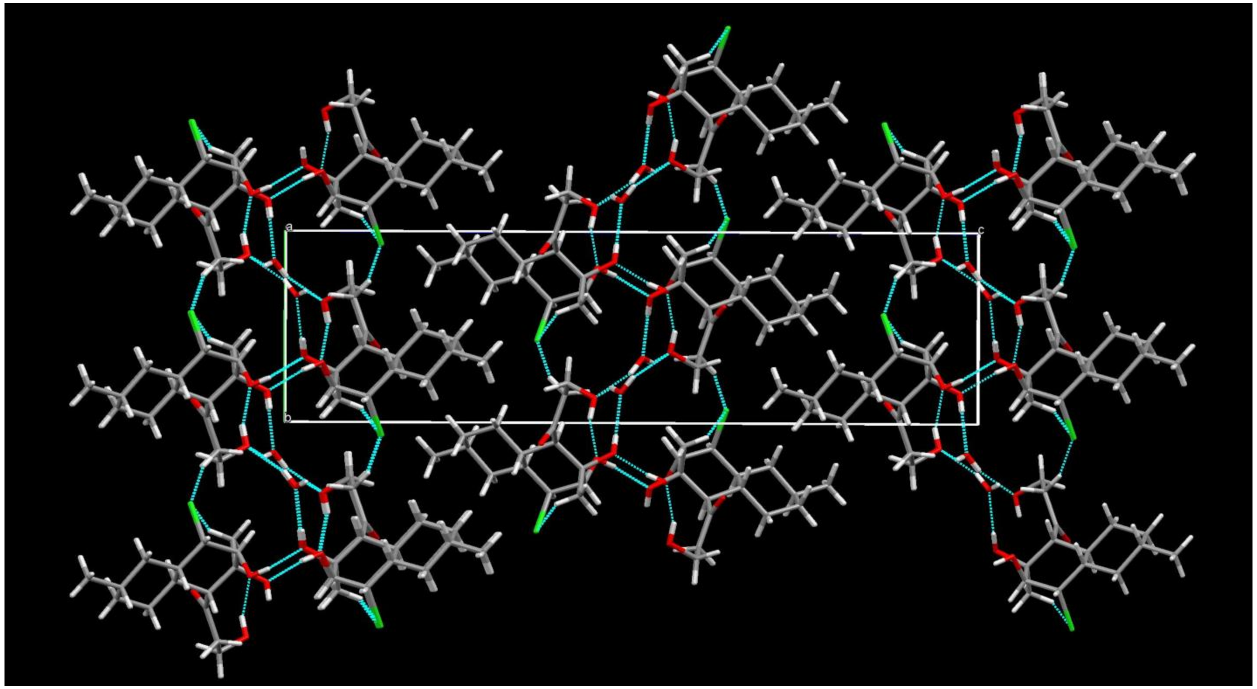

Table S4. Calculated and experimental chemical shifts (δ_C/ppm) for compound **1**

Atom	$\delta_{C, exp}$	$\delta_{C, calc.}$ 12-*R*	ΔC, 12-*R*	$\delta_{C, calc.}$ 12-*S*	ΔC, 12-*S*
1	61.6	66.2	4.6	66.9	5.3
2	37.2	42.2	5.0	41.8	4.6
3	207.3	217.5	10.2	217.6	10.3
4	58.5	70.5	12.0	64.8	6.3
5	41.2	48.5	7.3	48.7	7.5
6	25.9	31.0	5.1	30.9	5.0
7	37.7	39.9	2.2	40.0	2.3
8	68.9	75.8	6.9	75.8	6.9
9	37.2	43.0	5.8	42.3	5.1
10	41.0	53.3	12.3	46.9	5.9
11	203.9	209.9	6.0	215.1	11.2
12	62.8	85.2	22.4	74.2	11.4
13	82.4	92.5	10.1	91.5	9.1
14	19.7	23.0	3.3	21.5	1.8
15	31.6	33.0	1.4	33.1	1.5

Theoretical data were obtained with B3LYP/6-311G(d)_PCM// B3LYP/6-311G(d)_PCM method for chloroform solutions

Figure S140. Dependence of the δ_C calculated and δ_C experimental chemical shifts for compound **1**

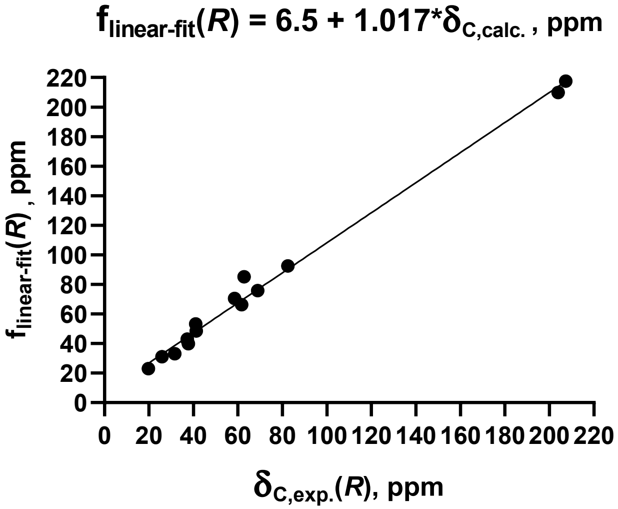

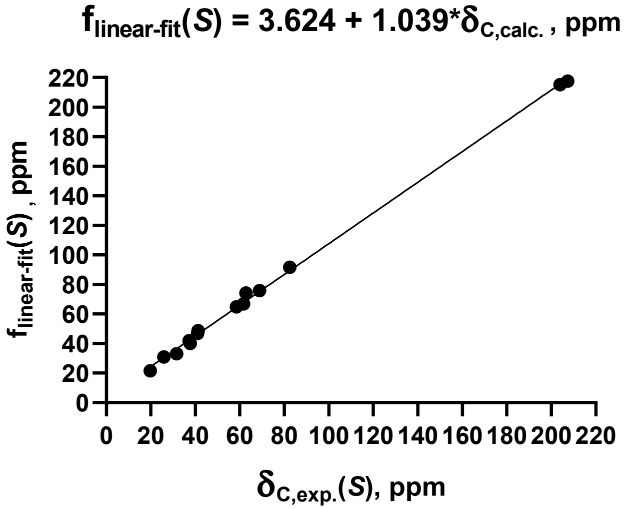

Figure S141. Comparison of calculated values for two conformers (12*R* and 12*S*) for compound **1**

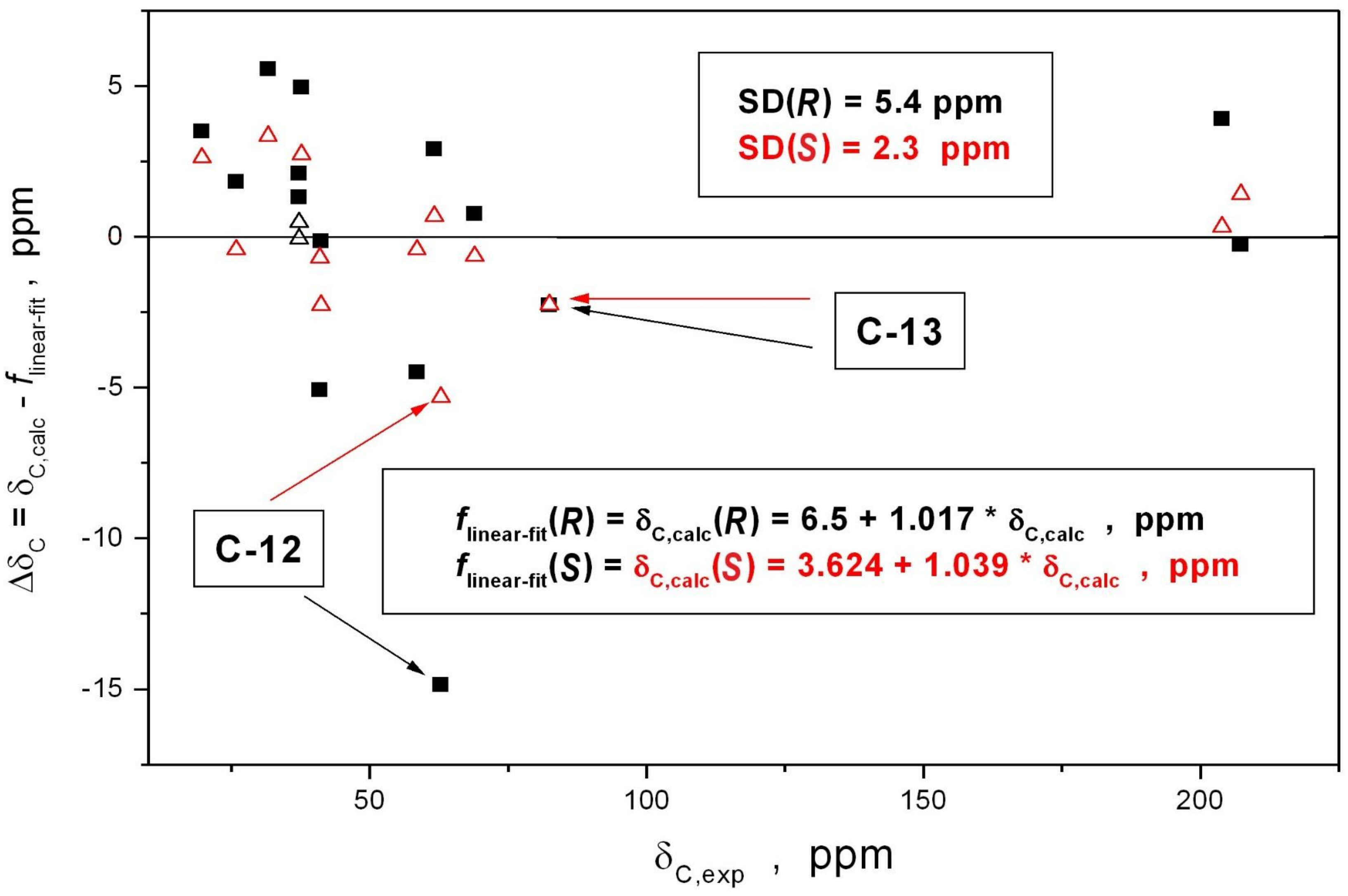

Figure S142. HPLC of fungal extracts cultivated on Rice Media (RM) and RM + $MgCl_2$

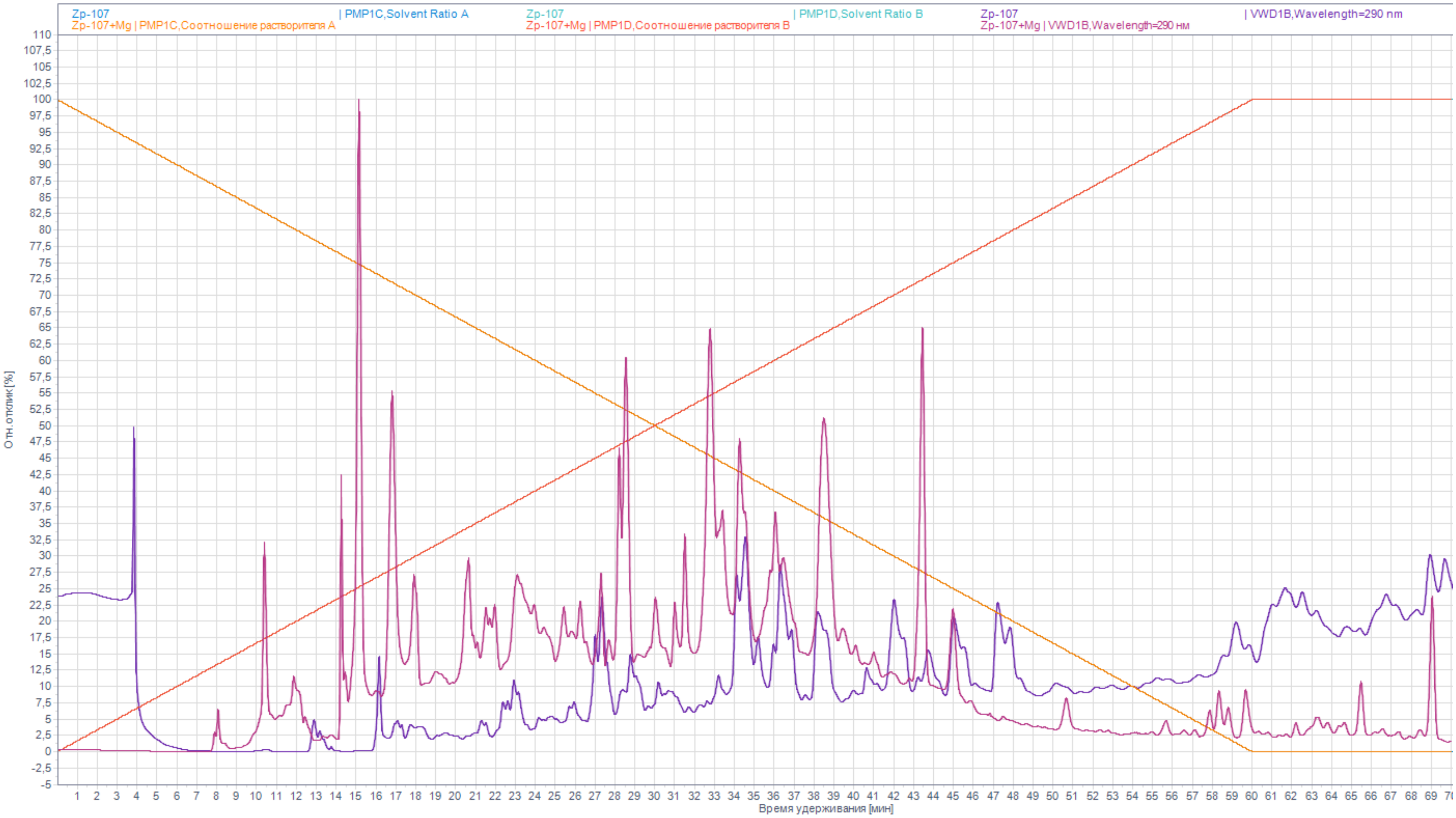

Supplement: Supplementary file 1 [file marinedrugs-24-00193-s001.zip › marinedrugs-4306897-supplementary.pdf]
